# H-Rubies, a new family of red emitting fluorescent pH sensors for living cells†
†Electronic supplementary information (ESI) available: Details of the organic synthesis, characterisations (HPLC, NMR, mass spectra) as well as measurements of their photophysical properties can be found in the supplementary information. See DOI: 10.1039/c5sc01113b


**DOI:** 10.1039/c5sc01113b

**Published:** 2015-07-14

**Authors:** Guillaume Despras, Alsu I. Zamaleeva, Lucie Dardevet, Céline Tisseyre, Joao Gamelas Magalhaes, Charlotte Garner, Michel De Waard, Sebastian Amigorena, Anne Feltz, Jean-Maurice Mallet, Mayeul Collot

**Affiliations:** a Laboratory of Biomolecules (LBM) , UPMC Université Paris 06 , Ecole Normale Supérieure (ENS) , CNRS, UMR 7203 , Paris F-75005 , France . Email: mayeul.collot@unistra.fr; b Ecole Normale Supérieure , Institut de Biologie de l'ENS (IBENS) , INSERM U1024 , CNRS UMR 8197 , Paris F-75005 , France; c Inserm U836 , LabEx Ion Channels, Science and Therapeutics , Grenoble Institute of Neuroscience , chemin fortuné ferrini, bâtiment Edmond Safra , 38042 Grenoble Cedex 09 , France; d Université Joseph Fourier , Grenoble , France; e Smartox Biotechnology , Saint Martin d’Hères , France; f INSERM U932 , Institute Curie , 75248 , Paris, Cedex 05 , France

## Abstract

Monitoring intracellular pH has drawn much attention due to its undeniably important function in cells. The widespread development of fluorescent imaging techniques makes pH sensitive fluorescent dyes valuable tools, especially red-emitting dyes which help to avoid the overcrowded green end of the spectral band. Herein, we present H-Rubies, a family of pH sensors based on a phenol moiety and a X-rhodamine fluorophore that display a bright red fluorescence upon acidification with p*K*_a_ values spanning from 4 to 9. Slight structural modifications led to dramatic changes in their physicochemical properties and a relationship between their structures, their ability to form *H*-aggregates, and their apparent p*K*_a_ was established. While molecular form H-Rubies can be used to monitor mitochondrial acidification of glioma cells, their functionalised forms were linked *via* click chemistry to dextrans or microbeads containing a near infrared Cy5 (Alexa-647) in order to provide ratiometric systems that were used to measure respectively the phagosomal and endosomal pH in macrophages (RAW 264.7 cells) using flow cytometry.

## Abstract 2

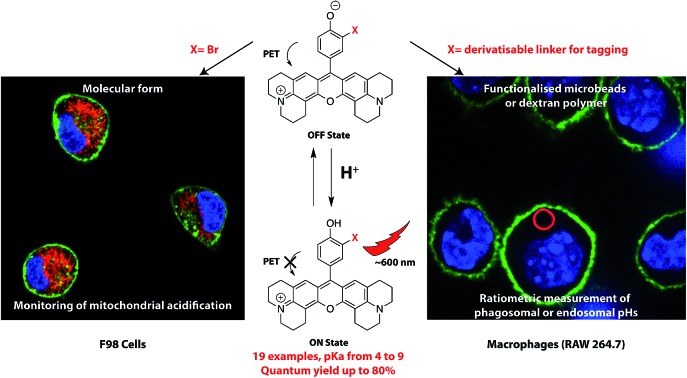
H-Rubies is a family of pH probes that display a bright red fluorescence upon acidification. They have been used as molecular form to monitor mitochondrial acidification and as functionalised forms to provide ratiometric systems to measure phagosomal and endosomal pH in macrophages.

## Introduction

Intracellular pH plays many different and important roles in cellular activity and is especially involved in ion transport,^1^ apoptosis,^2^,^3^ multidrug resistance^4^ and muscle contraction.^5^,^6^ Moreover, abnormal intracellular pH values are also associated with diseases such as Alzheimer's^7^ and cancer.^8^,^9^ Monitoring variations of intracellular pH in living cells is therefore of the utmost importance and is an essential parameter for studying phagocytosis,^10^ endocytosis^11^ and consequently cellular internalisation pathways. Fluorescence spectroscopy and fluorescence imaging techniques have many advantages including high sensitivity, high spatial and temporal resolution and are not destructive to the cells. As such, molecular fluorescent pH indicators have become indispensable tools for observing pH changes in cells. Although many fluorescent indicators have already been developed (for an extensive review see: Han and Burgess),^12^ several factors have to be taken into account in order to achieve efficient use within cells, including solubility in biological media, hydrophobicity, photostability and brightness. Our recent efforts to develop functionalisable red emitting fluorescent sensors^13^–^15^ were driven by the increasing use of cells transfected with green or yellow fluorescent proteins (FPs), thus allowing multicolour imaging. Whilst recent progresses have been reported in the development of red emitting genetically encoded pH sensors,^16^ there is still a demand for efficient molecular red fluorescent indicators. Among red-shifted fluorescent pH sensors, cyanines suffer from a weak photo-stability. Benzoxanthene dyes like SNARF, act as ratiometric probes but generally suffer from weak quantum yields. In contrast, red-emitting BODIPYs have recently attracted special attention due to their photostability and high brightness.^17^,^18^ Having said this they tend to suffer from high lipophilicity making them inefficient for cellular experiments as their apparent p*K*_a_ shifts in lipophilic environment.^19^

In his review, Han *et al.*^12^ pointed out that despite their advantageous spectral properties, pH probes based on rhodamine were not extensively developed.^20^ Rhodamine-based pH probes mostly rely on the spirolactam ring opening principle, generally yielding pH sensors with high dynamic ranges but low p*K*_a_ values as well as high hydrophobicity due to their neutrally charged OFF state.^21^–^26^

A key criteria for a pH sensor is its p*K*_a_ value, which should be in the physiological range. While the cytosol and some other cellular compartments, such as the nucleus and endoplasmic reticulum maintain their pH at ∼7.2, endosomes, lysosomes and secretory vesicles on the other hand are acidic environments with the pH ranging from 4.5 to 6.7. Conversely, alkaline pH can be found in mitochondria (∼8),^27^ phagosomes of dendritic cells^28^,^29^ and neutrophils.^30^ As such, a collection of pH probes with a large range of p*K*_a_ values is required in order to extend the scope of their application and thus a straightforward method of tuning their p*K*_a_ values without tedious chemical modifications would be a necessity. Finally, the large majority of fluorescent pH sensors do not offer an orthogonal and specific site of attachment required for functionalisation. Many common pH probes are too lipophilic and tend to compartmentalise in hydrophobic domains of the cell, however, to date no alternative methods of functionalisation have been proposed. In this report we present the development of a new family of red-emitting pH probes based on X-rhodamines and a phenol moiety as the pH reporter (see abstract scheme). In addition to their p*K*_a_ values ranging from ∼4 to ∼9, these ON–OFF type pH sensors, based on the PET (photoinduced electron transfer) quenching phenomena, exhibit high brightness in their acidic phenol form and can display very high turn ON of their fluorescence thanks to the formation of dark soluble *H*-aggregates in their OFF state. Among this new family, a number of H-Rubies were designed to bear a linker allowing the functionalisation of dextran and microbeads in order to measure the pH in living cells.

## Results and discussion

### Synthesis of phenolic X-rhodamines

**Fig. 1:**
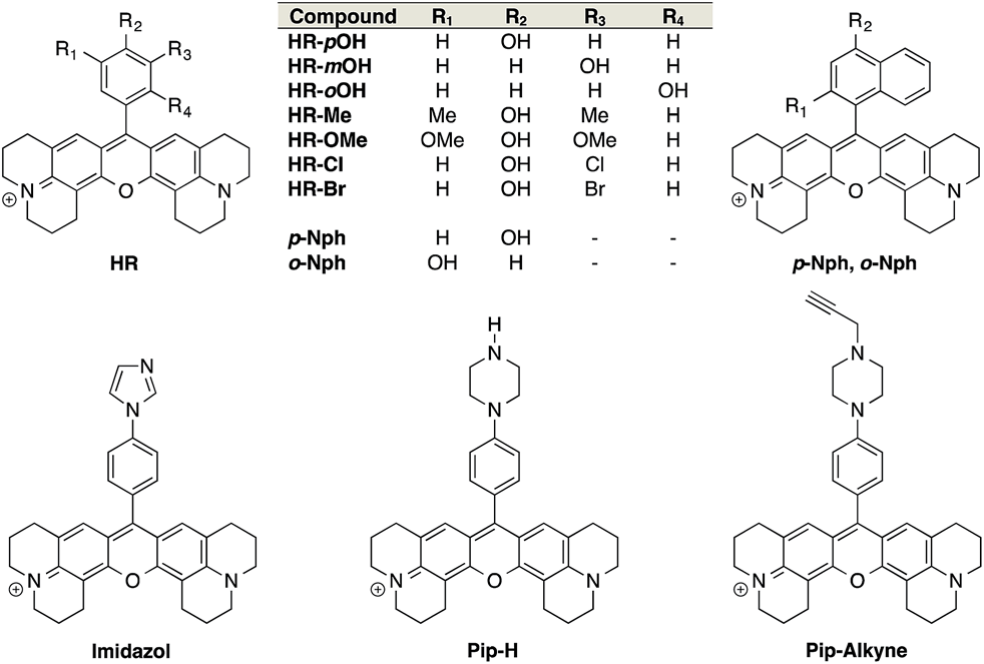
Structures of the synthesised X-rhodamines and the first set of H-Rubies.

ON–OFF pH probes generally require a pH-sensitive moiety whereby protonation/deprotonation modifies the quantum yield of a linked fluorophore either through PET (Photoinduced Electron Transfer) quenching phenomena or ICT (Internal Charge Transfer). Among these pH-sensitive moieties, phenol is the most popular as illustrated by the wide use of fluorescein and its derivatives: FITC, BCECF, *etc.* Moreover, the p*K*_a_ of phenols can be easily lowered and tuned to a biologically relevant range by introducing proper electron-withdrawing groups on the phenyl ring. Boens' group successfully converted an *ortho*-chlorophenol to obtain a pH-sensitive BODIPY with a p*K*_a_ of 7.6.^31^ Only few examples of phenolic rhodamines are given in the literature^32^ and none of those were exploited as pH probes, leading us to investigate the potential efficiency of these entities as fluorescent pH sensors. The red-emitting fluorophore X-rhodamine was chosen for its highly desirable spectral properties including a high molar absorption, high quantum yield and good photo-stability as well as its reduced hydrophobicity compared to BODIPY. Thus, an initial set of 9 X-rhodamines was synthesised from hydroxybenzaldehydes and hydroxynaphthaldehydes (for synthesis see ESI†). The use of phenyl-imidazol and (*N*-alkylated)-phenyl-piperazine^33^,^34^ as alternative pH-sensitive indicators was also explored giving rise to X-rhodamines: **Imidazole**, **Pip-H** and **Pip-Alkyne** (Fig. 1).

The spectral properties and p*K*_a_ values obtained for this first set of synthesised fluorophores are given in Table 1. Although X-rhodamine Imidazole did not behave as a fluorescent pH sensor (no significant variation of fluorescence intensity upon protonation), phenyl-piperazine and phenol-based X-rhodamines displayed clear pH sensitivity. Despite its physiologically relevant p*K*_a_ value and the capacity to be functionalised, **Pip-Alkyne** was not developed further as its dynamic range (3-fold) was found to be much lower than those obtained with phenol-based X-rhodamines.

**Table 1:** Spectral and physico-chemical properties of the first set of synthesised X-rhodamines (5 μM in MOPS 30 mM, 100 mM KCl)^*a*^

Compound		λabs_max_ (nm)	λem^*d*^ (nm)	Log *ε* (λabs_max_) (M^–1^ cm^–1^)	*Φ*	*Φ*_ON_/*Φ*_OFF_	Dynamic range^*e*^	Dynamic range @ pH ± 1 p*K*_a_^*f*^	p*K*_a_
**HR-*p*OH**	ON^*b*^	576	587	4.78	0.55	9	34	30	8.97
	OFF^*c*^	579^*f*^	598	4.53	0.06				±0.05
**HR-*m*OH**	ON^*b*^	578	602	4.70	0.30	3	132	75	9.33
	OFF^*c*^	577	603	4,63	0.11				±0.13
**HR-*o*OH**	ON^*b*^	581	603	4.89	0.75	3	866	460	9.68
	OFF^*c*^	579	603	4.69	0.27				±0.17
**HR-Me**	ON^*b*^	574	597	4.71	0.09	High	955	833	8.75
	OFF^*c*^	N/A^*g*^	598	N/A^*g*^	N/A^*h*^				±0.16
**HR-OMe**	ON^*b*^	577	598	4,72	0.22	8	302	N/A^*i*^	N/A^*i*^
	OFF^*c*^	576	600	4.74	0.03				
**HR-Cl**	ON^*b*^	579	601	4.68	0.47	High	753	512	6.17
	OFF^*c*^	544	599	4.52	N/A^*h*^				±0.10
**Hr-Br**	ON^*b*^	581	601	4.39	0.55	High	384	291	6.51
	OFF^*c*^	544	600	4.40	N/A^*h*^				±0.10
***p*-Nph**	ON^*b*^	581	600	4.79	0.28	6	23	9.7	8.51
	OFF^*c*^	579	602	4.34	0.05				±0.17
***o*-Nph**	ON^*b*^	584	604	4.43	0.77	8	337	166	8.73
	OFF^*c*^	576	604	4.46	0.10				±0.17
**Pip-H**	ON^*b*^	578	600	4.88	0.16	4	6	3.9	8.67
	OFF^*c*^	579	601	4.84	0.04				±0.07
**Pip-Alkyne**	ON^*b*^	577	600	4.98	0.34	2	3	1.9	4.95
	OFF^*c*^	579	602	4.45	0.15				±0.09
**Imidazole**	ON^*b*^	583	609	4.80	0.40	N/A^*j*^	N/A^*j*^	N/A^*j*^	N/A^*j*^
	OFF^*c*^	584	608	N/A^*j*^	N/A^*j*^				

^*a*^ *ε* = molar extinction coefficient, *λ* = wavelength, *Φ* = quantum yield.

^*b*^Protonated form: pH 4.

^*c*^Deprotonated form: pH 10.

^*d*^Excitation at 535 nm.

^*e*^[(*I*_max_ – *I*_min_)/*I*_min_] with *I* = fluorescence intensity.

^*f*^Fluorescence enhancement between ±1 pH unit around the p*K*_a_ value.

^*g*^Broad absorbance.

^*h*^Non-fluorescent.

^*i*^p*K*_a_ value was too high.

^*j*^No pH sensitivity.

### Structure/properties relationship of H-Rubies

**Fig. 2:**
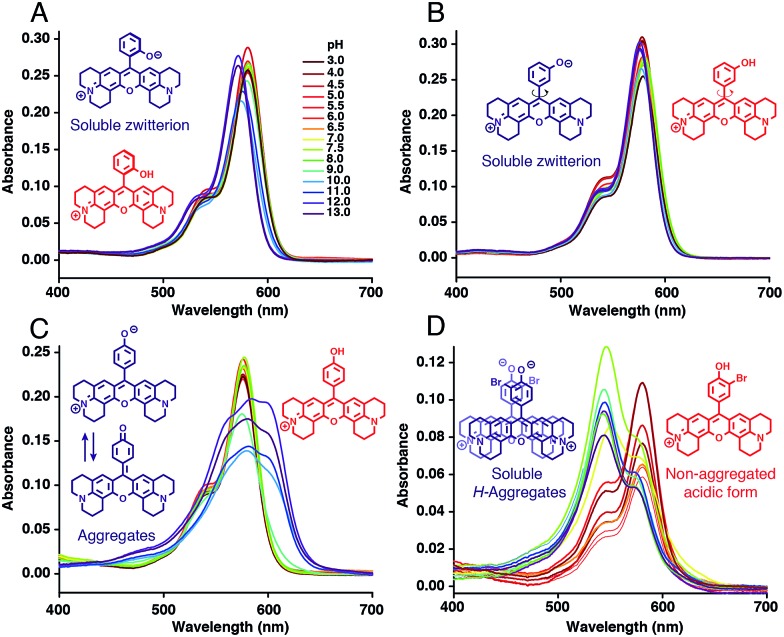
Absorption spectra of (A) **HR-*o*OH**, (B) **HR-*m*OH**, (C) **HR-*p*OH** and (D) **HR-Br** at 5 μM in MOPS 30 mM, 100 mM KCl at different pH and their basic form (blue) and acidic form (red) structures.

Absorption spectra of H-Rubies at different pH values provided us with information regarding their aggregation states (Fig. 2) since rhodamines can form different patterns of aggregations including *H*- and *J*-aggregates.^35^,^36^ The observed broadening of absorption spectra for **HR-*p*OH** clearly indicated the formation of non-defined aggregates at high pH values. This could be explained by the equilibrium between the zwitterionic phenolate form and the neutral, hydrophobic and planar quinone form (Fig. 2C). With regard to its isomers **HR-*m*OH** and **HR-*o*OH**, the neutral quinone forms cannot be formed due to the impossible delocalization of the electrons in the **HR-*m*OH** form and a twisted structure imposed by hindered sterical effect in **HR-*o*OH**; therefore, their absorption spectra only display slight changes upon deprotonation with no sign of aggregation (Fig. 2A and B). Interestingly, absorption spectra of **HR-Br** at pH values above its p*K*_a_ exhibited a second band indicating formation of soluble *H*-aggregates (Fig. 2D). Basic forms of **HR-Cl** and **HR-Br** both form *H*-aggregates, the stabilisation of which could be attributed to intermolecular halogen bonding^37^,^38^ between the electron-rich oxygen of the phenolate and the halogen atom forming typical head to head *H*-aggregate dimers. It is also noted that H-Rubies that forms *H*-aggregates in their basic forms have significantly enhanced acidity. For example, **HR-Br** is 1000-times more acidic than **HR-*p*OH**. This difference in acidity cannot be exclusively attributed to the electronic withdrawing effect of the bromine atom. These results tend to suggest that *H*-aggregation stabilises the basic forms of H-Rubies and hence, enhances their acidity. In this line of thought, since aggregation is concentration dependent, absorbance spectra and titration curves of **HR-Br** were measured at different concentrations of dye ranging from 500 nM to 5 μM (see Fig. S3 and S4†). The results showed that aggregation of basic form occurred at concentration above 500 nM and impacted the p*K*_a_ which varied from 6.5 at 5 μM to 7.3 at 500 nM. Moreover at a concentration of 500 nM where no aggregation of the basic form occurs the fluorescence enhancement was diminished as the OFF state was only due to the PET quenching phenomenon. *H*-aggregates are virtually non-fluorescent and thus, for the H-Rubies that are able to undergo this type of aggregation, this property can confer impressive levels of fluorescence enhancement (dynamic range) between their basic and acidic forms making them promising pH sensor for bio-imaging purposes. By controlling the *H*-aggregation of fluorophores one can achieve highly sensitive fluorogenic systems with a pronounced turning ON of fluorescence upon de-aggregation. This principle has been successfully used for bioimaging of ligand–receptor interactions^39^ and was also applied in monitoring intracellular pH with fluorogenic polymers.^40^

### Synthesis of functionalised H-Rubies

**Fig. 3:**
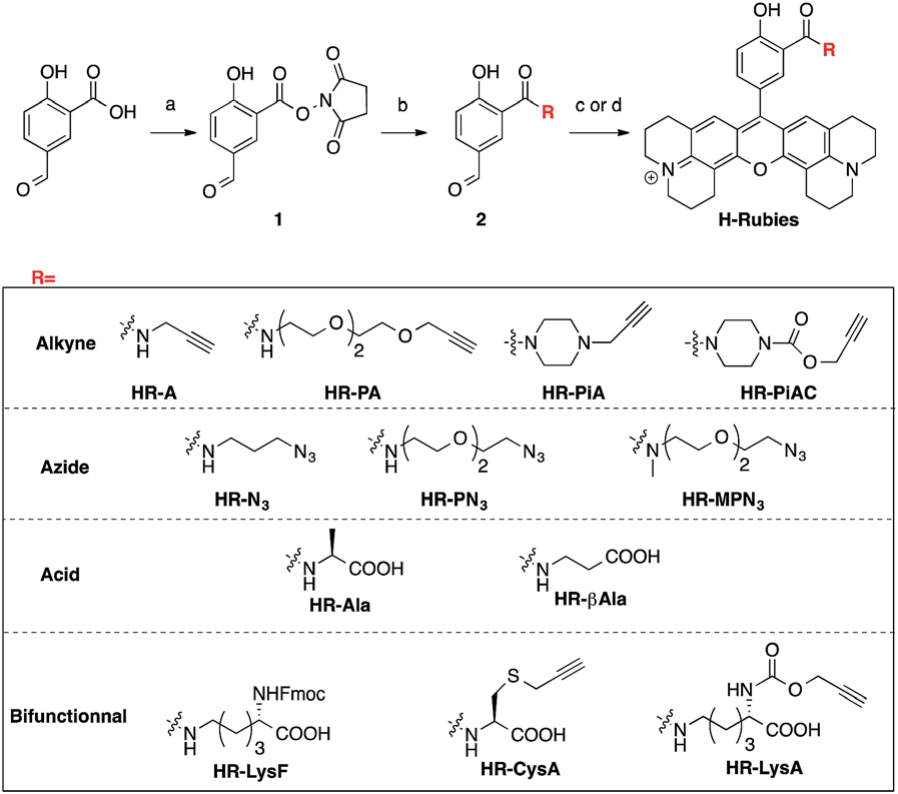
Synthesis and structures of the functionalisable H-Rubies: (a) NHS, DCC, THF; (b) amine, DIEA, DMSO; (c) 8-hydroxyjulolidine, PTSA, propionic acid then *p*-chloranil, DCM/MeOH (1 : 1); (d) 8-hydroxyjulolidine, TfOH, DCM, then *p*-chloranil.

Despite the first phenol-based X-rhodamines yielding excellent results as fluorescent pH probes, the importance of the ability to functionalise such fluorescent sensors must be emphasised and thus, the basic principle was pushed further in order to develop functionalisable H-Rubies. For this purpose, a linker was added at the *ortho* position of the phenol *via* an amide bond: a mild electron-withdrawing group that would ensure the conservation of a biologically relevant p*K*_a_. For this new set of functionalisable H-Rubies, 5-formylsalicylic acid **1** was converted into its activated ester **2** onto which were condensed primary and secondary amines bearing one or two orthogonal functions. Once the desirable aldehydes were obtained, they were transformed into their corresponding X-rhodamines with a custom optimized methodology allowing us to obtain the H-Rubies with yields of up to 93% (see ESI†). This new set of sensors can be categorised as follows: alkynes and azides, allowing linkage *via* bio-orthogonal Huisgen cycloaddition “click chemistry”; acids that can be coupled with amines and bifunctional H-Rubies, bearing two different functional groups (Fig. 3). Among the bifunctional H-Rubies that were developed is an Fmoc-protected Lysine H-Ruby (**HR-LysF**), which can be incorporated into a peptide sequence.

### Structure/properties relationship of functionalisable H-Rubies

Once again, it was noticed that slight modifications of the base structure led to dramatic changes in the photophysical properties of H-Rubies such as epsilon, quantum yields and p*K*_a_ values which ranged from 4 to 8 (Table 2). The most notable differences were observed between secondary and tertiary amides whereby secondary amides appeared much more acidic than tertiary amides. To emphasise this phenomena, we synthesised **HR-PN_3_** and its methylated tertiary amide equivalent **HR-MPN_3_** and showed that the latter was 60-times less acidic. Moreover, absorption spectra of these functionalisable H-Rubies clearly showed different behaviours of their basic forms in water. While deprotonated tertiary amide H-Rubies showed slight hypsochromic shifts of their maximum absorption wavelengths (1–5 nm), absorption spectra of secondary amide H-Rubies displayed a second absorption band (530 nm) indicating typical formation of soluble *H*-aggregates.

**Table 2:** Spectral and physicochemical properties of the functionalisable H-Rubies (5 μM in MOPS 30 mM, 100 mM KCl)

Compound		λabs_max_ (nm)	λem^*c*^ (nm)	Log *ε* (λabs_max_) (M^–1^ cm^–1^)	*Φ*	*Φ*_ON_/*Φ*_OFF_	Dynamic range^*d*^	Dynamic range @ pH ± 1 p*K*_a_^*e*^	p*K*_a_
**HR-A**	ON^*a*^	574	600	4.26	0.09	6	6	4.2	5.33
	OFF^*b*^	534	598	4.58	0.02				±0.13
**HR-PA**	ON^*a*^	580	602	4.87	0.46	1150	31	22	6.89
	OFF^*b*^	573	596	4.86	4 × 10^–4^				±0.04
**HR-PiA**	ON^*a*^	580	601	4.55	0.80	35	98	29	7.64
	OFF^*b*^	574	600	4.32	0.02				±0.10
**HR-PiAC**	ON^*a*^	580	601	4.86	0.64	40	86	45	7.68
	OFF^*b*^	575	602	4.69	0.02				±0.09
**HR-N_3_**	ON^*a*^	580	600	4.43	0.25	28	88	74	3.96
	OFF^*b*^	523	596	4.43	0.01				±0.03
**HR-PN_3_**	ON^*a*^	578	603	4.72	0.41	21	21	19	6.20
	OFF^*b*^	573	597	4.74	0.02				±0.03
**HR-MPN_3_**	ON^*a*^	578	605	5.20	0.65	65	78	66	8.00
	OFF^*b*^	573	603	5.20	0.01				±0.03
**HR-Ala**	ON^*a*^	577	601	4.81	0.15	8	9	8.3	7.85
	OFF^*b*^	573	597	5.03	0.019				±0.03
**HR-βAla**	ON^*a*^	577	602	5.10	0.20	11	11	N/A^*f*^	N/A^*f*^
	OFF^*b*^	572	598	5.30	0.018				
**HR-LysF**	ON^*a*^	583	607	4.73	0.11	37	45	33	4.84
	OFF^*b*^	580	600	4.66	0.003				±0.10
**HR-CysA**	ON^*a*^	577	602	4.69	0.09	N/A^*f*^	N/A^*f*^	N/A^*f*^	N/A^*f*^
	OFF^*b*^	572	597	5.08	0.014				
**HR-LysA**	ON^*a*^	577	602	4.75	0.25	13	13	11	7.51
	OFF^*b*^	572	596	4.96	0.02				±0.04

^*a*^Protonated form: pH 4.

^*b*^Deprotonated form: pH 10.

^*c*^Excitation at 535 nm.

^*d*^[(*I*_max_ – *I*_min_)/*I*_min_] with *I* = fluorescence intensity.

^*e*^Fluorescence enhancement between ±1 pH unit around the p*K*_a_ value.

^*f*^Complex titration curve.

**Fig. 4:**
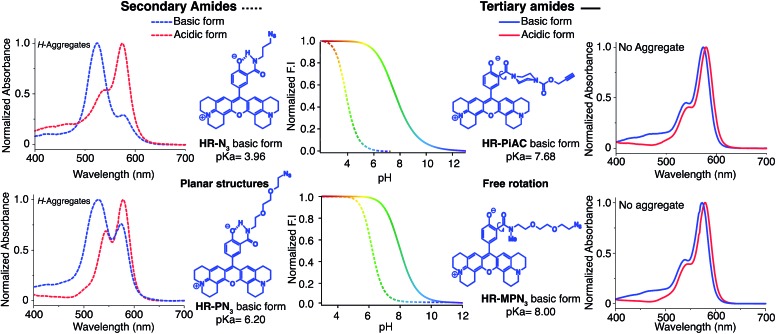
Difference of acidity and *H*-aggregation capability between secondary amide H-Rubies (dashed lines) and tertiary amide H-Rubies (plain lines). Concentration of probes was 5 μM in MOPS 30 mM, 100 mM KCl, excitation wavelength at 535 nm.

These differences can be explained by the formation of a H-bond between the phenolate and the proton of the secondary amide whereby the former is stabilised and hence, the p*K*_a_ is lowered (Fig. 4). Moreover, the six membered ring formed *via* H-bonding leads to a constrained conformation which aids the formation of planar, head-to-head *H*-aggregates. As seen in our initial observations, the acidity of H-Rubies is enhanced by their ability to form *H*-aggregates in their OFF state (Fig. 4).

### Imaging of acidic domains in living cells with molecular H-Rubies

**Fig. 5:**
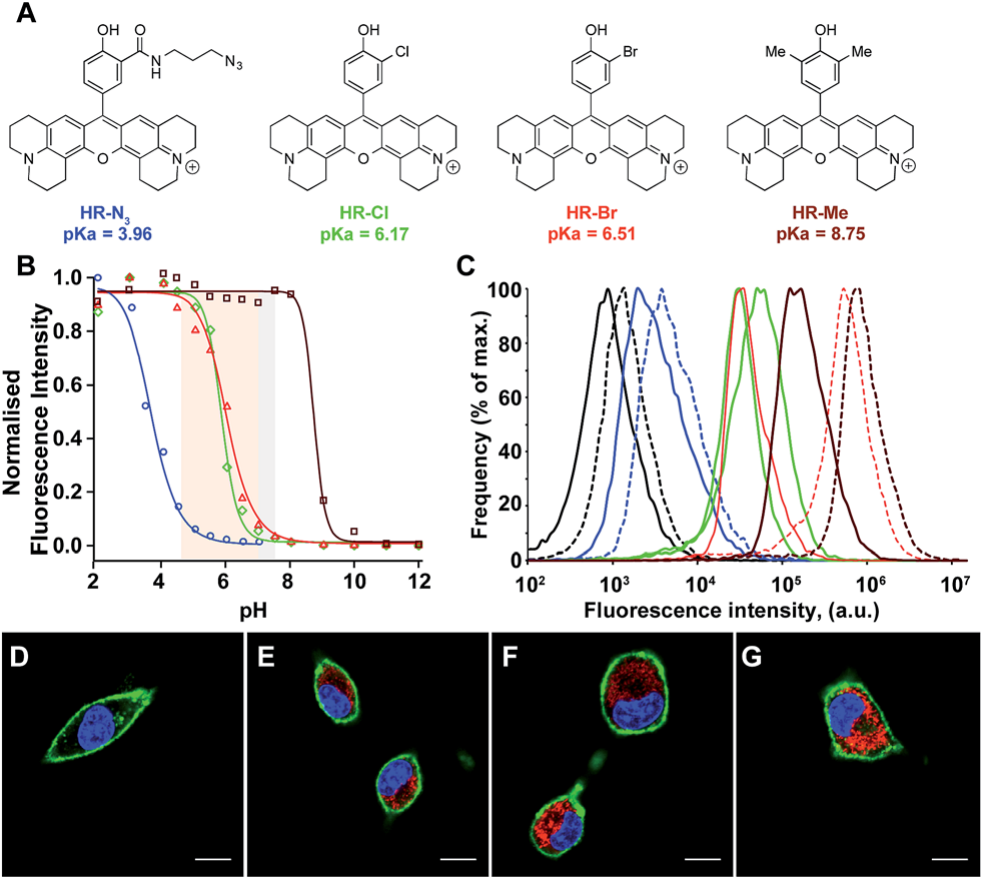
(A) Structures and p*K*_a_ values of the H-Rubies used for cellular experiments. (B) pH titration curves (Hill equation fit) of **HR-N_3_** (blue), **HR-Cl** (green), **HR-Br** (red) and **HR-Me** (brown) at 5 μM in MOPS (30 mM) KCl (100 mM), cytosolic and cellular acidic pH ranges are delimited with coloured areas. (C) Comparison of fluorescence intensity of F98 cells incubated for 20 min with 1 μM of **HR-N_3_** (blue), **HR-Cl** (green), **HR-Br** (red) and **HR-Me** (brown) at pH 7.4 (plain lines) and pH 6 (dashed lines) assessed by flow cytometry. Each curve is an average of 3 independent measurements. The control corresponds to the intrinsic fluorescence of cells (black line). Bottom: laser scanning confocal microscopy images of F98 cells incubated for 20 min with 1 μM of (D) **HR-N_3_**, (E) **HR-Cl**, (F) **HR-Br**, (G) **HR-Me**. The H-Rubies were visualized in red, the nuclei were stained with Hoechst (blue) and the membrane with Alexa 647-conjugated concanavalin A (green). Pictures show the three merged channels. Scale bars are 10 μm.

The impressive physical and optical properties of H-Rubies lead us to investigate their ability to image acidic domains in living cells. After establishing their insensitivity towards biologically significant metals (Zn^2+^, Mg^2+^, Ca^2+^, Fe^3+^, Mn^2+^) and their nontoxicity (see ESI Fig. S5† for LDH cytotoxicity assay), **HR-N_3_**, **HR-Me**, **HR-Cl** and **HR-Br** were involved in cellular experiments (Fig. 5A). These probes were specifically chosen for their respective p*K*_a_ values: **HR-Me** served as a positive control since its p*K*_a_ is more than two units above maximum physiological pH, **HR-N_3_** was expected to be a negative control since it has a p*K*_a_ of 4, whereas **HR-Cl** and **HR-Br** were expected to sense variations of pH within the cell (Fig. 5B). Firstly, Fischer's rat glioblastoma F98 cells (primary tumor) were incubated with the four dyes (1 μM) for only 20 min and visualised by confocal laser scanning microscopy. The obtained images indicated that these H-Rubies are cell-permeant and stain the mitochondria; this was confirmed by colocalisation experiments with mitotracker-green (see Fig. S6†). In a second time, flow cytometry assays with these cells showed a clear correlation between the observed fluorescence intensity of the cells and the measured p*K*_a_ of the probes (Fig. 5B and C plain lines). Finally, we checked the pH sensitivity of these H-Rubies upon cytosolic acidification. The intracellular pH was decreased to pH 6 and flow cytometry assays were performed (Fig. 5C dashed lines). The results showed that albeit **HR-N_3_** and **HR-Cl** displayed non-significant fluorescence enhancement (blue and green lines), **HR-Br** (orange line) displayed an impressive *in cellulo* response to this acidification (ΔpH = 1.4, fluorescence enhancement: up to 13-fold). **HR-Me** also showed an interesting fluorescence enhancement (∼5-fold) but behaved in an inconsistent manner when the intracellular pH was alkalinised (Fig. S7†). Despite **HR-Br** cannot be used to determine absolute pH values *in cellulo* because of the effect of its concentration on its p*K*_a_, its cell permeability combined with its high fluorescence enhancement in the physiological pH range make it an attractive tool for tracking mitochondrial acidification which is a important field in cell biology as it is involved in mitophagy that is associated to various pathologies including neurodegenerative and cardiovascular diseases.^41^,^42^

### Measurements of intraorganellar pH using functionalisable H-Rubies

**Fig. 6:**
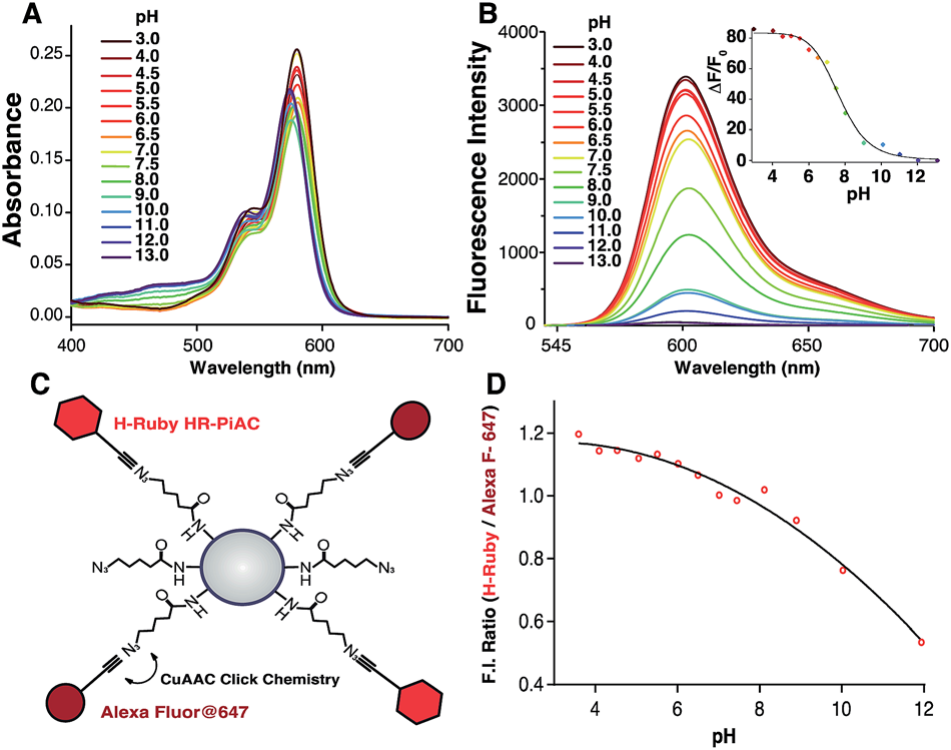
Absorption (A) and emission spectra (B) of **HR-PiAC** (5 μM, MOPS 30 mM, 100 mM KCl) at different pH (excitation wavelength = 535 nm), inset is the titration curve fitted with the Hill equation providing a p*K*_a_ of 7.68 ± 0.09 and an enhancement of fluorescence of ∼80-fold. Scheme of bead-based pH sensor, containing pH-sensitive dye **HR-PiAC** and pH-insensitive Alexa Fluor 647 (C); titration curve of the pH beads measured by flow cytometry (D).

To demonstrate the different biological applications of H-Rubies we developed two systems to measure sub-organellar pH in living cells. Our first approach aimed at the calibration of phagosomal pH in living cells using flow cytometry. As pH reporter, **HR-PiAC** was chosen for its useful properties in cellular experiments such as its high brightness, its high dynamic range (∼80-fold) and its p*K*_a_ value of 7.68 (Fig. 6A and B). 3 μm latex beads containing amino groups were combined with the bifunctional linker azido-PEG_4_-NHS ester followed by double functionalisation using click chemistry with **HR-PiAC** and a pH-insensitive near infrared emitting dye, Alexa Fluor 647, to allow ratiometric measurements (Fig. 6C). It was noticed that the covalent binding of two dyes on the surface of the beads influences the physicochemical properties of **HR-PiAC**, namely its dynamic range and its p*K*_a_. This effect has already been noticed in our previous work involving a molecular fluorescent Ca^2+^ sensor linked to a quantum dot^43^ and can be attributed to the proximity of the dye to the hydrophobic environment of the polystyrene bead that enhances the basal fluorescence of the sensor. Compared to the titration curve of the free dye, the fluorescence ratio of the two dyes obtained by flow cytometry shows an almost linear dependence when the pH is varied from 5 to 9. As a result, although these beads have a decreased sensitivity in the pH of physiological domain, it provides a ratiometric system which displays a pH sensitivity over a wider range when compared to a free **HR-PiAC** (Fig. 6D).

**Fig. 7:**
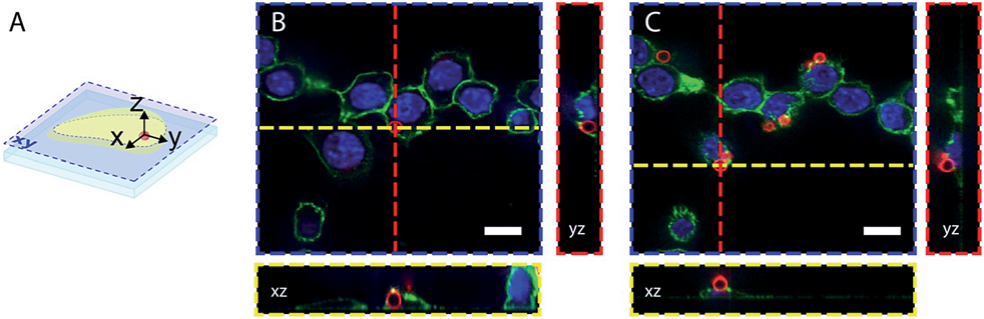
Confocal microscopy images of fixed RAW 264.7 cells after bead phagocytosis. DAPI nuclear staining, concanavalin-Alexa 488 membrane staining and pH beads are in blue, green and red respectively. (A) illustrates the orthogonal views of the cell that allow differentiation of the internalized beads (B) and the beads attached to the cell surface (C). Scale bars are 10 μm.

The dyes-modified beads were then added to a suspension of RAW 264.7 macrophages and incubated for 30 min at 37 °C to complete the process of phagocytosis. After several washes the cells were fixed and visualized by confocal microscopy to assess the efficiency of bead internalisation. Orthogonal view analysis of confocal sections shows partial internalisation of the beads by macrophages. As shown in Fig. 7, in addition to fully internalised beads, some beads are attached to the outer cell membrane. On average only 30% of beads were fully internalised by the cells. This could interfere with flow cytometric measurements of phagosomal pH because the laser-based technique in this case will identify all fluorescent objects, *i.e.* cells with internal and/or external beads. We, therefore, performed an additional modification of the beads by coating them with a biotinylated ovalbumin (OVA), *via* passive absorption. The coated beads, firstly, have a reduced non-specific binding to the cell surface and favour phagocytosis through the interaction between the mannosylated structure present on the surface of OVA^44^ and the mannose receptors of macrophages.^45^ Secondly, accessible biotin groups of OVA can be tagged by FITC-streptavidin.

**Fig. 8:**
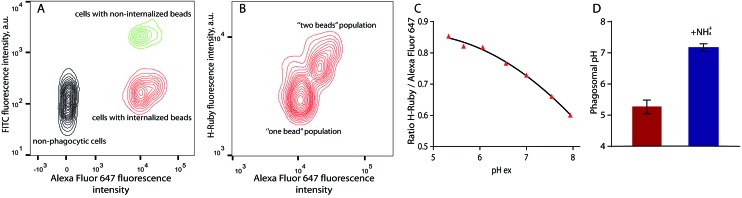
Phagosomal pH measurement in RAW 264.7 cells by flow cytometry. (A) Density plot of the cells after bead internalisation showing three distinct populations: non-phagocytic cells, cells with internalised beads and cells with non-internalised beads on which a biotin – streptavidin–FITC interaction takes place; (B) the cells with only one internalised bead were used for analysis of phagosomal pH. (C) Typical calibration curve of H-Ruby/Alexa Fluor 647 ratio of the cells clamped at different pHs with the ionophore nigericin; (D) phagosomal pH (5.3 ± 0.2, in red) in RAW 264.7 cells measured after 3 hours of phagosomes maturation NH_4_Cl addition shows alkalinisation of phagolysosomes (7.2 ± 0.1, in blue). The data represent the mean of 3 independent experiments ± SD.

For phagosomal pH measurement by flow cytometry, adherent RAW 264.7 macrophages were incubated for 30 min at 37 °C with the OVA-modified beads and let rest for 3 more hours for phagosomal maturation. Afterwards the cells were harvested and incubated with streptavidin-FITC that only binds to the non-internalised beads containing OVA-biotin. Two populations of phagocytic and non-phagocytic cells can be differentiated by the fluorescence intensity of Alexa Fluor 647 of the beads. A cell population with beads attached to the cell surface can be separated by the higher fluorescence intensity of FITC at extracellular pH (Fig. 8A). Further detailed analysis of the phagocytic population shows the presence of subpopulations according to the fluorescence intensities of Alexa Fluor 647 and H-Ruby: cells that have internalised one bead per cell exhibit a lower fluorescence intensity (Fig. 8B). For precise analysis of phagosomal pH the cells were gated on one bead population and a ratiometric calibration curve of phagosomal pH was obtained changing the external pH over the 5 to 8 range using a citric acid/HEPES buffer and imposing the same pH intracellularly using nigericin, a K^+^/H^+^ ionophore (Fig. 8C). Finally, to determine the phagosomal pH, the ratio of the mean fluorescence intensities of HRuby *vs.* Alexa Fluor 647 obtained from the intact cells was interpolated to the calibration curve yielding a phagolysosomal pH of 5.3 ± 0.2. This result is in a good agreement with a previously published study where authors showed a phagosomal pH value of 5.22 ± 0.03 in RAW cells using FITC-sRBC particles.^46^ Addition of the weak base NH_4_^+^/NH_3_ caused fast alkalinisation, and demonstrated the intracellular localisation of the beads (Fig. 8D).

**Fig. 9:**
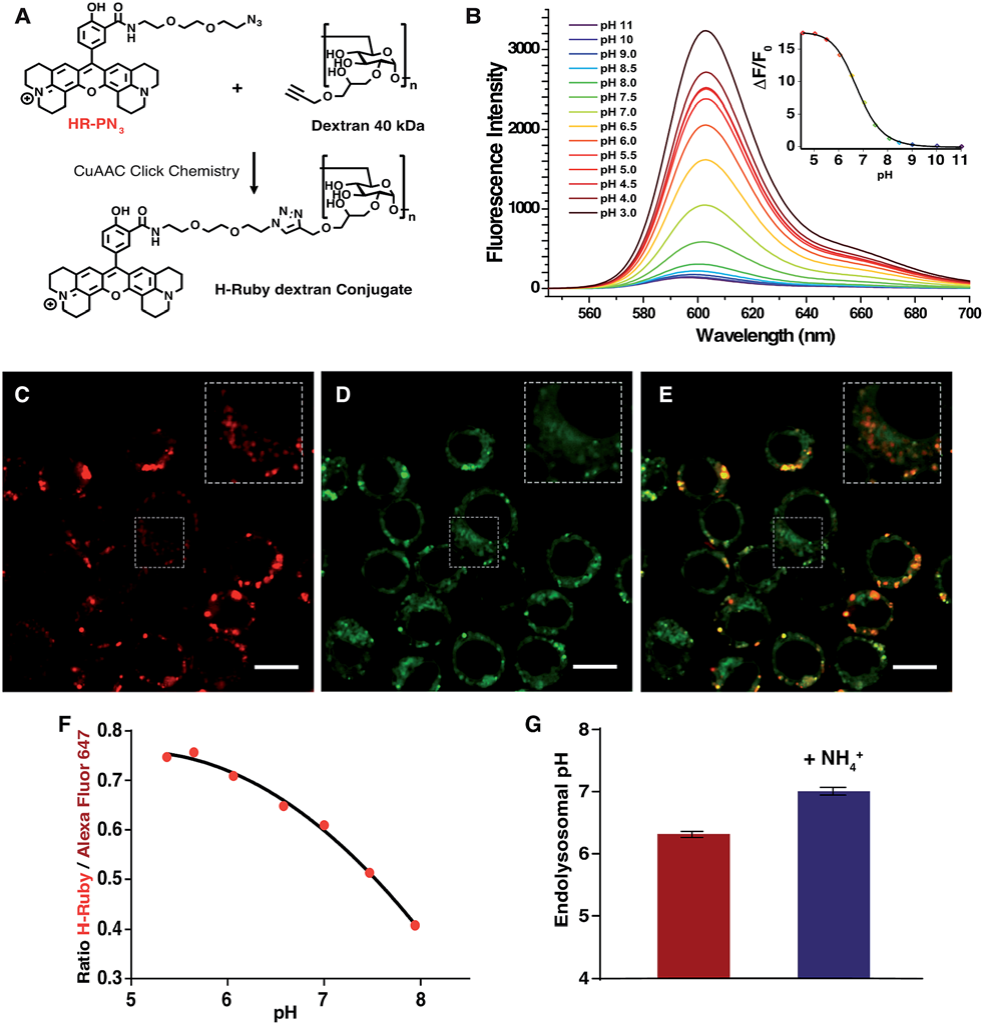
(A) Synthesis of **HR-PN_3_ dextran conjugate***via* CuAAC Click Chemistry. (B) Emission spectra of **HR-PN_3_ dextran conjugate** (MOPS 30 mM, 100 mM KCl) at different pH (excitation wavelength = 535 nm), inset is the titration curve fitted to the Hill equation yielding a p*K*_a_ of 6.77 ± 0.02 and an enhancement of fluorescence of ∼18-fold and 13 fold between ±1 pH unit around the p*K*_a_. (C–E) Confocal images of RAW 264.7 cells after incubation with 1 mg mL^–1^**HR-PN_3_ dextran conjugate** (C), co-stained by Lysotracker Green (D) and a merged image of two channels (E). **HR-PN_3_ dextran conjugate** labels lysosomes (in red) that colocalises with Lysotracker, and endosomes that are shown in insets and do not colocalise with Lysotracker. Scale bars are 10 μm. (F) Typical calibration curve of H-Ruby/Alexa Fluor 647 ratio of the cells clamped at different pHs with the ionophore nigericin. (G) Endosomal pH (6.3 ± 0.05, in red) in RAW 264.7 cells measured after 3 hours of incubation with **HR-PN_3_ dextran**. NH_4_Cl addition shows alkalinisation of endosomal organelles (7.0 ± 0.06, in blue). The data represent the mean of 3 independent experiments ± SD.

Another application of these newly synthesized H-Rubies is the calibration of endosomal pH by flow cytometry and to achieve this we used a polysaccharide dextran that is known to target the endolysosomal pathway.^47^ 40 kDa dextran was converted to an alkyne containing dextran and was subsequently clicked to **HR-PN_3_** (Fig. 9A). In comparison to its parent molecular form **HR-PN_3_**, the fluorescent pH-sensitive dextran displayed a slightly shifted apparent p*K*_a_ value (respectively 6.20 ± 0.03 and 6.77 ± 0.02; Fig. 9B) with a virtually non-fluorescent OFF state (see ESI†). Functionalisation did not significantly affect the fluorescence enhancement between pH 10 and 4 (respectively 21- and 19-fold) making this water-soluble polymer a valuable tool for monitoring endosomal pH. To demonstrate this, adherent RAW 264.7 cells were incubated for 30 min with **HR-PN_3_ dextran conjugate** and let rest for 3 more hours. As shown in Fig. 9C, vesicles of different shapes and sizes could be observed inside the cells due to the movement and segregation of the dextran molecules between the endocytic organelles.^48^ Addition of Lysotracker Green led to its partial colocalisation with **HR-PN_3_ dextran conjugate** (Fig. 9C–E) suggesting that **HR-PN_3_ dextran** accumulates both in the more acidic lysosomes and in the mildly acidic vesicles of the endocytic pathway as well. This observation is consistent with the flow cytometry experiments where, applying the same strategy as described above, the pH value of 6.3 ± 0.04 was obtained (Fig. 9F–G).

## Methods and materials

### LDH-cytotoxicity assay

CHO-K1 cells (20 000 per well) were seeded in 96 multiple well plates 24 h before the experiment. Cells (40 000 per well) were incubated with the fluorescent compounds in 100 μL DMEM for 2 hours at 37 °C. The incubation milieu was then replaced by 100 μL fresh DMEM containing 10% of cell-counting solution (Dojindo Laboratories) and incubated for a further 2 h at 37 °C. The absorbance measured at 450 nm had a direct relationship to the number of viable cells. Experiments were conducted in triplicate and repeated twice.

### Cell culture

Undifferentiated malignant glioma rat (F98) and Chinese Hamster Ovary (CHO) cell lines (from ATCC) were maintained at 37 °C in 5% CO_2_ in DMEM/F-12 nutrient medium (Invitrogen, Cergy Pontoise, France) supplemented with 2% (v/v) heat-inactivated fetal bovine serum (Invitrogen) and 100 μg mL^–1^ streptomycin and 100 units per mL penicillin (Invitrogen). RAW 264.7 macrophages cell line was maintained at 37 °C in 5% CO_2_ in DMEM nutrient medium (Gibco, Invitrogen) supplemented with 10% fetal calf serum (Eurobio, France).

### Confocal microscopy

Cell cultures were incubated with the pH probes (in DMEM/F-12 nutrient medium only) for 20 min, and then washed twice with phosphate-buffered saline (PBS) alone. After 4 hours, the nucleus were stained with 60 μg mL^–1^ Hoechst 34580 for 15 min, the cell cultures were then washed with PBS and the plasma membrane was stained with 30 μg mL^–1^ Alexa 647-conjugated concanavalin A and the mitochondria stained with 50 nM mitotracker green for 5 min (stainings were purchased at Invitrogen, Cergy Pontoise, France). Cells were washed once more. Live cells were then immediately analysed by confocal laser scanning microscopy using a Zeiss LSM operating system. Alexa 647 (633 nm), Hoechst 34580 (405 nm), pH probes (561 nm) and mitotracker green (488 nm) were sequentially excited and emission fluorescence was collected.

To assess the efficiency of bead internalisation, the cells, after completing the process of phagocytosis (as described in “Organellar pH measurement by flow cytometry”), were plated on coverslips (*d* = 12 mm) coated with 10 μg mL^–1^ fibronectin and incubated at 37 °C in 5% CO_2_: the cell membrane was stained with 25 μg mL^–1^ Alexa Fluor 488 – conjugated concanavalin (Invitrogen, USA) at 4 °C for 5 min and 4% paraformaldehyde was added for 20 min to fix the cells. The coverslips with the cells were then mounted on microscope slides using a mounting medium DAPI-Fluoromount G (Southern Biotech, USA) and left at room temperature overnight. Imaging was performed on a spinning disk microscope. The pH-sensitive beads were excited with 561 nm laser line and detected with ET630/75 nm filter, DAPI was excited with 405 nm laser line and detected with ET460/50 nm band-pass filter and Concanavalin A-Alexa Fluor 488 was excited with 488 nm laser line and detected with D505/40 nm band-pass filter. Images were collected using microscope software Software: MetaMorph 7.8.2.0. The subsequent analysis of the images was conducted on an open source image processing program ImageJ.

### Fluorescence activated cell sorting analyses – effect of the pHi on the fluorescence intensity of the H-Rubies

For the dose-response curve, F98 cells were incubated with various concentrations of the H-Rubies in DMEM/F-12 culture medium without serum at 37 °C for 2 h. The cells were then washed with PBS to remove excess extracellular H-Ruby and were then treated with 0.05% Trypsin–EDTA for 2 min at 37 °C to detach cells from the surface, and centrifuged at 200 g in DMEM/F-12 culture medium before suspension in PBS. Flow cytometry analyses were performed with live cells using an Accuri C6 flow cytometer (BD Biosciences, Le Pont de Claix, France). For acquisition a 488 nm wavelength laser was used for H-Rubies excitation, the fluorescence emissions were recorded in a 584 ± 20 nm spectral detection channel. Data were obtained and analyzed using CFlow Sampler (BD Biosciences). Live cells were gated by forward/side scattering for a total of 10 000 events.

The pHi of F-98 cells was modified using the pseudo-null calibration method describe by Chow *et al.*^49^ F98 cells were incubated with 1 μM of H-Rubies in DMEM/F-12 culture medium without serum at 37 °C for 20 min. The cells were then washed with PBS to remove excess extracellular probe and were then treated with 0.05% Trypsin–EDTA for 2 min at 37 °C to detach the cells from the surface, and centrifuged at 200 g in DMEM/F 12 culture medium before suspension in culture medium. Just before the analysis the pseudo null solution were added (ratio of culture medium: pseudo-null solution 1 : 1) and the data were acquired immediately (within 30 s). Pseudo-null solutions were made according to the method of Chow *et al.* Six times concentrated standard solution was made by adding 1 M ammonium hydroxide and 1 M acetic acid to the basal solution (culture medium). This provided a set of pseudo-null solution as shown in Table S1 (see ESI†). Flow cytometry analyses were performed with live cells using an Accuri C6 flow cytometer (BD Biosciences, Le Pont de Claix, France). Data were obtained and analyzed using CFlow Sampler (BD Biosciences). Live cells were gated by forward/side scattering for a total of 10 000 events.

### Functionalisation of the beads by H-Ruby

Polybead® Amino Microspheres 3.00 μm (Polysciences) were used. First the beads were washed with dH_2_O. In order to convert the NH_2_ groups on the beads surface to N_3_ groups for further click chemistry reactions, 100 nmol of the bifunctional linker N_3_–PEG_4_–NHS was added to 100 μL of the beads containing ∼20 nmol of NH_2_ groups and incubated at room temperature for 2 h. The alkyne-modified H-Ruby **HR-PiAC** and alkyne-containing Alexa Fluor 647 (Molecular Probes®) were then mixed with the bead solution in a ratio 1 nmol of NH_2_ group: 10 nmol of H-Ruby: 2.5 nmol of Alexa Fluor 647. 5 μL of 25 mM CuSO_4_·5H_2_O and 5 μL of 25 mM sodium ascorbate were added to initiate a click-reaction. The mixture was incubated overnight at room temperature. The dyes modified beads were successively washed with 0.1 M EDTA, 10% ethanol and PBS to remove non-reacted dyes.

### Organellar pH measurement by flow cytometry

Measurement of phagosomal or endosomal pH was performed using macrophages cell line RAW 264. The cells were plated on a Petri dish and at 80% confluence pH beads in a 4 : 1 ratio or 0.7 mg mL^–1^ 40 kDa **HR-PN_3_ dextran conjugate** and 0.3 mg mL^–1^ 40 kDa Alexa Fluor 647 dextran conjugate were added and incubated 30 min at 37 °C to complete internalisation. Afterwards the non-internalised beads/dextran were washed out, fresh medium was added and the cells were let rest at 37 °C, 5% CO_2_ for 3 more hours. For FACS analysis the cells were harvested and resuspended in CO_2_-independent medium (GIBCO, Invitrogen). For NH_4_^+^/NH_3_ tests, 20 mM NH_4_Cl was added to the extracellular medium and let to incubate for 3 min.

The calibration of phagosomal and endosomal pH was performed using the K^+^/H^+^ ionophore nigericin that, together with a high concentration of potassium in the buffer, equalises the intracellular and extracellular pH.^50^ The cells containing internalised pH beads or dextran were resuspended in buffers containing 143 mM KCl, 1.17 mM MgCl_2_, 1.3 mM CaCl_2_, 5 mM glucose and 10 μM nigericin with defined pH buffers ranging from pH 5.0 to pH 8.0 with 0.5 increments. 20 mM citric acid was used for a buffer range of pH 5.0–6.5 and 20 mM (4-(2-hydroxyethyl)-1-piperazineethanesulfonic acid) (HEPES) – for buffers in the pH range 7.0–8.0. 0.05 μM DAPI was added to exclude the dead cells. After incubation for 5 min at RT the samples were analyzed by a flow cytometer (BD LSR Fortessa, BD Biosciences). For acquisition, a 532 nm wavelength laser was used for H-Rubies excitation, and its emission fluorescence was recorded in a 610 ± 20 nm spectral detection channel. For Alexa Fluor 647 a 640 nm wavelength laser was used for excitation and a 670 ± 30 nm spectral detection channel to record its emission fluorescence. Each time 10 000 events were acquired. For analysis, a population of live cells was gated by forward/side scatter, and within this population only the cells which have phagocyted one pH bead were selected for precise pH determination.

## Conclusion

We have developed a new set of efficient red-emitting fluorescent pH sensors based on PET quenching phenomena. H-Rubies exhibit specific p*K*_a_ values and a structure/properties relationship based on their ability to form non-emissive dark *H*-aggregates was established. Four non-functionalisable H-Rubies were chosen for cellular experiments and were shown to cross the plasma membrane to compartmentalise in mitochondria. Among them, **HR-Br** displays a strong fluorescence enhancement upon acidification of mitochondria in live cells. Functionalisable H-Rubies were linked to a dextran polymer or to microbeads that were also decorated with a pH-insensitive dye leading to ratiometric pH probe systems. These systems were successfully used to measure phagosomal and endocytic pHs in live cells. The authors believe that this new family of fluorescent indicators, with their large choice of p*K*_a_ values and spectral properties specifically adapted to cellular imaging, represents a valuable toolkit for imaging pH variations in living cells. Moreover, their diverse functionalisable side arms allows them to be attached to particles, polymers and biomolecules such as peptides or antibodies for accurate pH readout of subcellular micro-domains.

## Supplementary Material

Supplementary information

Supplementary information

Supplementary information
