# Supplementary material for: H-Rubies, a new family of red emitting fluorescent pH sensors for living cells
Source: Chem Sci. 2015 Jul 14;6(10):5928–37. doi: 10.1039/c5sc01113b (PMC5950754; doi:10.1039/c5sc01113b)
Supplement: Supplementary file 1 [file SC-006-C5SC01113B-s001.pdf]

Electronic Supplementary Material (ESI) for Chemical Science.
This journal is © The Royal Society of Chemistry 2015

H-Rubies, a New Family of Red Emitting Fluorescent pH sensors for Living Cells

Guillaume Despras+, Alsu Zamaleeva+, Lucie Dardevet, Céline Tisseyre, Joao Gamelas Magalhaes, Charlotte Garner, Michel De Waard, Sebastian Amigorena, Anne Feltz, Jean-Maurice Mallet, Mayeul Collot*

Supplementary information

General methods (page 1)
Synthesis (page 2)
General procedures (page 2)
Synthesis & caracterisation of non phenolic X-Rhodamines (page 3)
Synthesis & caracterisation of the first set of H-Rubies (page 5)
Synthesis & caracterisation of Functionalisable H-Rubies (page 13)
Preparation of the dextran conjugates (page 34)
General Calculations and Terms (page 35)
Effect of HR-Br concentration on its apparent pKa and fluorescence enhancement (page 37)
LDH-Cytotoxicity assay (page 39)
Colocalisation experiments with H-Rubies and mitotracker green (page 40)
Effect of intracellular acidification and alkalinization on the H-Rubies' fluorescence enhancement (page 41)

General methods

All the solvents were of analytical grade. Chemicals were purchased from commercial sources. The salts used in stock solutions of metal ions were $CaCl_2 \cdot 2H_2O$, $CuCl_2 \cdot 2H_2O$, $FeCl_3 \cdot 6H_2O$, $MgCl_2 \cdot 6H_2O$, $MnCl_2 \cdot 4H_2O$, $ZnBr_2$. ^{1}H-NMR and ^{13}C-NMR were measured on a Bruker avance III-300 MHz spectrometer with chemical shifts reported in ppm (TMS as internal standard). Mass spectra were measured on a Focus GC / DSQ II spectrometer (ThermoScientific) for IC and an API 3000 spectrometer (Applied Biosystems, PE Sciex) for ES. All pH measurements were made with a Mettler Toledo pH-Meter. Fluorescence spectra were recorded on a JASCO FP-8300 spectrofluorometer. Absorption spectra were determined on a VARIAN CARY 300 Bio UV-Visible spectrophotometer. All measurements were done at a set temperature of 25°C and in a buffered solution containing MOPS (30 mM and KCl 100 mM, the pH was corrected with concentrated solutions of KOH or HCl. The purity of the dyes were checked by RP-HPLC C-18, eluant: ACN 0.1% TFA/Water 0.1% TFA, method: 20/80 to 100/0 within 20 min. then 100/0 for 10 min. detection at λ_{abs}= 254 nm. All H-Rubies were obtained as their trifluoroacetate salts.

Synthesis

The first set of H-Rubies were synthesised according to a general method described below, in some cases a protection step of the phenol group as an acetate was found to be necessary.

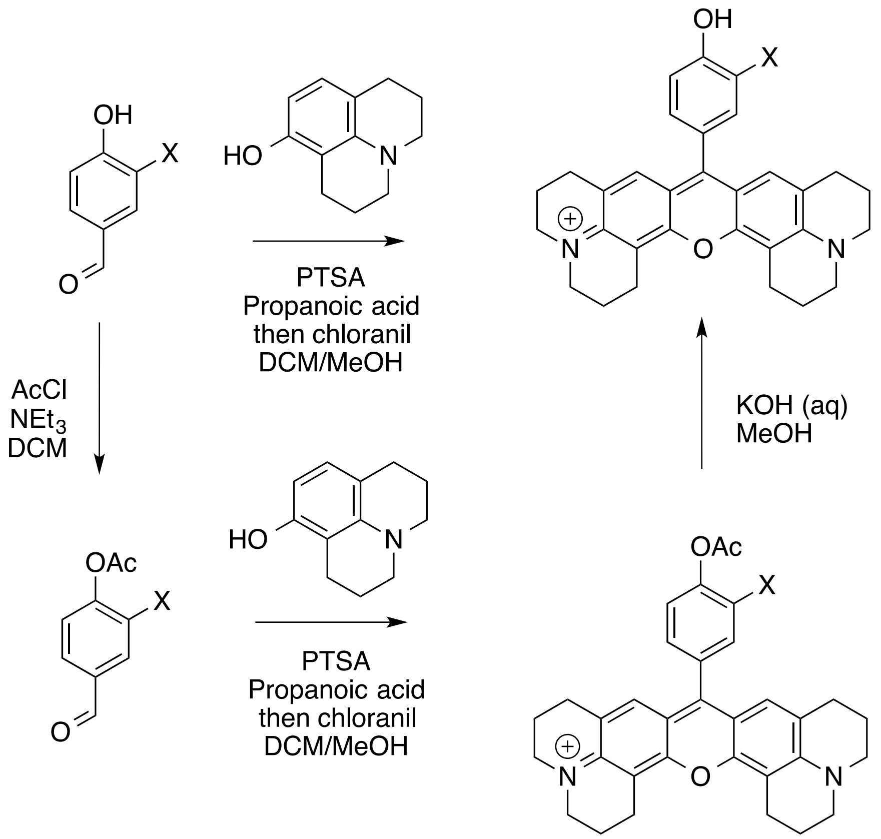

Scheme S1. General scheme for preparation of H-Rubies

General procedures

General procedure for X-rhodamine synthesis. To a solution of aldehyde (1eq) in propanoic acid was added 8-hydroxyjulolidine (2 eq) and PTSA (1 eq). The solution was protected from light and stirred at room temperature overnight. To the brown mixture was added a solution of chloranil (1 eq) in DCM./MeOH. The reaction turned dark and was allowed to stir overnight at room temperature. The dark purple solution was evaporated to dryness. The crude was purified by column chromatography on silica gel (DCM/MeOH, initially 98/2 reaching 90/10 over 30 min.) to obtain the X-Rhodamine product as a purple solid.

General procedure for phenol acetylation. To a solution of starting material (1 eq) in DCM was added triethylmine (3 eq) to form a homogeneous solution. Upon addition of acetyl chloride (1.5 eq) the solution became inhomogeneous and left to stir for 2h. The mixture was quenched with HCl and extracted 3 times with DCM. The combined organic phases were washed once with HCl, once with brine and dried over $MgSO_4$. The resulting solution was left under vacuum to yield the product.

General procedure for acetoxy deprotection. To a solution of starting material (1eq) in MeOH was added a 1M solution of KOH (35 eq). The solution was left stirring for 2h and extracted with DCM/HCl. The combined organic phases were dried over $MgSO_4$ and evaporated to dryness yielding the product as a purple solid after lyophilisation (dioxane/water : 1/1).

Synthesis & caracterisation of non phenolic X-Rhodamines

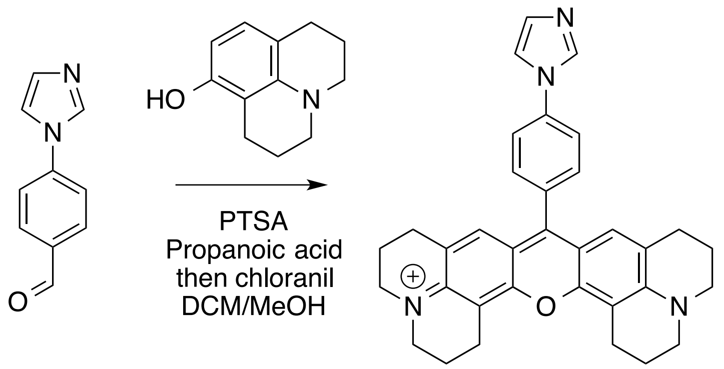

Scheme S2. Synthesis of Phenyl-Imidazole based X-rhodamine

Phenyl-Imidazol based X-rhodamine was synthesised via the general method and was obtained as a purple solid (4%). MS (ES+), calcd for $C_{34}H_{32}N_4O^+$ $[M]^+$ 513.26, found 513.5. . **HRMS:** *m/z* calcd for $C_{38}H_{45}N_4O_5^+$: 513.2649 $[M]^+$, found 513.2642.

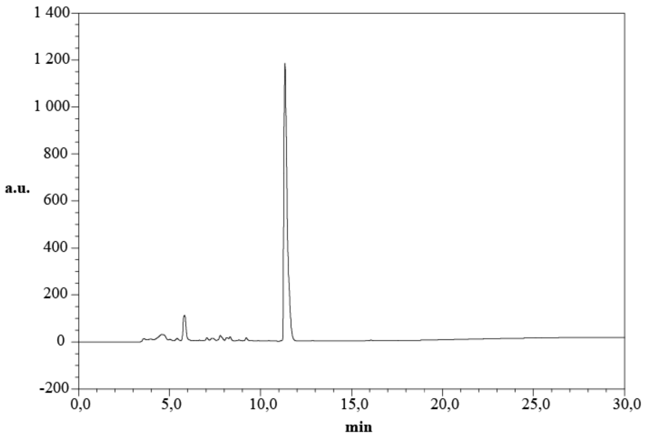

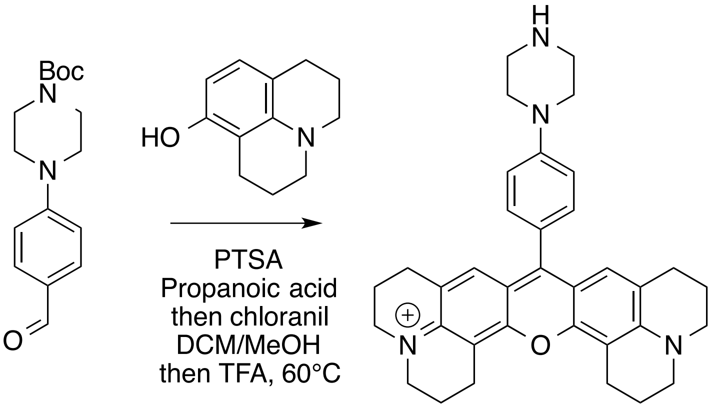

Scheme S3. Synthesis of Phenyl-piperazine based X-rhodamine **Pip-H**

Phenyl-piperazine based X-rhodamine was synthesised from starting aldehyde 1-Boc-4-(4-formylphenyl)piperazine via the general method and the Boc-protecting group removed via addition of TFA and heating at 60˚C yielding a purple solid (40%). **MS (ES+)**, calcd for $C_{35}H_{39}N_4O^+$ $[M]^+$ 531.31, found 5531.7.

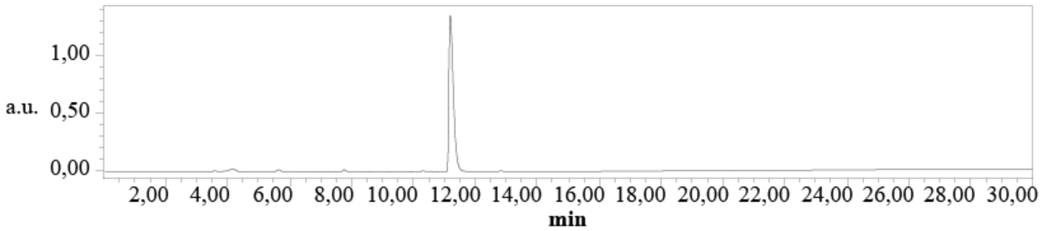

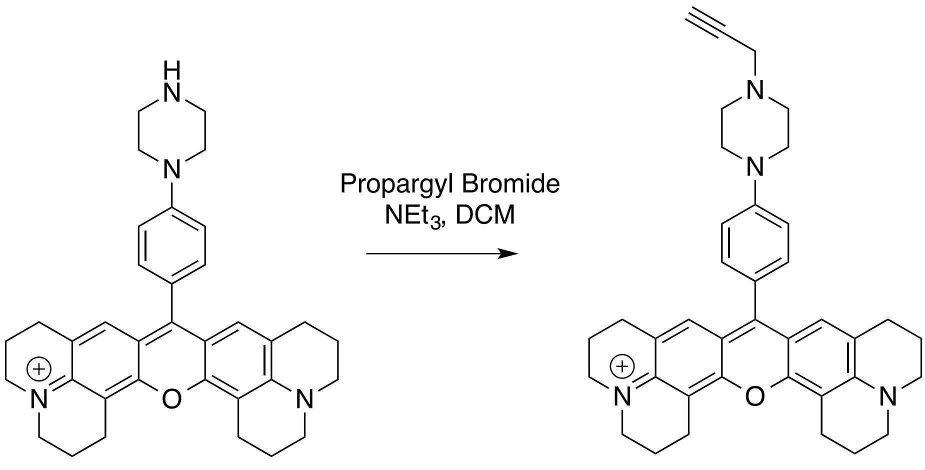

Scheme S4. Synthesis of **Pip-Alkyne**

To a solution of **Pip-H** (30mg, 0.05 mmol, 1 eq) in DCM (2 ml) was added triethylamine (0.04 ml, 0.28 mmol, 6 eq) and propargyl bromide (80 % in toluene) (0.021 ml, 0.14 mmol, 3 eq). The solution was left to stir at room temperature for 15h before the addition of a further 1.5 eq propargyl bromide and 3 eq triethylamine. Again stirred for 5h before the addition of a further 1.5 eq propargyl bromide and 30 eq triethylamine. The solution was left stirring for 15h and evaporated to dryness and

purified by column chromatography on silica gel (DCM/MeOH, initially 98/2 reaching 93/7 over 30 min.) to obtain the product as a purple solid (24.4 mg, 0.04, 80%) after lyophilisation (dioxane/water : 1/1). **MS (ES+)**, calcd for $C_{38}H_{41}N_4O^+$ $[M]^+$ 569.33, found 569.6. **HRMS:** *m/z* calcd for $C_{38}H_{41}N_4O^+$: 569.3275 $[M]^+$, found 569.3267.

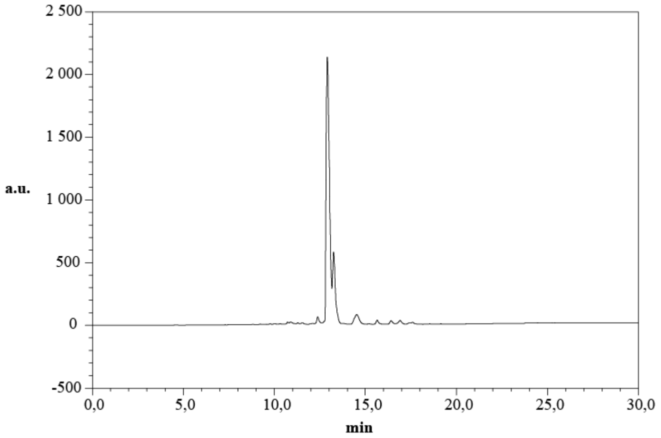

Synthesis & caracterisation of the first set of H-Rubies

Acetylated phenols and naphthols

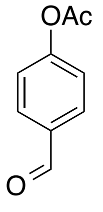

4-formylphenyl acetate

4-formylphenyl acetate was synthesized according to the general protocol (1.310 g, 8.0 mmol, 98%). **^{1}H NMR** (300 MHz, Chloroform-*d*) δ 9.93 (s, 1H, CHO), 7.91 – 7.80 (m, 2H, H_c, H_d), 7.27 – 7.16 (m, 2H, H_a, H_b), 2.27 (s, 3H, Me).[1]

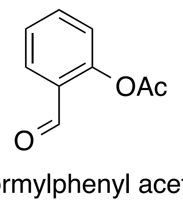

2-formylphenyl acetate

2-formylphenyl acetate was synthesized according to the general protocol (0.660 g, 4.0 mmol, 97%). **^{1}H NMR** (300 MHz, Chloroform-*d*) δ 10.02 (s, 1H, CHO), 7.80 (dd,

[1] B. R. Kim, G. H. Sung, S.-G. Lee, and Y. J. Yoon, *Tetrahedron*, 2013, **69**, 3234–3237.

$J_{c\text{-}d}$ = 7.7 Hz, $J_{b\text{-}d}$ = 1.8 Hz, 1H, H_d), 7.60 – 7.51 (m, 1H, H_b), 7.36 – 7.26 (m, 1H, H_c), 7.10 (dd, J = 8.2, 1.1 Hz, 1H, H_a), 2.31 (s, 3H, Me).[2]

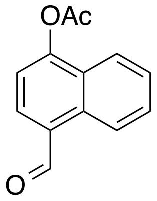

4-formylnaphthalen-1-yl acetate

4-formylnaphthalen-1-yl acetate was synthesized according to the general protocol (0.60g, 2.8 mmol, 97%). **^{1}H NMR** (300 MHz, Chloroform-d) δ 10.29 (s, 1H, C<u>H</u>O), 9.25 (ddd, $J_{e\text{-}f}$ = 8.6 Hz, $J_{f\text{-}d}$ = 1.3 Hz, $J_{f\text{-}c}$ = 0.7 Hz, 1H, H_f), 7.96-7.93 (m, 2H, H_b, H_c), 7.67 (ddd, $J_{e\text{-}f}$ = 8.2 Hz, $J_{e\text{-}d}$ = 6.9 Hz, $J_{e\text{-}c}$ = 1.3 Hz, 1H, H_e), 7.57 (ddd, $J_{c\text{-}d}$ = 8.2 Hz, $J_{e\text{-}d}$ = 6.9 Hz, $J_{d\text{-}f}$ = 1.3 Hz, 1H, H_d), 7.39 (d, $J_{a\text{-}b}$ = 7.8 Hz, 1H, H_a), 2.44 (s, 3H, Me). **^{13}C NMR** (75 MHz, $CDCl_3$) δ 192.62 (<u>C</u>HO), 168.74 (<u>C</u>OMe), 151.62 (CqAr), 137.04 (CqAr), 132.03 (CqAr), 129.69 (CqAr), 129.41 (CqAr), 127.52 (CqAr), 127.00 (CqAr), 125.22 (CqAr), 121.67 (CqAr), 117.40 (CqAr), 21.20 (<u>C</u>H_3). **MS (CI):** calcd for $C_{13}H_{14}NO_3$ $[M+NH_4]^+$, 232.06; found 231.94.

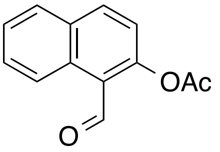

1-formylnaphthalen-2-yl acetate

1-formylnaphthalen-2-yl acetate was synthesized according to the general protocol (0.61g, 2.9mmol, 100%). **^{1}H NMR** (300 MHz, Chloroform-*d*) δ 10.64 (s, 1H, C<u>H</u>O), 9.07 (d, $J_{e\text{-}f}$ = 8.8 Hz, 1H, H_f), 8.05 (d, $J_{a\text{-}b}$ = 9.0 Hz, 1H, H_a), 7.83-7.80 (m, 1H, H_c), 7.56 (m, 2H, H_d, H_e) , 7.21 (d, $J_{a\text{-}b}$ = 9.0 Hz, 1H, H_b), 2.38 (s, 3H, Me).[3]

Acetylated H-Rubies

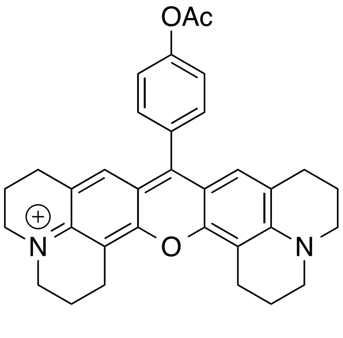

[2] G. Pelletier, W. S. Bechara, and A. B. Charette, *J. Am. Chem. Soc.*, 2010, **132**, 12817–12819.

[3] J.-L. Débieux, A. Cosandey, C. Helgen, and C. G. Bochet, *Eur. J. Org. Chem.*, 2007, **2007**, 2073–2077.

Acetylated **HR-*p*OH** was synthesized according to the general protocol from starting 4-formylphenyl acetate (40 mg, 0.06 mmol, 10%). **^{1}H NMR** (300 MHz, Chloroform-*d*) δ 7.80 – 7.69 (m, 2H, H_a, H_e), 7.27 (s, 4H, Hpy_{PTSA}), 7.01 – 6.92 (m, 2H, H_b, H_d), 6.73 (s, 2H, H_f), 3.47 (m, 8H, H_i, H_j), 2.95 (t, *J* = 6.4 Hz, 4H, H_l), 2.63 (t, *J* = 6.2 Hz, 4H, H_g), 2.33 (s, 3H, Me), 2.18 (s, 3H, Me $_{PTSA}$), 2.02 (m, 4H, H_k), 1.90 (m, 4H, H_h). **^{13}C NMR** (75 MHz, $CDCl_3$) δ 169.31 (<u>C</u>OMe), 153.23 (C Ar), 152.11 (C Ar), 151.66 (C Ar), 151.17 (C Ar), 144.53 (C Ar), 138.20 (C Ar), 130.66 (C_b or C_d), 130.13 (C Ar), 128.16 (C_b or C_d), 126.42 (C Ar), 126.27 (C Ar), 123.81 (C_a or C_e), 122.15 (C_a or C_e), 112.76 (C Ar), 105.50 (C Ar), 50.97 (C_i or C_j), 50.51 (C_i or C_j), 27.68 (C_g) , 21.30 (<u>C</u>H_3), 20.65 (C_h), 19.92 (C_l), 19.70 (C_k). **MS (ES+)**, calcd for $C_{33}H_{33}N_2O_3^+$ $[M]^+$ 505.25, found 505.7.

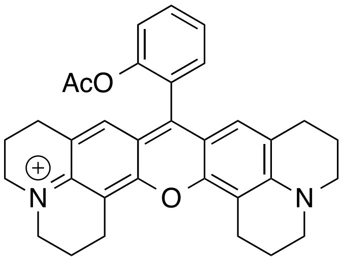

Acetylated **HR-*o*OH** was synthesized according to the general protocol from starting 2-formylphenyl acetate (52 mg, 0.08 mmol, 13%). **^{1}H NMR** (300 MHz, Chloroform-*d*) δ 7.73 (d, *J* = 7.8 Hz, 2H, ArH $_{PTSA}$), 7.63 – 7.34 (m, 2H, H_d and H_b), 7.32 – 7.15 (m, 2H, H_c and H_a), 6.97 (d, *J* = 7.8 Hz, 2H, ArH $_{PTSA}$), 6.64 (s, 2H, H_f), 3.47 (m, 8H, H_i and H_j), 2.95 (t, *J* = 6.5 Hz, 4H, H_l), 2.61 (t, *J* = 6.3 Hz, 4H, H_g), 2.19 (s, 3H, Me), 2.08 – 1.83 (m, 8H, H_h and H_k), 1.84 (s, 3H, Me $_{PTSA}$). **^{13}C NMR** (75 MHz, $CDCl_3$) δ 168.94 (<u>C</u>OMe), 151.96 (C Ar), 151.28 (C Ar), 149.74 (C Ar), 148.13 (C Ar), 144.29 (C Ar), 138.37, 131.15, 130.98, 128.20, 126.32 (C Ar), 126.27 (C Ar), 125.88 (C Ar), 123.84 (C Ar), 123.63 (C Ar), 112.65 (C Ar), 105.38 (C Ar), 51.00 (C_i or C_j), 50.55 (C_i or C_j), 27.54 (C_g), 21.29 (<u>C</u>H_3), 20.84 (<u>C</u>H_3), 20.64 (C_h), 19.90 (C_l), 19.66 (C_k). **MS (ES+)**, calcd for $C_{33}H_{33}N_2O_3^+$ $[M]^+$ 505.25, found 505.5.

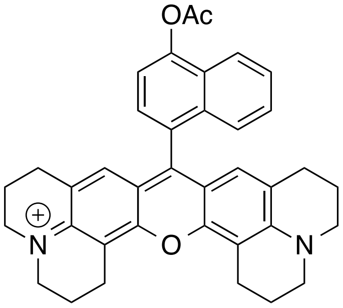

Acetylated *p*-Nph was synthesized according to the general protocol from starting 4-formylnaphthalen-1-yl acetate (23 mg, 0.032 mmol, 7%). **^{1}H NMR** (300 MHz, Chloroform-*d*) δ 7.99 (d, *J* = 8.5 Hz, 1H, ArH), 7.71 (d, *J* = 7.7 Hz, 2H, PTSA), 7.59 – 7.21 (m, 5H, ArH), 6.97 (d, *J* = 7.7 Hz, 2H, PTSA), 6.47 (s, 2H, H_f), 3.47 (m, 8H, H_i and H_j), 3.00 (t, *J* = 6.4 Hz, 4H, H_l), 2.49 (s, 7H, CO<u>CH</u>$_3$, H_g), 2.19 (s, 3H, Me $_{PTSA}$), 2.06 (p, *J* = 6.2 Hz, 4H, H_k), 1.85 (q, *J* = 6.4, 5.9 Hz, 4H, H_h). **^{13}C NMR** (75

MHz, $CDCl_3$) δ 169.35 (COMe), 152.53 (C Ar), 152.07 (C Ar), 151.36 (C Ar), 147.81 (C Ar), 138.55 (C Ar), 132.85 (C Ar), 128.32 (C Ar), 128.28 (C Ar), 128.00 (C Ar), 127.40 (C Ar), 127.18 (C Ar), 126.86 (C Ar), 126.37 (C Ar), 126.19 (C Ar), 125.73 (C Ar), 123.97 (C Ar), 121.90 (C Ar), 117.63 (C Ar), 113.79 (C Ar), 105.49 (C Ar), 51.01 (C_i or C_j), 50.61 (C_i or C_j), 27.52 (C_g), 21.31 (CH_3), 21.22 (CH_3), 20.57 (C_h), 19.95 (C_l), 19.72 (C_k). **MS (ES+)**, calcd for $C_{37}H_{35}N_2O_3^+$ $[M]^+$ 555.26, found 555.1.

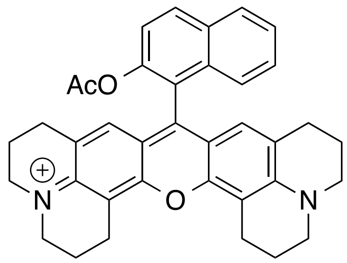

Acetylated ***o*-Nph** was synthesized according to the general protocol from 1-formylnaphthalen-2-yl (18 mg, 0.025 mmol, 5%). **^{1}H NMR** (300 MHz, Chloroform-*d*) δ 8.09 (dd, *J* = 34.3, 8.6 Hz, 2H), 7.85 (d, *J* = 7.9 Hz, 2H, PTSA), 7.64 – 7.37 (m, 2H, ArH), 7.37 – 7.18 (m, 1H), 7.09 (d, *J* = 7.8 Hz, 1H), 6.54 (s, 2H, H_f), 3.58 (m, 8H, H_i and H_j), 3.12 (t, *J* = 6.5 Hz, 2H, H_l), 2.59 (m, 4H, H_g), 2.31 (s, 3H, $COCH_3$), 2.15 (m, 4H, H_k), 2.02 (s, 3H, Me $_{PTSA}$), 1.92 (m, 4H, H_h). **^{13}C NMR** (75 MHz, $CDCl_3$) δ 169.19 (COMe), 151.93 (C Ar), 151.48 (C Ar), 146.28 (C Ar), 143.54 (C Ar), 138.47 (C Ar), 132.31 (C Ar), 131.30 (C Ar), 128.49 (C Ar), 128.22 (C Ar), 127.98 (C Ar), 126.57 (C Ar), 126.25 (C Ar), 126.06 (C Ar), 125.21 (C Ar), 123.98 (C Ar), 121.89 (C Ar), 119.35 (C Ar), 113.37 (C Ar), 105.57 (C Ar), 51.01 (C_i or C_j), 50.66 (C_i or C_j), 27.42 (C_g), 21.29 (CH_3), 20.90 (CH_3), 20.56 (C_h), 19.94 (C_l), 19.69 (C_k). **MS (ES+)**, calcd for $C_{37}H_{35}N_2O_3^+$ $[M]^+$ 555.26, found 555.0.

H-Rubies

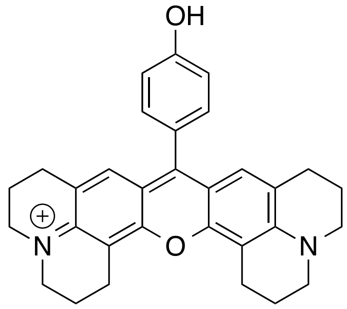

HR-*p*OH was synthesised from acetylated HR-*p*OH according to the general protocol (100%). **MS (ES+),** calcd for $C_{31}H_{31}N_2O_2^+$ $[M]^+$ 463.24, found 463.7. **HRMS:** *m/z* calcd for $C_{31}H_{31}N_2O_2^+$: 463.2380 $[M]^+$, found 463.2378.

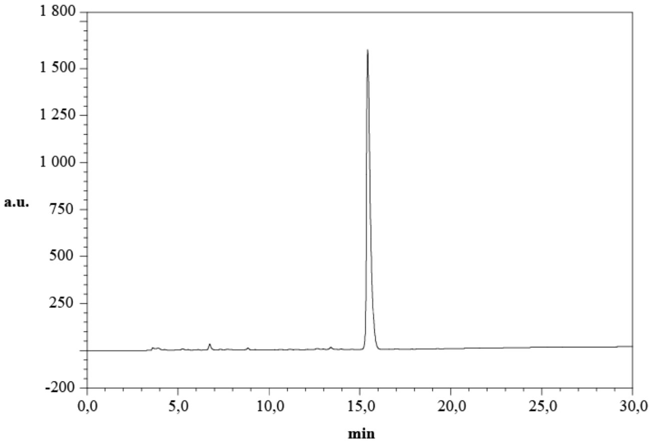

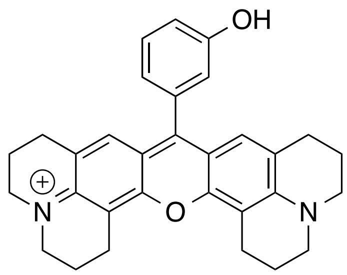

HR-*m*OH was synthesised from meta-hydroxybenzaldehyde according to the general protocol (13%). **MS (ES+),** calcd for $C_{31}H_{31}N_2O_2^+$ $[M]^+$ 463.24, found 463.7. **HRMS:** *m/z* calcd for $C_{31}H_{31}N_2O_2^+$: 463.2380 $[M]^+$, found 463.2378.

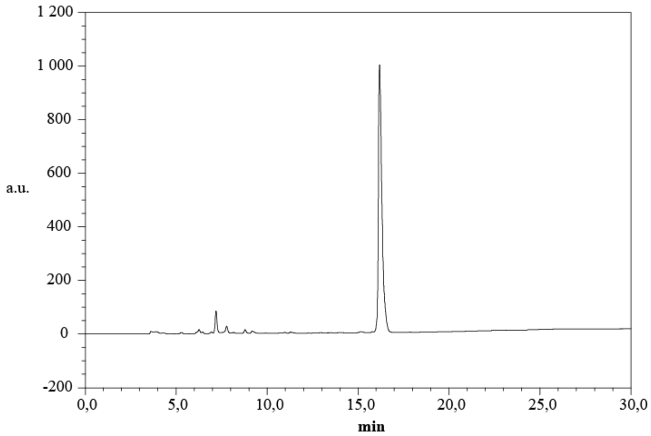

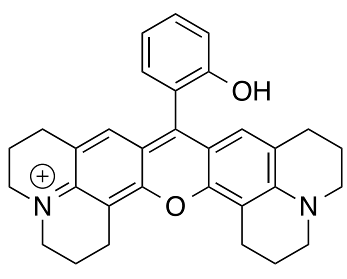

HR-*o*OH was synthesised from acetylated HR-*o*OH according to the general protocol (100%). **MS (ES+)**, calcd for $C_{31}H_{31}N_2O_2^+$ $[M]^+$ 463.24, found 463.6. **HRMS:** *m/z* calcd for $C_{31}H_{31}N_2O_2^+$: 463.2380 $[M]^+$, found 463.2378.

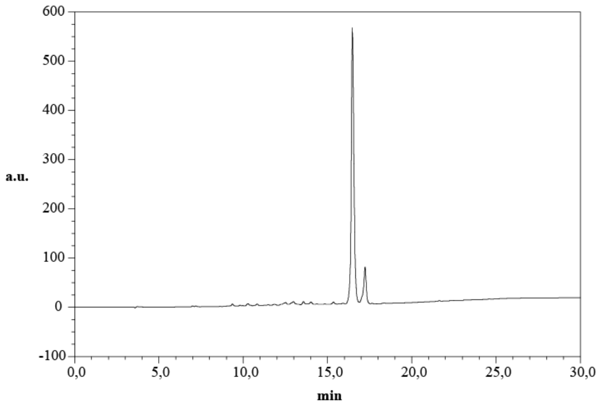

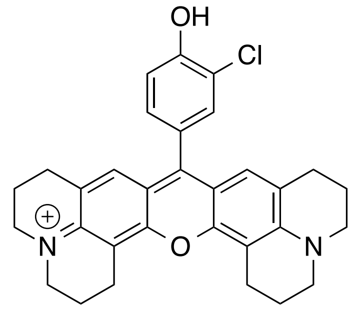

HR-Cl was synthesised from 3-Chloro-4-hydroxybenzaldehyde according to the general protocol (16%). **MS (ES+)**, calcd for $C_{31}H_{30}ClN_2O_2^+$ $[M]^+$ 497.20, found 497.4. **HRMS:** *m/z* calcd for $C_{31}H_{30}ClN_2O_2^+$: 497.1990 $[M]^+$, found 497.1986.

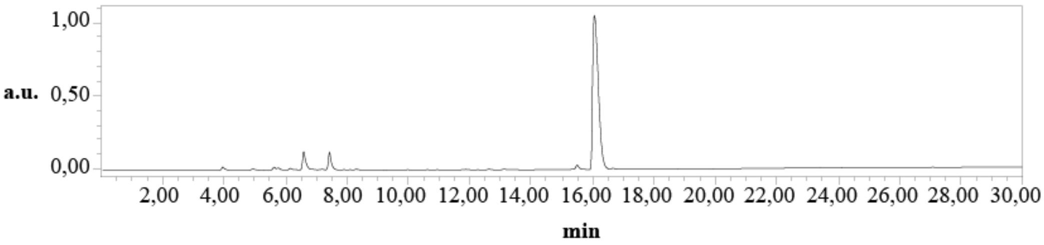

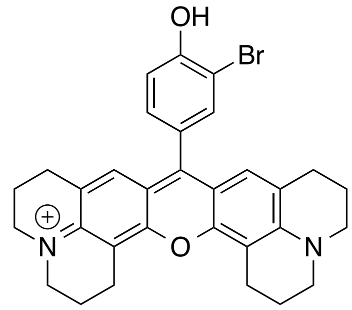

HR-Br was synthesised from 3-Bromo-4-hydroxybenzaldehyde according to the general protocol (16%). **MS (ES+),** calcd for $C_{31}H_{30}BrN_2O_2^+$ $[M]^+$ 541.15, found 541.4. **HRMS:** *m/z* calcd for $C_{31}H_{30}BrN_2O_2^+$: 541.1485 $[M]^+$, found 541.1482

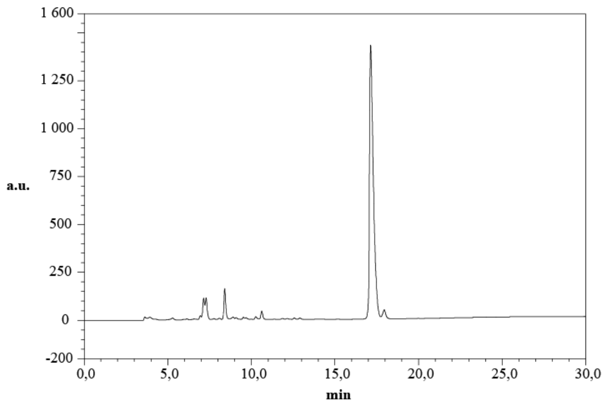

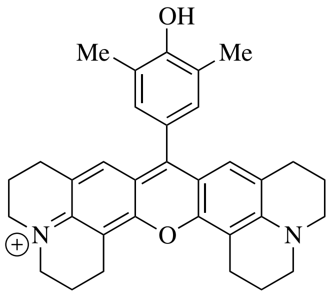

Chemical Formula: $C_{33}H_{35}N_2O_2^+$
Exact Mass: 491.27
Molecular Weight: 491.64

HR-Me was synthesised from 4-Hydroxy-3,5-dimethylbenzaldehyde according to the general protocol (10%). **MS (ES+),** calcd for $C_{33}H_{35}N_2O_2^+$ $[M]^+$ 491.27, found 491.8. **HRMS:** *m/z* calcd for $C_{33}H_{35}N_2O_2^+$: 491.2693 $[M]^+$, found 491.2687.

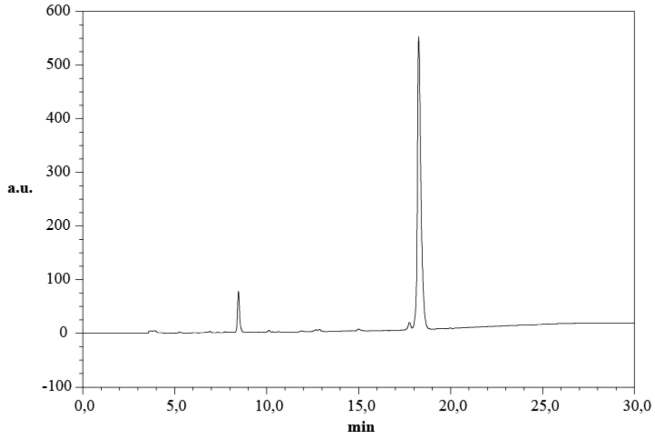

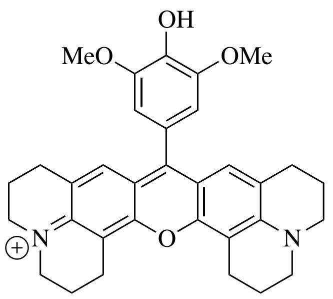

Chemical Formula: $C_{33}H_{35}N_2O_4^+$
Exact Mass: 523.26
Molecular Weight: 523.64

HR-OMe was synthesised from 3,5-Dimethoxy-4-hydroxybenzaldehyde according to the general protocol (10%). **MS (ES+),** calcd for $C_{33}H_{35}N_2O_4^+$ $[M]^+$ 523.26, found 523.7. **HRMS:** *m/z* calcd for $C_{33}H_{35}N_2O_4^+$: 523.2591 $[M]^+$, found 523.2585.

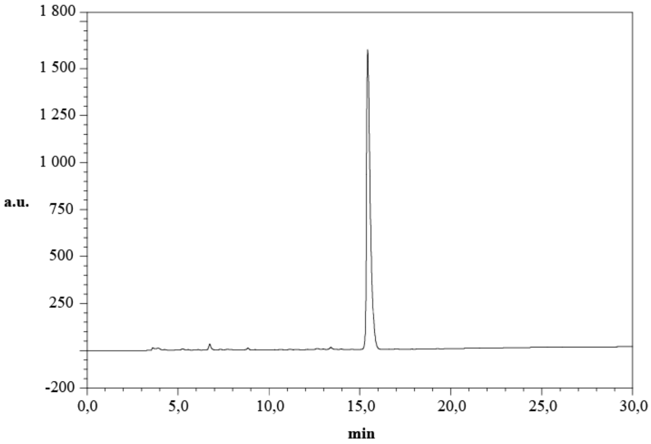

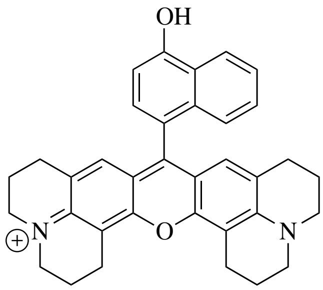

Chemical Formula: $C_{35}H_{33}N_2O_2^+$
Exact Mass: 513.25
Molecular Weight: 513.65

p-Nph was synthesised from acetylated *p*-Nph according to the general protocol (69%). **MS (ES+)**, calcd for $C_{35}H_{33}N_2O_2^+$ $[M]^+$ 513.25, found 513.4. **HRMS:** *m/z* calcd for $C_{35}H_{33}N_2O_2^+$: 513.2537 $[M]^+$, found 513.2531.

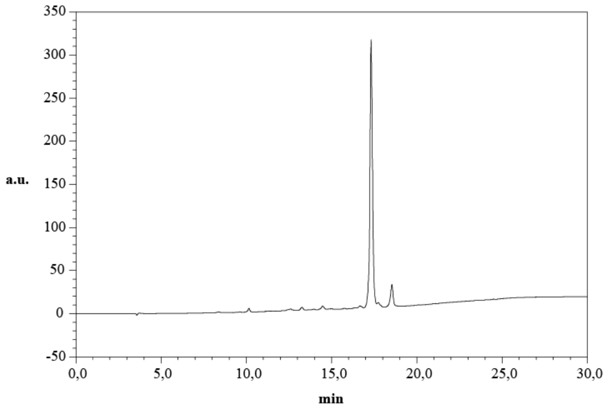

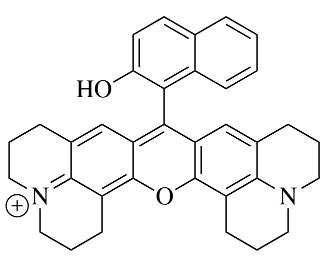

Chemical Formula: $C_{35}H_{33}N_2O_2^+$
Exact Mass: 513.25
Molecular Weight: 513.65

o-Nph was synthesised from acetylated *o*-Nph according to the general protocol (100%). **MS (ES+)**, calcd for $C_{35}H_{33}N_2O_2^+$ $[M]^+$ 513.25, found 513.4. **HRMS:** *m/z* calcd for $C_{35}H_{33}N_2O_2^+$: 513.2537 $[M]^+$, found 513.2533.

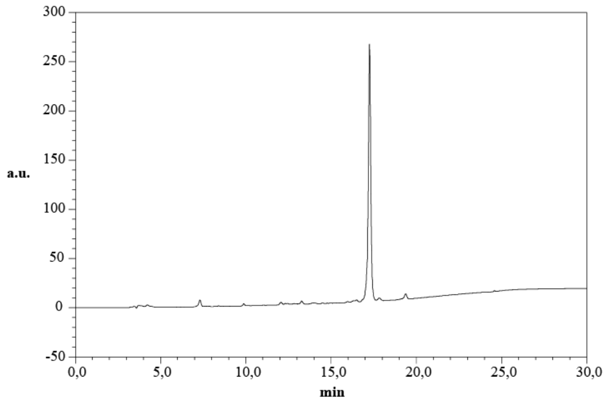

Synthesis & caracterisation of Functionalisable H-Rubies

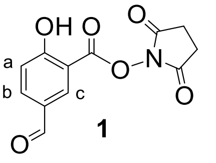

1

2,5-dioxopyrrolidin-1-yl 5-formyl-2-hydroxybenzoate 1: To a suspension of 5-formyl salicylic acid (4 g, 24.1 mmol) and N-hydroxysuccinimide (3.05 g, 26.5 mmol) in THF (50 mL) was added DCC (5.47 g, 26.5 mmol). The mixture was stirred at room temperature for 30 min then filtered over a pad of celite. The filtrate was concentrated under reduced pressure then the residue was purified by flash chromatography (cyclohexane/ethyl acetate 1:1 to 2:3) to give 1 (3 g, 47 %) as a white solid; ^{1}H NMR ($CDCl_3$, 300MHz) δ 10.02 (bs, 1H, OH), 9.90 (s, 1H, CHO), 8.52 (d, $J_{c,b}$ = 1.9 Hz, 1H, Hc), 8.12 (dd, $J_{b,a}$ = 8.8 Hz, $J_{b,c}$ = 2.0 Hz, 1H, Hb), 7.18 (d, $J_{a,b}$ = 8.7 Hz, 1H, Ha), 2.94 (s, 4H, C(O)(C$\underline{H_2}$)$_2$C(O)); ^{13}C NMR ($CDCl_3$, 75 MHz) δ 189.3 (CHO), 168.7, 166.5, 164.8 (C Ar), 137.3 (C Ar), 134.2 (C Ar), 129.4 (C Ar), 119.5 (C Ar), 108.7, 25.8 (C(O)($\underline{C}H_2$)$_2$C(O)); **HRMS:** *m/z* calcd for $C_{12}H_9NO_6Na$: 286.0322 $[M+Na]^+$, found 286.0325.

Procedures for the preparation of non commercially available amines

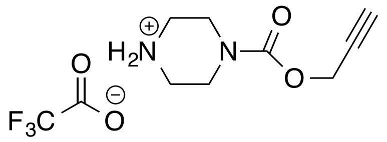

4-((prop-2-yn-1-yloxy)carbonyl)piperazin-1-ium 2,2,2-trifluoroacetate: To a solution of 1-Boc-piperazine (0.510 g, 2.74 mmol) and triethylamine (1.15 mL, 8.22 mmol) in DCM (10 mL), was added dropwise at 0°C propargyl chloroformate (0.400 mL, 4.11 mmol). The mixture was allowed to warm to room temperature, stirred for 16 h then concentrated. The residue was taken up in ethyl acetate then washed with 1N aq. HCl. The aqueous layer was extracted with ethyl acetate then the combined organic layers were washed with brine, dried over MgSO4, filtered and concentrated. The residue was purified by flash chromatography (cyclohexane/ethyl acetate 95:5 to 85:15) to afford 1-tert-butyl 4-prop-2-yn-1-yl piperazine-1,4-dicarboxylate (0.600 g, 82 %) as an amorphous white solid; ^{1}H NMR ($CDCl_3$, 300MHz) δ 4.71 (d, J = 2.4 Hz, 1H, $OC\underline{H_2}C\equiv CH$), 3.49 – 3.39 (m, 8H, $(CH_2)_4$), 2.46 (t, J = 2.4 Hz, 1H, $OCH_2C\equiv C\underline{H}$); ^{13}C NMR ($CDCl_3$, 75 MHz) δ 154.7 (NC=O), 154.5 (NC=O), 80.4, 78.4 ($OCH_2\underline{C}\equiv CH$), 74.8 ($OCH_2C\equiv \underline{C}H$), 53.3 ($(CH_2)_4$), 43.9 ($O\underline{C}H_2C\equiv CH$), 28.5 ($C(\underline{C}H_3)_3$).

1-tert-butyl 4-prop-2-yn-1-yl piperazine-1,4-dicarboxylate (0.395 g, 1.47 mmol) was dissolved in a 1:1 DCM/TFA mixture. The mixture was sonicated for 30 min then concentrated to dryness. The residue was taken up in methanol then stirred with Amberlite IRA 67 for 10 min, filtered and concentrated to dryness to afford 4-((prop-2-yn-1-yloxy)carbonyl)piperazin-1-ium 2,2,2-trifluoroacetate (0.359 g, 87 %) as a white amorphous solid; ^{1}H NMR ($CDCl_3$, 300MHz) δ 4.67 (d, J = 2.4 Hz, 1H, $OC\underline{H_2}C\equiv CH$), 3.58 – 3.55 (m, 4H, $C\underline{H_2}N(CO)C\underline{H_2}$), 3.07 (bs, 2H, NH_2), 2.96 – 2.92 (m, 4H, $C\underline{H_2}NH_2C\underline{H_2}$), 2.47 (t, J = 2.4 Hz, 1H, $OCH_2C\equiv C\underline{H}$); ^{13}C NMR ($CDCl_3$, 75 MHz) δ 154.5 (NC=O), 78.5 ($OCH_2\underline{C}\equiv CH$), 77.4, 74.7 ($OCH_2C\equiv \underline{C}H$), 53.2 ($(CH_2)_4$), 45.4 ($O\underline{C}H_2C\equiv CH$), 44.5; HRMS: m/z calcd for $C_8H_{13}N_2O_2^+$: 169.0972 $[M]^+$, found 169.0972.

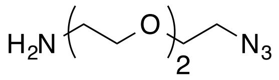

2-(2-(2-azidoethoxy)ethoxy)ethanamine: To a solution of 2-[2-(2-chloroethoxy)ethoxy]ethanol (20 mL, 137 mmol) in water (80 mL) was added sodium azide (18 g, 277 mmol). The solution was stirred at 80°C overnight then cooled to room temperature. The aqueous mixture was extracted with ethyl acetate then the combined organic layers were dried over $MgSO_4$, filtered and concentrated to afford 2-[2-(2-azidoethoxy)ethoxy]ethanol. Analytical and spectroscopic data were found to be in agreement with reported literature. [4] To a solution of 2-[2-(2-azidoethoxy)ethoxy]ethanol (5.26 g, 30.1 mmol) in DCM (60 mL) was added

[4] Natarajan, A.; Du, W.; Xiong, C.-Y.; DeNardo, G. L.; De Nardo, S. J.; Gervay-Hague, J. *Chem. Commun.* **2007**, 695-697.

triethylamine (5 mL, 36.1 mmol). The solution was cooled to 0°C then mesyl chloride (2.7 mL, 34.6 mmol) in DCM (10 mL) was added dropwise. The mixture was allowed to warm to room temperature, stirred overnight then filtered. The filtrate was washed with brine then dried over MgSO4, filtered and concentrated to afford 2-(2-(2-azidoethoxy)ethoxy)ethyl methanesulfonate (7.6 g, quantitative) as a yellowish oil. Analytical and spectroscopic data were found to be in agreement with reported literature.[5] 2-(2-(2-azidoethoxy)ethoxy)ethyl methanesulfonate was dissolved in 30% NH_4OH (70 mL) then the mixture was stirred at 80°C for 4 h. After cooling to room temperature, the mixture was concentrated and washed with 3N aq. NaOH then extracted with DCM. The combined organic layers were dried over MgSO4, filtered and concentrated to afford 2-(2-(2-azidoethoxy)ethoxy)ethanamine (3.52 g, 90 %) as a yellow oil. Analytical and spectroscopic data were found to be in agreement with reported literature.[4]

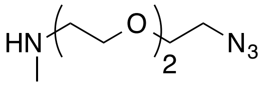

2-(2-(2-azidoethoxy)ethoxy)-N-methylethanamine: To a solution of 2-(2-(2-azidoethoxy)ethoxy)ethyl methanesulfonate (0.360 g, 1.42 mmol) in THF (1.5 mL) was added at 60°C 2M methylamine in methanol (7 mL, 14.2 mmol). The mixture was stirred overnight at 60°C then concentrated. The residue was taken up in DCM and washed with aq. 3N NaOH. The aqueous layer was extracted with DCM then the combined organic layers were dried over $MgSO_4$, filtered and concentrated. The residue was purified by flash chromatography (DCM/methanol 95:5 to 85:15) to give 2-(2-(2-azidoethoxy)ethoxy)ethanamine (0.150 g, 56 %) as a yellowish oil; ^{1}H NMR ($CDCl_3$, 300MHz) δ 3.70 – 3.59 (m, 8H), 3.39 (t, J = 5.1 Hz, 2H), 2.77 (t, J = 5.2 Hz, 2H), 2.45 (s, 3H, CH_3); HRMS: *m/z* calcd for $C_7H_{17}N_4O_2$: 189.1346 $[M+H]^+$, found 189.1349.

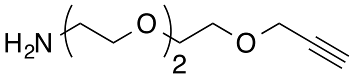

2-(2-(2-(prop-2-yn-1-yloxy)ethoxy)ethoxy)ethanamine: 2-{2-[2-(Prop-2-ynyloxy)ethoxy]ethoxy}ethyl 4-methylbenzenesulfonate (2.69 g, 7.86 mmol) was treated as described for 2-(2-azidoethoxy)ethanamine to afford 2-(2-(2-(prop-2-yn-1-yloxy)ethoxy)ethoxy)ethanamine (1.20 g, 82 %) as a yellowish oil. Analytical and spectroscopic data were found to be in agreement with reported literature.[4]

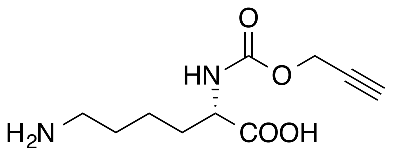

[5] Huang, C.-Y.; Hong, C.-W.; Ko, F.-H.; Chang, F.-C. *Soft Matter* **2011**, *7*, 10850-10855.

5-carboxy-5-(((prop-2-yn-1-yloxy)carbonyl)amino)pentan-1-aminium 2,2,2-trifluoroacetate: To a solution of H-Lys(Boc)-OH (1 g, 4.06 mmol) and $NaHCO_3$ (0.750 g, 8.93 mmol) in a 1:1 mixture of dioxane and water (20 mL) was added dropwise propargyl chloroformate (0.600 mL, 6.09 mmol). The mixture was stirred for 2 h at room temperature then dioxane was removed under reduced pressure. After dilution with satd. aq. $NaHCO_3$, the aqueous mixture was washed three times with ethyl acetate then acidified with 1N aq. HCl (pH ~ 3) and extracted with ethyl acetate. The combined organic layers were dried over $MgSO_4$, filtered and concentrated. The residue was purified by flash chromatography (DCM/methanol (0.5 % AcOH v/v) 98:2 to 92:8) to afford 6-((tert-butoxycarbonyl)amino)-2-(((prop-2-yn-1-yloxy)carbonyl)amino)hexanoic acid (0.820 g, 62 %, mixture of rotameres a/b = 2:1) as a colourless syrup; ^{1}H NMR ($CDCl_3$, 300MHz) δ 7.83 (bs, 1H, CO_2H), 6.32 (bs, 1H, NH rotamer a), 6.08 (bs, 1H, NH rotamer b), 5.81 – 5.69 (m, 1H, NH), 4.74 – 4.63 (m, 2H, OC$\underline{H_2}$C≡CH), 4.37 (bs, 1H, HNC$\underline{H}$(CO_2H)CH_2), 3.13 – 3.11 (m, 2H, C$\underline{H_2}$NHBoc), 1.93 – 1.72 (m, 2H), 1.52 – 1.44 (m, 13H, C(C$\underline{H_3}$)$_3$, (C$\underline{H_2}$)$_3$$CH_2$NHBoc); ^{13}C NMR ($CDCl_3$, 75 MHz) δ 79.8, 78.2, 75.0, 53.9, 53.0, 40.1, 31.8, 29.7, 28.6, 22.3; HRMS: *m/z* calcd for $C_{15}H_{24}N_2O_6Na$: 351.1527 $[M+Na]^+$, found 351.1525.

6-((tert-butoxycarbonyl)amino)-2-(((prop-2-yn-1-yloxy)carbonyl)amino)hexanoic acid (0.770 g, 2.34 mmol) was sonicated for 1 h in a 49:49:1 mixture of DCM/TFA/water then concentrated. The residue was taken up in methanol then stirred with Amberlite IRA 67 for 10 min, filtered and concentrated to dryness to afford 5-carboxy-5-(((prop-2-yn-1-yloxy)carbonyl)amino)pentan-1-aminium 2,2,2-trifluoroacetate (0.424 g, 53 %) as a white amorphous solid; ^{1}H NMR (CD_3OD, 300MHz) δ 4.71 – 4.59 (m, 2H, OC$\underline{H_2}$C≡CH), 4.02 (t, J = 6.0 Hz, 1H, HNC$\underline{H}$(CO_2H)CH_2), 2.94 – 2.86 (m, 3H, C$\underline{H_2}$$NH_3^+$, O$CH_2$C≡C$\underline{H}$), 1.90 – 1.79 (m, 1H), 1.76 – 1.63 (m, 3H), 1.50 – 1.40 (m, 2H); ^{13}C NMR (CD_3OD, 75 MHz) δ 178.4 (CO_2H), 157.3 (NHC=O), 79.5, 75.8, 57.0, 53.1, 40.5, 33.3, 28.1, 23.4; HRMS: *m/z* calcd for $C_{10}H_{17}N2O_4^+$: 229.1183 $[M]^+$, found 229.1183.

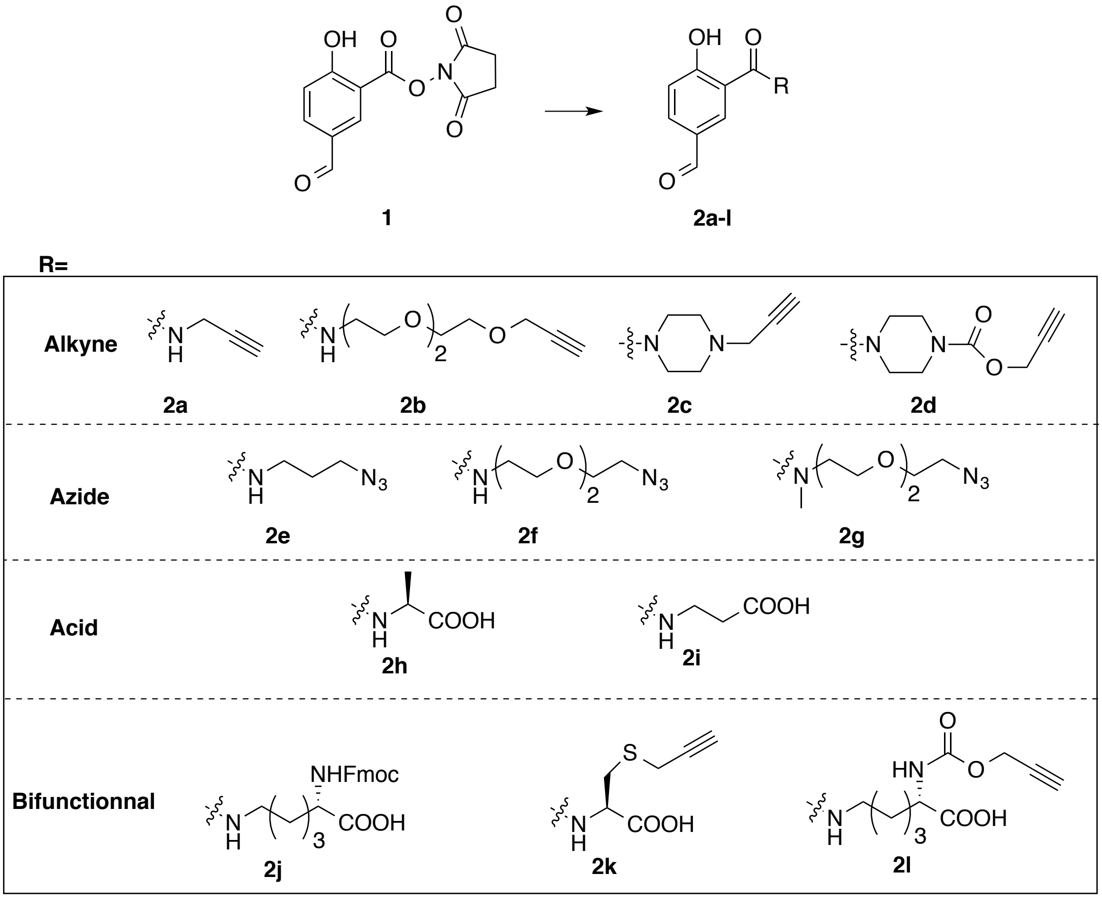

Scheme S5. General procedure for the preparation of aldehydes **2a** to **12a**

To a solution of **1** in DMSO (C = 0.05 - 0.1 M) was added amino linker (1.2 - 1.5 equiv) and DIEA (2 - 5 equiv). The solution was stirred overnight at room temperature then diluted in ethyl acetate and washed three times with 1N aq. HCl. The aqueous phase was extracted with ethyl acetate then the combined organic layers were dried over $MgSO_4$, filtered and concentrated. The residue was purified by flash chromatography to afford the desired functionalized aldehyde.

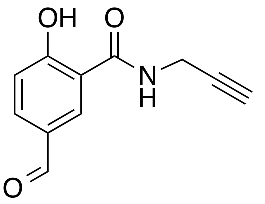

5-formyl-2-hydroxy-N-(prop-2-yn-1-yl)benzamide 2a: 1 (0.700 g, 2.66 mmol); propargylamine (0.250 mL, 3.99 mmol); DIEA (0.930 mL, 5.32 mmol). **2a** (0.308 g, 57 %) as a white amorphous solid after flash chromatography (cyclohexane/ethyl acetate 7:3 to 1:1); ^{1}H NMR ($(CD_3)_2CO$, 300MHz) δ 13.36 (bs, 1H, OH), 9.86 (s, 1H, CHO), 8.93 (bs, 1H, NH), 8.41 (d, $J_{c,b}$ = 2.0 Hz, 1H, Hc), 7.99 (dd, $J_{b,a}$ = 8.6 Hz, $J_{b,c}$ = 2.0 Hz, 1H, Hb), 7.10 (d, $J_{a,b}$ = 8.6 Hz, 1H, Ha), 4.28 (dd, J = 5.6 Hz, J = 2.6 Hz, 2H, NC<u>H</u>$_2$C≡CH), 2.77 (t, J = 2.5 Hz, 1H, NCH$_2$C≡C<u>H</u>); ^{13}C NMR ($(CD_3)_2CO$, 75 MHz) δ 190.5 (CHO), 170.2 (C Ar), 167.5 (C Ar), 135.7 (C Ar), 130.7 (C Ar), 129.4 (C Ar),

119.6 (C Ar), 80.3 ($CH_2C≡CH$), 72.7 ($CH_2C≡CH$). HRMS: *m/z* calcd for $C_{11}H_{10}NO_3$: 204.0655 $[M+H]^+$, found 204.0655.

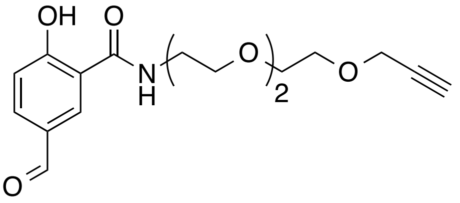

5-formyl-2-hydroxy-N-(2-(2-(2-(prop-2-yn-1-loxy)ethoxy)ethoxy)ethyl)benzamide 2b: 1 (0.500 g, 1.90 mmol); 2-(but-3-yn-1-yloxy)ethanamine (0.533 g, 2.85 mmol); DIEA (1 mL, 5.70 mmol). **2b** (0.530 g, 83 %) as a yellowish syrup after flash chromatography (cyclohexane/ethyl acetate 3:2 to 1:1); ^{1}H NMR ($CDCl_3$, 300MHz) δ 13.32 (bs, 1H, OH), 9.84 (s, 1H, CHO), 8.13 (d, $J_{c,b}$ = 2.0 Hz, 1H, Hc), 7.86 (dd, $J_{b,a}$ = 8.6 Hz, $J_{b,c}$ = 2.0 Hz, 1H, Hb), 7.59 (bs, 1H, NH), 7.04 (d, $J_{a,b}$ = 8.6 Hz, 1H, Ha), 4.11 (d, J = 2.4 Hz, 2H, $OCH_2C≡CH$), 3.73 – 3.62 (m, 12H, $((CH_2)_2O)_3$), 2.39 (t, J = 2.4 Hz, 1H, $OCH_2C≡CH$); ^{13}C NMR ($CDCl_3$, 75 MHz) δ 190.3 (CHO), 169.5 (NC=O), 167.1 (C Ar), 135.5 (C Ar), 128.8 (C Ar), 127.9 (C Ar), 119.2 (C Ar), 114.8 (C Ar), 79.4 ($OCH_2C≡CH$), 74.9 ($OCH_2C≡CH$), 70.7, 70.3, 70.3, 69.4, 69.2, 58.4 ($OCH_2C≡CH$), 39.8; HRMS: *m/z* calcd for $C_{17}H_{21}NO_6Na$: 358.1261 $[M+Na]^+$, found 358.1261.

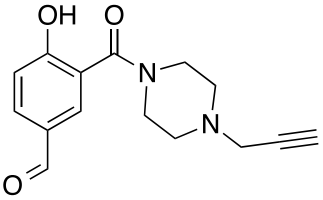

4-hydroxy-3-(4-(prop-2-yn-1-yl)piperazine-1-carbonyl)benzaldehyde 2c was obtained in a different manner following this synthetical scheme :

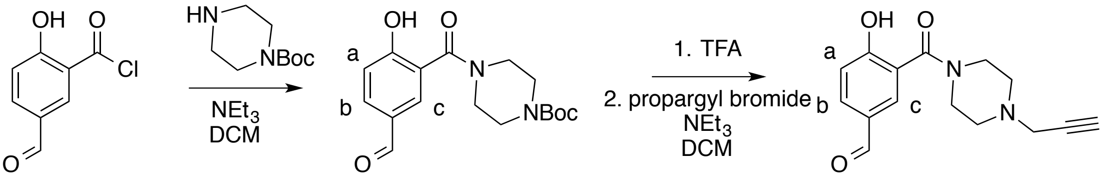

2c' **2c**

5-formyl-2-hydroxybenzoic acid (2 g, 12.04 mmol, 1 eq) was placed under argon and $SOCl_2$ (20 mL, 274.1 mmol, 23 eq) was added. The mixture was stirred and heated to 85˚C overnight. The mixture was evaporated to dryness and left overnight under vacuum to yield the aromatic acyl chloride product as a white solid (2.22 g, 12.02 mmol, 99%).

To a solution of the previously synthesized aromatic acyl chloride (2.22 g, 12.03 mmol, 1 eq) in DCM (60 mL) was added a solution of tert-butyl-1-piperazinecarboxylate (2.70, 14.43 mmol, 1.2 eq) in DCM (30 mL) and triethylamine (5.1 mL, 36.9 mmol, 3 eq). THF (20 mL) was added to the inhomogeneous solution to

try and aid solubility however no change was seen. The reaction mixture was left stirring overnight and evaporated to dryness. The crude product was treated with DCM/HCl (1M) and a precipitate formed. The solid was filtered off and the filtrate further extracted with DCM. The combined organic phases were washed with $NaHCO_3$, dried over $MgSO_4$ and evaporated to dryness. The solid was purified by column chromatography on silica gel (DCM/MeOH, 97/3) to yield **2c'** as a yellow solid (1.31 g, 3.91 mmol, 33%). ^{1}**H NMR** (300 MHz, Chloroform-d) δ 10.45 (s, 1H, O<u>H</u>), 9.79 (s, 1H, C<u>H</u>O), 7.80 (dd, J_{a-b} = 8.1 Hz , J_{c-b} = 1.8 Hz, 1H, H_b), 7.75 (d, J_{b-c} = 2.1 Hz, 1H, H_c), 7.07 (d, J_{a-b} = 8.5 Hz, 1H, H_a), 3.66 - 3.38 (m, 8H, $(CH_2)_4$), 1.42 (s, 9H, $(Me)_3$). ^{13}**C NMR** (75 MHz, $CDCl_3$) δ 189.91 (<u>C</u>HO), 170.09 (<u>C</u>ON), 164.49 (COO^{t}Bu), 154.46 (C Ar), 134.74 (C Ar), 130.26 (C Ar), 127.97 (C_b), 118.90 (C_c), 116.96 (C_a), 80.72 (<u>C</u>$(Me)_3$), 45.71 (CH_2), 43.56 (CH_2), 28.38 (Me).

To a solution of **2c'** (200 mg, 0.60 mmol, 1 eq) in DCM (10 mL) was added TFA (0.23 mL, 2.98 mmol, 5 eq). The solution was stirred at 0˚C for 1h before being left under ultrasound at room temperature for a further 1h. A further 22 eq TFA was added and the reaction stirred for 1h. The reaction mixture was poured into diethyl ether (125 mL) to form a sticky, white precipitate. The solid was filtered, redissolved in MeOH and evaporated to dryness to yield the corresponding ammonium salt as a white solid (90 mg, 0.26 mmol, 43%). To a solution of this ammonium salt (156 mg, 0.45 mmol, 1 eq) in DCM (5 mL) was added triethylamine (0.40 ml, 2.69 mmol, 6 eq) and propargyl bromide (80 % in toluene) (0.20 mL, 1.34 mmol, 3 eq) to form an inhomogeneous solution. The mixture was stirred for 24h before a further 3 eq -bromo-2-propyne and 6 eq triethylamine were added. The mixture was stirred for a further 15h and the mixture evaporated to dryness. The solid was purified by column chromatography on silica gel (DCM/MeOH, 95/5) via solid deposition to yield the product **2c** (101 mg, 0.37 mmol, 82%). ^{1}**H NMR** (300 MHz, Chloroform-*d*) δ 9.88 (s, 1H, C<u>H</u>O), 7.91 – 7.81 (m, 2H, H_b, H_c), 7.13 (d, J_{a-b} = 8.4 Hz, 1H, H_a), 3.82 (t, *J* = 5.0 Hz, 4H, CON<u>$(CH_2)_2$</u>), 3.40 (d, *J* = 2.5 Hz, 2H, N<u>CH_2</u>), 2.68 (t, *J* = 5.0 Hz, 4H, N<u>$(CH_2)_2$</u>), 2.33 (s, 1H, H-alkyne). ^{13}**C NMR** (75 MHz, $CDCl_3$) δ 190.07 (<u>C</u>HO), 169.57 (<u>C</u>ON), 164.37 (Ar<u>C</u>OH), 134.46 (C Ar), 130.43 (C Ar), 127.88 (C Ar), 118.76 (C Ar), 117.38 (C Ar), 77.77 (CH_2<u>C</u>CH), 74.10 (CH_2C<u>C</u>H), 51.62 (C-pip), 46.73 (<u>C</u>H_2), 45.57.

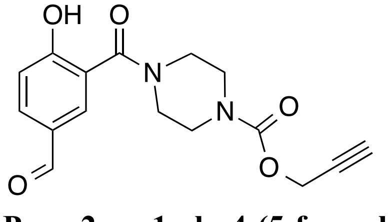

Prop-2-yn-1-yl 4-(5-formyl-2-hydroxybenzoyl)piperazine-1-carboxylate 2d: 1 (0.280 g, 1.06 mmol); 4-((prop-2-yn-1-yloxy)carbonyl)piperazin-1-ium 2,2,2-trifluoroacetate (0.360 g, 1.28 mmol); DIEA (0.650 mL, 3.71 mmol). **2d** (0.216 g, 64 %) as a yellowish amorphous solid after flash chromatography (cyclohexane/ethyl acetate 1:4 to 1:9); ^{1}H NMR ($CDCl_3$ + εCD_3OD, 300 MHz) δ 9.81 (s, 1H, CHO), 7.80 (d, $J_{b,a}$ = 8.4 Hz, 1H, Hb), 7.76 (s, 1H, Hc), 7.01 (d, $J_{a,b}$ = 8.4 Hz, 1H, Ha), 4.70 (s,

2H, OC$\underline{H_2}$C≡CH), 3.64 – 3.55 (m, 8H, $(CH_2)_4$), 2.48 (s, 1H, OCH_2C≡C$\underline{H}$); ^{13}C NMR ($CDCl_3$ + εCD_3OD, 75 MHz) δ 190.2 (CHO), 169.2 (NC=O), 162.1 (C Ar), 154.4 (NC=O), 133.9 (C Ar), 130.7 (C Ar), 128.6 (C Ar), 120.1 (C Ar), 117.9 (C Ar), 78.1 ($OCH_2\underline{C}$≡CH), 75.1 (OCH_2C≡$\underline{C}$H), 53.5 ($(CH_2)_4$), 43.9 (OC$\underline{H_2}$C≡CH); HRMS: *m/z* calcd for $C_{16}H_{16}N_2O_5Na$: 339.0551 $[M+Na]^+$, found 339.0550.

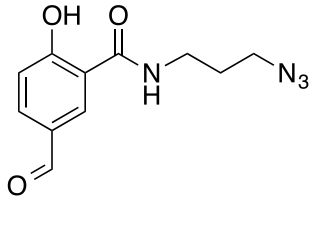

N-(3-azidopropyl)-5-formyl-2-hydroxybenzamide 2e: 1 (0.355 g, 1.35 mmol); 3-azidopropylamine (0.202 g, 2.02 mmol); DIEA (0.880 mL, 5.06 mmol). **2e** (0.190 g, 57 %) as a white amorphous solid after flash chromatography (cyclohexane/ethyl acetate 4:1 to 3:1); ^{1}H NMR ($CDCl_3$, 300MHz) δ 13.35 (s, 1H, OH), 9.85 (s, 1H, CHO), 8.25 (d, $J_{c,b}$ = 1.8 Hz, 1H, Hc), 7.87 (dd, $J_{b,a}$ = 8.6 Hz, $J_{b,c}$ = 1.9 Hz, 1H, Hb), 7.57 (bs, 1H, NH), 7.08 (d, $J_{a,b}$ = 8.6 Hz, 1H, Ha), 3.58 (dt, J = 6.6 Hz, J = 6.1 Hz, 2H, NHC$\underline{H_2}$), 3.46 (t, J = 6.5 Hz, 2H, C$\underline{H_2}N_3$), 1.94 (quint, J = 6.6 Hz, 2H, $CH_2C\underline{H_2}CH_2N_3$); ^{13}C NMR ($CDCl_3$, 75 MHz) δ 190.3 (CHO), 169.4 (NC=O), 167.1 (C Ar), 136.5 (C Ar), 127.6 (C Ar), 127.2 (C Ar), 119.2 (C Ar), 114.6 (C Ar), 49.3 ($(CH_2)_2C\underline{H_2}N_3$), 37.5 ($C\underline{H_2}(CH_2)_2N_3$), 28.4 ($CH_2C\underline{H_2}CH_2N_3$); HRMS: *m/z* calcd for $C_{11}H_{12}N_4O_3Na$: 271.0802 $[M+Na]^+$, found 271.0803.

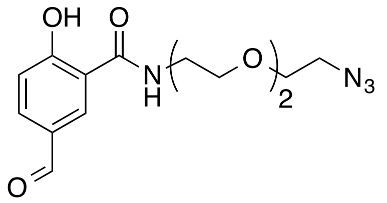

N-(2-(2-(2-azidoethoxy)ethoxy)ethyl)-5-formyl-2-hydroxybenzamide 2f: 1 (0.277 g, 1.05 mmol); 2-(2-azidoethoxy)ethanamine (0.221 g, 1.27 mmol); DIEA (0.460 mL, 2.63 mmol). **2f** (0.248 g, 73 %) as a yellowish syrup after flash chromatography (cyclohexane/ethyl acetate 3:2 to 1:1); ^{1}H NMR ($CDCl_3$, 300MHz) δ 13.25 (s, 1H, OH), 9.84 (s, 1H, CHO), 8.05 (d, $J_{c,b}$ = 1.8 Hz, 1H, Hc), 7.87 (dd, $J_{b,a}$ = 8.6 Hz, $J_{b,c}$ = 1.9 Hz, 1H, Hb), 7.26 (bs, 1H, NH), 7.06 (d, $J_{a,b}$ = 8.6 Hz, 1H, Ha), 3.73 – 3.64 (m, 10H, $((CH_2)_2O)_2CH_2$), 3.37 (t, J = 5.0 Hz, 1H, CH_2N_3); ^{13}C NMR ($CDCl_3$, 75 MHz) δ 190.2 (CHO), 169.4 (NC=O), 167.1 (C Ar), 136.0 (C Ar), 127.9 (2C, 2C Ar), 119.3 (C Ar), 114.7 (C Ar), 70.7, 70.5, 70.2, 69.4, 39.7; HRMS: *m/z* calcd for $C_{14}H_{18}N_4O_5Na$: 345.1169 $[M+Na]^+$, found 345.1168.

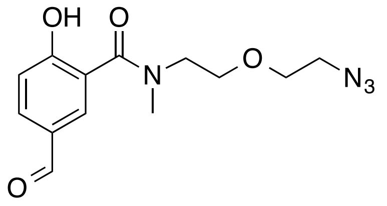

N-(2-(2-(2-azidoethoxy)ethoxy)ethyl)-5-formyl-2-hydroxy-N-methylbenzamide 2g: 1 (0.163 g, 0.619 mmol); 2-(2-azidoethoxy)-N-methylethanamine (0.140 g, 0.743 mmol); DIEA (0.270 mL, 1.55 mmol). **2g** (0.162 g, 78 %) as a yellowish syrup after flash chromatography (cyclohexane/ethyl acetate 1:1 to 2:3); ^{1}H NMR ($CDCl_3$, 300MHz) δ 10.64 (bs, 1H, OH), 9.86 (s, 1H, CHO), 8.04 (s, 1H, Hc), 7.86 (dd, $J_{b,a}$ = 8.6 Hz, $J_{b,c}$ = 1.9 Hz, 1H, Hb), 7.11 (d, $J_{a,b}$ = 8.5 Hz, 1H, Ha), 3.80 – 3.75 (m, 4H, $((CH_2)_2O)_2CH_2$), 3.68 – 3.65 (m, 6H, $((CH_2)_2O)_2CH_2$), 3.38 (t, J = 4.9 Hz, 1H, CH_2N_3), 3.26 (s, 3H, CH_3); ^{13}C NMR ($CDCl_3$, 75 MHz) δ 190.3 (CHO), 164.7 (NC=O), 134.3 (C Ar), 131.1 (C Ar), 128.0 (C Ar), 118.8 (C Ar), 118.0 (C Ar), 77.4, 70.8, 70.3, 68.7, 50.9; HRMS: *m/z* calcd for $C_{15}H_{20}N_4O_5Na$: 359.1326 $[M+Na]^+$, found 359.1327.

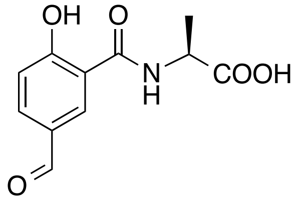

(S)-2-(5-formyl-2-hydroxybenzamido)propanoic acid 2h: 1 (0.210 g, 0.798 mmol); L-alanine (0.107 g, 1.20 mmol); DIEA (0.420 mL, 2.39 mmol). **2h** (0.095 g, 50 %) as a white amorphous solid after flash chromatography (DCM/acetone (0.5 % TFA v/v) 4:1 to 7:3); ^{1}H NMR ($(CD_3)_2CO$, 300MHz) δ 13.28 (bs, 1H, COOH), 9.86 (s, 1H, CHO), 8.68 (d, J = 5.3 Hz, 1H, OH), 8.44 (s, 1H, Hc), 7.98 (dd, $J_{b,a}$ = 8.6 Hz, $J_{b,c}$ = 1.3 Hz, 1H, Hb), 7.08 (d, $J_{a,b}$ = 8.6 Hz, 1H, Ha), 4.74 (quint, J = 7.3 Hz, 1H, $NC\underline{H}(CH_3)CO_2H$), 1.57 (d, J = 7.3 Hz, 1H, CH_3); ^{13}C NMR ($(CD_3)_2CO$, 75 MHz) δ 190.6 (CHO), 173.6 (CO_2H), 170.3 (NC=O), 167.5 (C Ar), 135.7 (C Ar), 130.7 (C Ar), 127.4 (C Ar), 119.5 (C Ar), 115.4 (C Ar), 49.2 ($NH\underline{C}H(CH_3)CO_2H$), 17.4 ($CH_3$); HRMS: *m/z* calcd for $C_{11}H_{11}NO_5Na$: 260.0529 $[M+Na]^+$, found 260.0532.

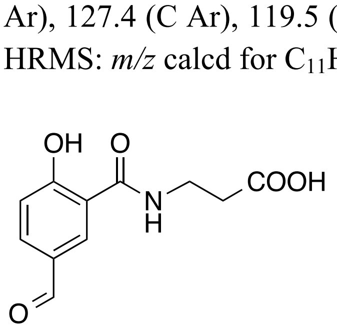

3-(5-formyl-2-hydroxybenzamido)propanoic acid 2i: 1 (0.243 g, 0.923 mmol); β-alanine (0.123 g, 1.38 mmol); DIEA (0.560 mL, 3.23 mmol). **2i** (0.055 g, 26 %) as a white amorphous solid after flash chromatography (DCM/acetone (0.5 % TFA v/v)

9:1 to 4:1); ^{1}H NMR ($(CD_3)_2CO$, 300MHz) δ 13.28 (bs, 1H, COOH), 9.86 (s, 1H, CHO), 8.68 (d, J = 5.3 Hz, 1H, OH), 8.44 (s, 1H, Hc), 7.98 (dd, $J_{b,a}$ = 8.6 Hz, $J_{b,c}$ = 1.3 Hz, 1H, Hb), 7.08 (d, $J_{a,b}$ = 8.6 Hz, 1H, Ha), 3.76 – 3.70 (m, 2H, $NHC\underline{H_2}CH_2CO_2H$), 2.73 (t, J = 6.7 Hz, 2H, $NHCH_2C\underline{H_2}CO_2H$); ^{13}C NMR ($(CD_3)_2CO$, 75 MHz) δ 190.6 (CHO), 173.0 (CO_2H), 170.6 (NC=O), 167.7 (C Ar), 135.7 (C Ar), 130.3 (C Ar), 129.2 (C Ar), 119.6 (C Ar), 115.5 (C Ar), 36.5, 33.7; HRMS: *m/z* calcd for $C_{11}H_{12}NO_5$: 238.0710 $[M+H]^+$, found 238.0710.

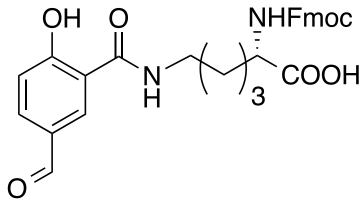

(S)-2-((((9H-fluoren-9-yl)methoxy)carbonyl)amino)-6-(5-formyl-2-hydroxybenzamido)hexanoic acid 2j: 1 (0.255 g, 0.968 mmol); Fmoc-Lys-OH (0.535 g, 1.45 mmol); DIEA (0.600 mL, 3.39 mmol). **2j** (0.128 g, 26 %) as a brownish foam after flash chromatography (cyclohexane/ethyl acetate (0.5 % TFA v/v) 7:3 to 1:1); ^{1}H NMR ($(CD_3)_2CO$, 300MHz) δ 9.81 (s, 1H, CHO), 8.61 (bs, 1H, OH or NH), 8.34 (bs, 1H, OH or NH), 7.93 (d, I = 7.4 Hz, 1H, H Ar), 7.85 – 7.82 (m, 2H, 2H Ar), 7.70 – 7.68 (m, 2H, 2H Ar), 7.41 – 7.28 (m, 4H, 4H Ar), 7.04 (d, J = 7.7 Hz, 1H, H Ar), 6.75 (d, J = 3.6 Hz, 1H, H Ar), 4.33 – 4.22 (m, 4H), 3.51 (s, 2H), 1.96 – 1.94 (m, 1H), 1.84 – 1.72 (m, 3H), 1.60 – 1.56 (m, 2H); ^{13}C NMR ($(CD_3)_2CO$, 75 MHz) δ 190.6 (CHO), 170.5, 167.8, 145.1, 145.0, 142.1, 135.5, 130.1, 129.1, 128.5, 127.9, 126.1, 120.8, 119.5, 115.7, 67.2, 48.0, 40.1, 32.3, 24.0; HRMS: *m/z* calcd for $C_{29}H_{28}N_2O_7Na$: 539.1789 $[M+Na]^+$, found 539.1786.

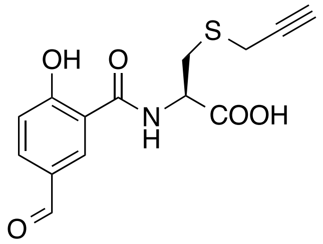

(R)-2-(5-formyl-2-hydroxybenzamido)-3-(prop-2-yn-1-ylthio)propanoic acid 2k: 1 (0.125 g, 0.475 mmol); S-propargyl cysteine (0.113 g, 0.712 mmol); DIEA (0.250 mL, 1.43 mmol). **2k** (0.087 g, 59 %) as a brownish syrup after flash chromatography (DCM/acetone (0.5 % TFA v/v) 95:5 to 85:15); ^{1}H NMR ($(CD_3)_2CO$, 300MHz) δ 13.14 (bs, 1H, COOH), 9.88 (s, 1H, CHO), 8.82 (d, J = 7.6 Hz, 1H, OH), 8.47 (d, $J_{c,b}$ = 2.1 Hz, 1H, Hc), 8.02 (dd, $J_{b,a}$ = 8.5 Hz, $J_{b,c}$ = 2.0 Hz, 1H, Hb), 7.12 (d, $J_{a,b}$ = 8.7 Hz, 1H, Ha), 5.04 – 4.96 (m, 1H, $NHC\underline{H}(CH_2SCH_2C{\equiv}CH)CO_2H$), 3.56 – 3.39 (m, 3H, $CH_2SC\underline{H_2}C{\equiv}CH$, $C\underline{H_2}SCH_2C{\equiv}CH$), 3.18 (dd, ^2J = 14.1 Hz, J = 9.4 Hz, 1H, $C\underline{H_2}SCH_2C{\equiv}CH$), 2.77 (t, J = 2.6 Hz, 1H, $SCH_2C{\equiv}C\underline{H}$); ^{13}C NMR DEPT 135 ($(CD_3)_2CO$, 75 MHz) δ 190.6 (CHO), 136.0 (C Ar), 130.7 (C Ar), 119.6 (C Ar), 73.2, 52.8, 33.3, 19.7.**HRMS:** *m/z* calcd for $C_{14}H_{13}NO_6SNa$: 346.0356 $[M+Na]^+$, found

346.0556. Mass spectrometry could only detect the sulfoxyde product M+O probably due to oxidation in the source since the mass spectrum of the corresponding **HR-CysA** was found to be correct.

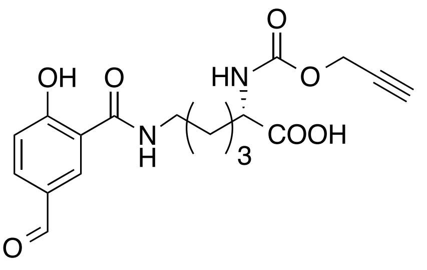

(S)-6-(5-formyl-2-hydroxybenzamido)-2-(((prop-2-yn-1 yloxy)carbonyl)amino)hexanoic acid 2l: 1 (0.140 g, 0.536 mmol); 3-carboxy-3-(((prop-2-yn-1-yloxy)carbonyl)amino)propan-1-aminium 2,2,2-trifluoroacetate (0.275 g, 0.803 mmol); DIEA (0.470 mL, 2.68 mmol). **2l** (0.170 g, 84 %) as a brownish foam after flash chromatography (DCM/acetone (0.5 % TFA v/v) 95:5 to 85:15); ^{1}H NMR ($CDCl_3$, 300MHz) δ 9.78 (s, 1H, CHO), 8.21 (s, 1H, Hc), 7.83 (d, $J_{b,a}$ = 8.3 Hz, 1H, Hb), 7.69 (bs, 1H, OH), 7.03 (d, $J_{a,b}$ = 8.5 Hz, 1H, Ha), 5.86 (d, J = 7.0 Hz, 1H, NH), 4.61 (s, 2H, OC$\underline{H_2}$C≡CH), 4.05 – 4.01 (m, 1H, $CH_2C\underline{H}(CO_2H)NH$), 2.44 (s, 1H, $OCH_2C{\equiv}C\underline{H}$), 1.94 – 1.85 (m, 1H), 1.78 – 1.63 (m, 3H), 1.49 – 1.45 (m, 2H); ^{13}C NMR ($CDCl_3$ + εCD_3OD, 75 MHz) δ 191.2 (CHO), 174.6 (CO_2H), 169.5 (NC=O), 167.0 (C Ar), 155.7 (NC=O), 136.0 (C Ar), 128.8 (C Ar), 127.7 (C Ar), 119.1 (C Ar), 115.2 (C Ar), 77.9 ($OCH_2\underline{C}{\equiv}CH$), 75.1 ($OCH_2C{\equiv}\underline{C}H$), 52.9 ($O\underline{C}H_2C{\equiv}CH$), 39.6, 39.4, 32.0, 28.4, 22.6; HRMS: *m/z* calcd for $C_{18}H_{20}N_2O_7Na$: 399.1163 $[M+Na]^+$, found 399.1164.

General procedure for the preparation of the functionalisable H-Rubies.

To a mixture of aldehyde **2a** – **2l** and 8-hydroxyjulolidine (2 equiv) in DCM (C = 0.1 - 0.2 M) was added triflic acid (0.3 equiv). The solution was stirred in the dark at room temperature until quantitative consumption of the starting material (Friedel-Craft condensation) then *p*-chloranil (1 equiv) was added (oxidation). After stirring for 3 - 16 h at room temperature, the mixture was concentrated then submitted to flash chromatography (elution with DCM/MeOH mixture containing 0.5 % v/v of TFA).

General numerotation for NMR:

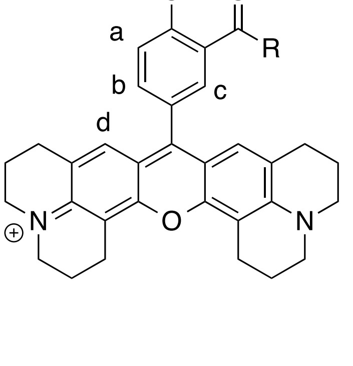

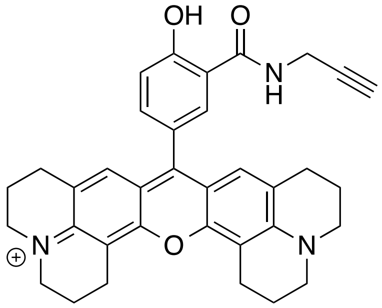

HR-A. 2a (0.030 g, 0.147 mmol); reaction time: 6 h for the condensation, 2 h for the oxidation. **HR-A** (0.060 g, 62 %) as a purple solid after flash chromatography (DCM/methanol (0.5 % TFA v/v) 100:0 to 94:6); ^{1}H NMR (CD_3OD, 300MHz) δ 7.80 (d, $J_{c,b}$ = 2.1 Hz, 1H, Hc), 7.35 (dd, $J_{b,a}$ = 8.4 Hz, $J_{b,c}$ = 2.1 Hz, 1H, Hb), 7.11 (d, $J_{a,b}$ = 8.5 Hz, 1H, Ha), 6.87 (s, 2H, 2Hd), 4.12 (d, *J* = 2.3 Hz, 2H, NC$\underline{H_2}$C≡CH), 3.57 – 3.48 (m, 8H), 3.06 (t, *J* = 6.3 Hz, 4H), 2.69 (t, *J* = 5.9 Hz, 4H), 2.62 (t, *J* = 2.2 Hz, 1H, NCH_2C≡C$\underline{H}$), 2.14 – 2.06 (m, 4H), 1.97 – 1.90 (m, 4H); ^{13}C NMR (CD_3OD, 75 MHz) δ 169.0, 161.6, 155.2, 153.6, 152.4, 136.0, 131.3, 127.6, 125.3, 124.7, 118.9, 117.6, 114.0, 106.7, 80.4, 72.4, 51.9, 51.4, 29.7, 28.6, 21.8, 21.0, 20.8; HRMS: *m/z* calcd for $C_{35}H_{34}N_3O_3^+$: 544.2595 $[M]^+$, found 544.2590.

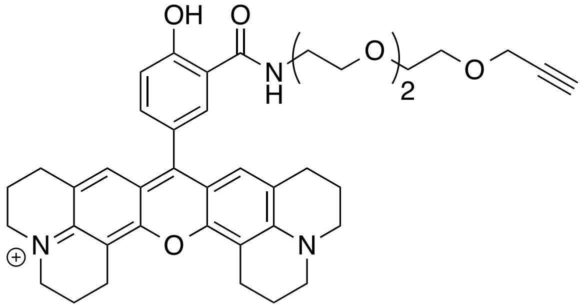

HR-PA: 2b (0.449 g, 1.34 mmol); reaction time: 15 h for the condensation, 24 h for the oxidation. **HR-PA** (0.805 g, 76 %) as a purple solid after flash chromatography (DCM/methanol (0.5 % TFA v/v) 100:0 to 95:5); ^{1}H NMR (CD_3OD, 300MHz) δ 7.86 (d, $J_{c,b}$ = 2.1 Hz, 1H, Hc), 7.41 (dd, $J_{b,a}$ = 8.5 Hz, $J_{b,c}$ = 2.2 Hz, 1H, Hb), 7.17 (d, $J_{a,b}$ = 8.4 Hz, 1H, Ha), 6.92 (s, 2H, 2Hd), 4.02 (d, *J* = 2.5 Hz, 2H, OC$\underline{H_2}$C≡CH), 3.69 – 3.50 (m, 20H), 3.08 (t, *J* = 6.4 Hz, 4H), 2.81 (t, *J* = 2.4 Hz, 1H, OCH_2C≡C$\underline{H}$), 2.74 (t, *J* = 6.0 Hz, 4H), 2.15 – 2.07 (m, 4H), 2.00 – 1.93 (m, 4H); ^{13}C NMR (CD_3OD, 75 MHz) δ 169.8, 162.1, 155.5, 153.7, 152.5, 135.8, 130.9, 127.7, 125.4, 124.7, 119.0, 117.7, 114.1, 106.7, 80.5, 76.0, 71.6, 71.3, 70.4, 70.0, 58.9, 51.9, 51.4, 40.7, 28.6, 21.8, 21.0, 20.9; HRMS: *m/z* calcd for $C_{41}H_{46}N_3O_6^+$: 676.3381 $[M]^+$, found 676.3381.

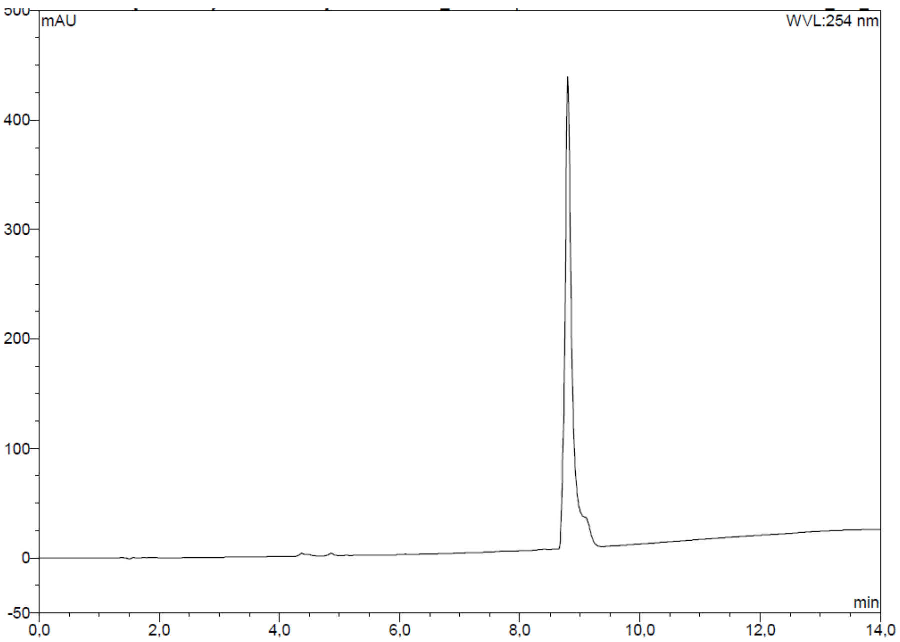

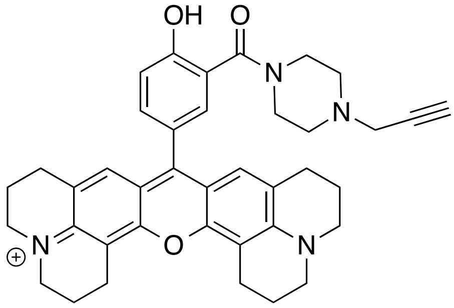

HR-PiA. Obtained from **2c** following the non optimised general procedure (82.3 mg, 0.11 mmol, 28%). **MS (ES+):** calcd for $C_{39}H_{41}N_4O_3^+$ [M]$^+$, 613.32; found 613.6. HRMS: *m/z* calcd for $C_{39}H_{41}N_4O_3^+$: 613.3173 [M]$^+$, found. 613.3171

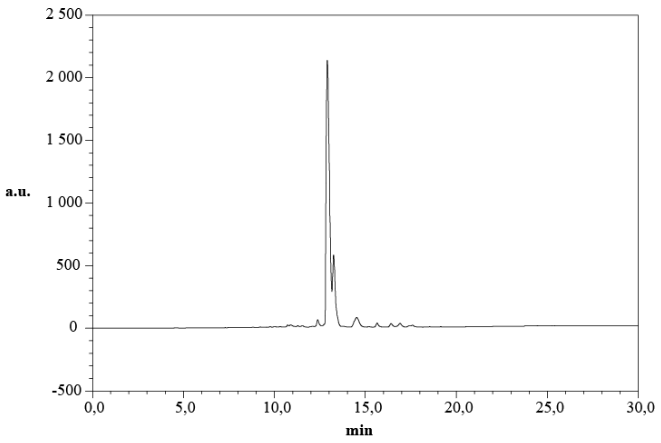

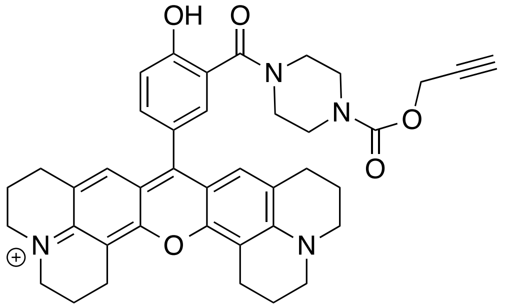

HR-PiAC. 2d (0.090 g, 0.285 mmol); reaction time: 14 h for the condensation, 3 h 30 for the oxidation. **HR-PiAC** (0.176 g, 80 %) as a purple solid after flash chromatography (DCM/methanol (0.5 % TFA v/v) 100:0 to 95:5); ^{1}H NMR ($CDCl_3$ + εCD_3OD, 300 MHz) δ 7.35 (dd, $J_{b,a}$ = 8.5 Hz, $J_{b,c}$ = 2.2 Hz, 1H, Hb), 7.26 (d, $J_{c,b}$ = 2.2 Hz, 1H, Hc), 7.14 (d, $J_{a,b}$ = 8.3 Hz, 1H, Ha), 7.01 (s, 2H, 2Hd), 4.73 (d, J = 2.4 Hz, 2H, OC$\underline{H_2}$C≡CH), 3.60 – 3.51 (m, 12H), 3.35 (s, 8H, $(CH_2)_4$), 3.07 (t, *J* = 6.3 Hz, 4H), 2.94 (t, *J* = 2.5 Hz, 1H, $OCH_2C≡C\underline{H}$), 2.77 (t, *J* = 5.93 Hz, 4H), 2.14 – 2.06 (m, 4H), 2.02 – 1.94 (m, 4H); ^{13}C NMR (CD_3OD, 75 MHz) δ 169.6, 156.4, 155.9, 155.3, 153.6, 152.4, 133.8, 131.1, 129.8, 129.1, 127.7, 126.2, 125.3, 125.2, 124.7, 117.2, 113.9, 106.6, 79.1, 76.3, 54.2, 51.8, 51.3, 28.5, 21.8, 21.1, 20.8; HRMS: *m/z* calcd for $C_{40}H_{41}N_4O_5^+$: 657.3072 $[M]^+$, found 657.3071.

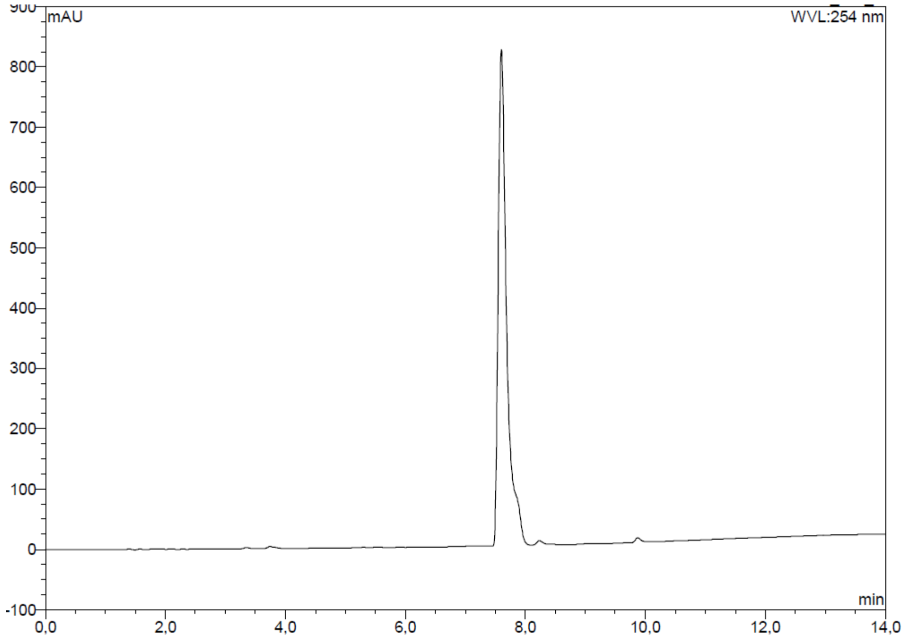

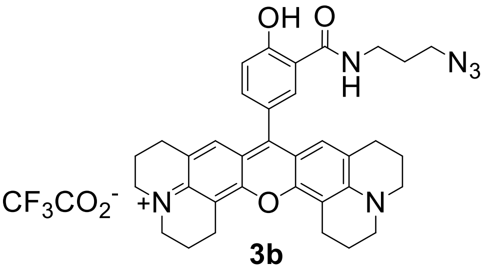

3b

HR-N$_3$. 2e (0.100 g, 0.403 mmol); reaction time: 6 h for the condensation, 16 h for the oxidation. **HR-N$_3$** (0.167 g, 59 %) as a purple solid after flash chromatography (DCM/methanol (0.5 % TFA v/v) 100:0 to 95:5); ^{1}H NMR (CD_3OD, 300MHz) δ 7.81 (d, $J_{c,b}$ = 2.1 Hz, 1H, Hc), 7.36 (dd, $J_{b,a}$ = 8.4 Hz, $J_{b,c}$ = 2.1 Hz, 1H, Hb), 7.11 (d, $J_{a,b}$ = 8.5 Hz, 1H, Ha), 6.88 (s, 2H, 2Hd), 3.57 – 3.49 (m, 8H), 3.45 – 3.36 (m, 4H, $C\underline{H_2}CH_2C\underline{H_2}N_3$), 3.06 (t, J = 6.3 Hz, 4H), 2.70 (t, J = 6.0 Hz, 4H), 2.14 – 2.06 (m, 4H), 1.98 – 1.90 (m, 4H), 1.94 (quint, J = 6.7 Hz, 2H, $CH_2C\underline{H_2}CH_2N_3$); ^{13}C NMR ($CDCl_3$, 75 MHz) δ 169.7, 161.9, 155.3, 153.6, 152.4, 135.8, 130.9, 127.6, 125.3, 124.6, 118.9, 117.7, 114.0, 106.7, 51.9, 51.4, 50.3, 38.2, 29.6, 28.6, 21.8, 21.0, 20.8; HRMS: *m/z* calcd for $C_{35}H_{37}N_6O_3^+$: 589.2922 [M]$^+$, found 589.2918.

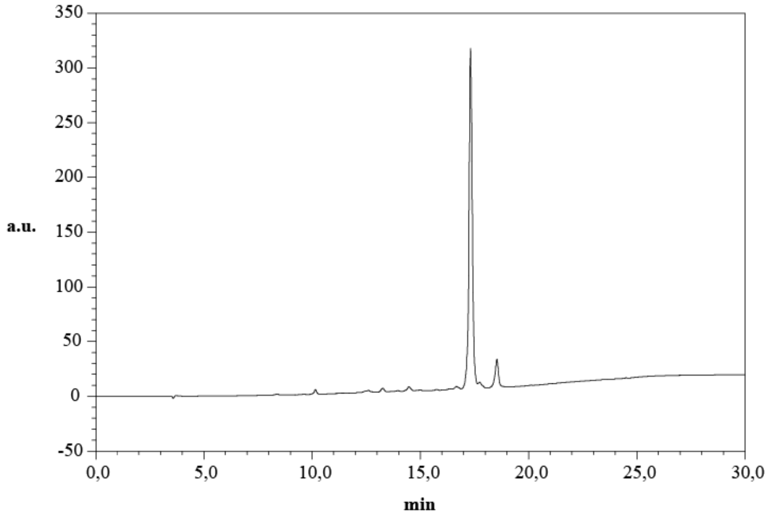

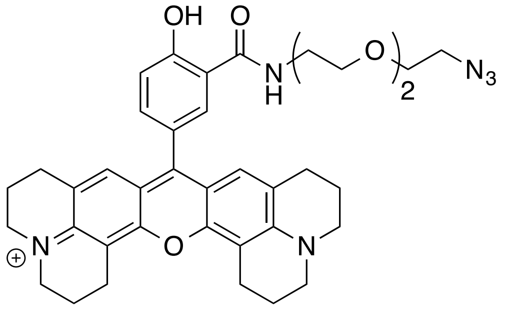

HR-PN$_3$. 2f (0.180 g, 0.558 mmol); reaction time: 16 h for the condensation, 7 h for the oxidation. **HR-PN$_3$** (0.321 g, 74 %) as a purple solid after flash chromatography (DCM/methanol (0.5 % TFA v/v) 100:0 to 95:5); ^{1}H NMR (CD_3OD, 300MHz) δ 7.84 (d, $J_{c,b}$ = 2.1 Hz, 1H, Hc), 7.40 (dd, $J_{b,a}$ = 8.4 Hz, $J_{b,c}$ = 2.2 Hz, 1H, Hb), 7.16 (d, $J_{a,b}$ = 8.4 Hz, 1H, Ha), 6.92 (s, 2H, 2Hd), 3.69 – 3.50 (m, 18H), 3.25 (t, J = 5.0 Hz, 2H), 3.07 (t, J = 6.3 Hz, 4H), 2.74 (t, J = 6.0 Hz, 4H), 2.15 – 2.06 (m, 4H), 2.00 – 1.93 (m, 4H); ^{13}C NMR (CD_3OD, 75 MHz) δ 169.7, 161.2, 155.5, 153.7, 152.5, 135.7, 131.0, 127.7, 125.3, 124.7, 118.9, 117.9, 114.1, 106.7, 71.5, 71.4, 71.1, 70.5, 51.9, 51.7, 51.4, 40.6, 28.6, 21.8, 21.1, 20.9; HRMS: *m/z* calcd for $C_{38}H_{43}N_6O_5^+$: 663.3289 [M]$^+$, found 663.3291.

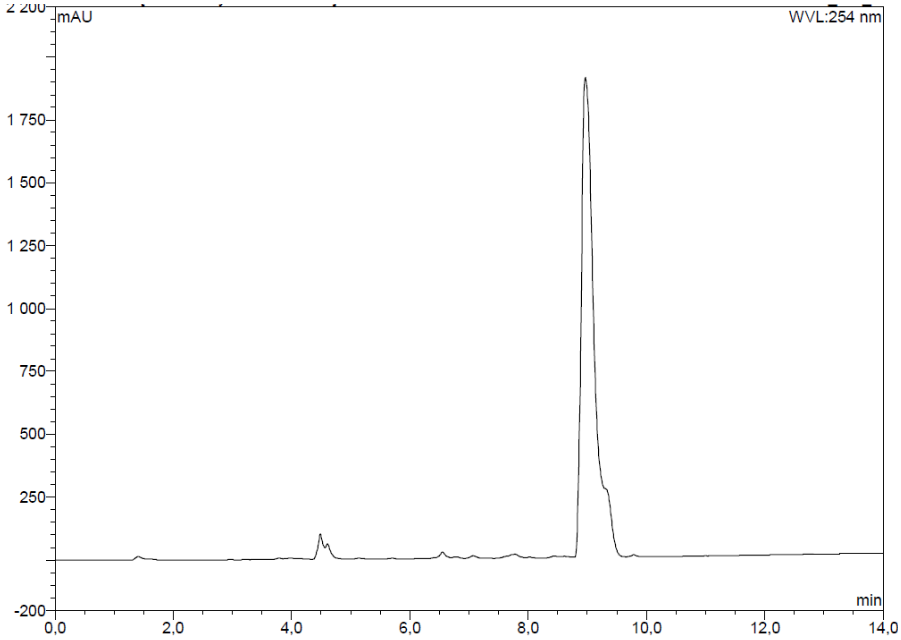

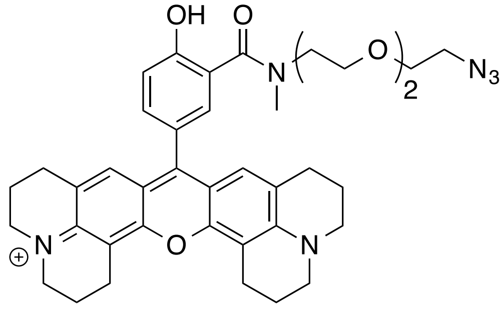

HR-MPN$_3$. 2g (0.155 g, 0.461 mmol); reaction time: 20 h for the condensation, 5 h for the oxidation. **HR-MPN$_3$** (0.339 g, 93 %) as a purple solid after flash chromatography (DCM/methanol (0.5 % TFA v/v) 100:0 to 94:6); ^{1}H NMR (CD_3OD, 300MHz) δ 7.31 – 7.23 (m, 2H, Hb, Hc), 7.12 (d, $J_{a,b}$ = 8.3 Hz, 1H, Ha), 7.01 (s, 2H, 2Hd), 3.76 – 3.36 (m, 18H), 3.24 – 3.14 (m, 5H), 3.06 (t, J = 6.3 Hz, 4H), 2.76 (t, J = 6.1 Hz, 4H), 2.14 – 2.06 (m, 4H), 2.02 – 1.94 (m, 4H); ^{13}C NMR (CD_3OD, 75 MHz) δ 153.7, 152.4, 127.9, 125.7, 125.3, 125.0, 117.1, 114.1, 106.7, 71.5, 70.9, 51.9, 51.6, 51.3, 28.6, 21.8, 21.0, 20.9; HRMS: *m/z* calcd for $C_{39}H_{45}N_6O_5^+$: 677.3446 $[M]^+$, found 677.3449.

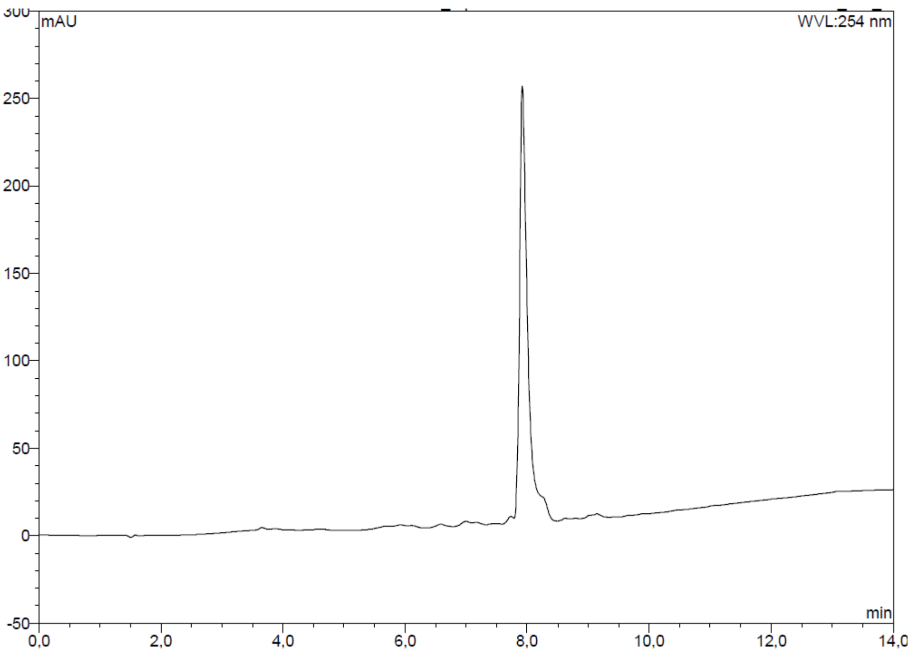

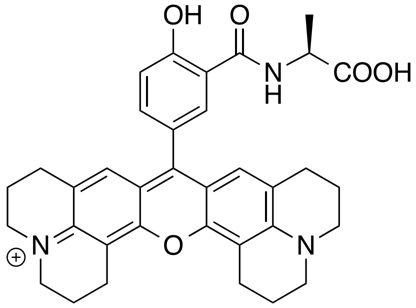

HR-Ala. 2h (0.090 g, 0.379 mmol); reaction time: 18 h for the condensation, 5 h 30 for the oxidation. **HR-Ala** (0.076 g, 29 %) as a purple solid after flash chromatography (DCM/methanol (0.5 % TFA v/v) 100:0 to 92:8); ^{1}H NMR (CD_3OD, 300MHz) δ 7.82 (s, 1H, Hc), 7.29 (dd, $J_{b,a}$ = 8.5 Hz, $J_{b,c}$ = 1.1 Hz, 1H, Hb), 7.04 (d, $J_{a,b}$ = 8.4 Hz, 1H, Ha), 6.78 (s, 2H, 2Hd), 4.47 – 4.40 (m, 1H, NHC<u>H</u>(CH_3)CO_2H), 3.49 – 3.41 (m, 8H), 2.98 (t, J = 6.0 Hz, 4H), 2.64 – 2.60 (m, 4H), 2.07 – 1.99 (m, 4H), 1.91 – 1.83 (m, 4H), 1.40 (d, J = 7.2 Hz, 3H, CH_3); ^{13}C NMR (CD_3OD, 75 MHz) δ 168.7, 161.3, 155.0, 153.4, 152.2 (2C), 135.6, 131.3, 127.4, 125.1, 125.0, 124.6, 118.7, 118.6, 117.5, 113.9, 113.8, 106.5, 51.6, 51.2, 28.4, 21.6, 20.8, 20.6, 17.7; HRMS: *m/z* calcd for $C_{35}H_{36}N_3O_5^+$: 578.2650 $[M]^+$, found 578.2644.

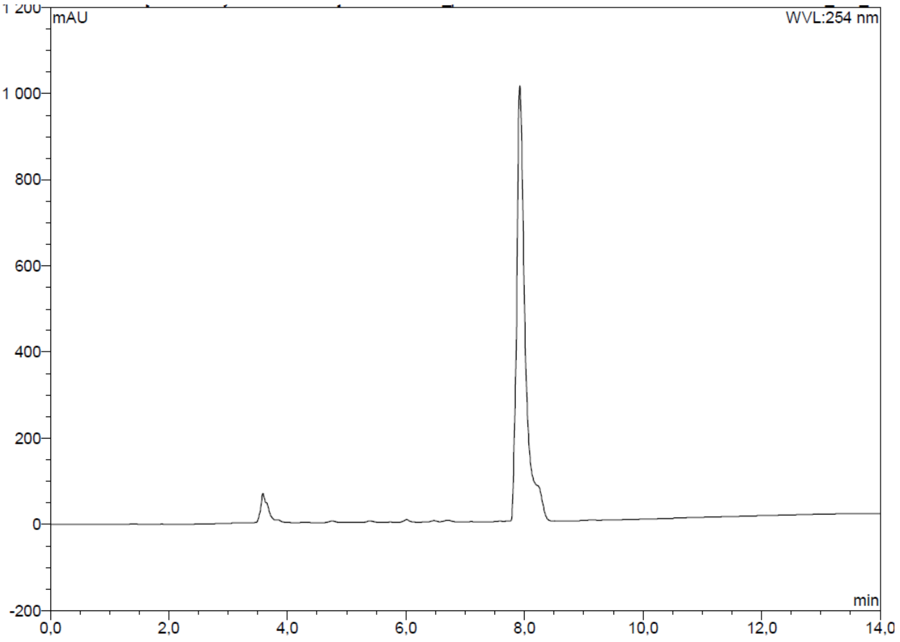

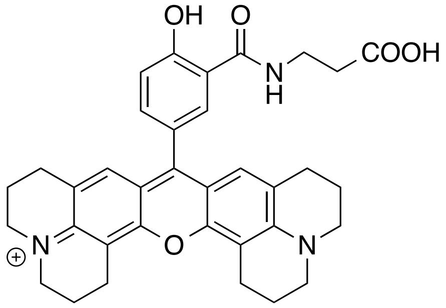

HR-βAla. 2i (0.032 g, 0.135 mmol); reaction time: 16 h for the condensation, 5 h for the oxidation. **HR-βAla** (0.034 g, 37 %) as a purple solid after flash chromatography (DCM/methanol (0.5 % TFA v/v) 98:2 to 93:7); ^{1}H NMR (CD_3OD, 300MHz) δ 7.80 (s, 1H, Hc), 7.36 (d, $J_{b,a}$ = 8.3 Hz, 1H, Hb), 7.12 (d, $J_{a,b}$ = 8.4 Hz, 1H, Ha), 6.87 (s, 2H, 2Hd), 3.65 – 3.59 (m, 2H), 3.54 – 3.47 (m, 4H), 3.03 (t, J = 6.3 Hz, 4H), 2.70 (t, J = 6.0 Hz, 4H), 2.60 (t, J = 6.8 Hz, 2H), 2.11 – 2.06 (m, 4H), 1.96 – 1.89 (m, 4H); ^{13}C NMR (CD_3OD, 75 MHz) δ 161.6, 155.3, 153.6, 152.4, 135.7, 131.1, 127.6, 125.2, 124.7, 118.8, 117.9, 114.0, 106.6, 51.8, 51.3, 36.5, 34.5, 30.6, 28.5, 21.7, 20.9, 20.8; HRMS: *m/z* calcd for $C_{35}H_{36}N_3O_5^+$: 578.2650 $[M]^+$, found 578.2649.

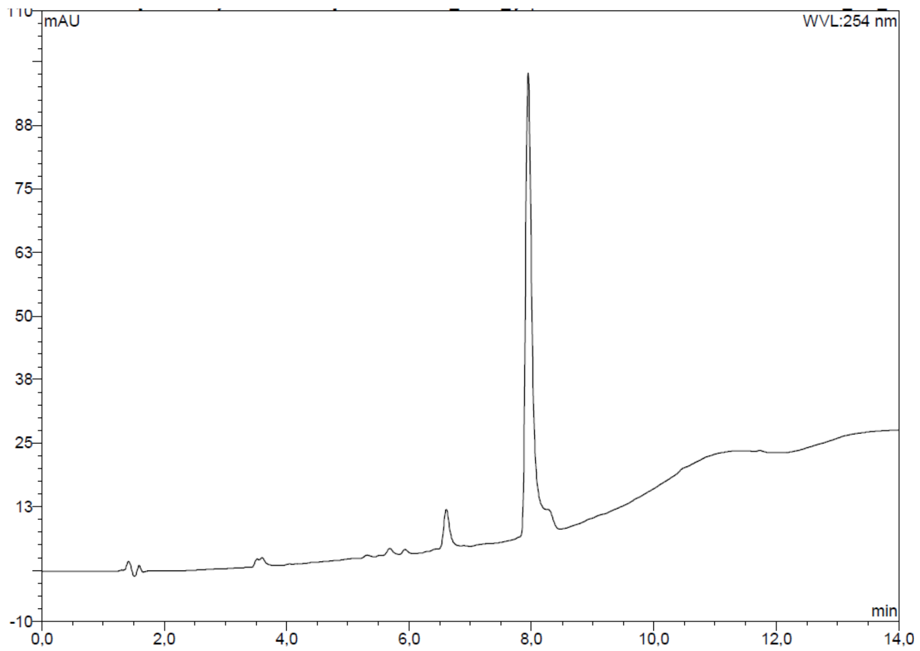

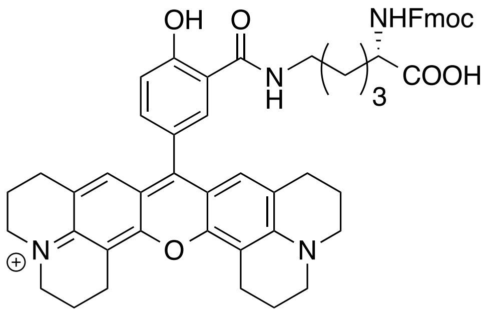

HR-LysF. 2j (0.090 g, 0.174 mmol); reaction time: 18 h for the condensation, 20 h for the oxidation. **HR-LysF** (0.056 g, 33 %) as a purple solid after flash chromatography (DCM/methanol (0.5 % TFA v/v) 100:0 to 92:8); ^{1}H NMR (CD_3OD, 300MHz) δ 8.02 (s, 1H, Hc), 7.58 – 7.55 (m, 2H, H Ar), 7.35 (d, J = 7.5 Hz, 1H, H Ar), 7.29 (d, J = 7.4 Hz, 1H, H Ar), 7.18 – 7.11 (m, 3H, H Ar), 7.05 (d, J = 8.3 Hz, 1H, H Ar), 7.01 – 6.95 (m, 2H, H Ar), 6.67 (s, 2H, Hd), 3.97 – 3.93 (m, 1H), 3.89 – 3.83 (m, 3H), 3.78 – 3.72 (m, 1H), 3.56 – 3.42 (m, 3H), 2.74 – 2.65 (m, 2H), 2.60 – 2.49 (m, 6H), 1.87 – 1.76 (m, 10H), 1.59 – 1.51 (m, 2H), 1.43 –1.36 (m, 2H); ^{13}C NMR (CD_3OD, 75 MHz) δ 168.9, 160.2, 158.2, 154.4, 152.9, 151.7, 144.7, 144.5, 141.8, 141.7, 135.4, 132.0, 128.3, 127.6, 127.0, 125.9, 125.6, 124.7, 124.5, 120.4, 118.5, 118.2, 113.5, 113.4, 106.1, 67.9, 54.7, 51.4, 50.8, 47.5, 38.6, 30.7, 29.9, 28.1, 22.9, 21.3, 20.4, 20.3; HRMS: *m/z* calcd for $C_{53}H_{53}N_4O_7^+$: 857.3909 $[M]^+$, found 857.3916.

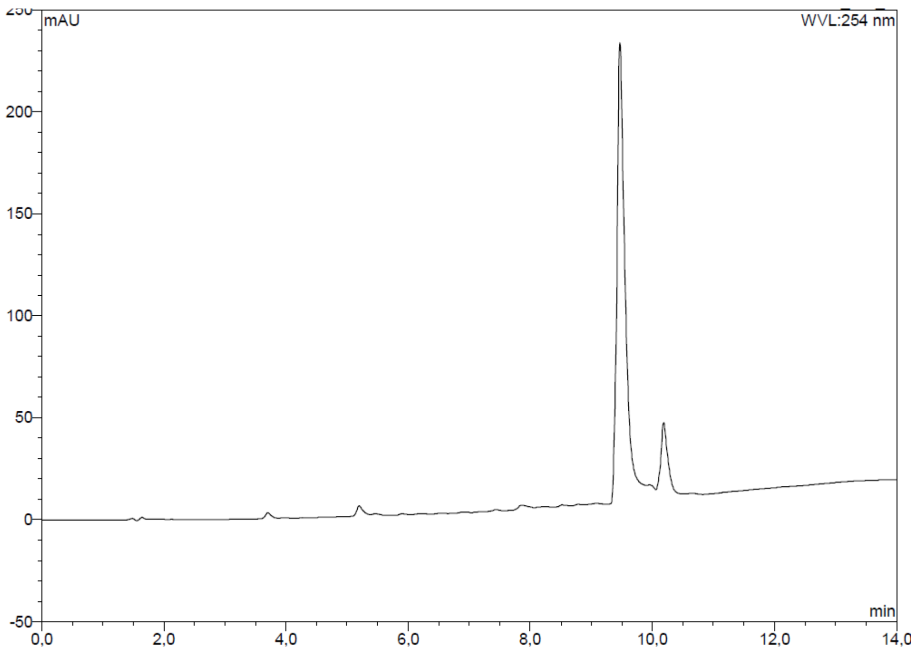

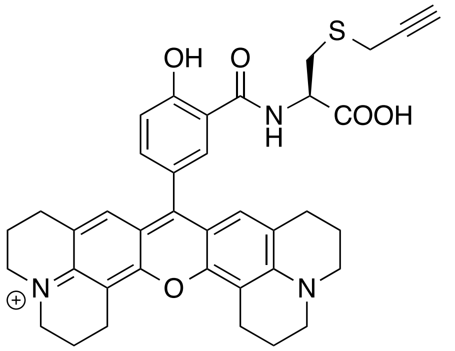

HR-CysA. 2k (0.079 g, 0.257 mmol); reaction time: 15 h for the condensation, 23 h for the oxidation. **HR-CysA** (0.060 g, 31 %) as a purple solid after flash chromatography (DCM/methanol (0.5 % TFA v/v) 98:2 to 90:10); ^{1}H NMR (CD_3OD, 300MHz) δ 7.93 (d, $J_{c,b}$ = 2.2 Hz, 1H, Hc), 7.41 (dd, $J_{b,a}$ = 8.4 Hz, $J_{b,c}$ = 2.0 Hz, 1H, Hb), 7.17 (d, $J_{a,b}$ = 8.4 Hz, 1H, Ha), 6.90 (s, 2H, 2Hd), 3.57 – 3.50 (m, 8H), 3.44 – 3.28 (m, 4H), 3.16 – 3.11 (m, 1H), 3.07 (t, *J* = 6.1 Hz, 4H), 2.74 – 2.69 (m, 4H), 2.59 (t, *J* = 2.7 Hz, 1H, $SCH_2C{\equiv}C\underline{H}$), 2.14 – 2.07 (m, 4H), 1.99 – 1.92 (m, 4H); ^{13}C NMR (CD_3OD, 75 MHz) δ 168.7, 161.2, 155.3, 153.7, 152.5, 136.0, 132.0, 127.7, 125.3, 118.8, 118.1, 114.1, 106.7, 80.3, 73.0, 51.9, 51.4, 33.8, 28.6, 21.8, 21.0, 20.9, 20.1; HRMS: *m/z* calcd for $C_{38}H_{38}N_3O_5S^+$: 648.2527 $[M]^+$, found 648.2527.

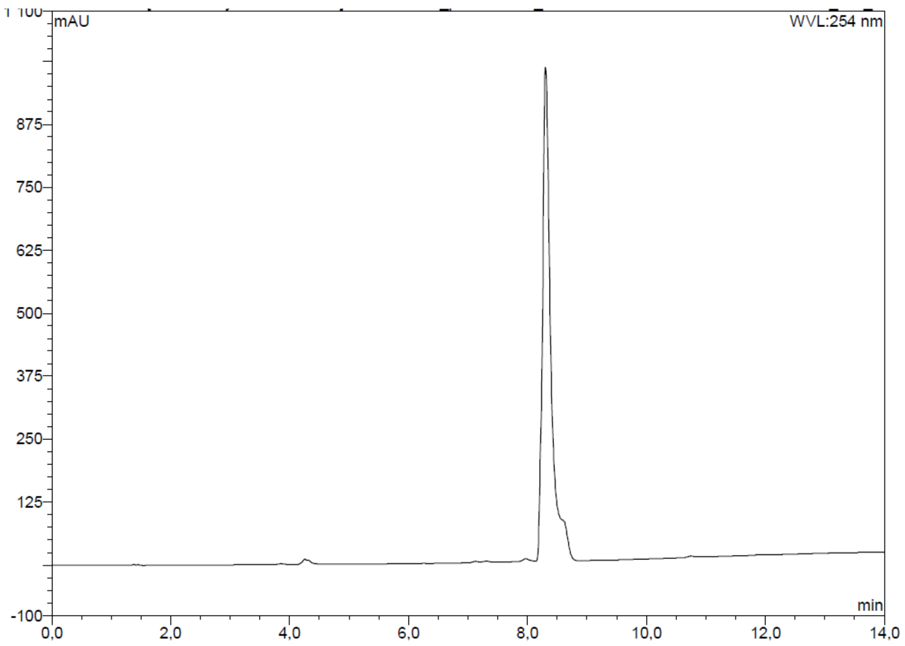

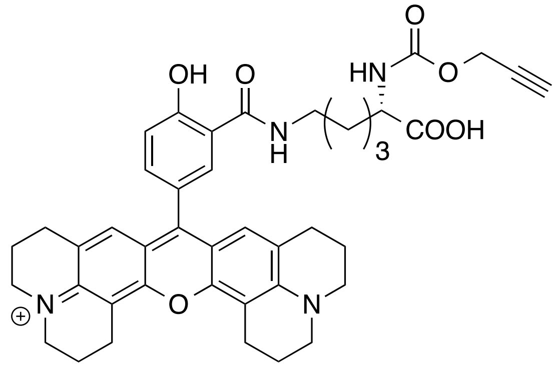

HR-LysA. 2l (0.150 g, 0.398 mmol); reaction time: 12 h for the condensation, 13 h for the oxidation. **HR-LysA** (0.120 g, 36 %) as a purple solid after flash chromatography (DCM/methanol (0.5 % TFA v/v) 100:0 to 90:10); ^{1}H NMR (CD_3OD, 300MHz) δ 7.80 (d, $J_{c,b}$ = 2.0 Hz, 1H, Hc), 7.37 (dd, $J_{b,a}$ = 8.4 Hz, $J_{b,c}$ = 2.2 Hz, 1H, Hb), 7.12 (d, $J_{a,b}$ = 8.5 Hz, 1H, Ha), 6.89 (s, 2H, 2Hd), 4.57 (d, *J* = 2.2 Hz, 2H, OC$\underline{H_2}$C≡CH), 4.10 (dd, *J* = 9.0 Hz, *J* = 4.8 Hz, 1H, $CH_2C\underline{H}(CO_2H)NH$), 3.56 – 3.49 (m, 8H), 3.37 (t, *J* = 6.9 Hz, 1H), 3.06 (t, *J* = 6.3 Hz, 1H), 2.83 (t, *J* = 2.4 Hz, 1H, $OCH_2C{\equiv}C\underline{H}$), 2.71 (t, *J* = 5.9 Hz, 4H), 2.13 – 2.05 (m, 4H), 2.00 – 1.81 (m, 5H), 1.75 – 1.55 (m, 3H), 1.50 – 1.39 (m, 2H); ^{13}C NMR (CD_3OD, 75 MHz) δ 169.7, 157.6, 155.5, 153.6, 152.5, 135.7, 130.8, 127.7 (2C), 125.3, 124.6, 119.0 (2C), 117.8, 114.1, 106.7, 79.4, 75.9, 53.2, 51.9, 51.4, 40.4, 32.9, 29.9, 28.6, 24.3, 21.8, 21.0, 20.9; HRMS: *m/z* calcd for $C_{42}H_{45}N_4O_7^+$: 717.3283 $[M]^+$, found 717.3284.

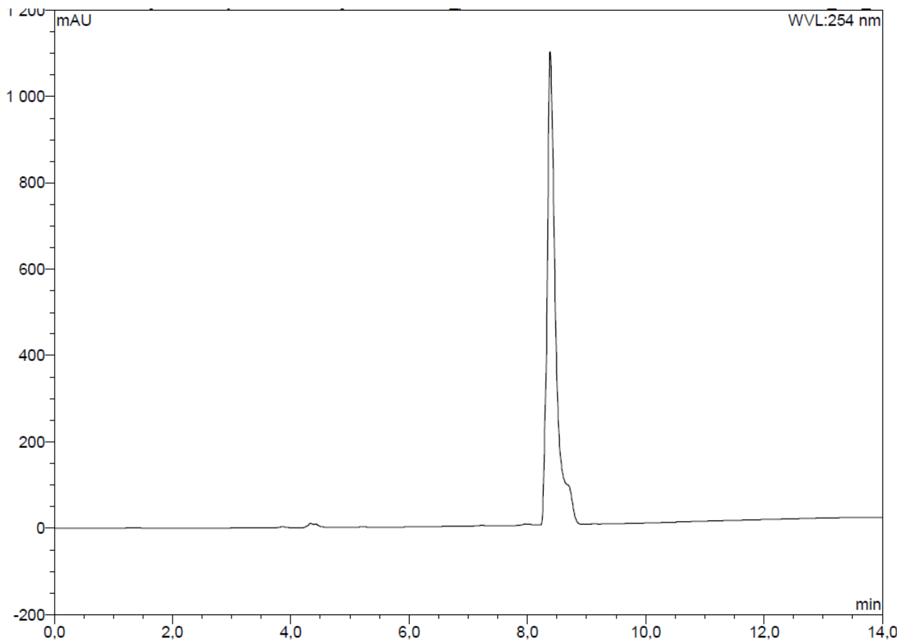

Preparation of the dextran conjugates

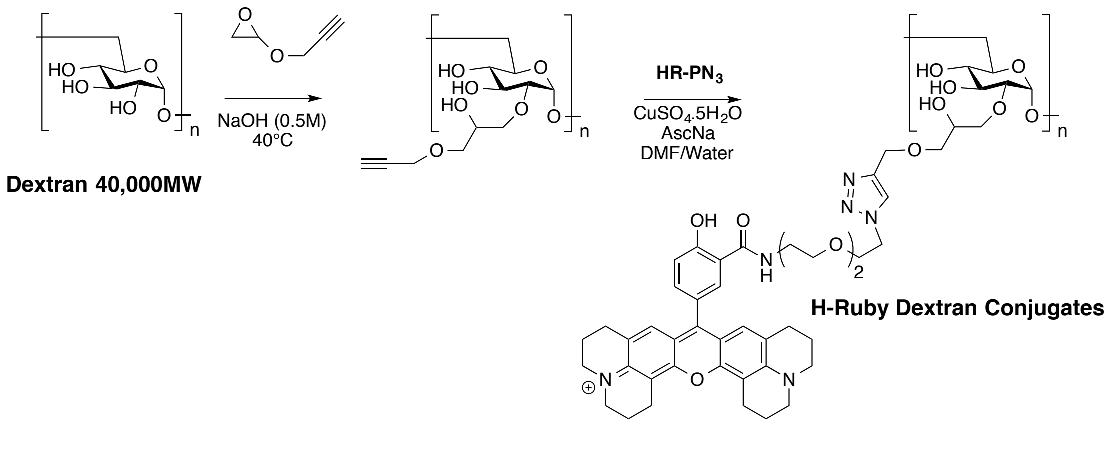

Scheme S6. Synthesis of **HR-PN$_3$ dextran conjugate**

Alkyne dextran. To a solution of dextran 40 kDa (0.209 g, 1.29 mmol) in 0.5 M aq. sodium hydroxide (2 mL) was added glycidyl propargyl ether (0.460 mL, 3.87 mmol). The mixture was stirred at 40°C for 20 h then added dropwise in isopropyl alcohol (20 mL). The mixture was cooled at -20°C for 10 min then centrifugated and the supernatant was removed. This operation was repeated once then the crude solid was purified over a G-25 exclusion size column (elution with water) to give the alkyne dextran (0.228 g, Degree of Substitution = 80 %, Final MW = 62 kDa) as an off-white solid after lyophilization. The degree of substitution (DS) was determined by proton NMR.

Azido dextran. To a solution of dextran 40 kDa (0.190 g, 1.17 mmol) in 0.5 M aq. sodium hydroxide (2 mL) was added 2-{2-[2-(2-Azidoethoxy)ethoxy]ethoxymethyl}oxirane[6] (0.819 g, 3.51 mmol). The mixture was stirred at 40°C for 18 h then filtered over a G-25 exclusion size column (elution with water) to give the azido dextran (0.212 g, Degree of Substitution = 30 %, Final MW = 57 kDa) as an off-white solid after lyophilization. The degree of substitution (DS) was determined by proton NMR.

H-Ruby dextran conjugate. To a solution of alkyne dextran **13** (30 mg, 0.5 μmol) and HR-PN_3 (2 mg, 2.5 μmol) in *N*,*N*-dimethylformamide (0.4 mL) was added $CuSO_4.5H_2O$ (2.5 mg, 10 μmol) and sodium ascorbate (2.5 mg, 12.5 μmol) in water (0.1 mL). The mixture was stirred in the dark at room temperature for 20 h then concentrated. The crude residue was dissolved in aq. 0.1 M EDTA (0.500 mL) then purified over G-25 size exclusion column (elution with water) to give **HR-PN_3 dextran conjugate** (24 mg, Molar ratio ~ 1.3 mol dye /mol dextran) as a purple solid after lyophilization.

Alexa-647 dextran conjugate. To a solution of azido dextran (5 mg, 0.09 μmol) and Alexa Fluor 647 alkyne (0.4 mg, 0.5 μmlo) in a 3:1 mixture of water/DMF (0.4 mL) was added $CuSO_4.5H_2O$ (0.5 mg, 2 μmol) and sodium ascorbate (0.5 mg, 2.5 μmol) in water (0.1 mL). The mixture was stirred in the dark at room temperature for 24 h then concentrated. The crude residue was dissolved in aq. 0.1 M EDTA (0.5 mL) then purified over G-25 size exclusion column (elution with water) to give the Alexa-647 dextran conjugate (3.7 mg) as a blue solid after lyophilization.

General Calculations and Terms

The term dynamic range has been used throughout and refers to the maximal value of equation 1 where I is the observed fluorescence intensity and $I_{min.}$ is the minimum fluorescence intensity observed for that probe.

$$Dynamic\ Range = \frac{(I - I_{\min.})}{I_{\min.}} \qquad \text{(Equation 1)}$$

pKa measurement. pK_a values were calculated by plotting the fluorescence enhancement ($[I-I_{min}]/I_{min}$) at $\lambda_{em.,max}$ versus the pH. The data was fit to the Hill Equation (equation 2) and the pK_a taken as $x_{1/2}$ as per the Hill equation.

$$base + \frac{(\max - base)}{1 + \left(\frac{x_{1/2}}{x}\right)^{rate}} \qquad \text{(Equation 2)}$$

[6] D. C. Knapp, J. D'Onofrio, J. W. Engels, *Bioconjugate Chem.* **2010**, *21*, 1043-1055.

base = y value at minimum x-value
max = y value at maximum x-value
rate = rise rate of the curve
$x_{1/2}$ = x value at which y = (base + max)/2.

Quantum Yield Calculations. The fluorescence quantum yields (ϕ) were calculated from the gradient of integrated fluorescence intensity (545 to 700 nm) versus optical density. Rhodamine 101 (ϕ = 1.0 in absolute ethanol) was used as a reference standard (ref) with the excitation wavelength at 535 nm. Equation 3 was used to determine the ϕ values.

$$\Phi = \Phi_{ref} \frac{grad.}{grad._{ref}} \cdot \frac{\eta^2}{\eta_{ref}^2} \qquad \text{(Equation 3)}$$

ϕ = quantum yield
η = refractive index of the solvent system (η (H_2O) = 1.33, η_{ref} (EtOH) = 1.36)
grad. = gradient of the curve
ϕ_{ON} (pH = 4) and ϕ_{OFF} (pH = 10) was calculated for each pH probe in (MOPS 30 mM, KCl 100 mM, pH 7.2). The plot for **HR-PiAC** is given as an example below.

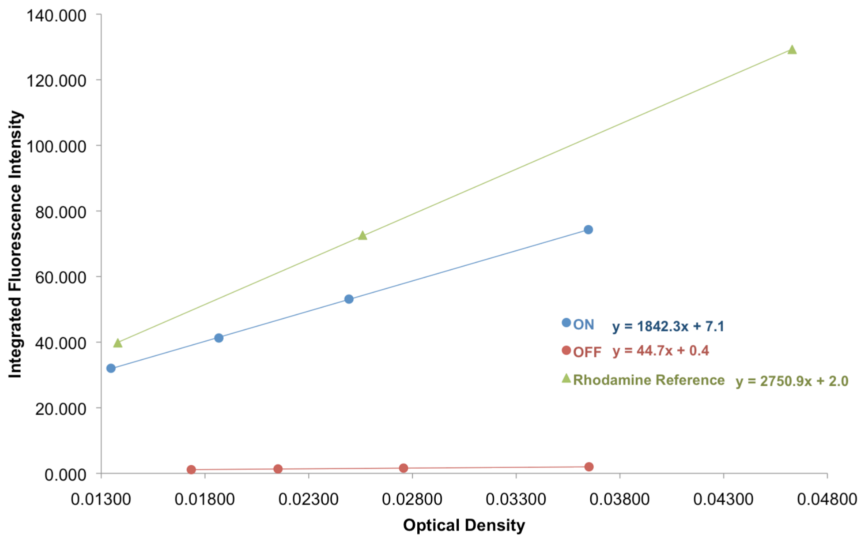

Figure S1. Plot of integrated fluorescence intensity versus optical density for the Rhodamine 101 standard and pH probe **HR-PiAC** ON (pH 4) and OFF (pH 10).

Molar Extinction Coefficient Calculations. Extinction coefficients (ε) were calculated using the Beer Lambert law (equation 4) plotting optical dentisy/absorption versus concentration for the pH probes. The gradient of the plot is equal to ε.

A = εcl (Equation 4)

A = optical density/absorption
ε = extinction coefficient = gradient of the plot
c = concentration
l = optical path length (1cm)

The plot for **HR-PiAC** is given as an example below (figure SI-2).

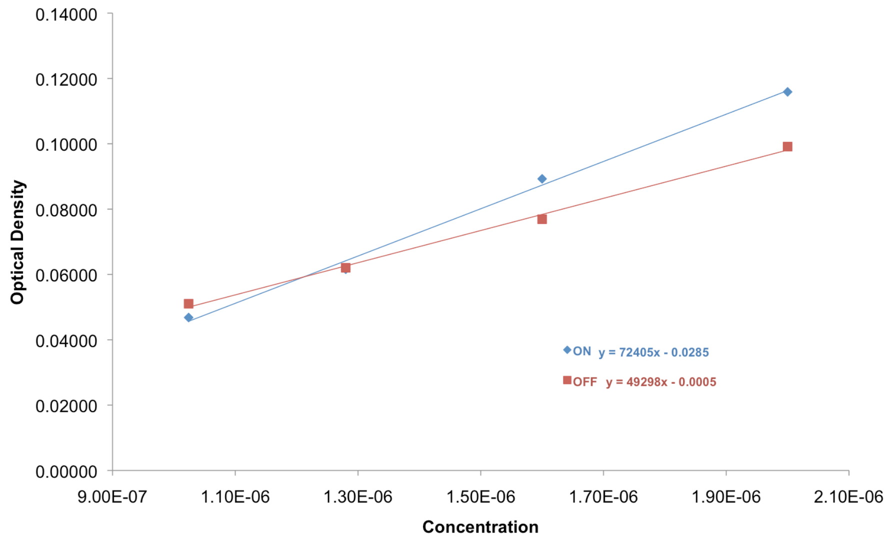

Figure S2. Plot of optical density versus concentration for pH probe **HR-PiAC** ON (pH 4) and OFF (pH 10).

Effect of HR-Br concentration on its apparent pKa and fluorescence enhancement

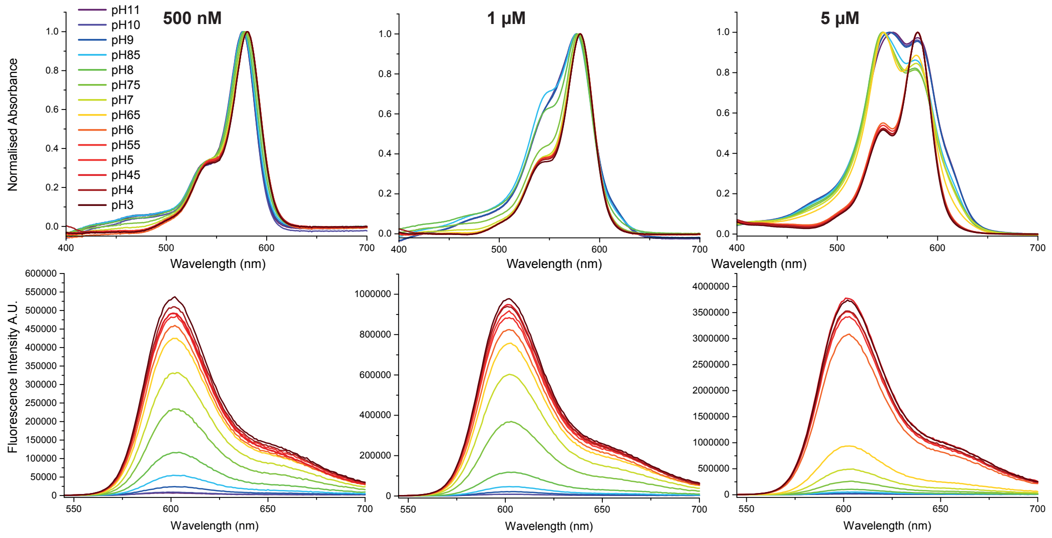

Figure S3. Absorbance and emission spectra of HR-Br at 500 nM (left), 1 µM (middle) and 5 µM (right) and at different pH value depicting the depency of *H*-aggregates formation on the concentration of the sensor.

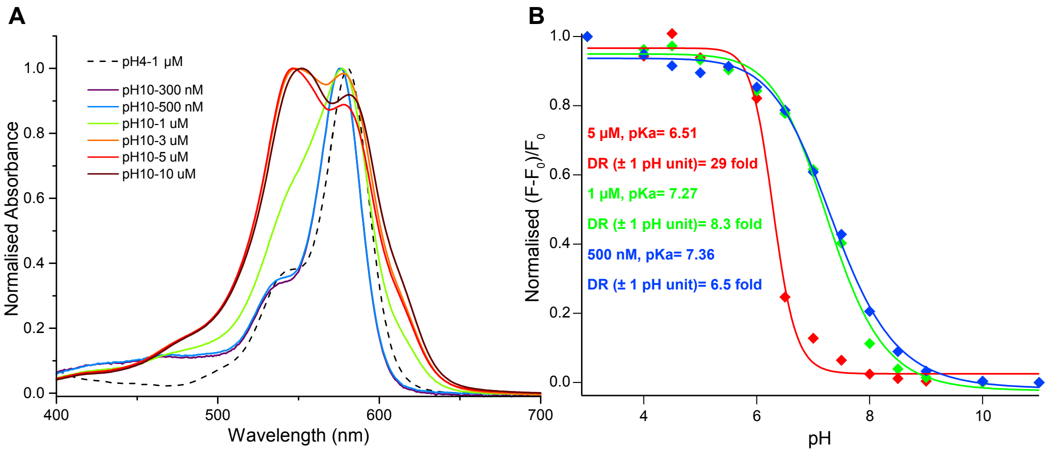

Figure S4. (A) Normalised absorption spectra of HR-Br at pH 10 and at different concentrations, spectra at pH 4 (1 μM) was added as a de-aggregated model (dashed line). This experiment shows that HR-Br has tendency to form aggregate at pH 10 at concentration up to 500 nM. (B) Normalised titration curves of HR-Br at different concentration (500 nM, 1 μM and 5 μM). This experiment shows that the concentration of the sensors influence the apparent pKa value as well as the dynamic range (DR).

LDH-Cytotoxicity assay

CHO-K1 cells (20,000 per well) were seeded in 96 multiple well plates 24 hours before the experiment. Cells (40,000 per well) were incubated with the fluorescent compounds in 100 μL DMEM for 2 hours at 37°C. The incubation milieu was then replaced by 100 μL fresh DMEM containing 10% of cell-counting solution (Dojindo Laboratories) and further incubated for 2hours at 37°C. The absorbance measured at 450 nm (reference at 620 nm) was directly related to the number of viable cells. Experiments were done in triplicate and repeated twice.

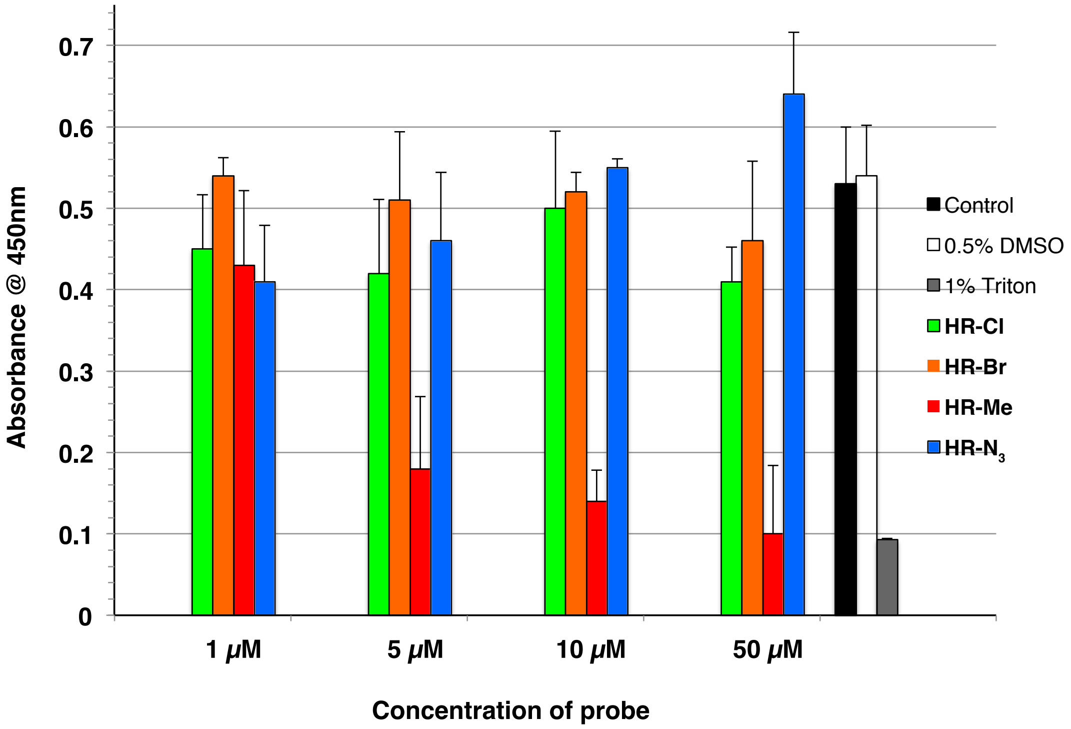

Figure S5. LDH results for **HR-N3, HR-Cl, HR-Br** and **HR-Me** indicating cytotoxicity. The probes were incubated with Chinese hamster ovary (CHO-K1) cells for 2 hours at 37˚C.

Colocalisation experiments with H-Rubies and mitotracker green

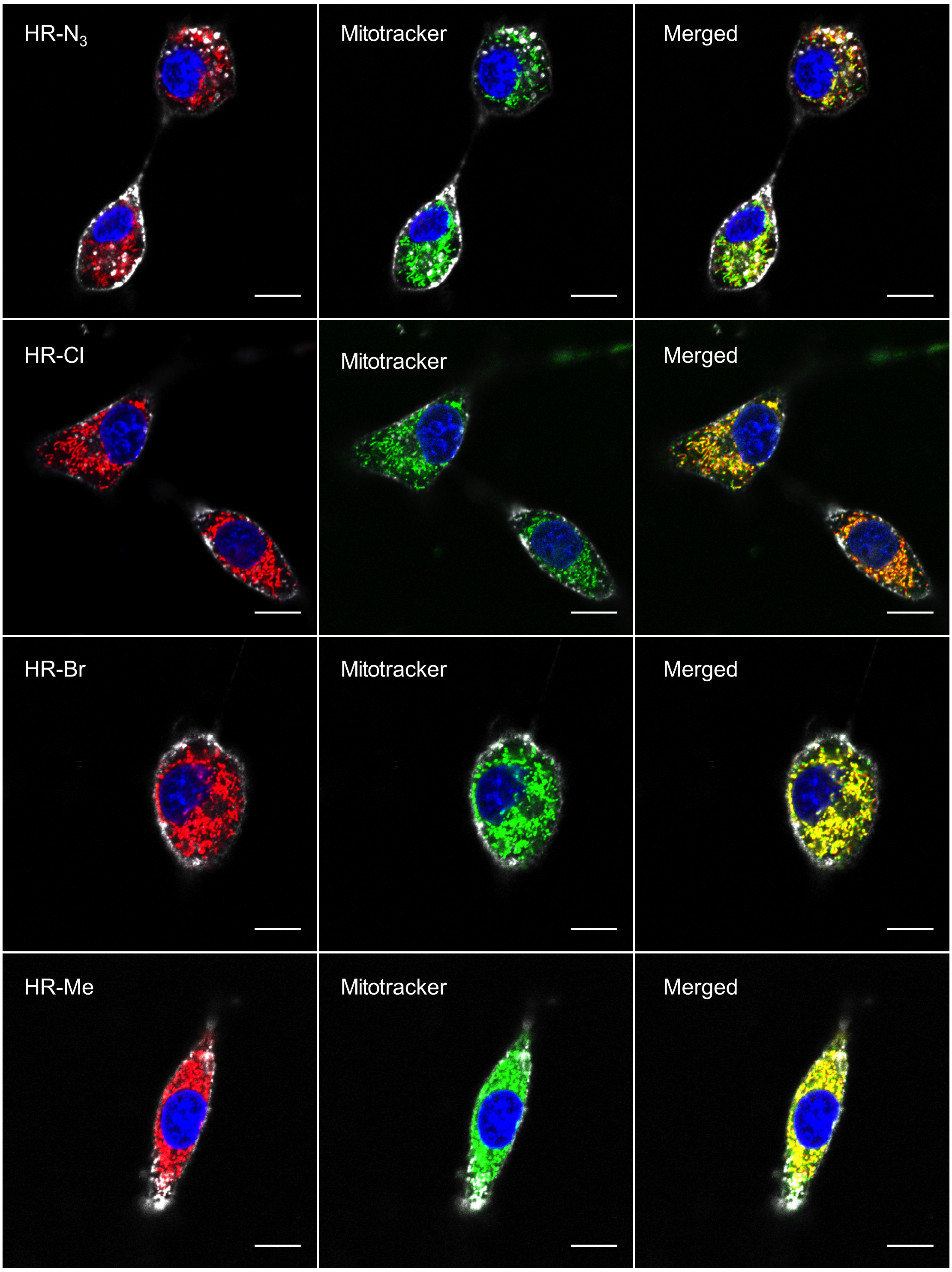

Figure S6. Laser scanning confocal microscopy images of F98 cells incubated with 1 μM of **HR-N**$_3$, **HR-Cl**, **HR-Br**, and **HR-Me**. The H-rubies were visualized in red, the Mitotracker green in green, the nuclei were stained with Hoechst (blue) and the plasma membrane with Alexa-647-conjugated concanavalin A (grey). Each image contains the nucleus and membrane staining. Left images show the H-Ruby, middle images show the Mitotracker and

right images show the merged channels. Scale bar is 10 µm. Note that the red color intensities have been adjusted with imageJ in order to correctly localize the H-Rubies.

Effect of intracellular acidification and alkalinization on the H-Rubies' fluorescence enhancement

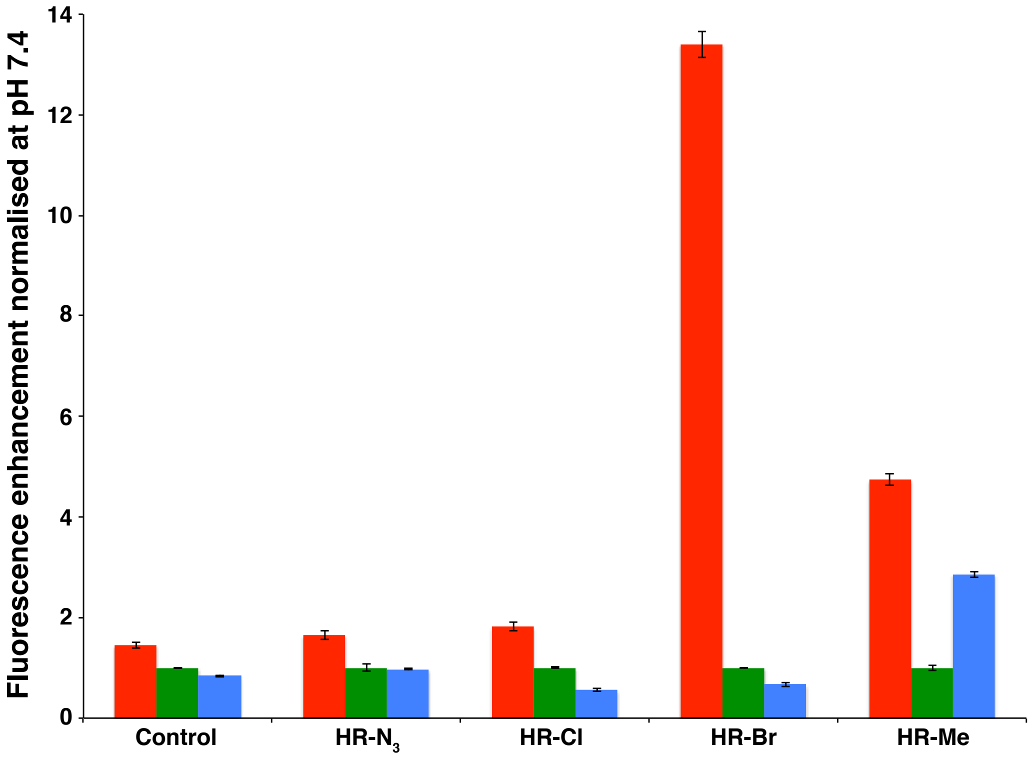

Figure S7. Fluorescence enhancement (normalized to fluorescence at pH 7.4) of the 4 H-Rubies assessed by flow cytometry. The control is cells without H-Ruby.

1X Concentration (acid, mM/ base, mM)[1]	0,5 log (acid/base)	Pseudo-null pH[2]
1/25,1	-1,4	8,8
1/1	0	7,4
25,1/1	1,4	6

[1]Weak acid, acetic acid; weak base, ammonium hydroxide

[2]Pseudo-null pH values are calculated from the following equation: pHi = pHe – 0,5 log([Acid]/[Base])

Table S1. Composition of pseudo-null solutions used for modification of intracellular pH
