# Supplementary material for: H-Rubies, a new family of red emitting fluorescent pH sensors for living cells
Source: Chem Sci. 2015 Jul 14;6(10):5928–37. doi: 10.1039/c5sc01113b (PMC5950754; doi:10.1039/c5sc01113b)
Supplement: Supplementary file 2 [file SC-006-C5SC01113B-s002.pdf]

Electronic Supplementary Material (ESI) for Chemical Science.
This journal is © The Royal Society of Chemistry 2015

H-Rubies, a New Family of Red Emitting Fluorescent pH sensors for Living Cells

Guillaume Despras[+], Alsu Zamaleeva[+], Lucie Dardevet, Céline Tisseyre, Joao Gamelas Magalhaes, Charlotte Garner, Michel De Waard, Sebastian Amigorena, Anne Feltz, Jean-Maurice Mallet, Mayeul Collot*

Supplementary information

Spectral and physico-chemical properties, absorption and emission spectra at different pH, pKa titration curves and metal sensitivities of H-Rubies

Imidazole

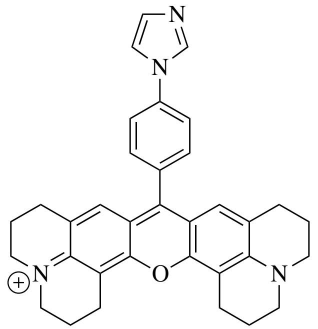

State	λ_{abs} (nm)	λ_{em} (nm)	pK_a	ε (M^{-1} cm^{-1})	Φ	Φ_{ON}/Φ_{OFF}	Dynamic Intensity
ON[a]	583	609	N/A[c]	63403	0.40	N/A[c]	N/A[c]
OFF[b]	584	608		N/A[c]	N/A[c]		

[a] Protonated form: pH 4

[b] Deprotonated form: pH 10

[c] Not found to be a pH probe

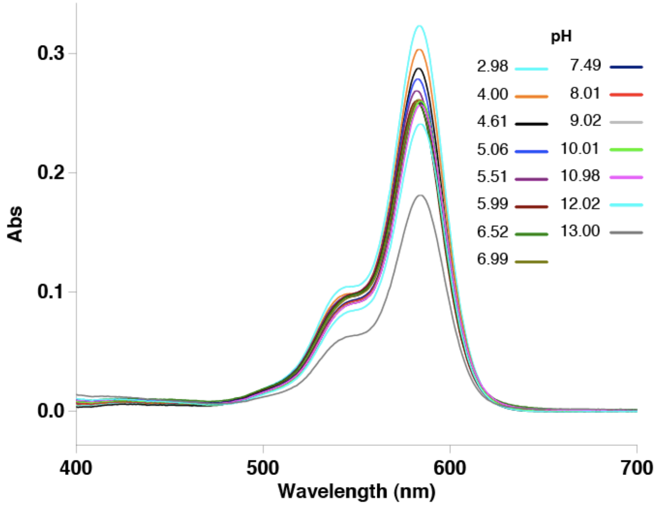

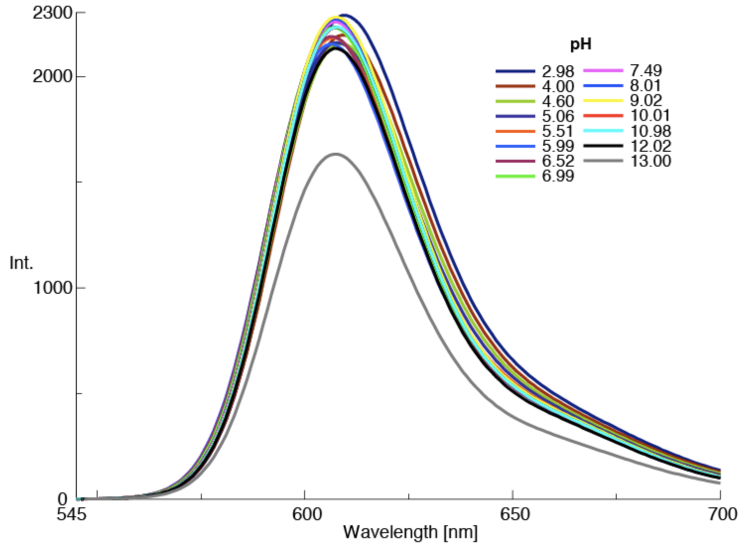

Left: Dependence of absorption on pH in a MOPS buffered aqueous solution. Right: Dependence of fluorescence intensity on pH in a MOPS buffered aqueous solution. Excitation at 535 nm.

HR-*m*OH

State	λ_{abs} (nm)	λ_{em} (nm)	pK$_a$	ε (M^{-1} cm^{-1})	Φ	Φ_{ON}/Φ_{OFF}	Dynamic Intensity
ON[a]	578	602	9.33 ± 0.13	50279	0.30	3	132
OFF[b]	577	603		43040	0.11		

[a] Protonated form: pH 4

[b] Deprotonated form: pH 10

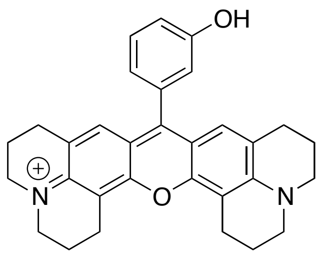

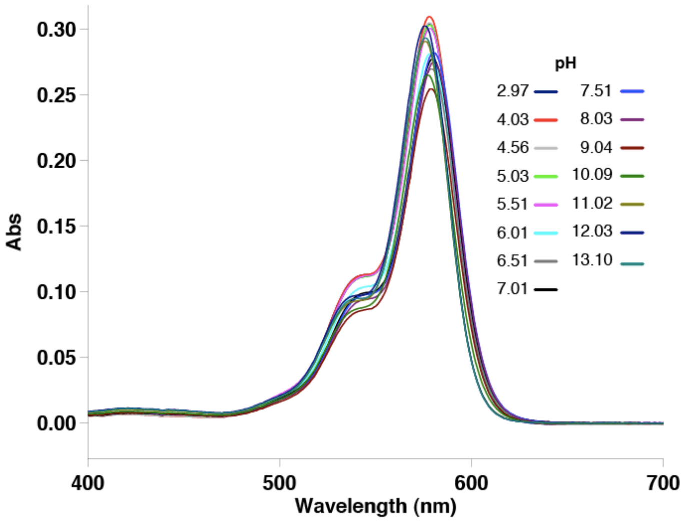

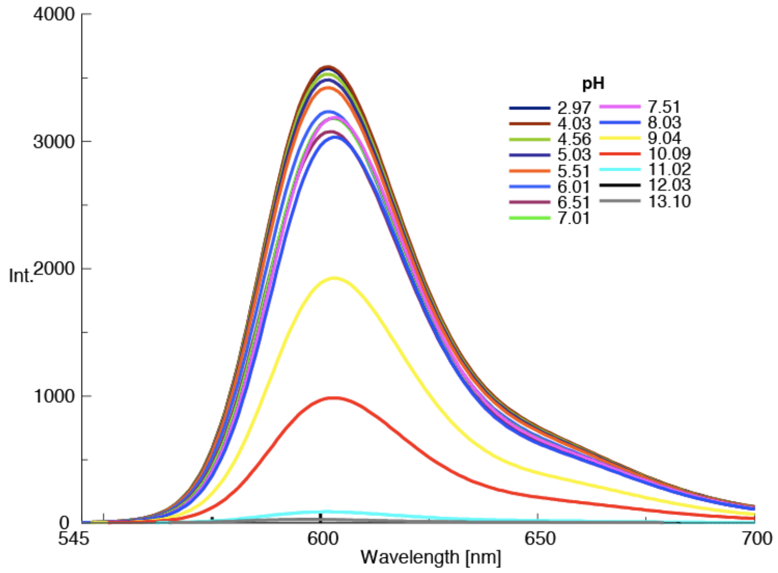

Left: Dependence of absorption on pH in a MOPS buffered aqueous solution. Right: Dependence of fluorescence intensity on pH in a MOPS buffered aqueous solution. Excitation at 535 nm.

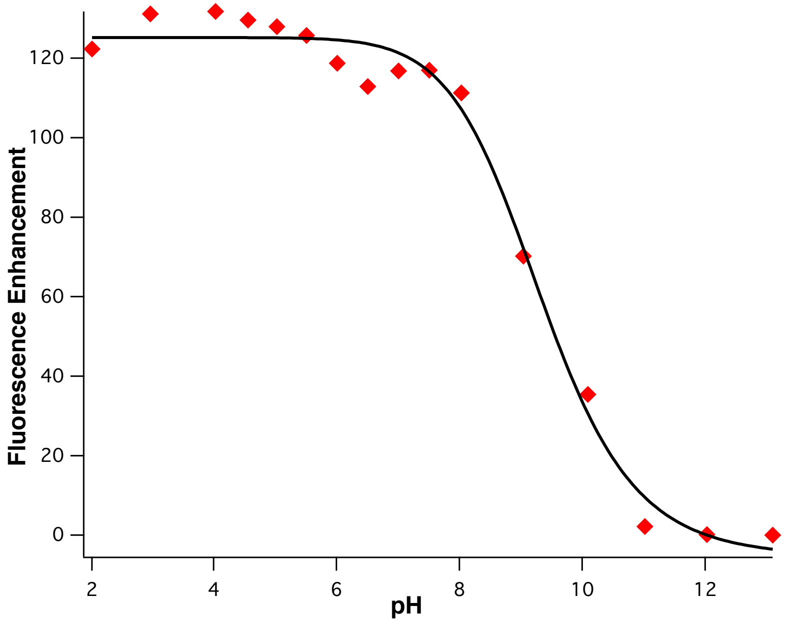

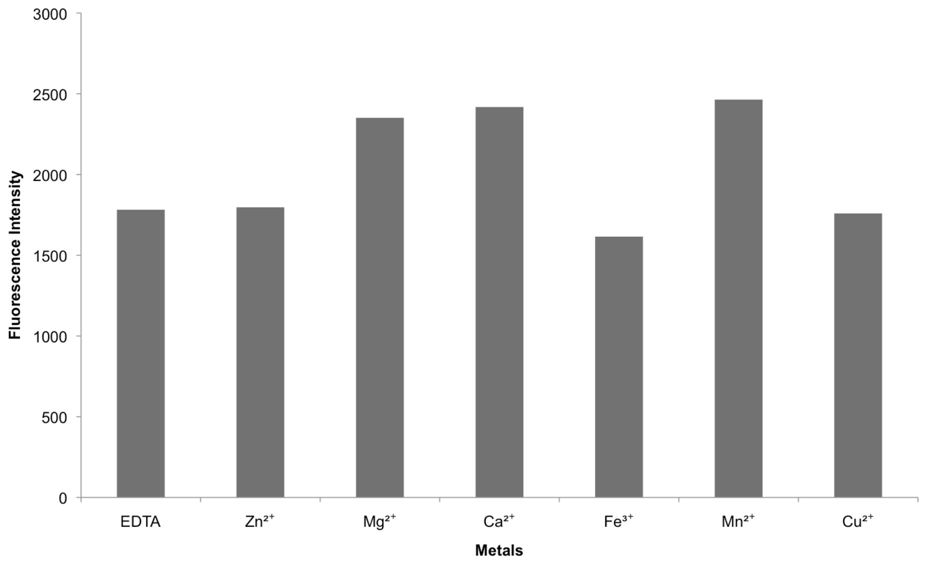

Left: Dependence of fluorescence enhancement on pH. Curve fitting was based on a modified Hill equation from which pK_a values were calculated. Right: Fluorescence intensities of the probe (5 μM) to a range of metal ions in a MOPS buffer (MOPS 30 mM, KCl 100 mM, pH 7.2). The concentration of cations is 10^{-3} M.

HR-Cl

State	λ_{abs} (nm)	λ_{em} (nm)	pK$_a$	ε (M^{-1} cm^{-1})	Φ	Φ_{ON}/Φ_{OFF}	Dynamic Intensity
ON[a]	579	601	6.17 ± 0.10	48432	0.47	-	753
OFF[b]	544	599		33146	N/A[c]		

[a] Protonated form: pH 4

[b] Deprotonated form: pH 10

[c] Non-fluorescent

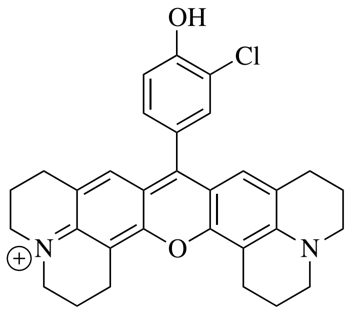

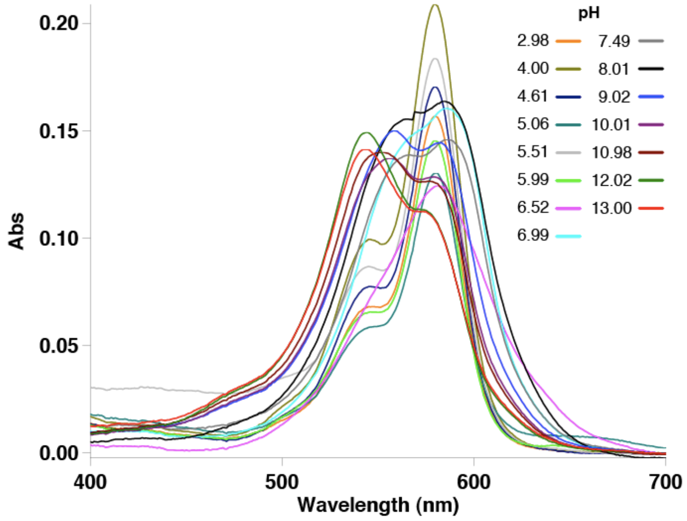

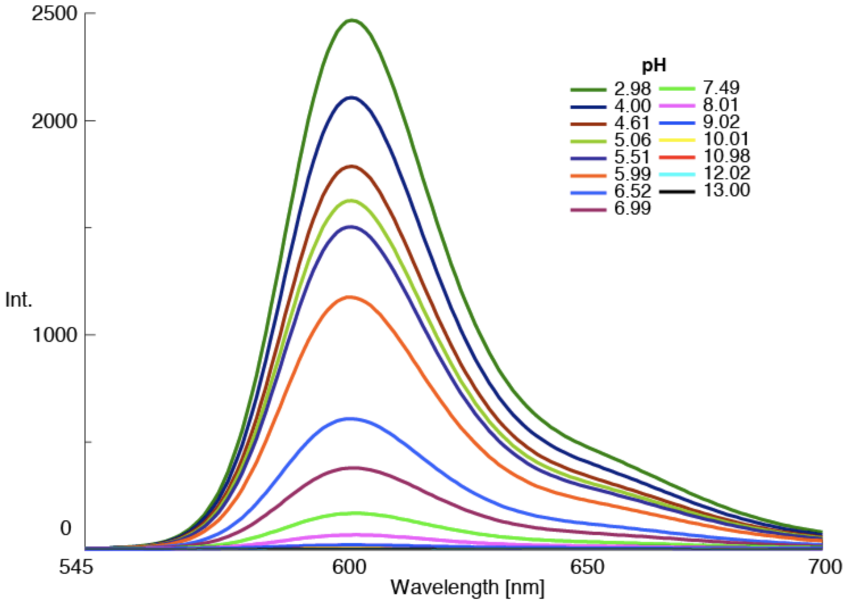

Left: Dependence of absorption on pH in a MOPS buffered aqueous solution. Right: Dependence of fluorescence intensity on pH in a MOPS buffered aqueous solution. Excitation at 535 nm.

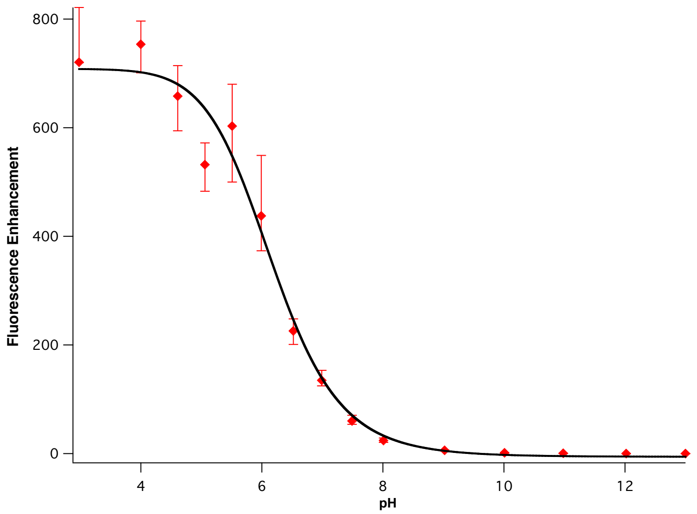

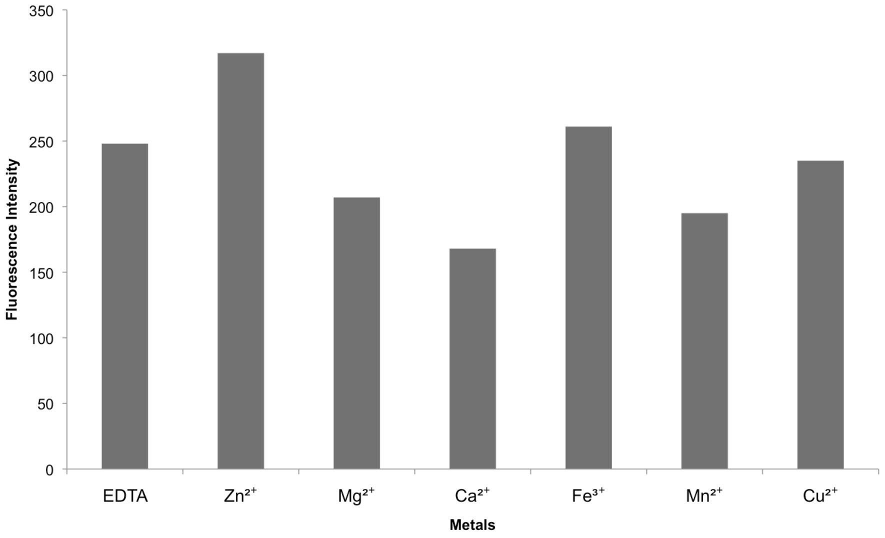

Left: Dependence of fluorescence enhancement on pH. Curve fitting was based on a modified Hill equation from which pK_a values were calculated. Right: Fluorescence intensities of the probe (5 μM) to a range of metal ions in a MOPS buffer (MOPS 30 mM, KCl 100 mM, pH 7.2). The concentration of cations is 10^{-3} M.

HR-Br

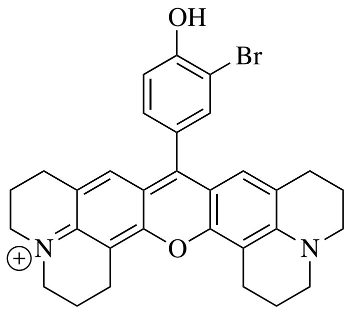

State	λ_{abs} (nm)	λ_{em} (nm)	pK$_a$	ε (M^{-1} cm^{-1})	Φ	$Φ_{ON}/Φ_{OFF}$	Dynamic Intensity
ON[a]	581	601	6.51 ± 0.10	24423	0.55	-	384
OFF[b]	544	600		25553	N/A[c]		

[a] Protonated form: pH 4
[b] Deprotonated form: pH 10
[c] Non-fluorescent

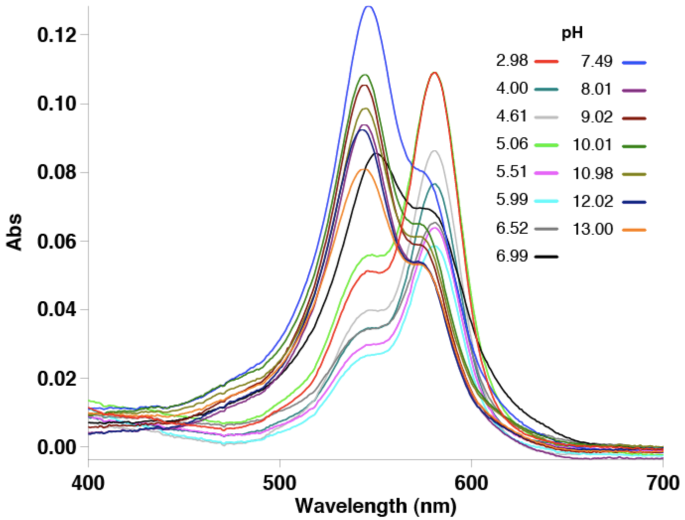

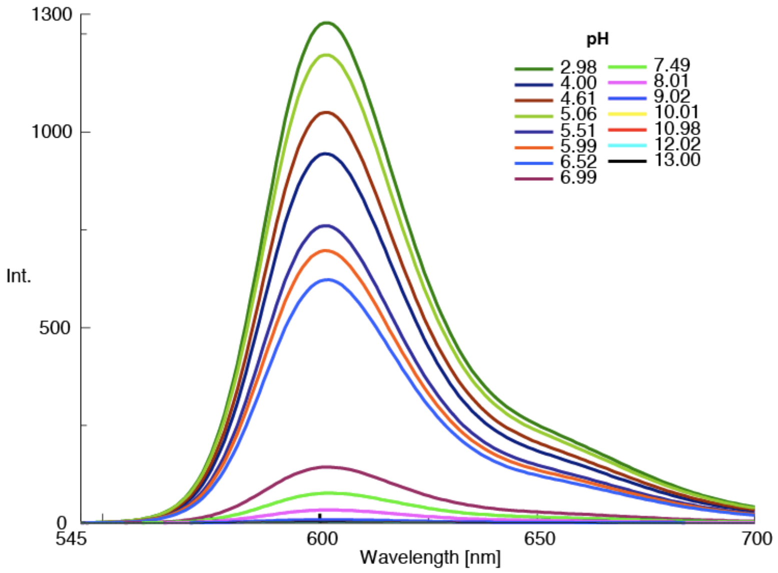

Left: Dependence of absorption on pH in a MOPS buffered aqueous solution. Right: Dependence of fluorescence intensity on pH in a MOPS buffered aqueous solution. Excitation at 535 nm.

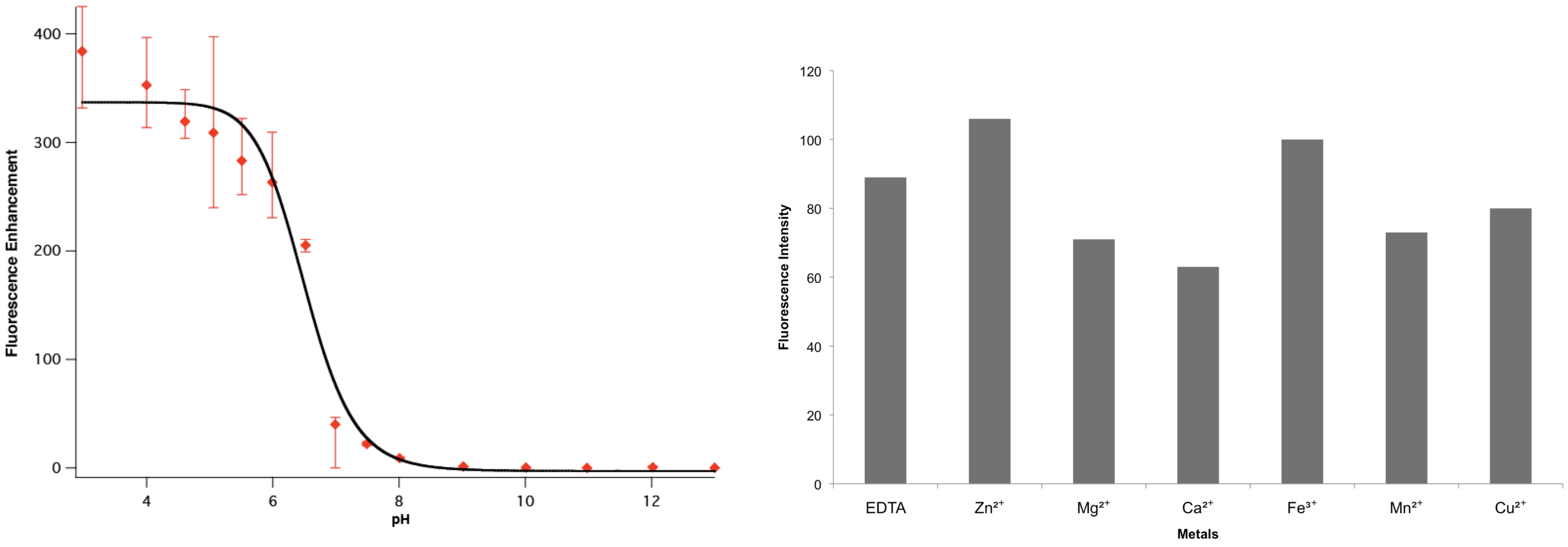

Left: Dependence of fluorescence enhancement on pH. Curve fitting was based on a modified Hill equation from which pK_a values were calculated. Right: Fluorescence intensities of the probe (5 μM) to a range of metal ions in a MOPS buffer (MOPS 30 mM, KCl 100 mM, pH 7.2). The concentration of cations is 10^{-3} M.

HR-Me

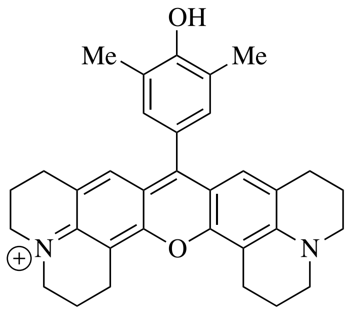

State	λ_{abs} (nm)	λ_{em} (nm)	pK$_a$	ε (M^{-1} cm^{-1})	Φ	Φ_{ON}/Φ_{OFF}	Dynamic Intensity
ON[a]	574	597	8.75 ± 0.16	52000	0.09	-	955
OFF[b]	N/A[c]	598		N/A[c]	N/A[d]		

[a] Protonated form: pH 4

[b] Deprotonated form: pH 10

[c] Poor solubility

[d] Non-fluorescent

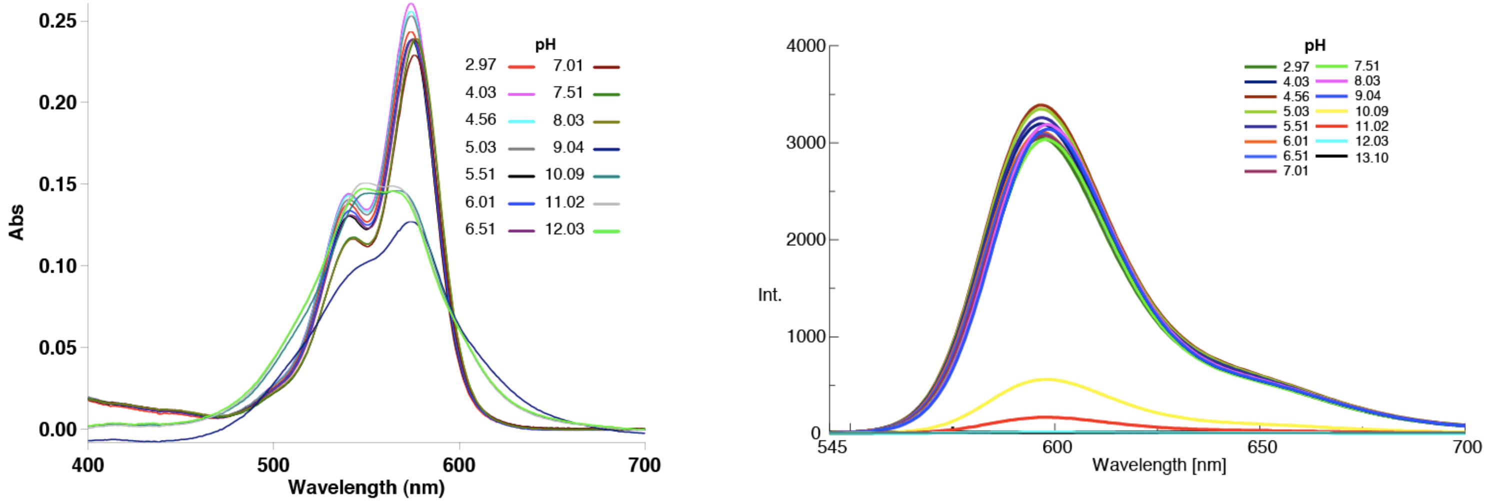

Left: Dependence of absorption on pH in a MOPS buffered aqueous solution. Right: Dependence of fluorescence intensity on pH in a MOPS buffered aqueous solution. Excitation at 535 nm.

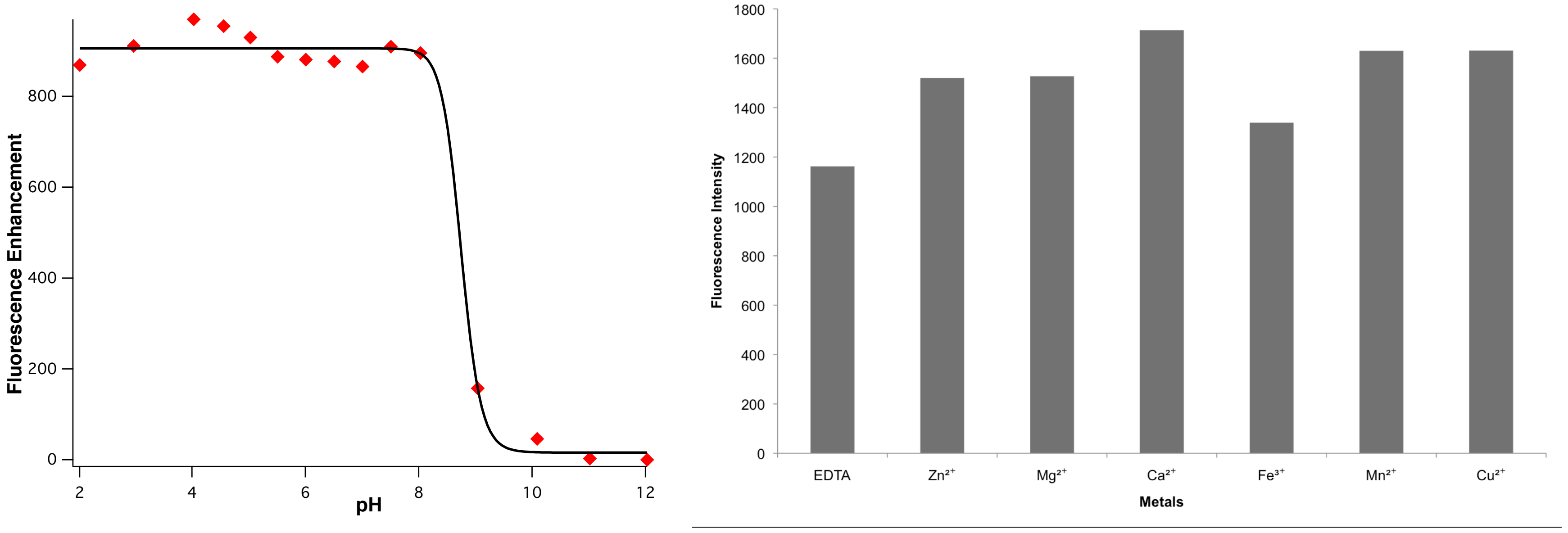

Left: Dependence of fluorescence enhancement on pH. Curve fitting was based on a modified Hill equation from which pK_a values were calculated. Right: Fluorescence intensities of the probe (5 μM) to a range of metal ions in a MOPS buffer (MOPS 30 mM, KCl 100 mM, pH 7.2). The concentration of cations is 10^{-3} M.

HR-OMe

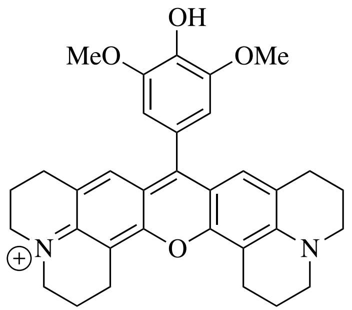

State	λ_{abs} (nm)	λ_{em} (nm)	pK_a	ε (M^{-1} cm^{-1})	Φ	Φ_{ON}/Φ_{OFF}	Dynamic Intensity
ON[a]	577	598	N/A[c]	51955	0.22	8	302
OFF[b]	576	600		54578	0.03		

[a] Protonated form: pH 4
[b] Deprotonated form: pH 10
[c] pK_a too high

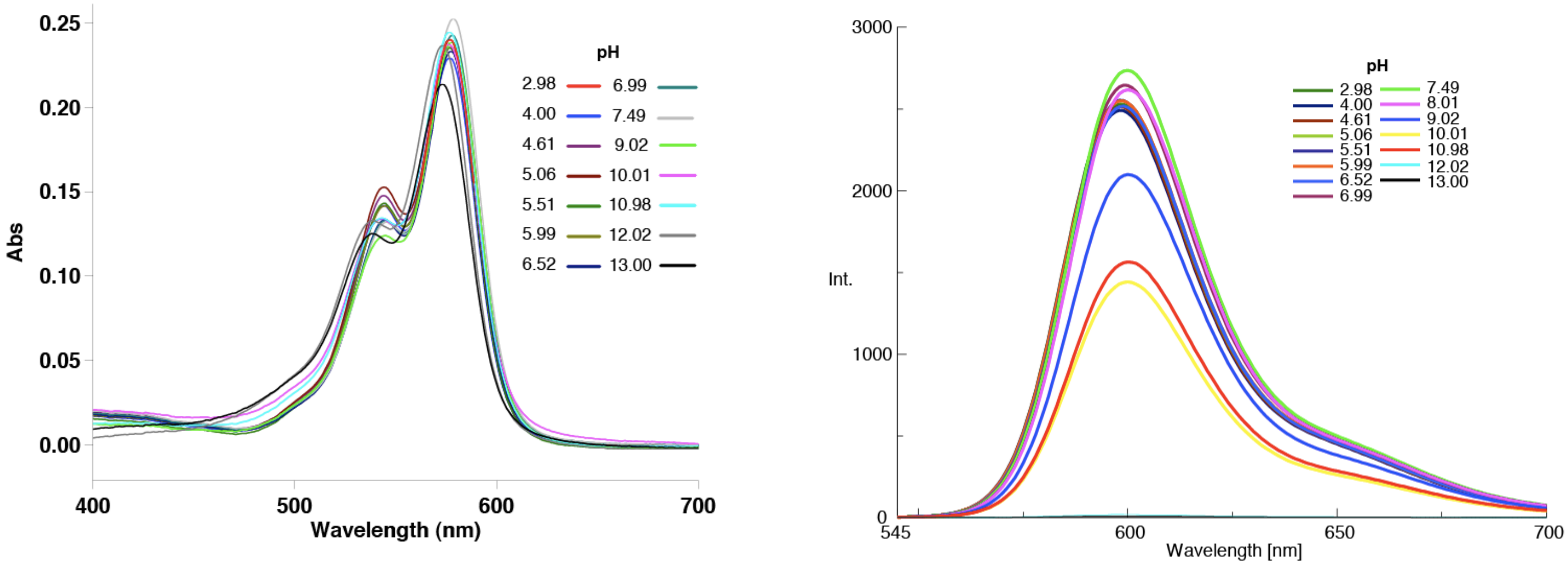

Left: Dependence of absorption on pH in a MOPS buffered aqueous solution. Right: Dependence of fluorescence intensity on pH in a MOPS buffered aqueous solution. Excitation at 535 nm.

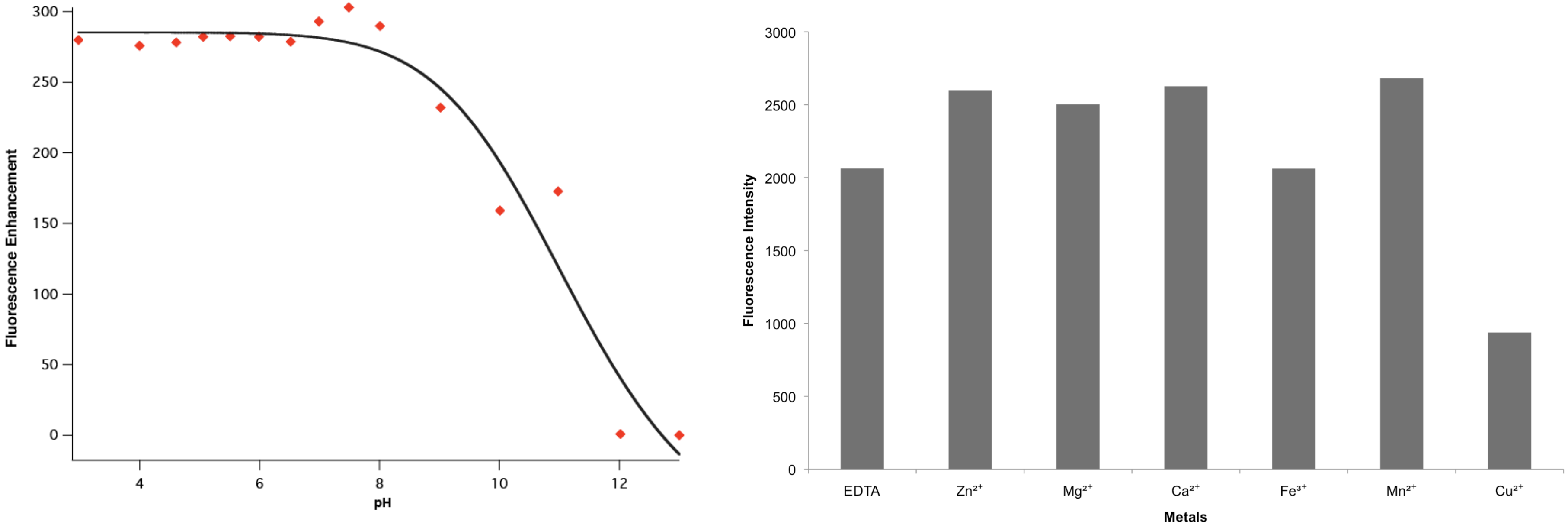

Left: Dependence of fluorescence enhancement on pH. Curve fitting was based on a modified Hill equation from which pK_a values were calculated. Right: Fluorescence intensities of the probe (5 μM) to a range of metal ions in a MOPS buffer (MOPS 30 mM, KCl 100 mM, pH 7.2). The concentration of cations is 10^{-3} M.

HR-*p*OH

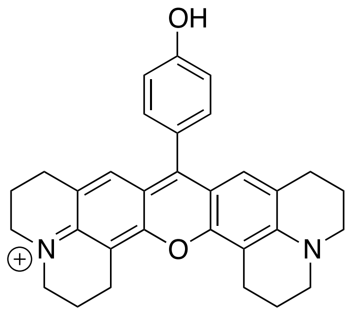

State	λ_{abs} (nm)	λ_{em} (nm)	pK$_a$	ε (M^{-1} cm^{-1})	Φ	Φ_{ON}/Φ_{OFF}	Dynamic Intensity
ON[a]	576	597	8.97 ± 0.05	61528	0.55	9	34
OFF[b]	579	598		34458	0.06		

[a] Protonated form: pH 4

[b] Deprotonated form: pH 10

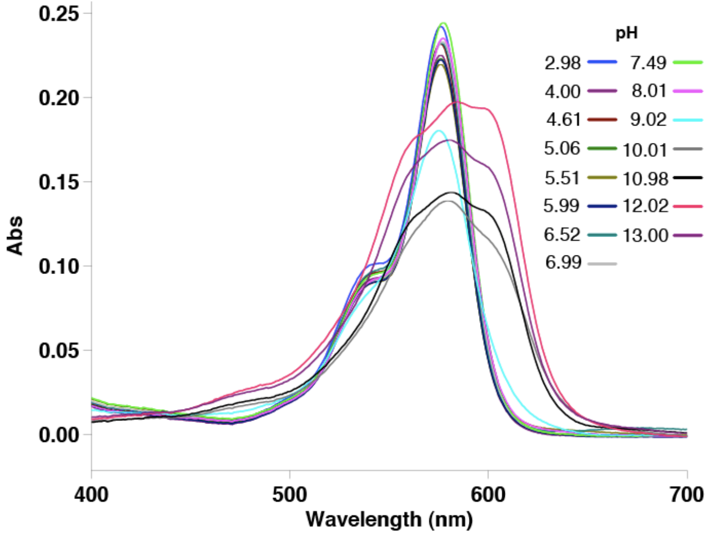

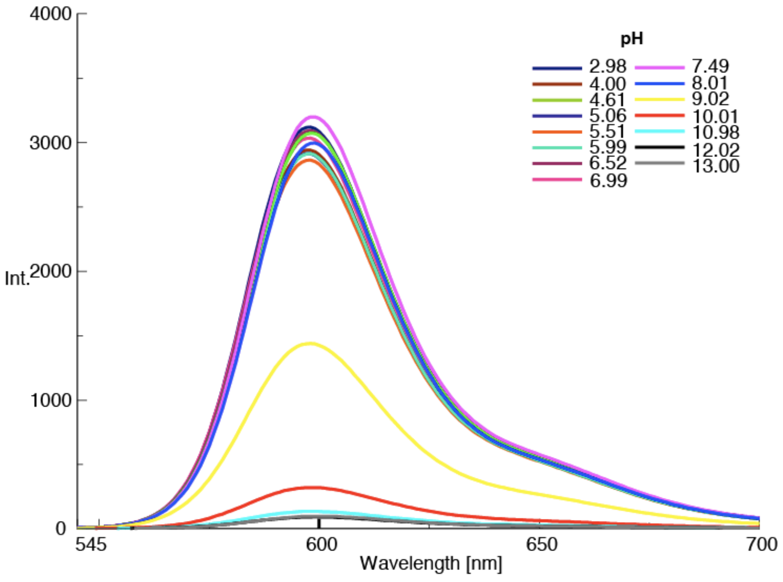

Left: Dependence of absorption on pH in a MOPS buffered aqueous solution. Right: Dependence of fluorescence intensity on pH in a MOPS buffered aqueous solution. Excitation at 535 nm.

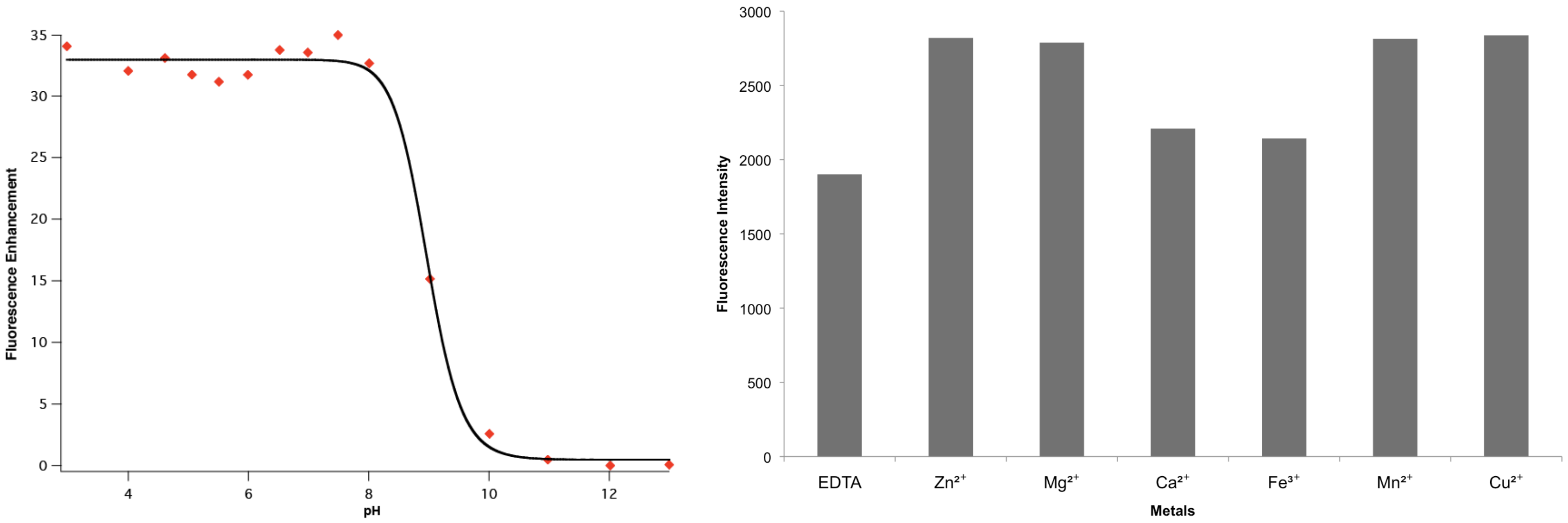

Left: Dependence of fluorescence enhancement on pH. Curve fitting was based on a modified Hill equation from which pK_a values were calculated. Right: Fluorescence intensities of the probe (5 μM) to a range of metal ions in a MOPS buffer (MOPS 30 mM, KCl 100 mM, pH 7.2). The concentration of cations is 10^{-3} M.

HR-*o*OH

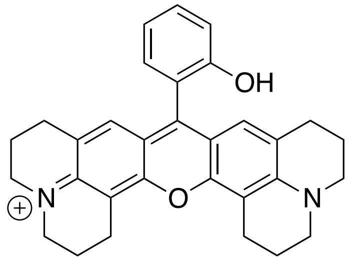

State	λ_{abs} (nm)	λ_{em} (nm)	pK$_a$	ε (M^{-1} cm^{-1})	Φ	Φ_{ON}/Φ_{OFF}	Dynamic Intensity
ON[a]	581	603	9.68 ± 0.17	79073	0.75	3	866
OFF[b]	576	603		49872	0.27		

[a] Protonated form: pH 4

[b] Deprotonated form: pH 10

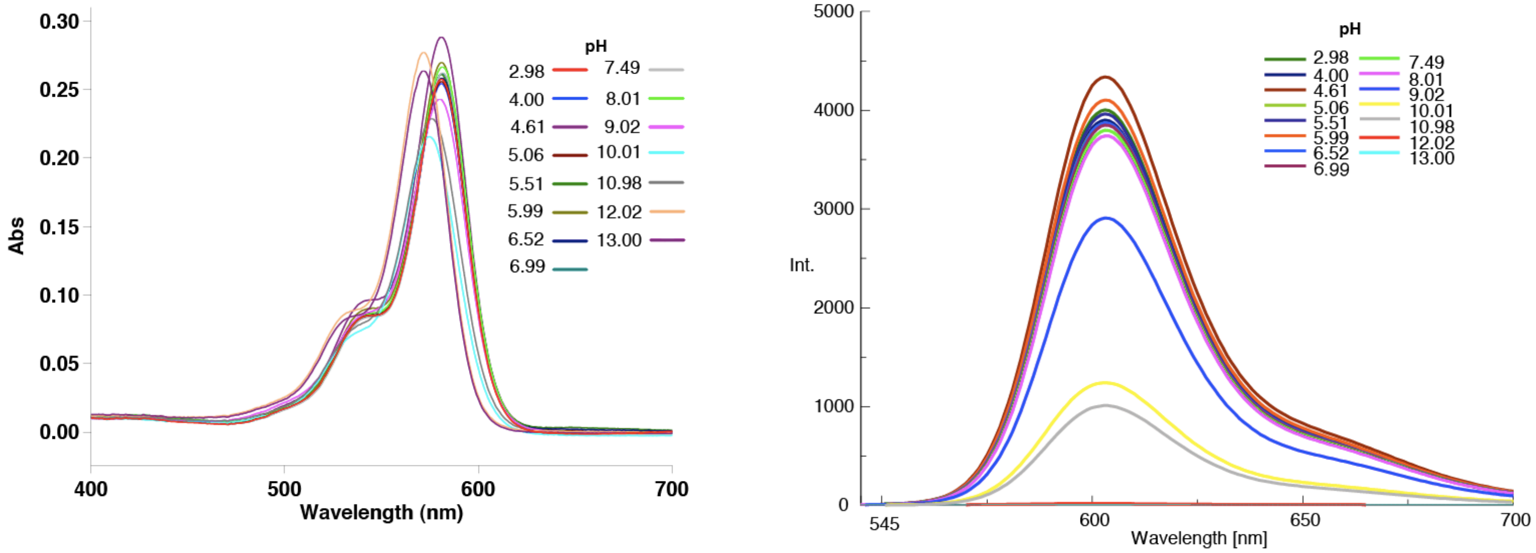

Left: Dependence of absorption on pH in a MOPS buffered aqueous solution. Right: Dependence of fluorescence intensity on pH in a MOPS buffered aqueous solution. Excitation at 535 nm.

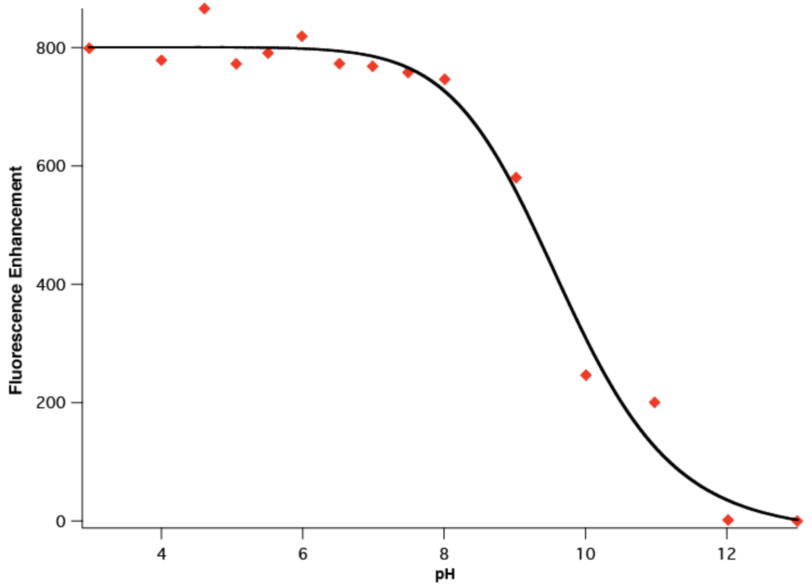

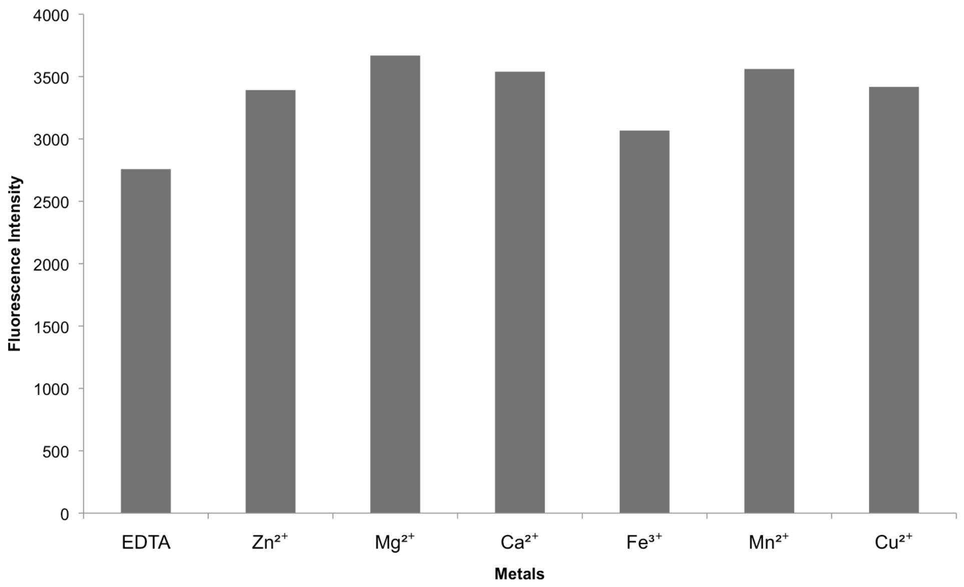

Left: Dependence of fluorescence enhancement on pH. Curve fitting was based on a modified Hill equation from which pK_a values were calculated. Right: Fluorescence intensities of the probe (5 μM) to a range of metal ions in a MOPS buffer (MOPS 30 mM, KCl 100 mM, pH 7.2). The concentration of cations is 10^{-3} M.

p-Nph

State	λ_{abs} (nm)	λ_{em} (nm)	pK$_a$	ε (M^{-1} cm^{-1})	Φ	Φ_{ON}/Φ_{OFF}	Dynamic Intensity
ON[a]	581	600	8.51 ± 0.17	62156	0.28	6	23
OFF[b]	579	602		22160	0.05		

[a] Protonated form: pH 4

[b] Deprotonated form: pH 10

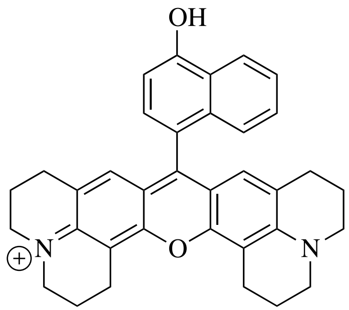

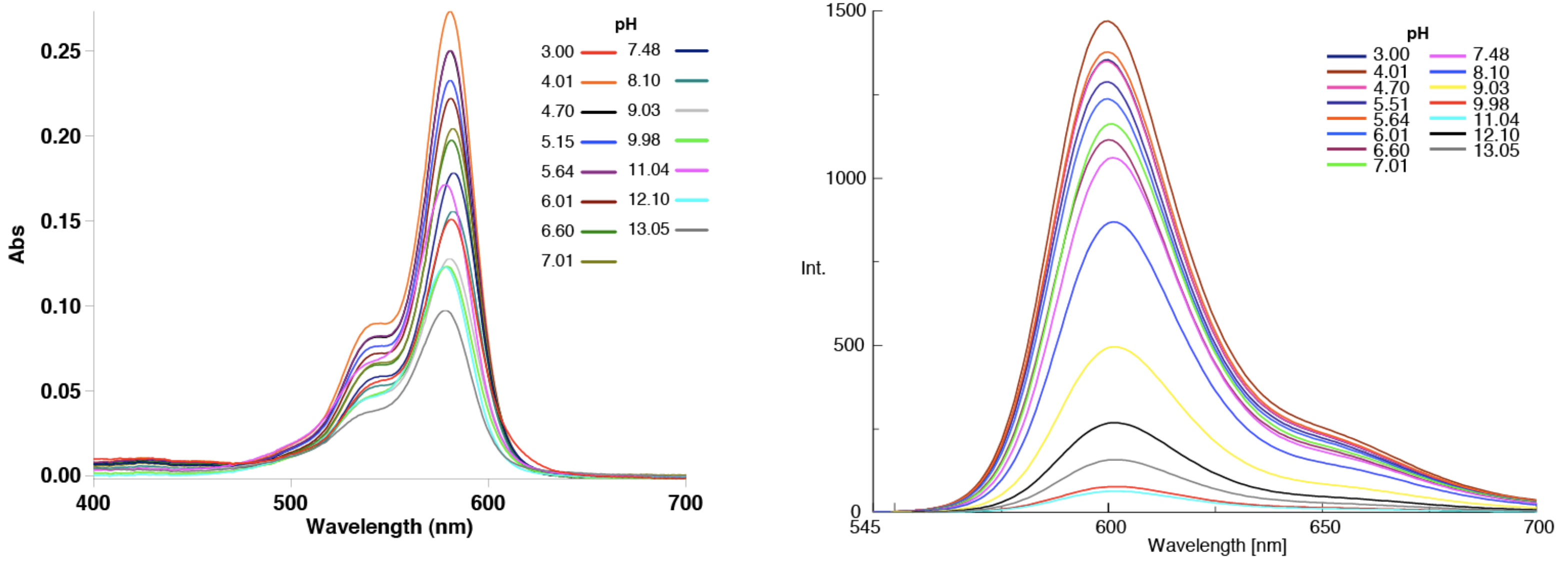

Left: Dependence of absorption on pH in a MOPS buffered aqueous solution. Right: Dependence of fluorescence intensity on pH in a MOPS buffered aqueous solution. Excitation at 535 nm.

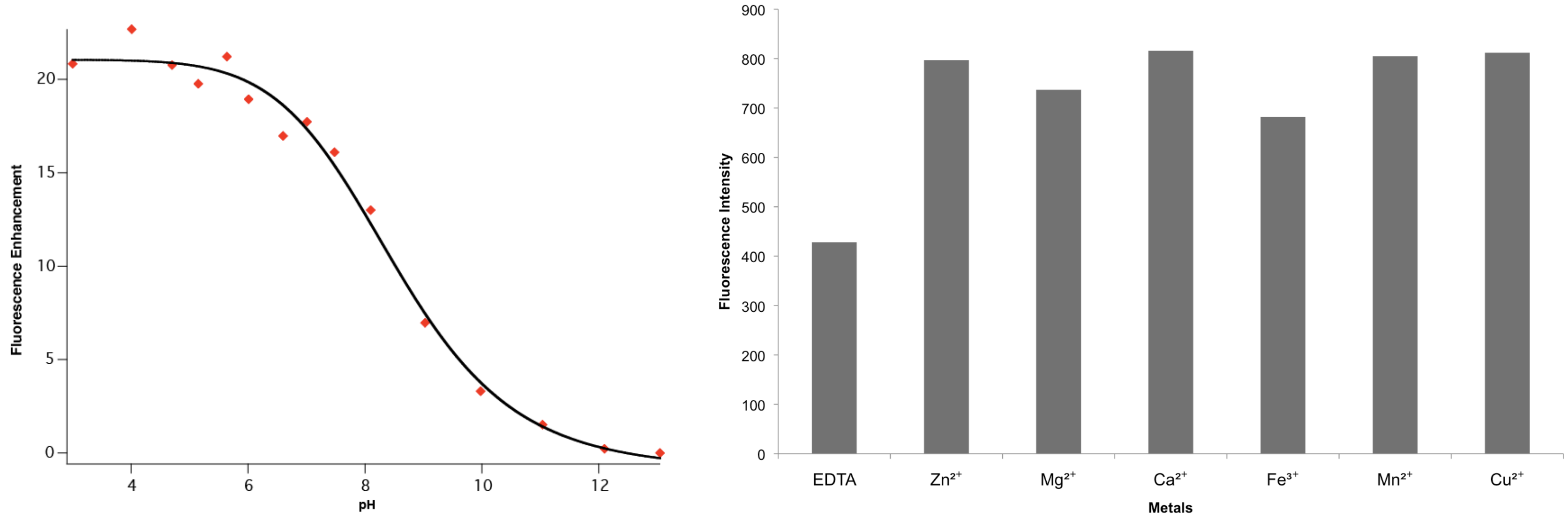

Left: Dependence of fluorescence enhancement on pH. Curve fitting was based on a modified Hill equation from which pK_a values were calculated. Right: Fluorescence intensities of the probe (5 μM) to a range of metal ions in a MOPS buffer (MOPS 30 mM, KCl 100 mM, pH 7.2). The concentration of cations is 10^{-3} M.

***o*-Nph**

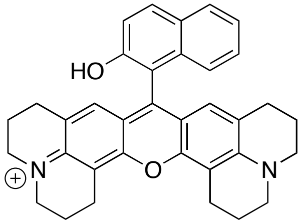

State	λ_{abs} (nm)	λ_{em} (nm)	pK$_a$	ε (M^{-1} cm^{-1})	Φ	Φ_{ON}/Φ_{OFF}	Dynamic Intensity
ON[a]	584	604	8.73 ± 0.17	26910	0.77	8	337
OFF[b]	576	604		28813	0.10		

[a] Protonated form: pH 4

[b] Deprotonated form: pH 10

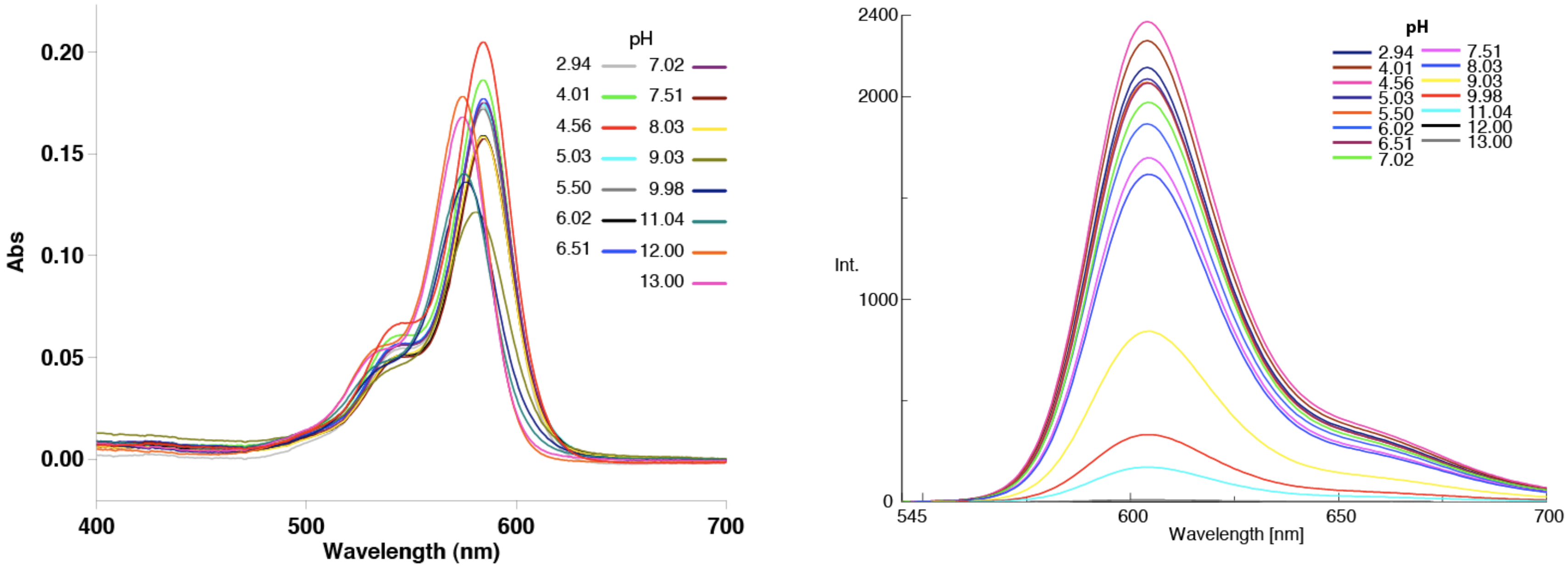

Left: Dependence of absorption on pH in a MOPS buffered aqueous solution. Right: Dependence of fluorescence intensity on pH in a MOPS buffered aqueous solution. Excitation at 535 nm.

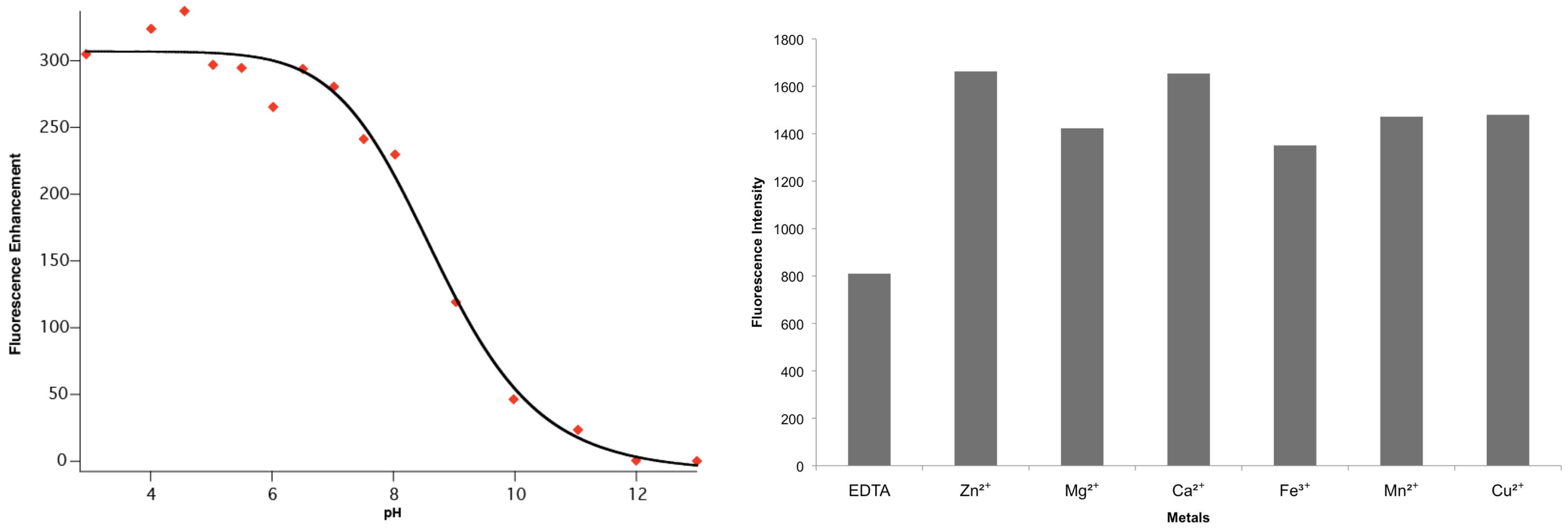

Left: Dependence of fluorescence enhancement on pH. Curve fitting was based on a modified Hill equation from which pK_a values were calculated. Right: Fluorescence intensities of the probe (5 μM) to a range of metal ions in a MOPS buffer (MOPS 30 mM, KCl 100 mM, pH 7.2). The concentration of cations is 10^{-3} M.

Pip-H

State	λ_{abs} (nm)	λ_{em} (nm)	pK_a	ε (M^{-1} cm^{-1})	Φ	Φ_{ON}/Φ_{OFF}	Dynamic Intensity
ON[a]	578	600	8.67 ± 0.07	76220	0.16	4	6
OFF[b]	579	601		69710	0.04		

[a] Protonated form: pH 4

[b] Deprotonated form: pH 10

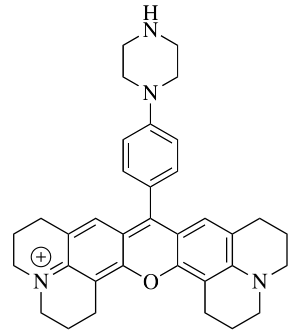

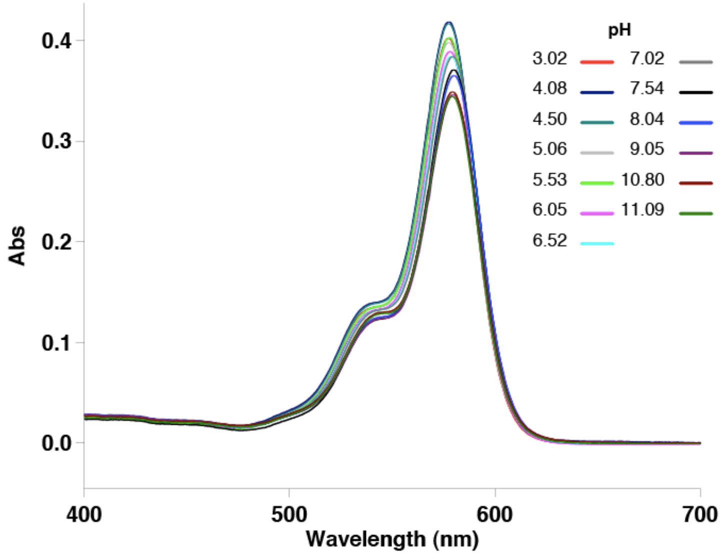

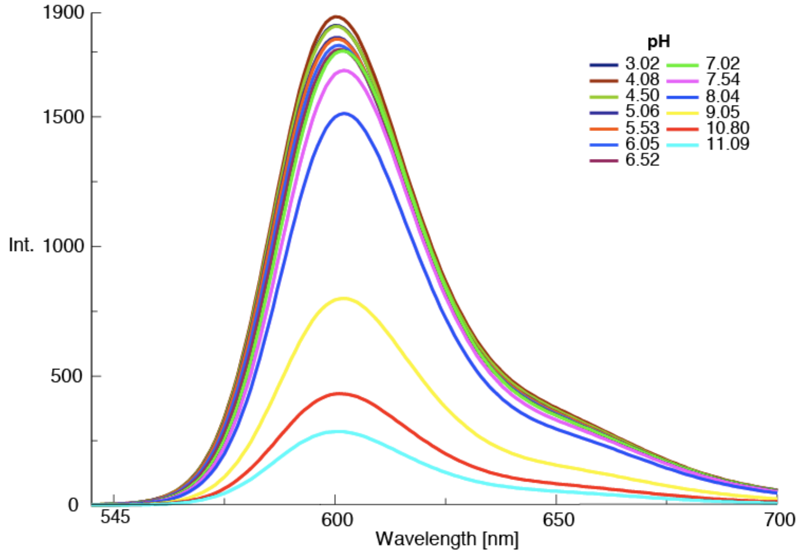

Left: Dependence of absorption on pH in a MOPS buffered aqueous solution. Right: Dependence of fluorescence intensity on pH in a MOPS buffered aqueous solution. Excitation at 535 nm.

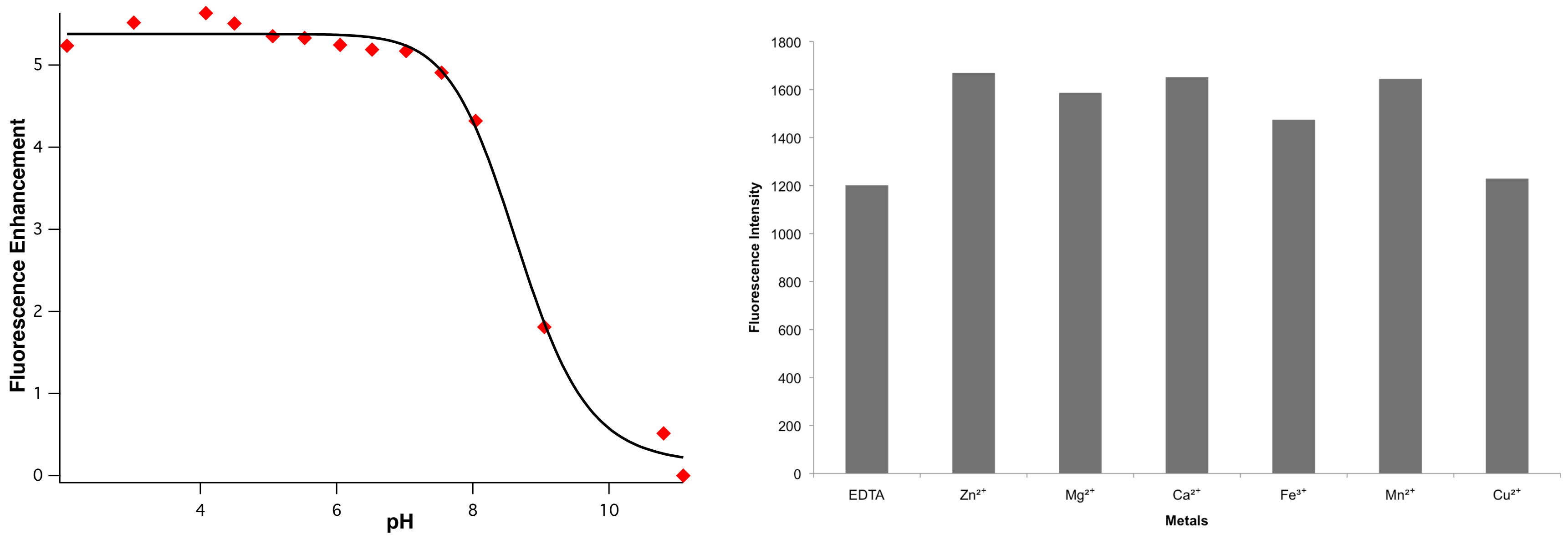

Left: Dependence of fluorescence enhancement on pH. Curve fitting was based on a modified Hill equation from which pK_a values were calculated. Right: Fluorescence intensities of the probe (5 μM) to a range of metal ions in a MOPS buffer (MOPS 30 mM, KCl 100 mM, pH 7.2). The concentration of cations is 10^{-3} M.

Pip-Alkyne

State	λ_{abs} (nm)	λ_{em} (nm)	pK_a	ε (M^{-1} cm^{-1})	Φ	Φ_{ON}/Φ_{OFF}	Dynamic Intensity
ON[a]	577	600	4.95 ± 0.09	95685	0.34	2	3
OFF[b]	579	602		28554	0.15		

[a] Protonated form: pH 4

[b] Deprotonated form: pH 10

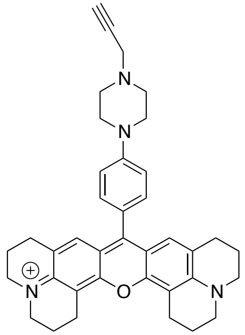

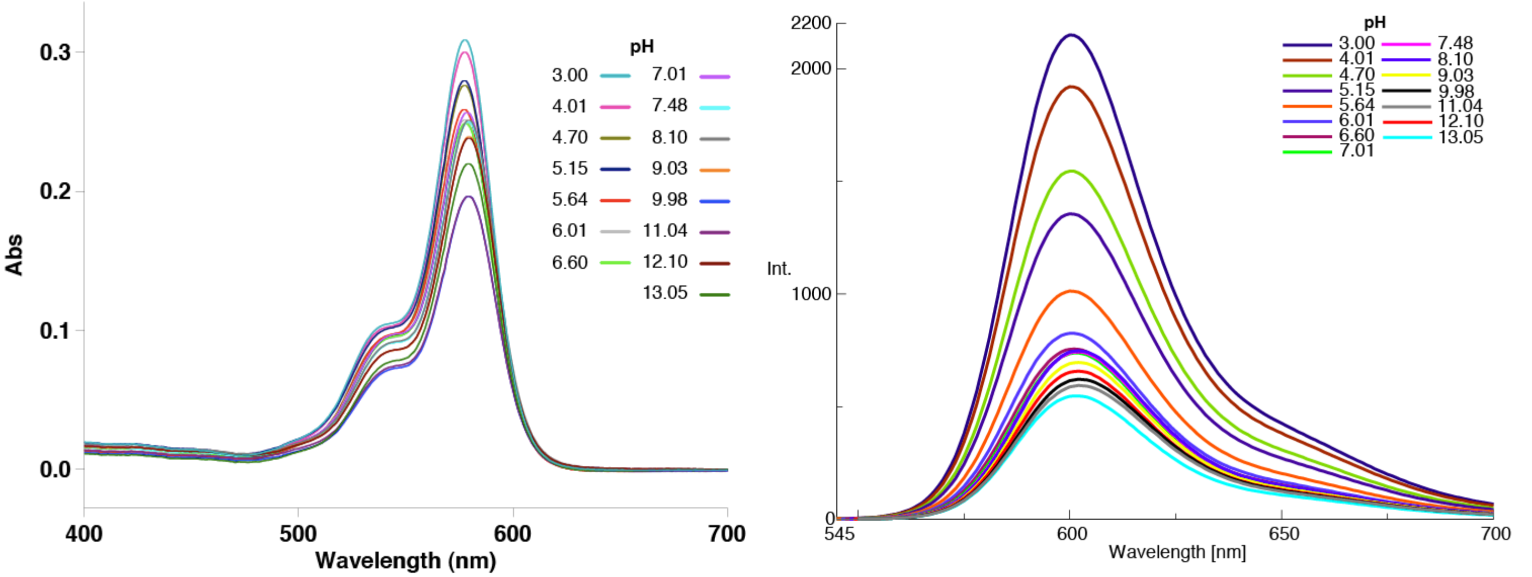

Left: Dependence of absorption on pH in a MOPS buffered aqueous solution. Right: Dependence of fluorescence intensity on pH in a MOPS buffered aqueous solution. Excitation at 535 nm.

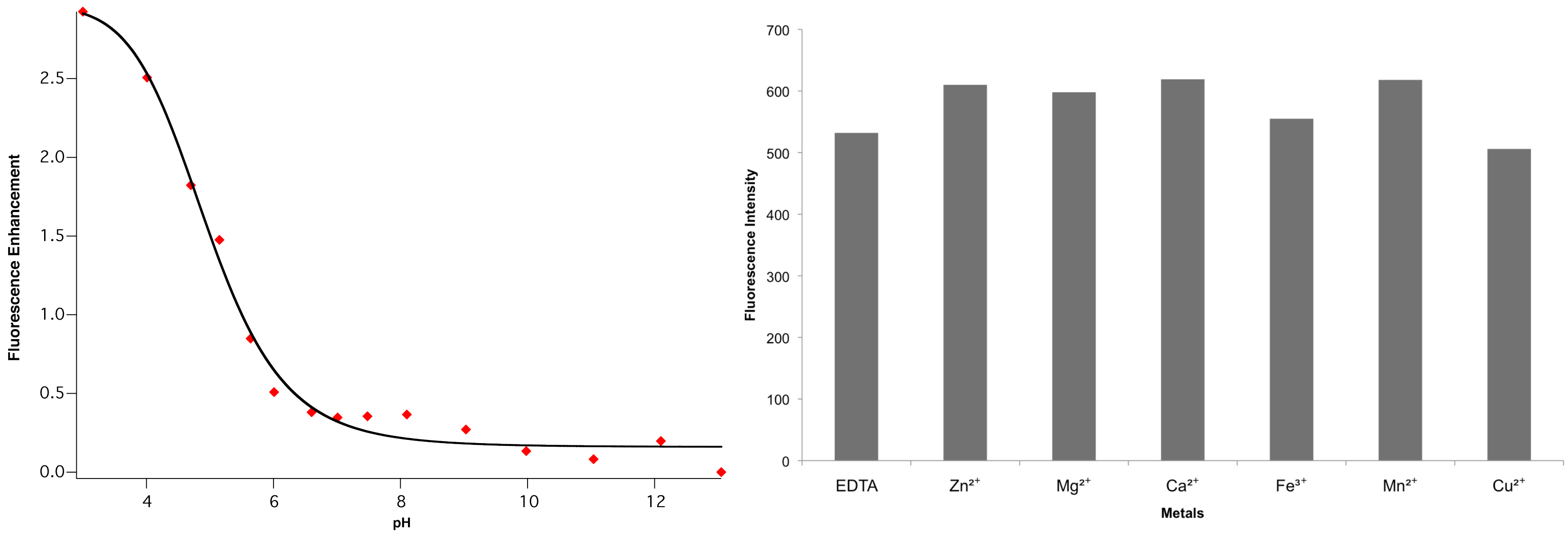

Left: Dependence of fluorescence enhancement on pH. Curve fitting was based on a modified Hill equation from which pK_a values were calculated. Right: Fluorescence intensities of the probe (5 μM) to a range of metal ions in a MOPS buffer (MOPS 30 mM, KCl 100 mM, pH 7.2). The concentration of cations is 10^{-3} M.

HR-PiAC

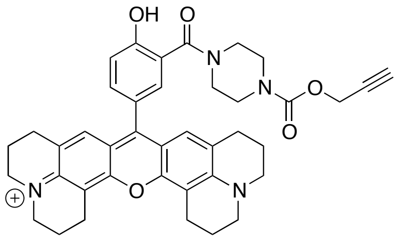

State	λ_{abs} (nm)	λ_{em} (nm)	pK_a	ε (M^{-1} cm^{-1})	Φ	Φ_{ON}/Φ_{OFF}	Dynamic Intensity
ON[a]	580	601	7.68 ± 0.09	72405	0.64	40	86
OFF[b]	575	602		49298	0.02		

[a] Protonated form: pH 4

[b] Deprotonated form: pH 10

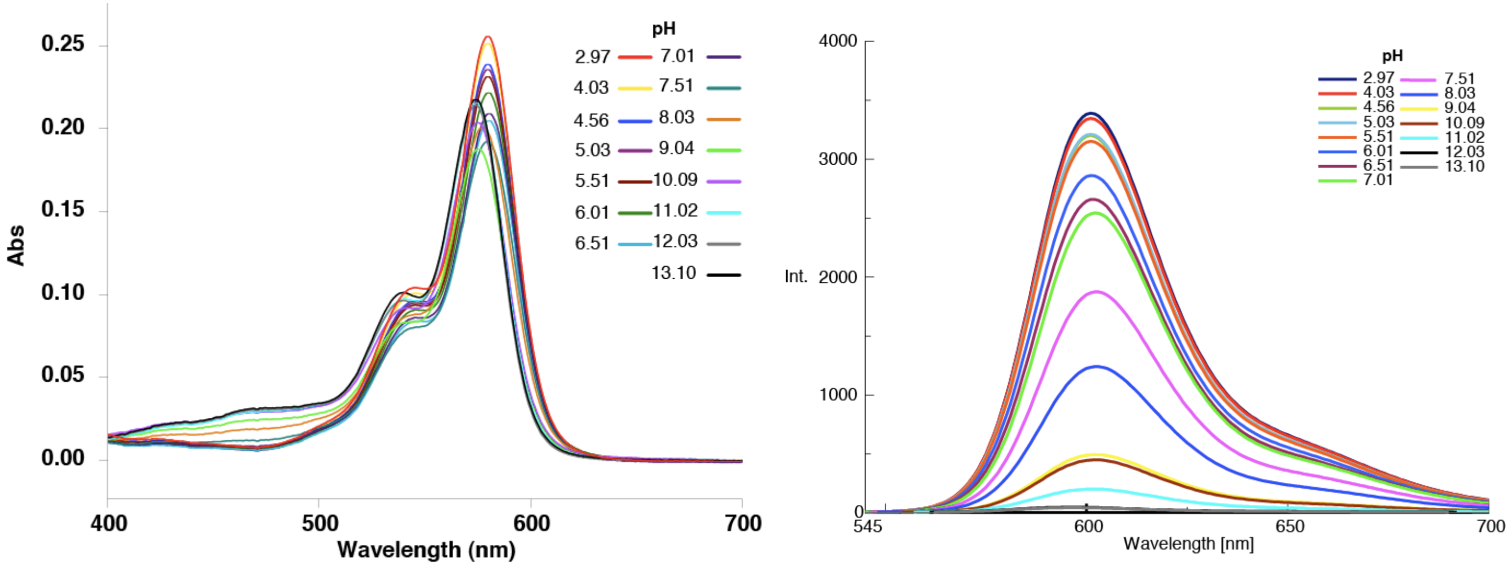

Left: Dependence of absorption on pH in a MOPS buffered aqueous solution. Right: Dependence of fluorescence intensity on pH in a MOPS buffered aqueous solution. Excitation at 535 nm.

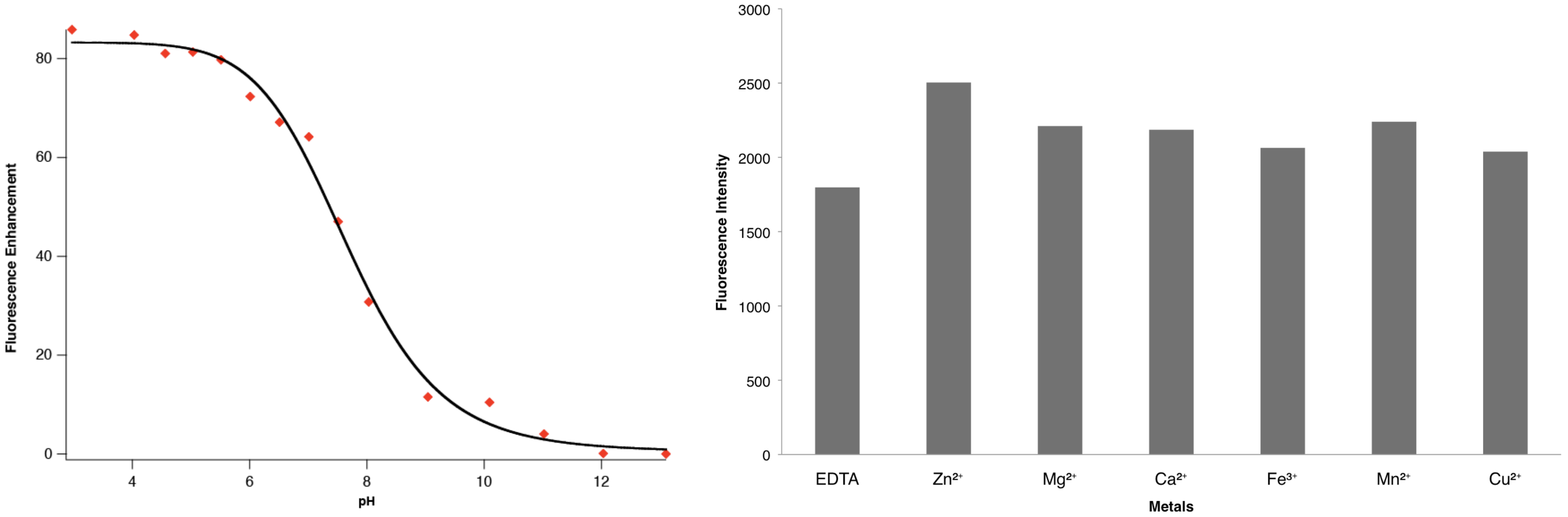

Left: Dependence of fluorescence enhancement on pH. Curve fitting was based on a modified Hill equation from which pK_a values were calculated. Right: Fluorescence intensities of the probe (5 μM) to a range of metal ions in a MOPS buffer (MOPS 30 mM, KCl 100 mM, pH 7.2). The concentration of cations is 10^{-3} M.

HR-PiA

State	λ_{abs} (nm)	λ_{em} (nm)	pK_a	ε (M^{-1} cm^{-1})	Φ	Φ_{ON}/Φ_{OFF}	Dynamic Intensity
ON[a]	580	601	7.64 ± 0.10	35109	0.80	35	98
OFF[b]	574	600		20712	0.02		

[a] Protonated form: pH 4

[b] Deprotonated form: pH 10

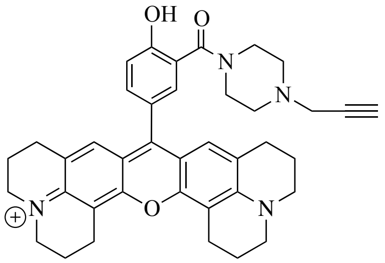

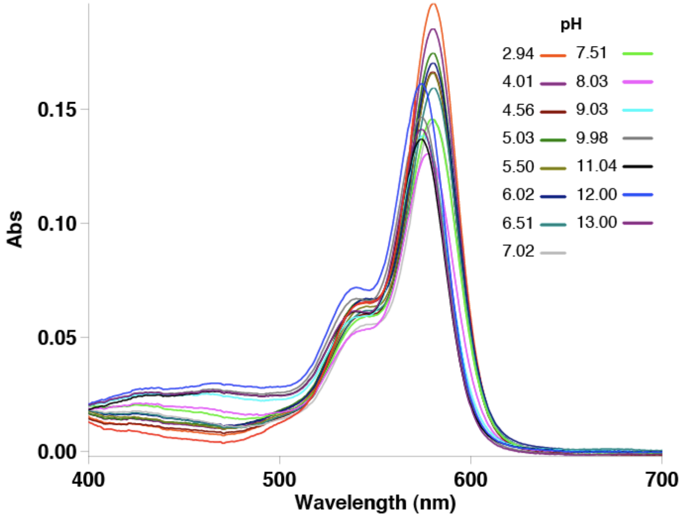

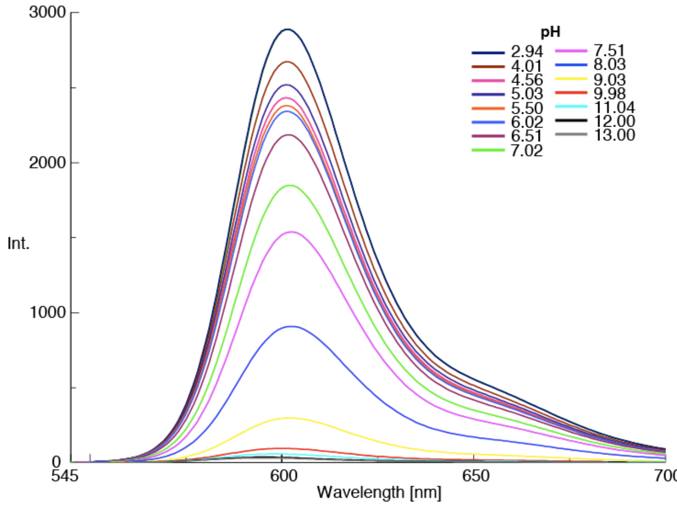

Left: Dependence of absorption on pH in a MOPS buffered aqueous solution. Right: Dependence of fluorescence intensity on pH in a MOPS buffered aqueous solution. Excitation at 535 nm.

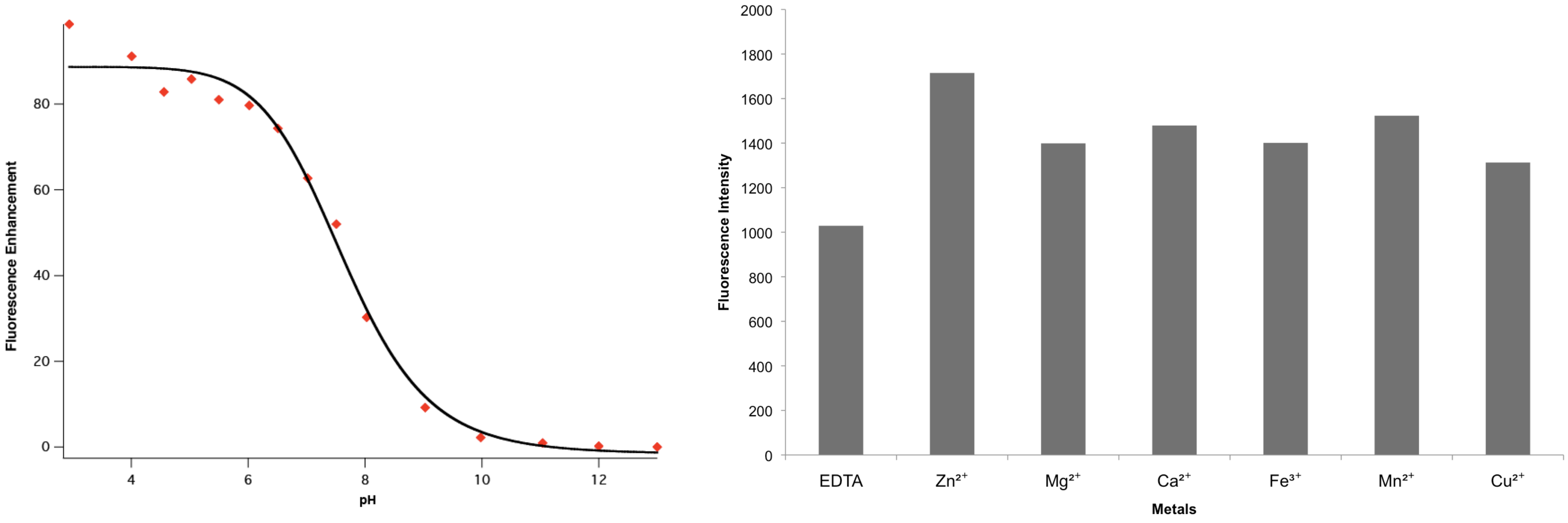

Left: Dependence of fluorescence enhancement on pH. Curve fitting was based on a modified Hill equation from which pK_a values were calculated. Right: Fluorescence intensities of the probe (5 μM) to a range of metal ions in a MOPS buffer (MOPS 30 mM, KCl 100 mM, pH 7.2). The concentration of cations is 10^{-3} M.

HR-A

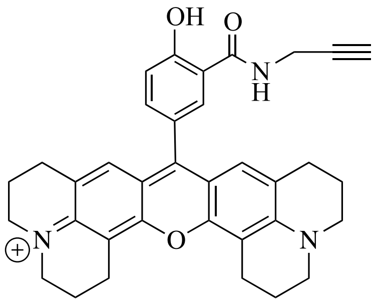

State	λ_{abs} (nm)	λ_{em} (nm)	pK_a	ε (M^{-1} cm^{-1})	Φ	Φ_{ON}/Φ_{OFF}	Dynamic Intensity
ON[a]	574	600	5.33 ± 0.13	18421	0.09	6	6
OFF[b]	534	598		38042	0.02		

[a] Protonated form: pH 4

[b] Deprotonated form: pH 10

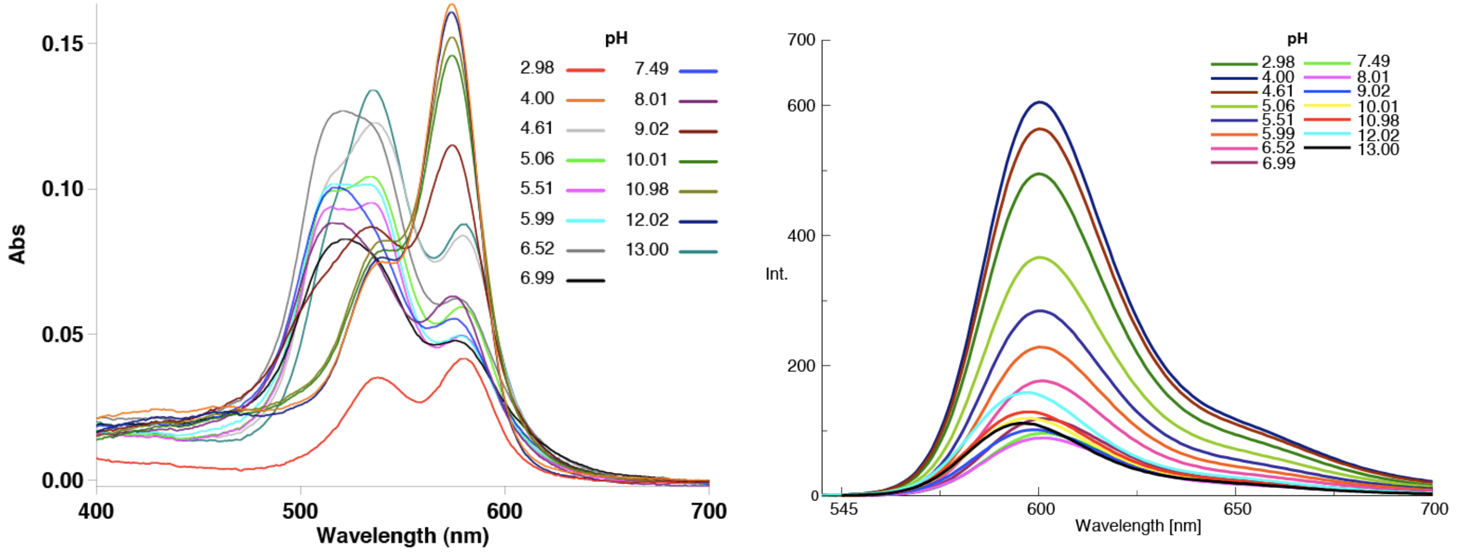

Left: Dependence of absorption on pH in a MOPS buffered aqueous solution. Right: Dependence of fluorescence intensity on pH in a MOPS buffered aqueous solution. Excitation at 535 nm.

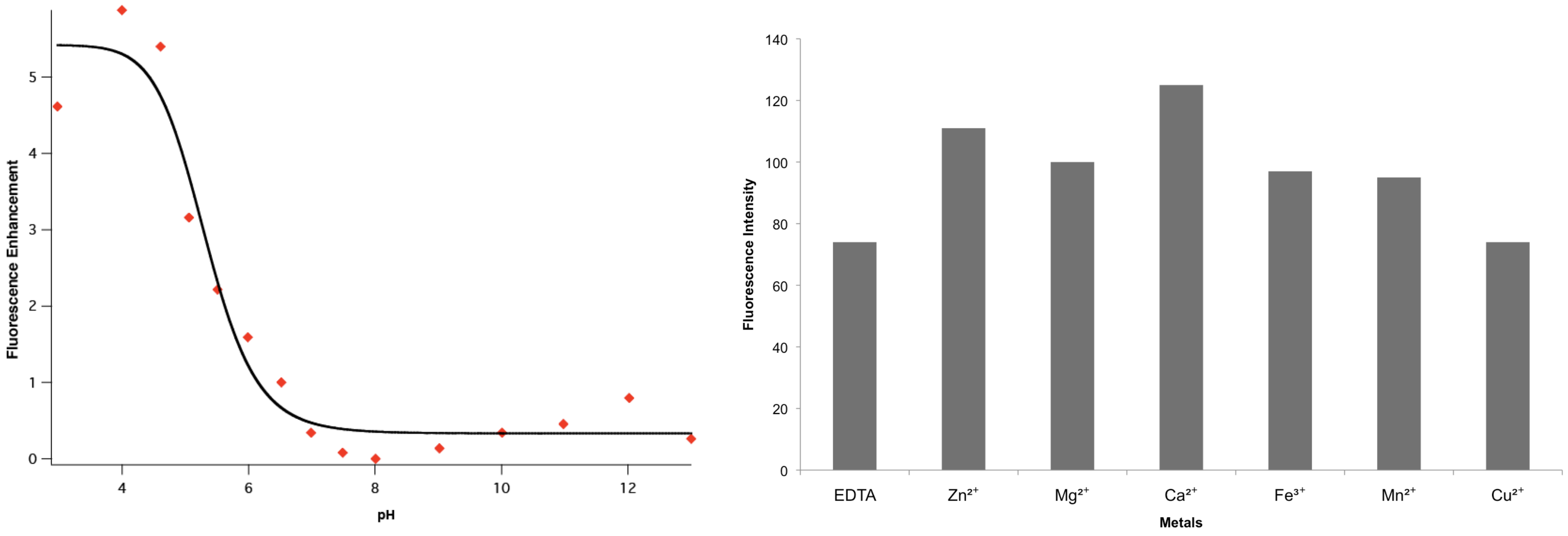

Left: Dependence of fluorescence enhancement on pH. Curve fitting was based on a modified Hill equation from which pK_a values were calculated. Right: Fluorescence intensities of the probe (5 μM) to a range of metal ions in a MOPS buffer (MOPS 30 mM, KCl 100 mM, pH 7.2). The concentration of cations is 10^{-3} M.

HR-N_3

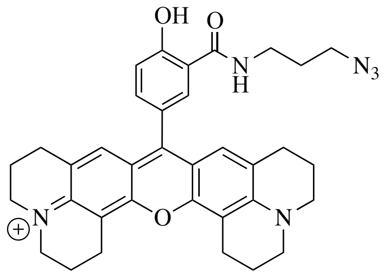

State	λ_{abs} (nm)	λ_{em} (nm)	pK_a	ε (M^{-1} cm^{-1})	Φ	Φ_{ON}/Φ_{OFF}	Dynamic Intensity
ON[a]	580	600	3.96 ± 0.03	26742	0.25	28	88
OFF[b]	574	596		26706	0.01		

[a] Protonated form: pH 4

[b] Deprotonated form: pH 10

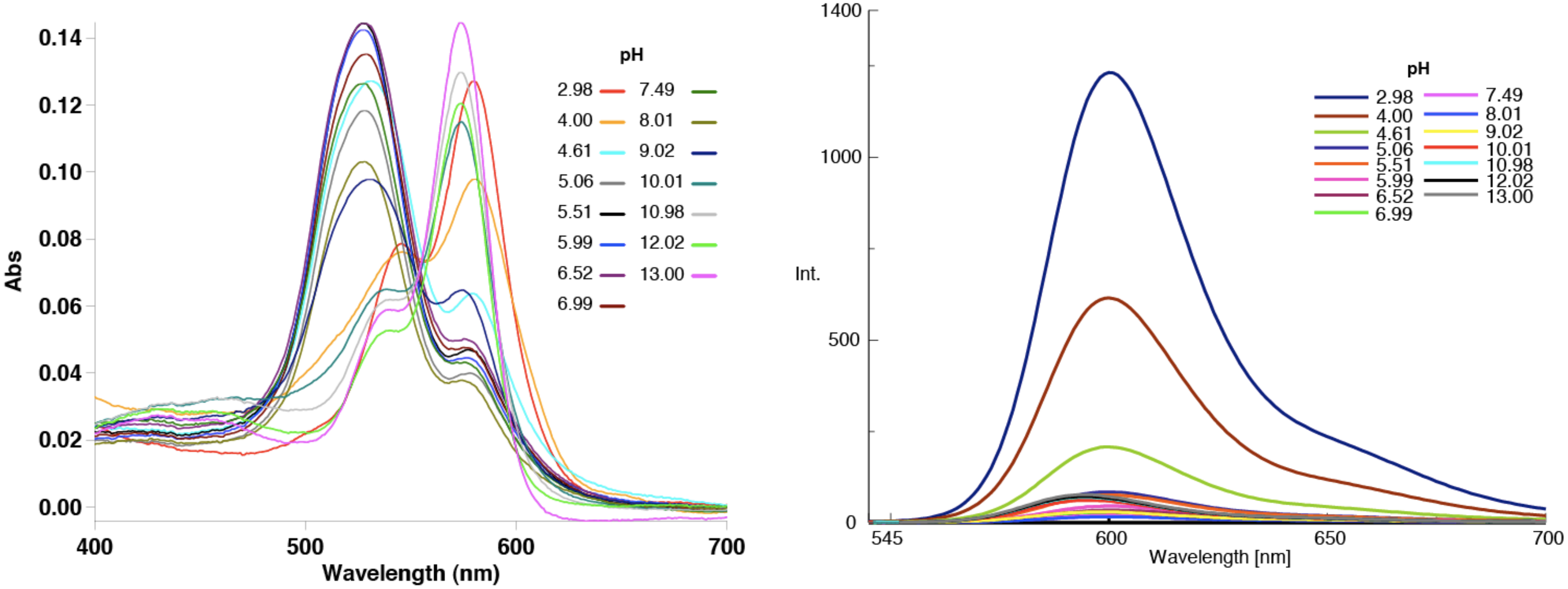

Left: Dependence of absorption on pH in a MOPS buffered aqueous solution. Right: Dependence of fluorescence intensity on pH in a MOPS buffered aqueous solution. Excitation at 535 nm.

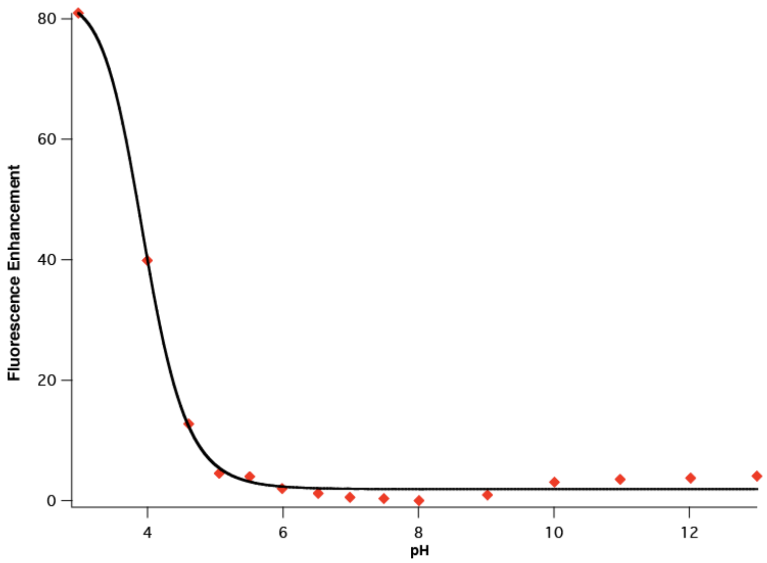

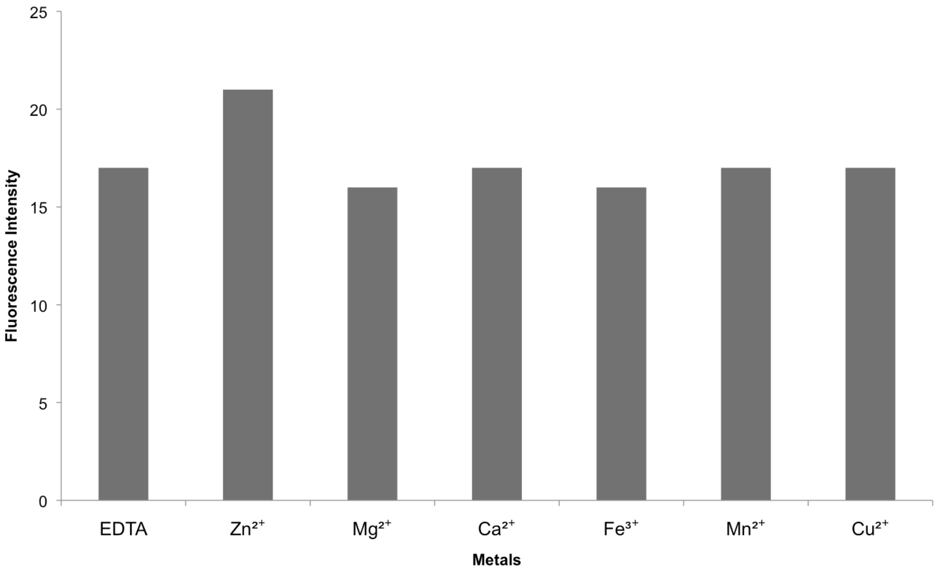

Left: Dependence of fluorescence enhancement on pH. Curve fitting was based on a modified Hill equation from which pK_a values were calculated. Right: Fluorescence intensities of the probe (5 μM) to a range of metal ions in a MOPS buffer (MOPS 30 mM, KCl 100 mM, pH 7.2). The concentration of cations is 10^{-3} M.

HR-Ala

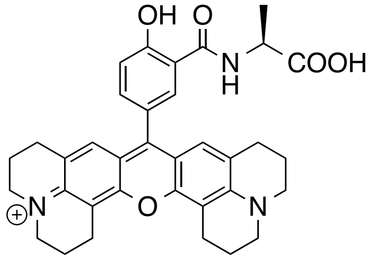

State	λ_{abs} (nm)	λ_{em} (nm)	pK_a	ε (M^{-1} cm^{-1})	Φ	Φ_{ON}/Φ_{OFF}	Dynamic Intensity
ON[a]	577	601	7.85 ± 0.03	65000	0.15	8	9
OFF[b]	573	597		107500	0.019		

[a] Protonated form: pH 4

[b] Deprotonated form: pH 10

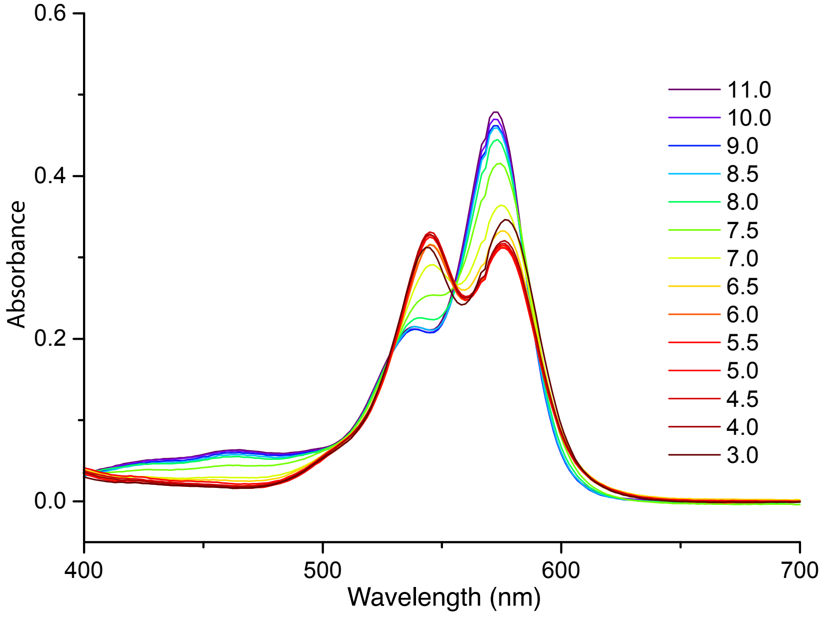

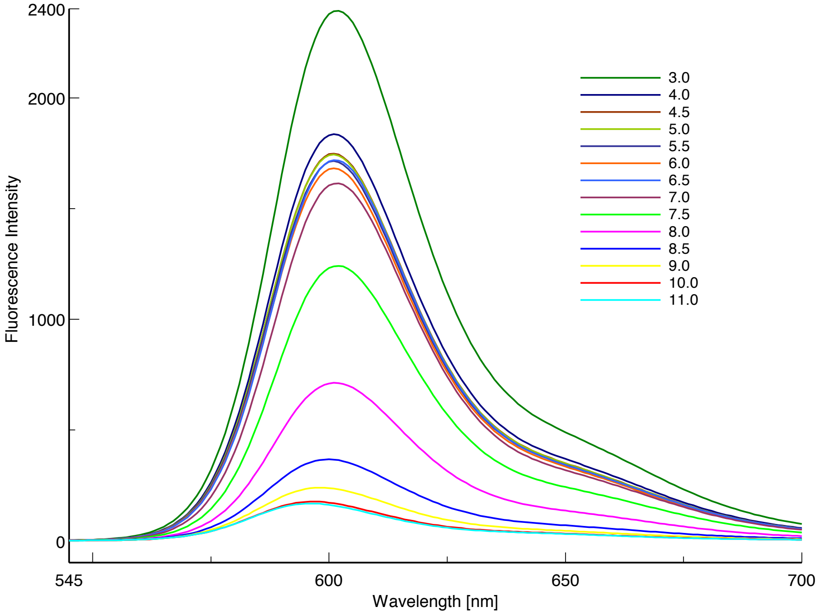

Left: Dependence of absorption on pH in a MOPS buffered aqueous solution. Right: Dependence of fluorescence intensity on pH in a MOPS buffered aqueous solution. Excitation at 535 nm.

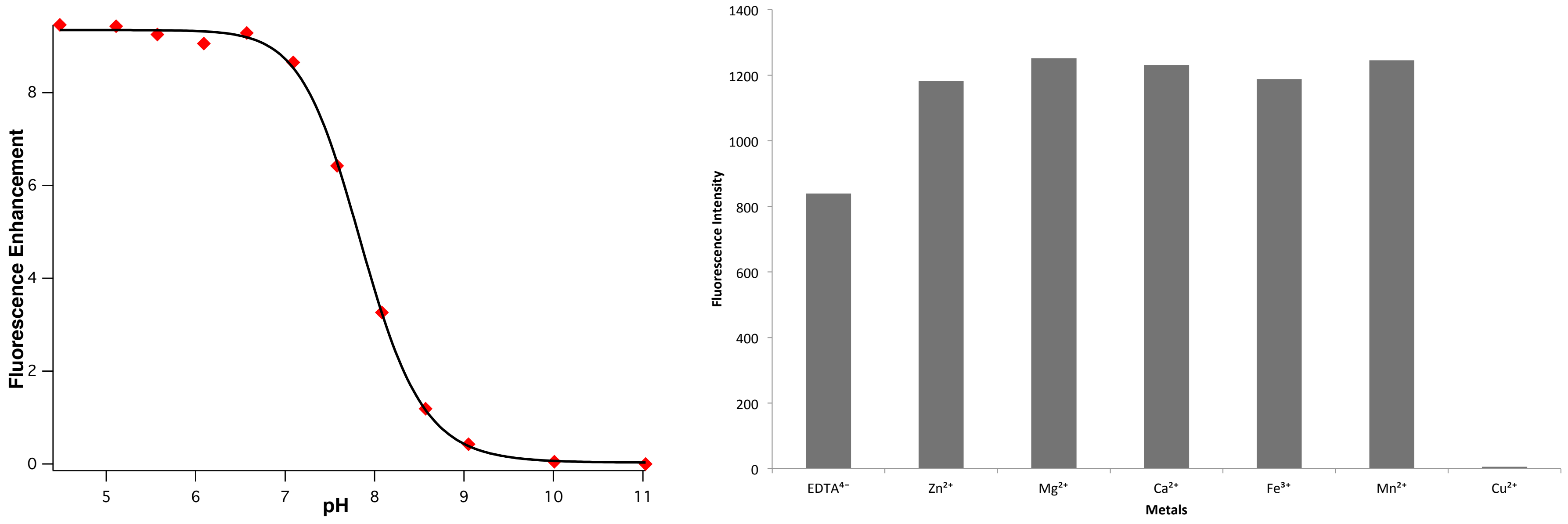

Left: Dependence of fluorescence enhancement on pH. Curve fitting was based on a modified Hill equation from which pK_a values were calculated. Right: Fluorescence intensities of the probe (5 μM) to a range of metal ions in a MOPS buffer (MOPS 30 mM, KCl 100 mM, pH 7.2). The concentration of cations is 10^{-3} M.

HR-βAla

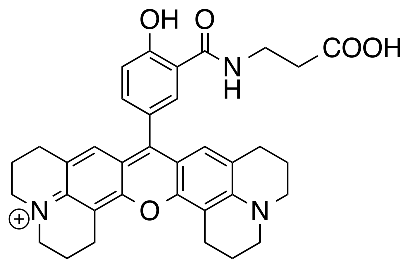

State	λ_{abs} (nm)	λ_{em} (nm)	pK_a	ε (M^{-1} cm^{-1})	Φ	Φ_{ON}/Φ_{OFF}	Dynamic Intensity
ON[a]	577	602	N/A	128000	0.20	11	12
OFF[b]	572	598		202000	0.018		

[a] Protonated form: pH 4

[b] Deprotonated form: pH 10

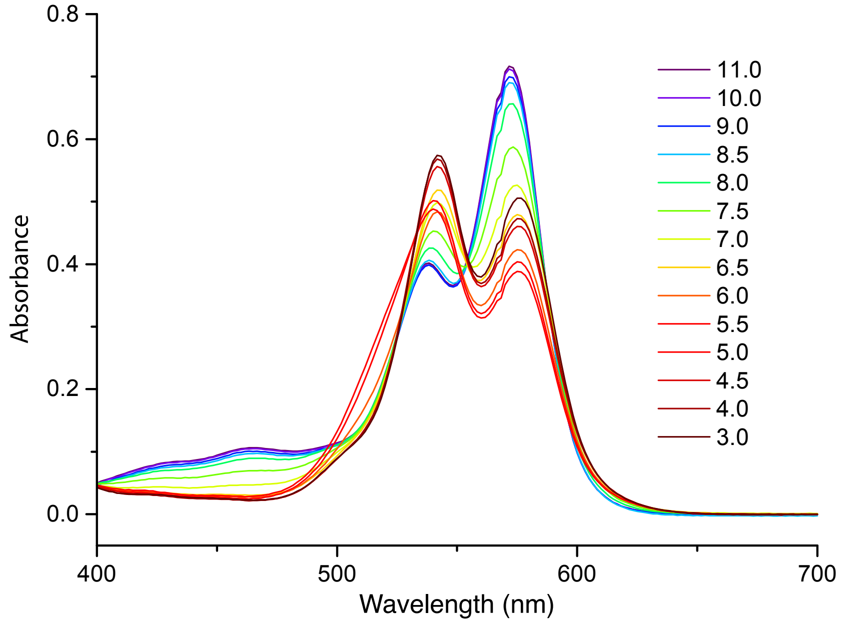

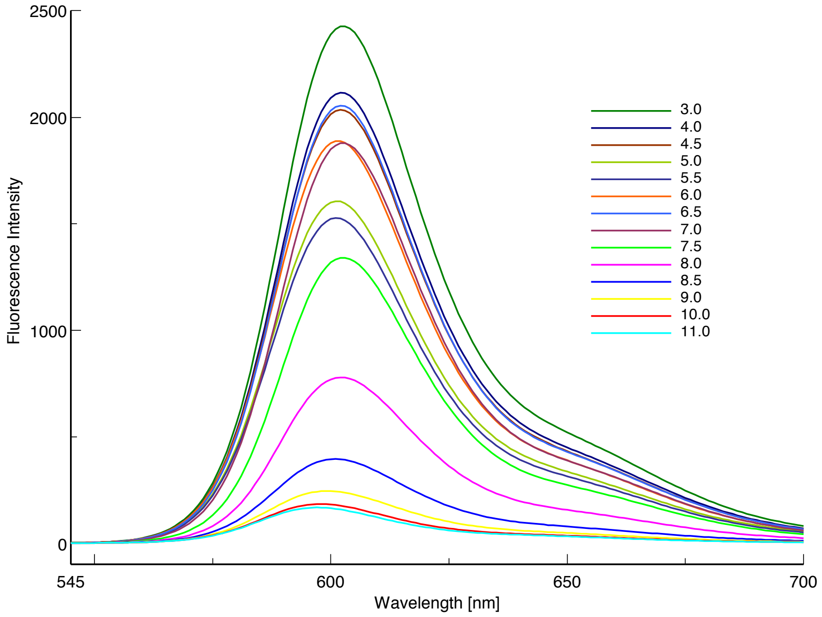

Left: Dependence of absorption on pH in a MOPS buffered aqueous solution. Right: Dependence of fluorescence intensity on pH in a MOPS buffered aqueous solution. Excitation at 535 nm.

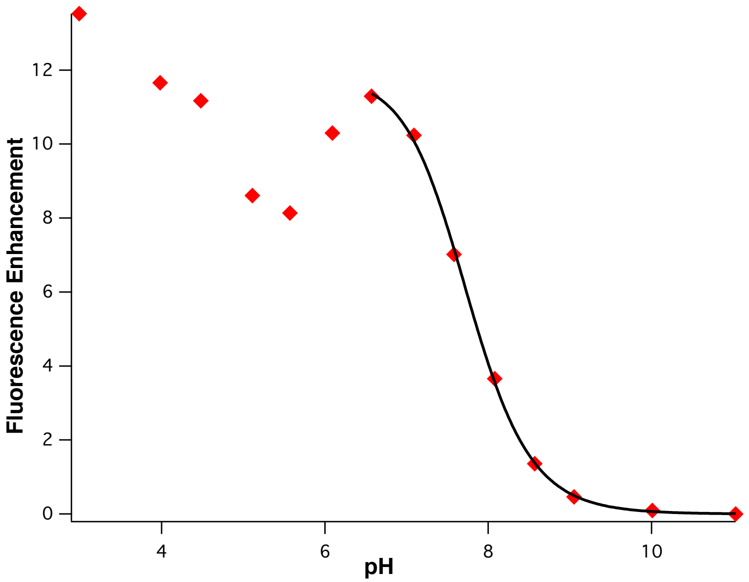

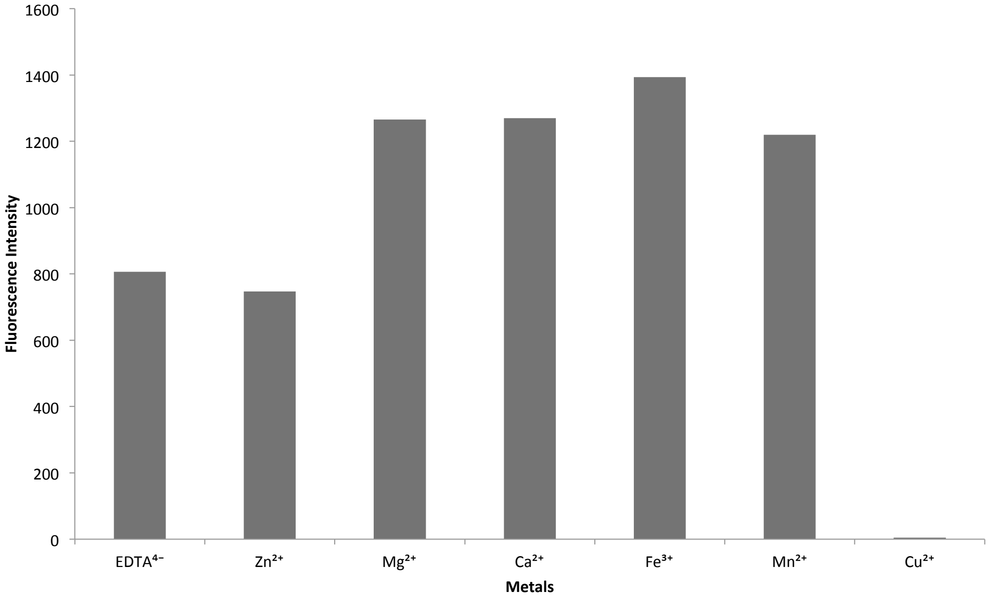

Left: Dependence of fluorescence enhancement on pH. Curve fitting was based on a modified Hill equation from which pK_a values were calculated. Fluorescence intensities of the probe (5 μM) to a range of metal ions in a MOPS buffer (MOPS 30 mM, KCl 100 mM, pH 7.2). The concentration of cations is 10^{-3} M.

HR-PN$_3$

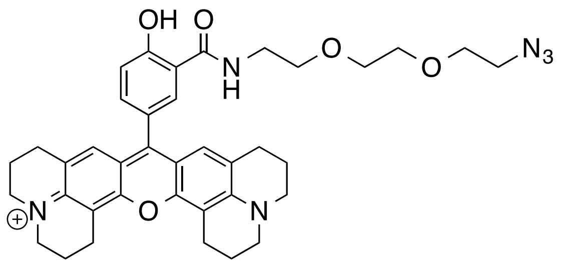

State	λ_{abs} (nm)	λ_{em} (nm)	pK$_a$	ε (M^{-1} cm^{-1})	Φ	Φ_{ON}/Φ_{OFF}	Dynamic Intensity
ON[a]	578	603	6.20 ± 0.03	52500	0.41	21	21
OFF[b]	573	597		55500	0.02		

[a] Protonated form: pH 4

[b] Deprotonated form: pH 10

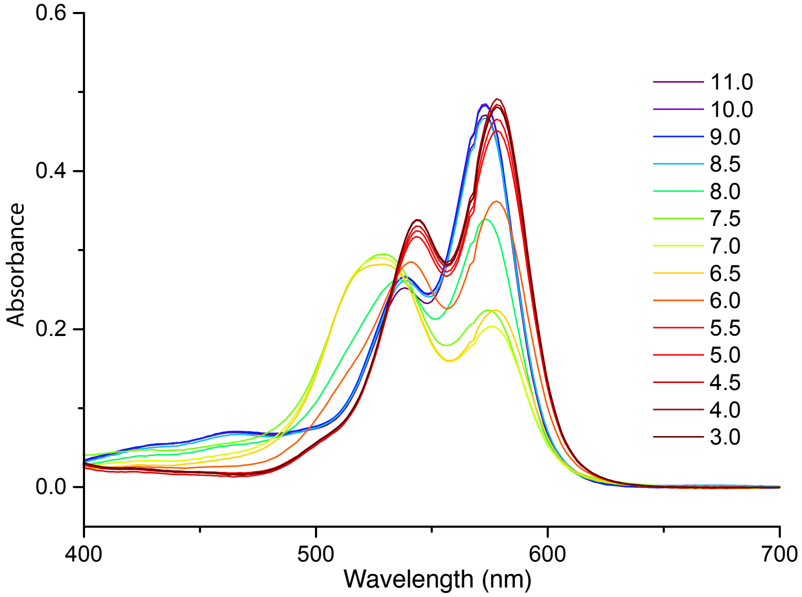

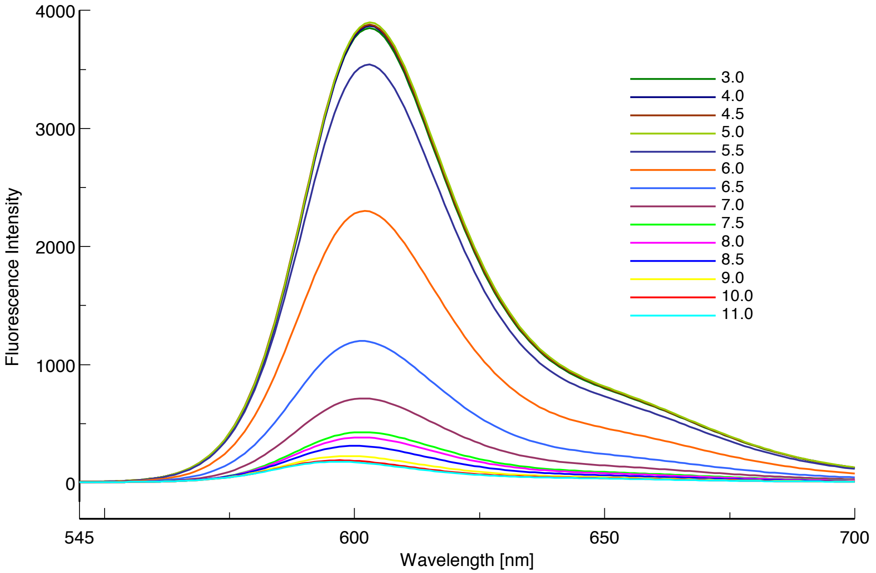

Left: Dependence of absorption on pH in a MOPS buffered aqueous solution. Right: Dependence of fluorescence intensity on pH in a MOPS buffered aqueous solution. Excitation at 535 nm.

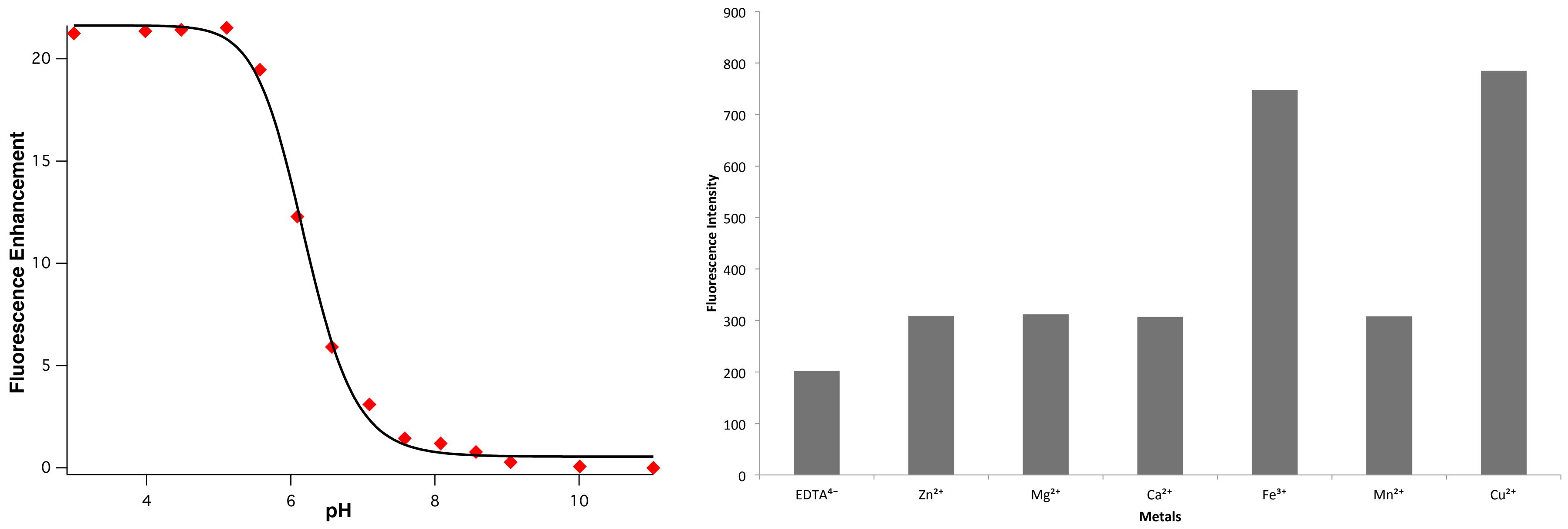

Left: Dependence of fluorescence enhancement on pH. Curve fitting was based on a modified Hill equation from which pK_a values were calculated. Right: Fluorescence intensities of the probe (5 μM) to a range of metal ions in a MOPS buffer (MOPS 30 mM, KCl 100 mM, pH 7.2). The concentration of cations is 10^{-3} M.

HR-CysA

State	λ_{abs} (nm)	λ_{em} (nm)	pK_a	ε (M^{-1} cm^{-1})	Φ	Φ_{ON}/Φ_{OFF}	Dynamic Intensity
ON[a]	577	602	N/A	49000	0.09	N/A	N/A
OFF[b]	572	597		123000	0.014		

[a] Protonated form: pH 4

[b] Deprotonated form: pH 10

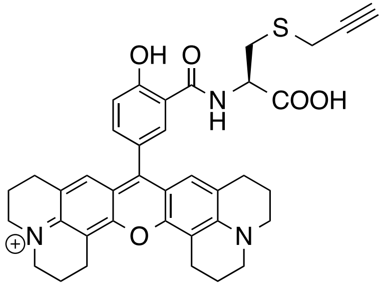

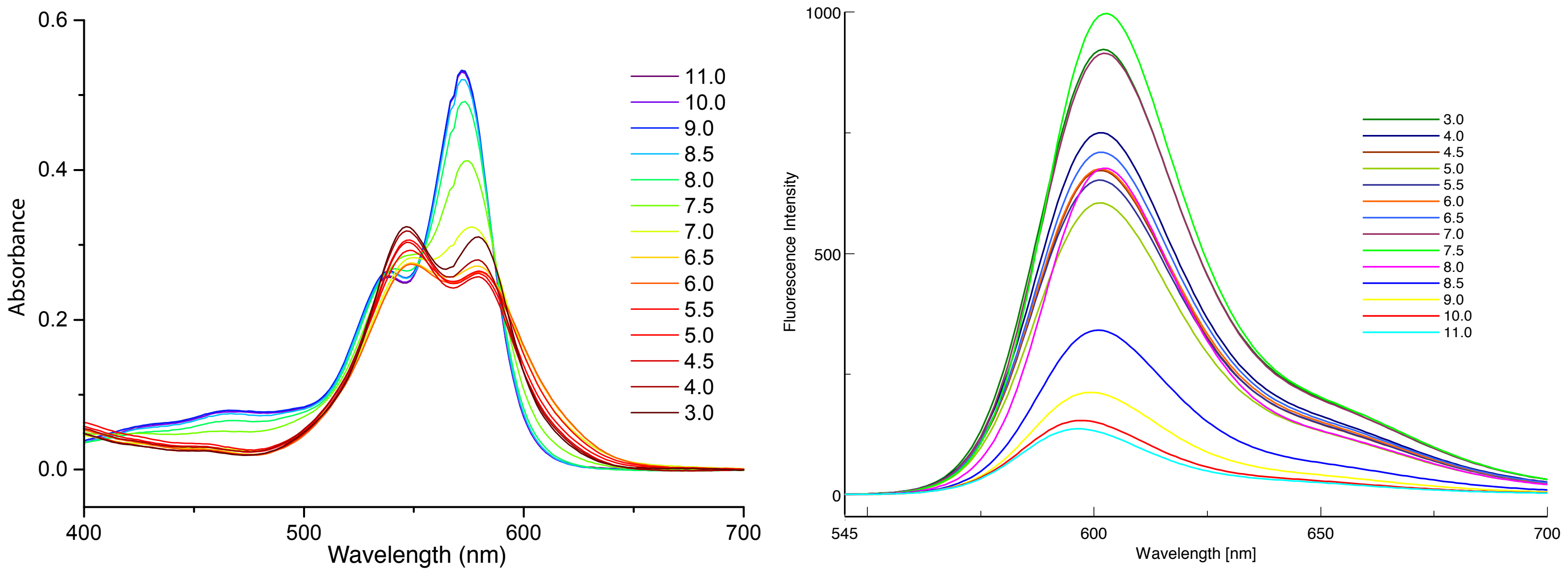

Left: Dependence of absorption on pH in a MOPS buffered aqueous solution. Right: Dependence of fluorescence intensity on pH in a MOPS buffered aqueous solution. Excitation at 535 nm.

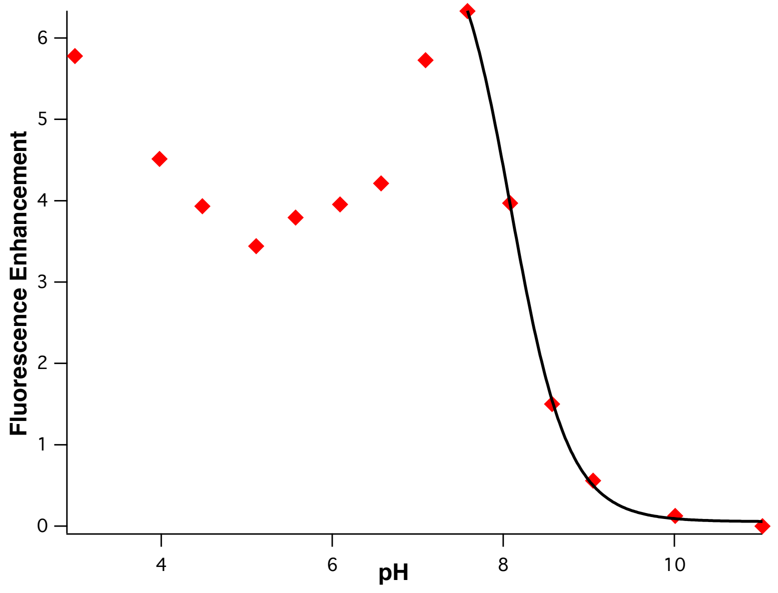

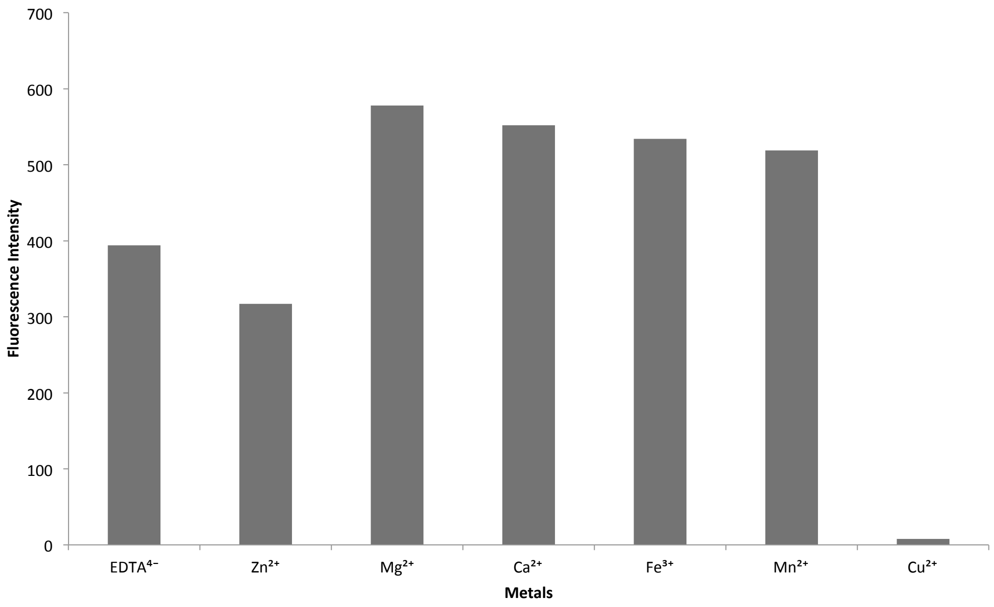

Left: Dependence of fluorescence enhancement on pH. Curve fitting was based on a modified Hill equation from which pK_a values were calculated. Right: Fluorescence intensities of the probe (5 μM) to a range of metal ions in a MOPS buffer (MOPS 30 mM, KCl 100 mM, pH 7.2). The concentration of cations is 10^{-3} M.

HR-MPN$_3$

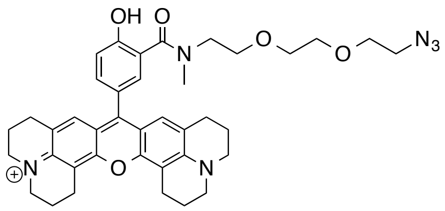

State	λ_{abs} (nm)	λ_{em} (nm)	pK_a	ε (M^{-1} cm^{-1})	Φ	Φ_{ON}/Φ_{OFF}	Dynamic Intensity
ON[a]	578	605	8.00 ± 0.03	162000	0.65	65	78
OFF[b]	573	603		162000	0.01		

[a] Protonated form: pH 4

[b] Deprotonated form: pH 11

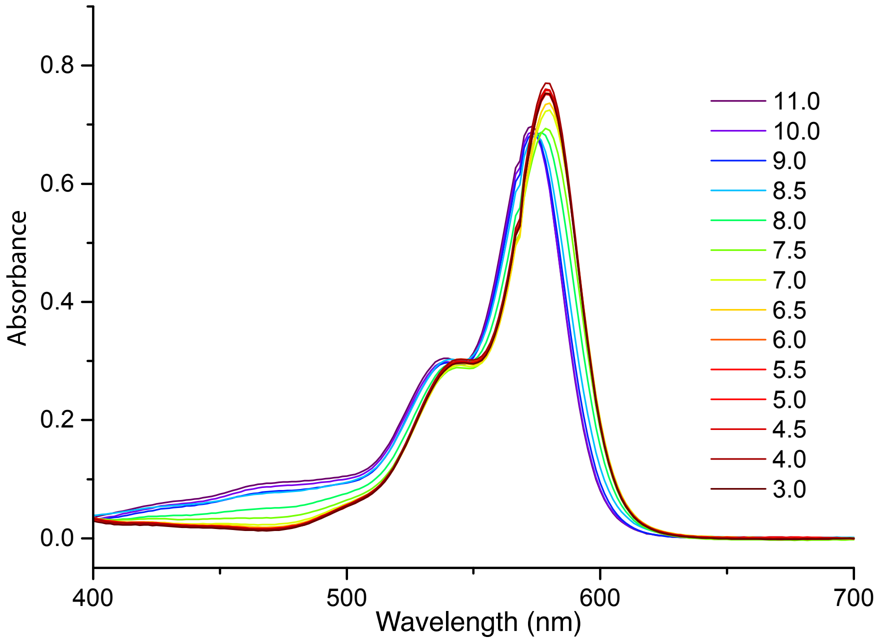

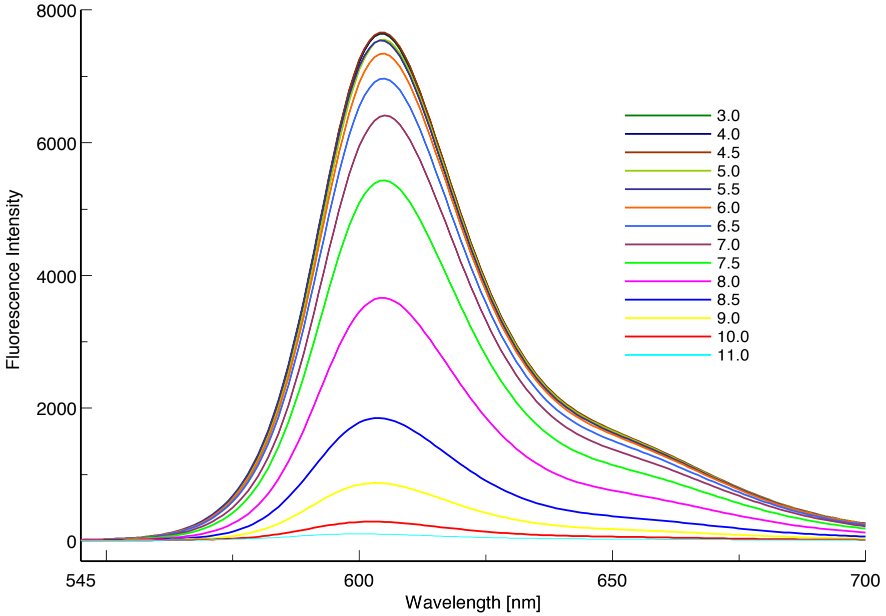

Left: Dependence of absorption on pH in a MOPS buffered aqueous solution. Right: Dependence of fluorescence intensity on pH in a MOPS buffered aqueous solution. Excitation at 535 nm.

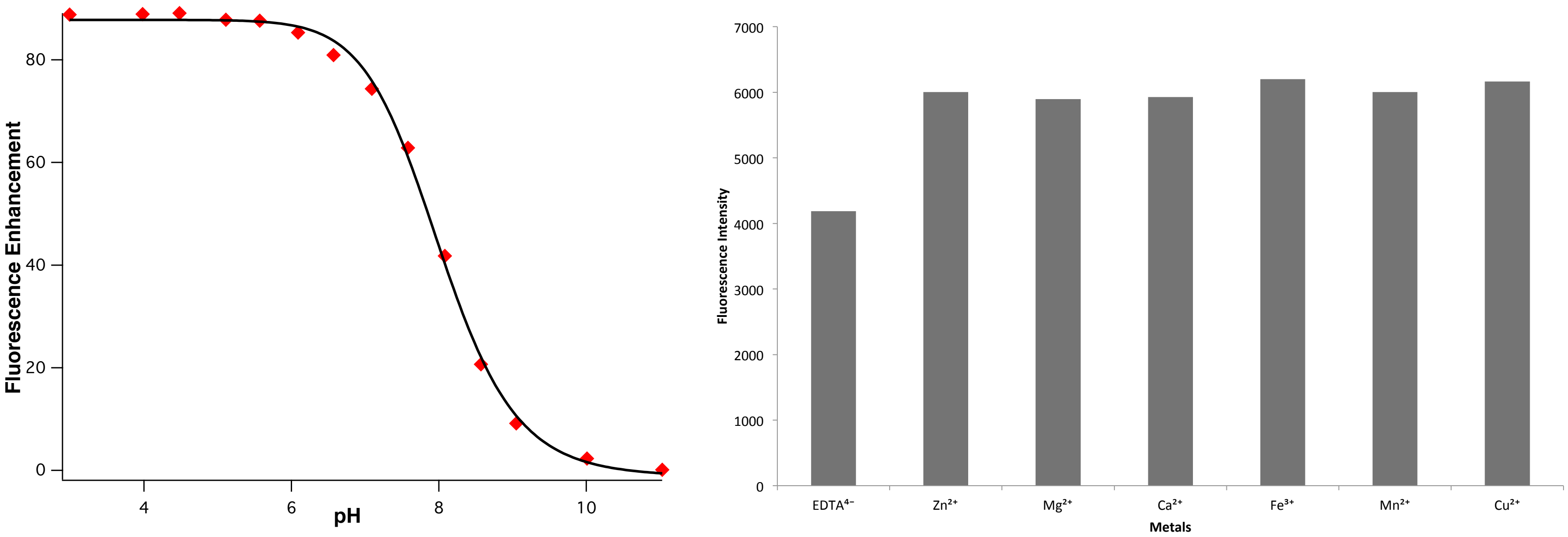

Left: Dependence of fluorescence enhancement on pH. Curve fitting was based on a modified Hill equation from which pK_a values were calculated. Right: Fluorescence intensities of the probe (5 μM) to a range of metal ions in a MOPS buffer (MOPS 30 mM, KCl 100 mM, pH 7.2). The concentration of cations is 10^{-3} M.

HR-LysA

State	λ_{abs} (nm)	λ_{em} (nm)	pK$_a$	ε (M^{-1} cm^{-1})	Φ	Φ_{ON}/Φ_{OFF}	Dynamic Intensity
ON[a]	577	602	7.51 ± 0.04	57000	0.25	13	13
OFF[b]	572	596		93000	0.02		

[a] Protonated form: pH 4

[b] Deprotonated form: pH 10

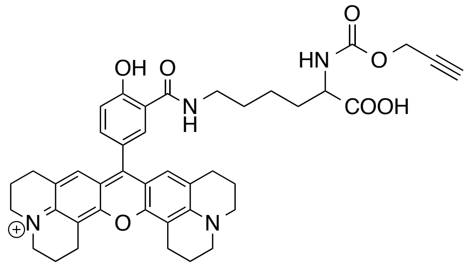

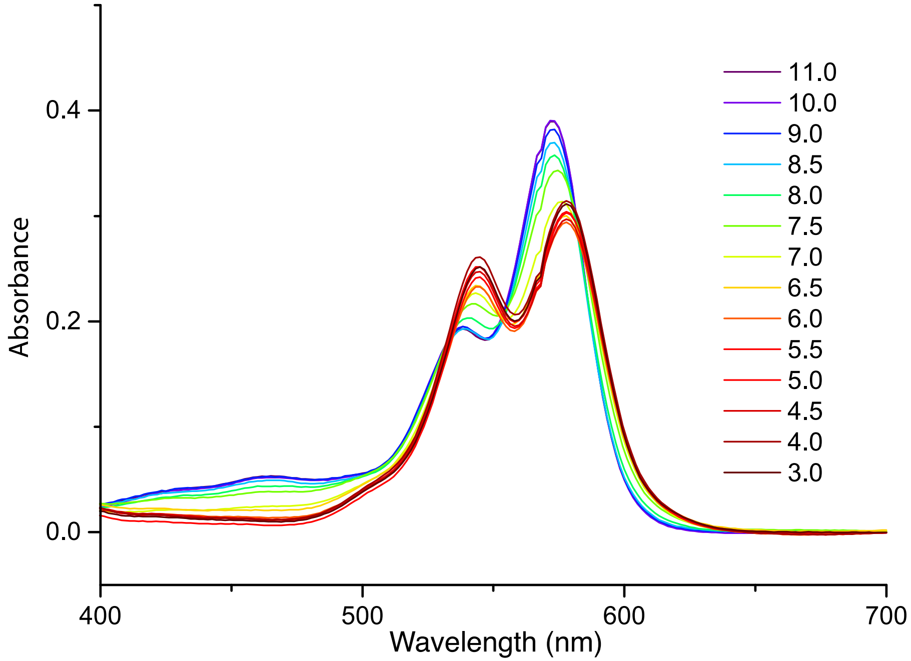

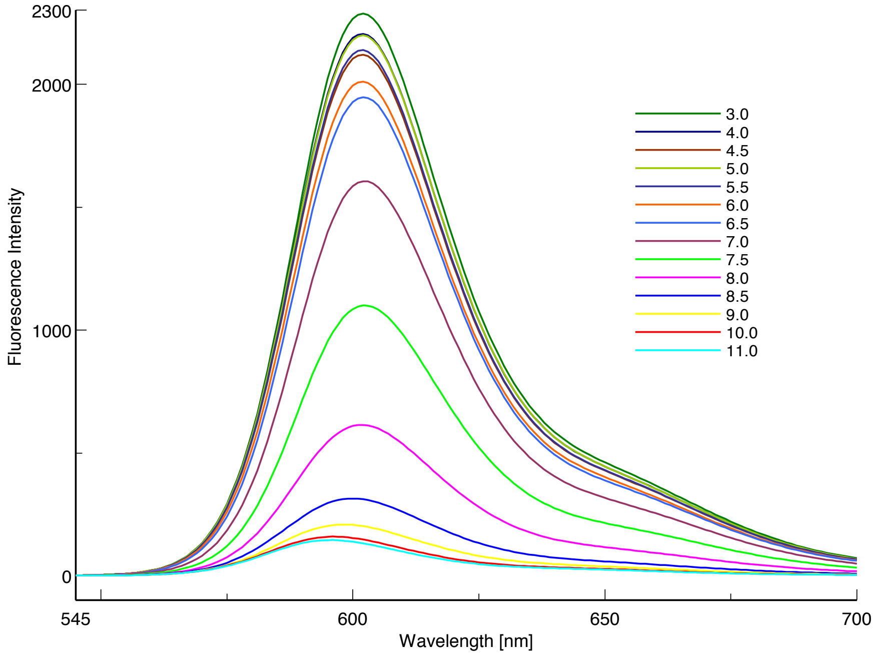

Left: Dependence of absorption on pH in a MOPS buffered aqueous solution. Right: Dependence of fluorescence intensity on pH in a MOPS buffered aqueous solution. Excitation at 535 nm.

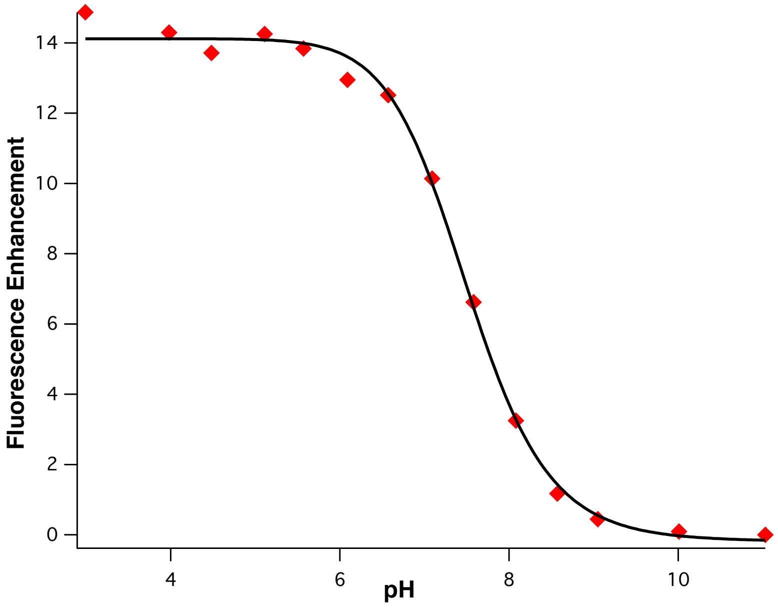

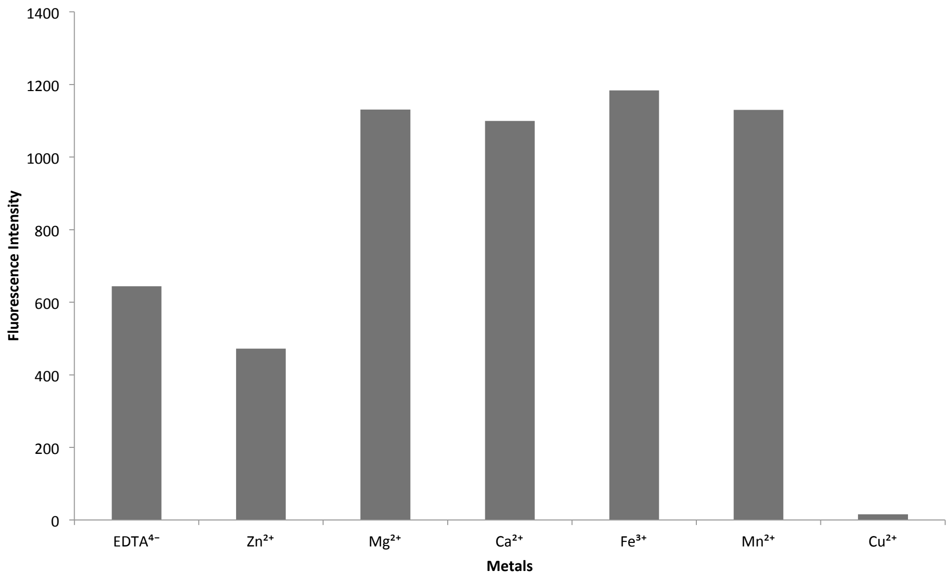

Left: Dependence of fluorescence enhancement on pH. Curve fitting was based on a modified Hill equation from which pK_a values were calculated. Right: Fluorescence intensities of the probe (5 μM) to a range of metal ions in a MOPS buffer (MOPS 30 mM, KCl 100 mM, pH 7.2). The concentration of cations is 10^{-3} M.

HR-PA

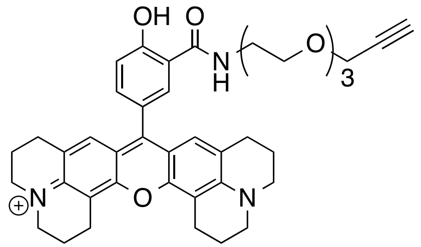

State	λ_{abs} (nm)	λ_{em} (nm)	pK$_a$	ε (M^{-1} cm^{-1})	Φ	Φ_{ON}/Φ_{OFF}	Dynamic Intensity
ON[a]	580	602	6.89 ± 0.04	75500	0.46	1150	31
OFF[b]	573	596		72500	0.0004		

[a] Protonated form: pH 4

[b] Deprotonated form: pH 11

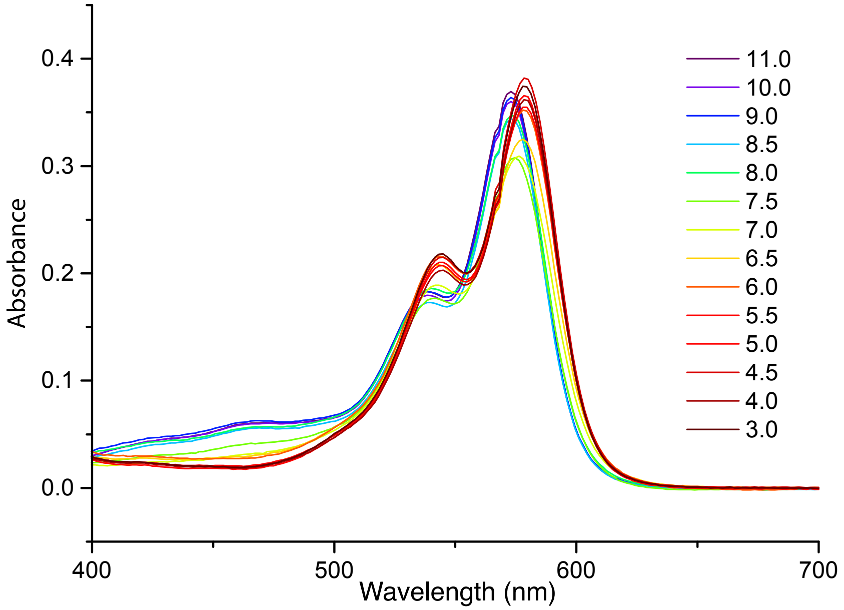

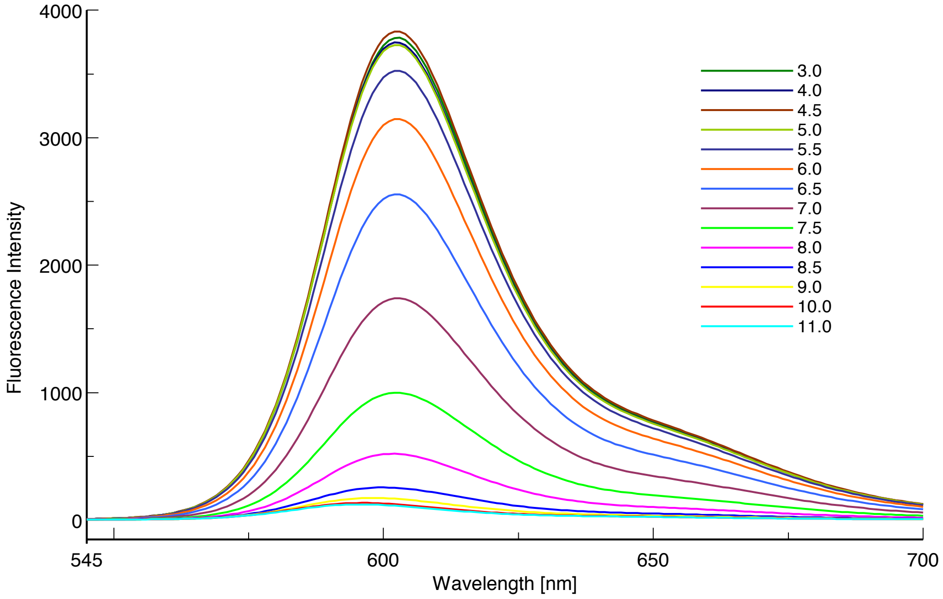

Left: Dependence of absorption on pH in a MOPS buffered aqueous solution. Right: Dependence of fluorescence intensity on pH in a MOPS buffered aqueous solution. Excitation at 535 nm.

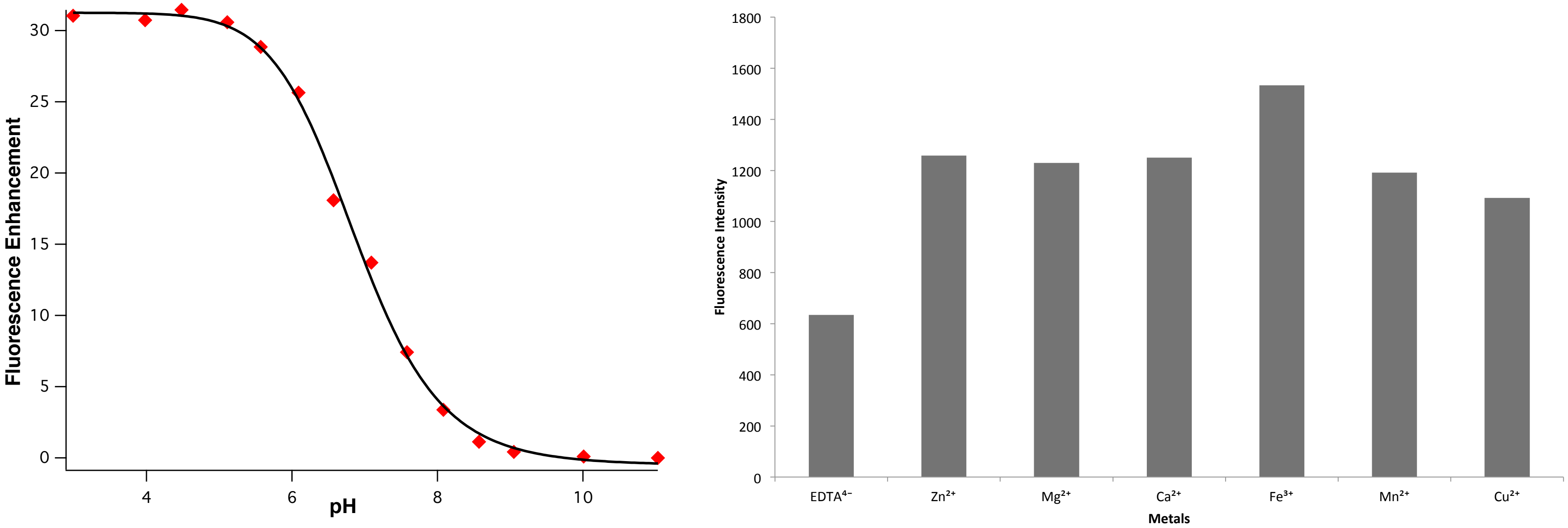

Left: Dependence of fluorescence enhancement on pH. Curve fitting was based on a modified Hill equation from which pK_a values were calculated. Right: Fluorescence intensities of the probe (5 μM) to a range of metal ions in a MOPS buffer (MOPS 30 mM, KCl 100 mM, pH 7.2). The concentration of cations is 10^{-3} M.

HR-LysF

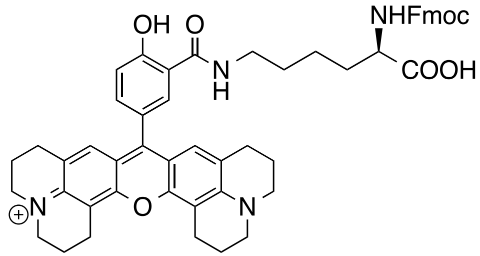

State	λ_{abs} (nm)	λ_{em} (nm)	pK$_a$	ε (M^{-1} cm^{-1})	Φ	Φ_{ON}/Φ_{OFF}	Dynamic Intensity
ON[a]	583	607	4.84 ± 0.10	54500	0.11	37	45
OFF[b]	580	600		46000	0.003		

[a] Protonated form: pH 2

[b] Deprotonated form: pH 8

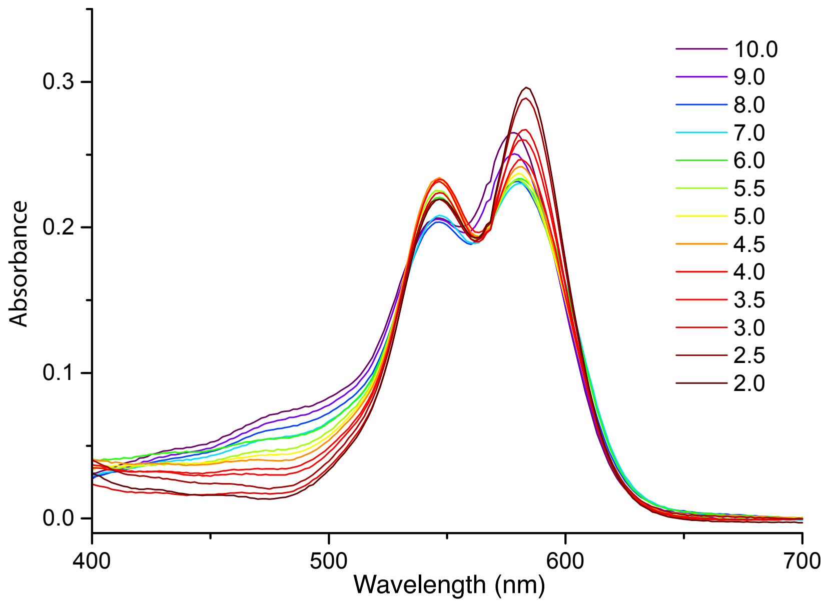

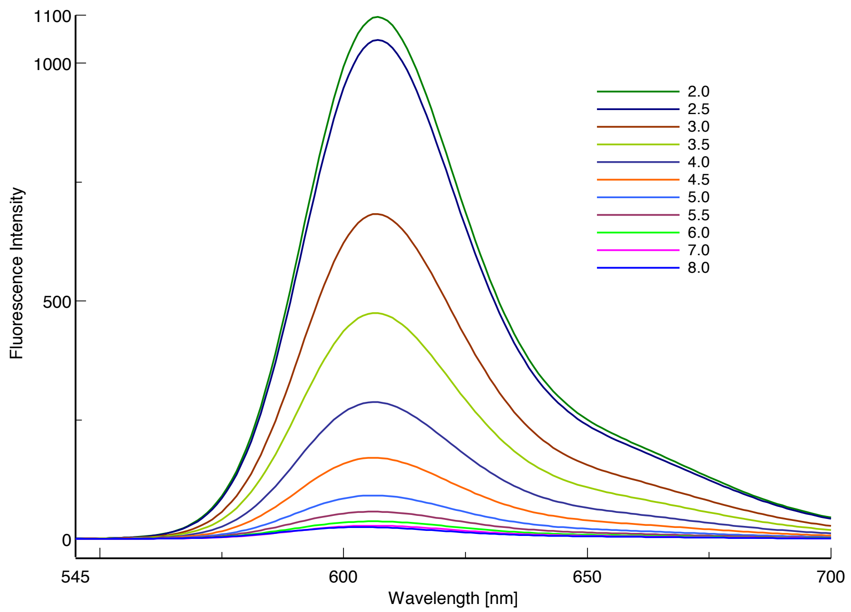

Left: Dependence of absorption on pH in a MOPS buffered aqueous solution. Right: Dependence of fluorescence intensity on pH in a MOPS buffered aqueous solution. Excitation at 535 nm.

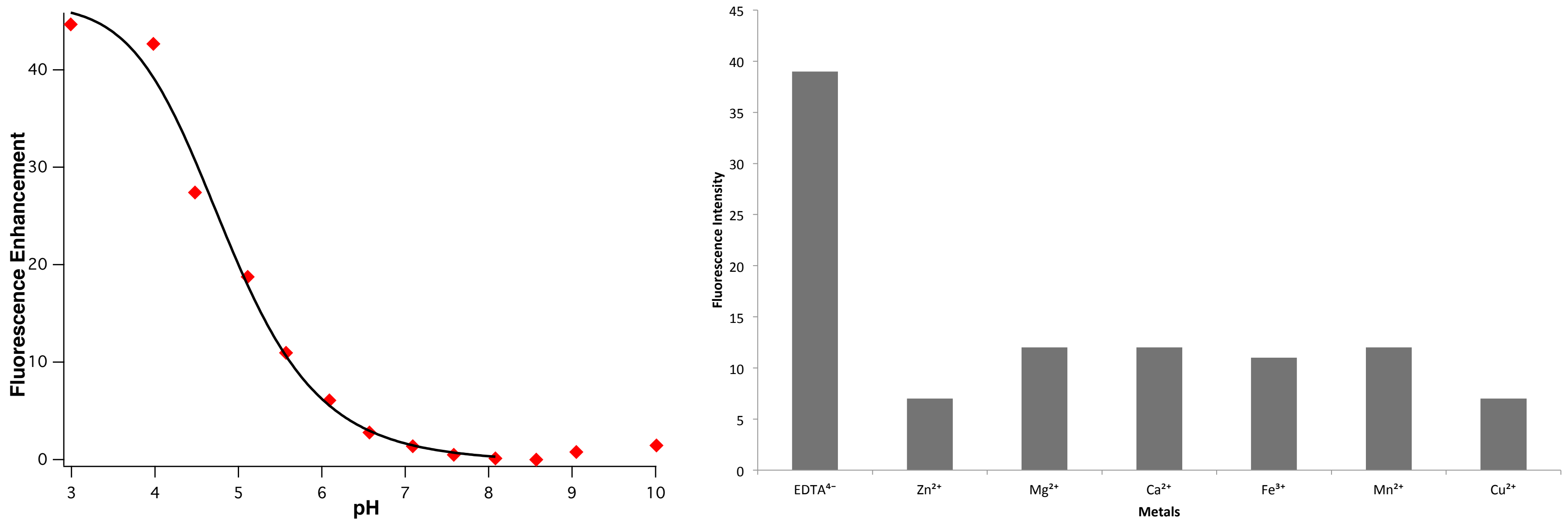

Left: Dependence of fluorescence enhancement on pH. Curve fitting was based on a modified Hill equation from which pK_a values were calculated. Right: Fluorescence intensities of the probe (5 μM) to a range of metal ions in a MOPS buffer (MOPS 30 mM, KCl 100 mM, pH 7.2). The concentration of cations is 10^{-3} M.

HR-PN$_3$ Dextran 40,000 conjugate

State	λ_{abs} (nm)	λ_{em} (nm)	pK$_a$	ε (M^{-1} cm^{-1})	Φ	Φ_{ON}/Φ_{OFF}	Dynamic Intensity
ON[a]	583	603	6.77 ± 0.02	–	0.167 ± 0.021	19	17
OFF[b]	573	597		–	0.009 ± 0.00001		

[a] Protonated form: pH 4

[b] Deprotonated form: pH 10

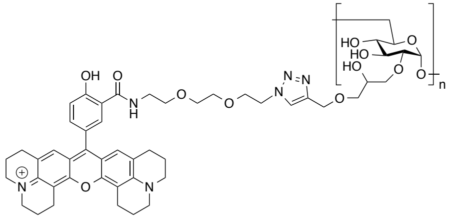

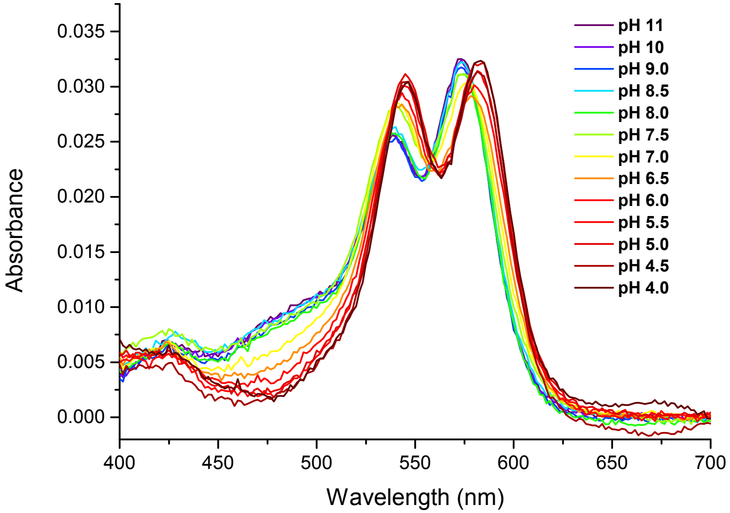

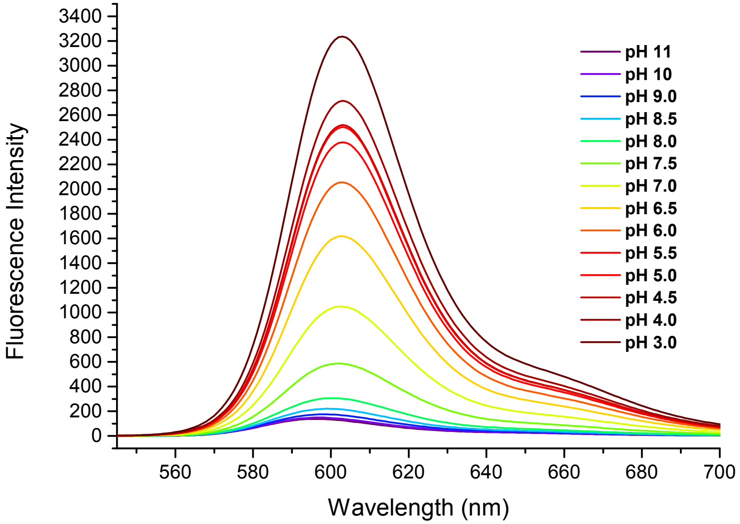

Left: Dependence of absorption on pH in a MOPS buffered aqueous solution. Right: Dependence of fluorescence intensity on pH in a MOPS buffered aqueous solution. Excitation at 535 nm.

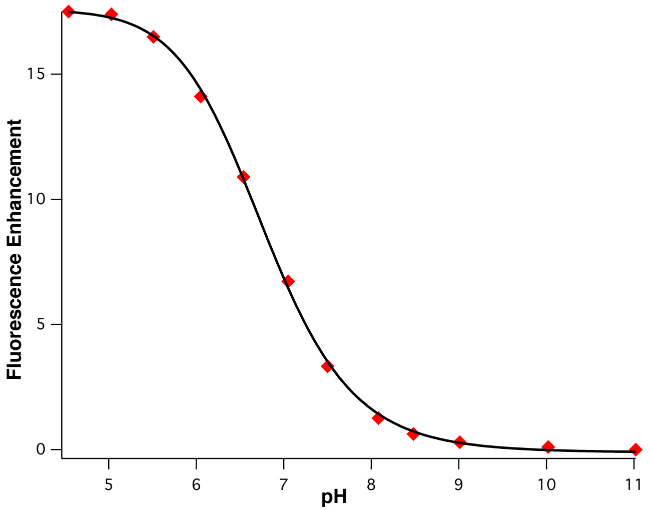

Dependence of fluorescence enhancement on pH. Curve fitting was based on a modified Hill equation from which pK_a values were calculated.
