# Supplementary material for: H-Rubies, a new family of red emitting fluorescent pH sensors for living cells
Source: Chem Sci. 2015 Jul 14;6(10):5928–37. doi: 10.1039/c5sc01113b (PMC5950754; doi:10.1039/c5sc01113b)
Supplement: Supplementary file 3 [file SC-006-C5SC01113B-s003.pdf]

Electronic Supplementary Material (ESI) for Chemical Science.
This journal is © The Royal Society of Chemistry 2015

H-Rubies, a New Family of Red Emitting Fluorescent pH sensors for Living Cells

Guillaume Despras[+], Alsu Zamaleeva[+], Lucie Dradevet, Céline Tisseyre, Joao Gamelas Magalhaes, Charlotte Garner, Michel De Waard, Sebastian Amigorena, Anne Feltz, Jean-Maurice Mallet, Mayeul Collot*

Supplementary information

NMR and Mass spectra

NMR and mass spectra of non phenolic X-rhodamines and intermediates
HRMS of the first set of H-Rubies
NMR spectra of functionalisable H-Rubies and their intermediates
HRMS spectra of functionalisable H-Rubies and their intermediates

NMR and mass spectra of non phenolic X-rhodamines and intermediates

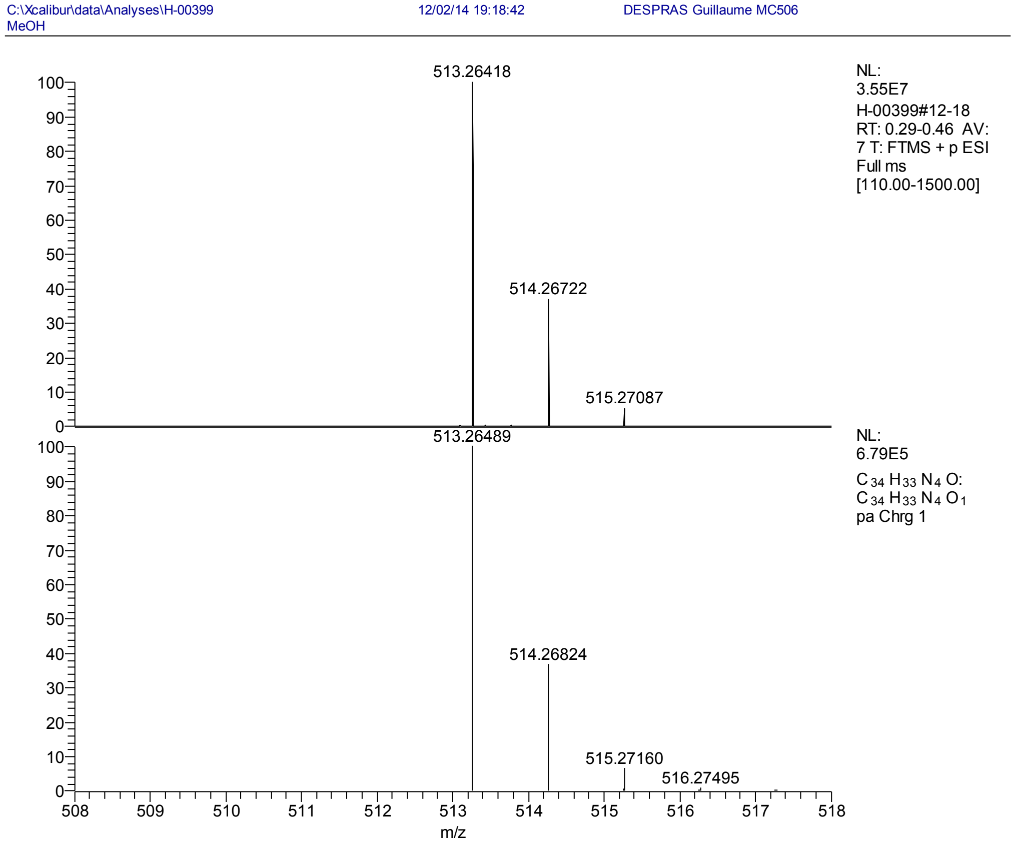

HRMS Spectrum of **Imidazole Based X-Rhodamine**

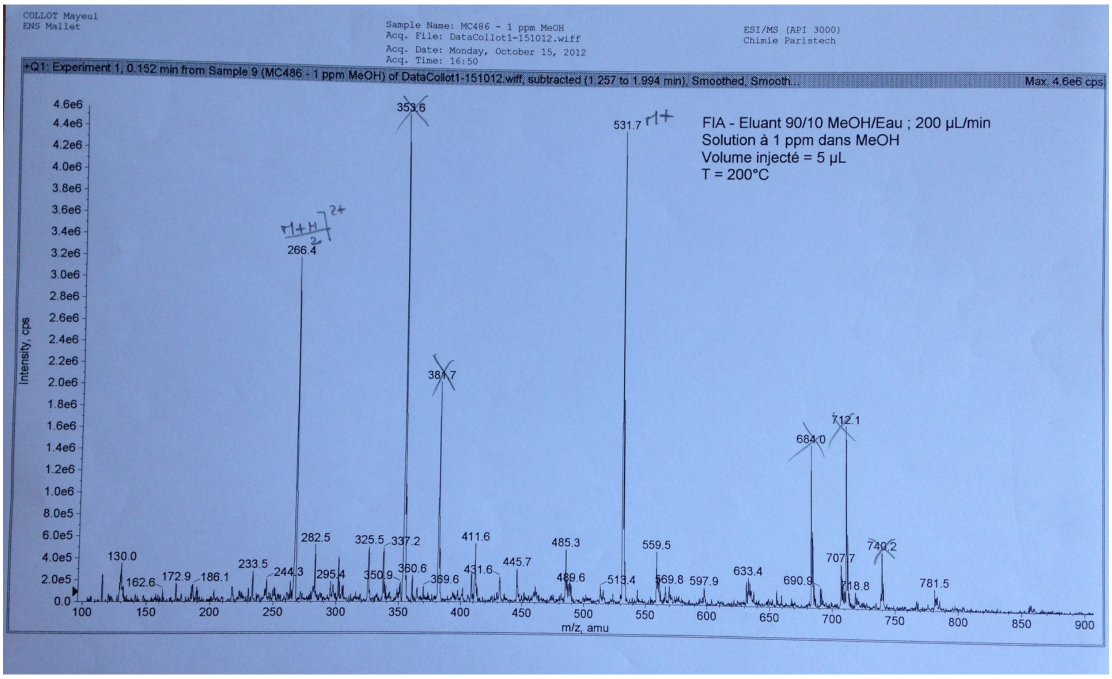

MS Spectrum of **Pip-H**

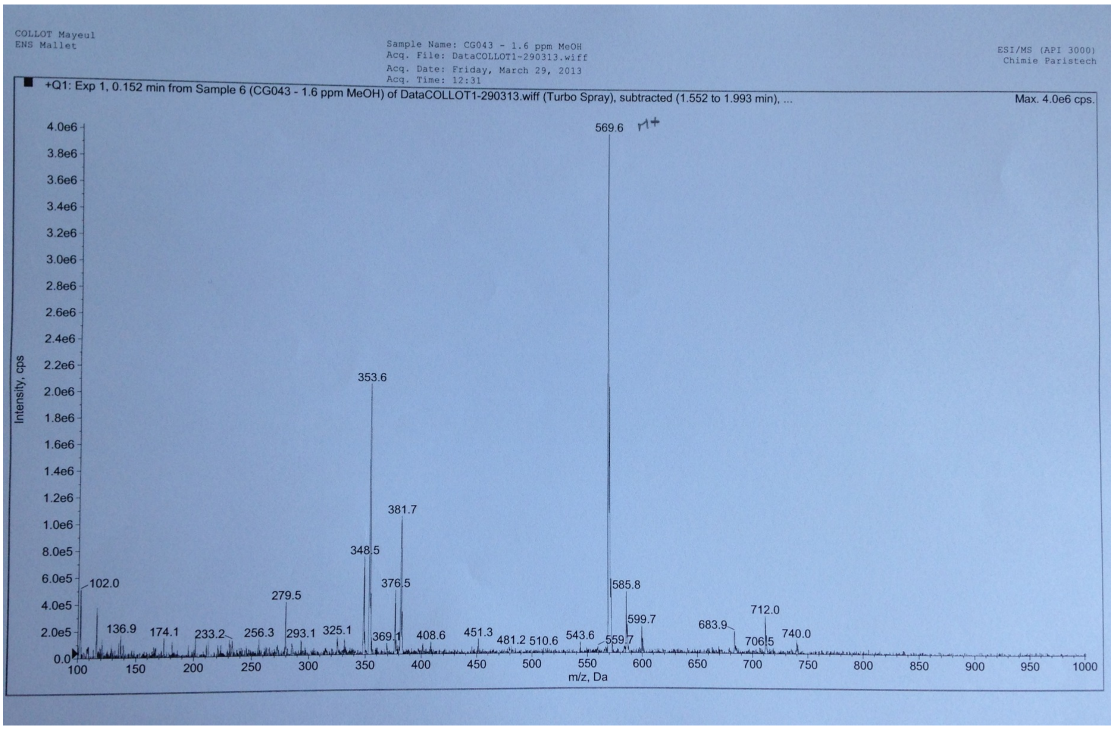

MS Spectrum of **Pip-Alkyne**

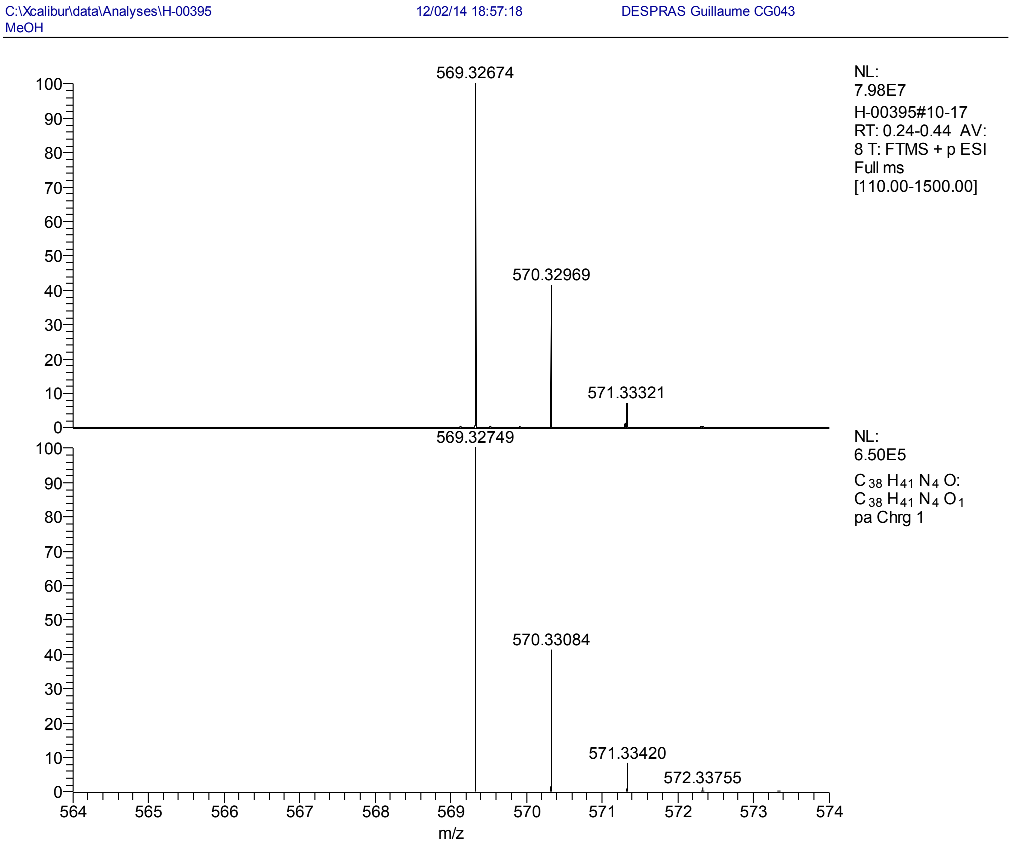

HRMS Spectrum of **Pip-Alkyne**

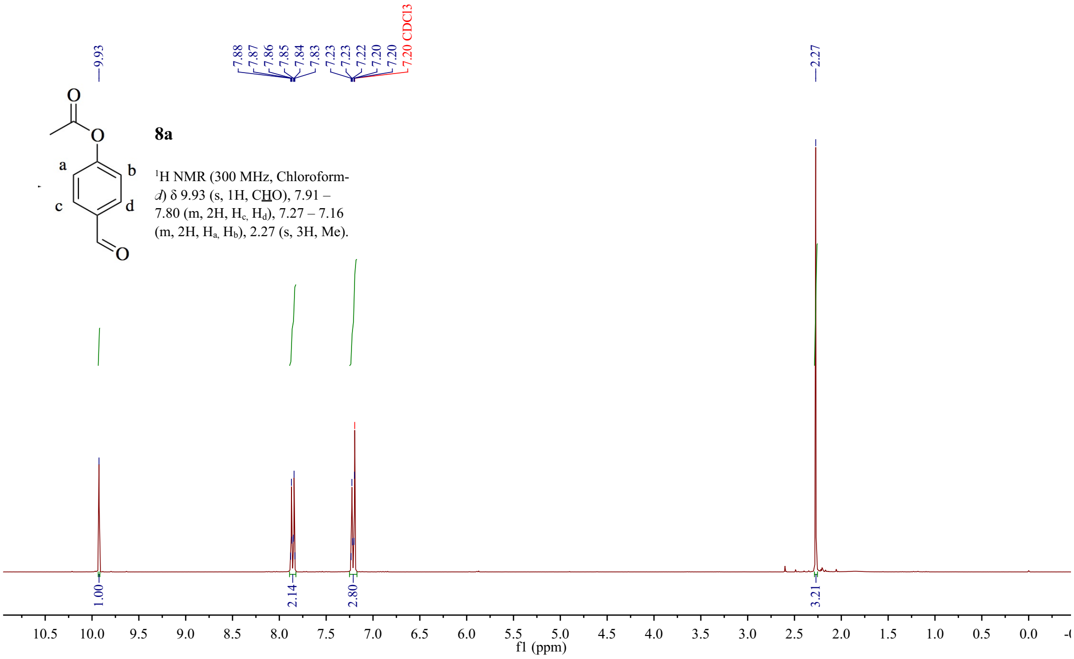

^{1}H NMR spectrum of **4-formylphenyl acetate**

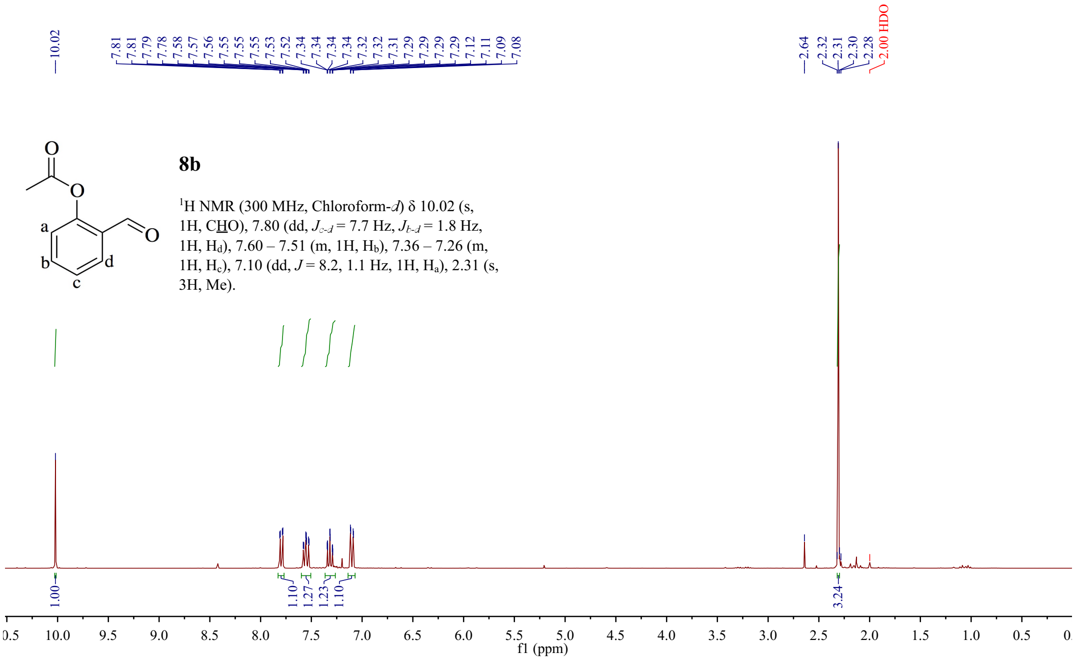

^{1}H NMR spectrum of **2-formylphenyl acetate**

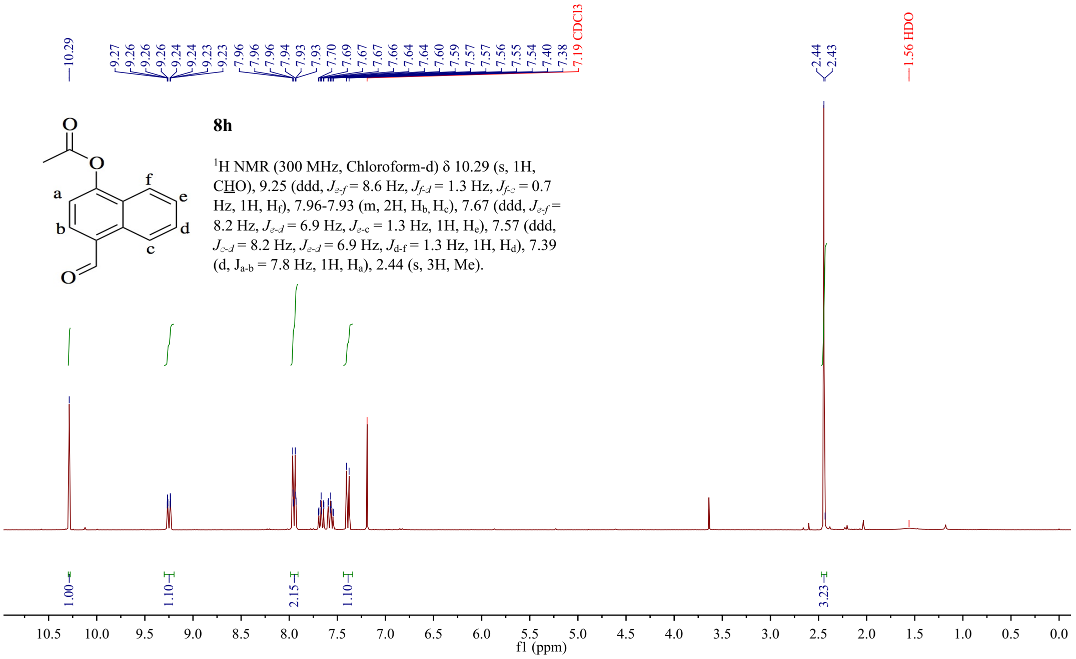

^{1}H NMR spectrum of **4-formylnaphthalen-1-yl acetate**

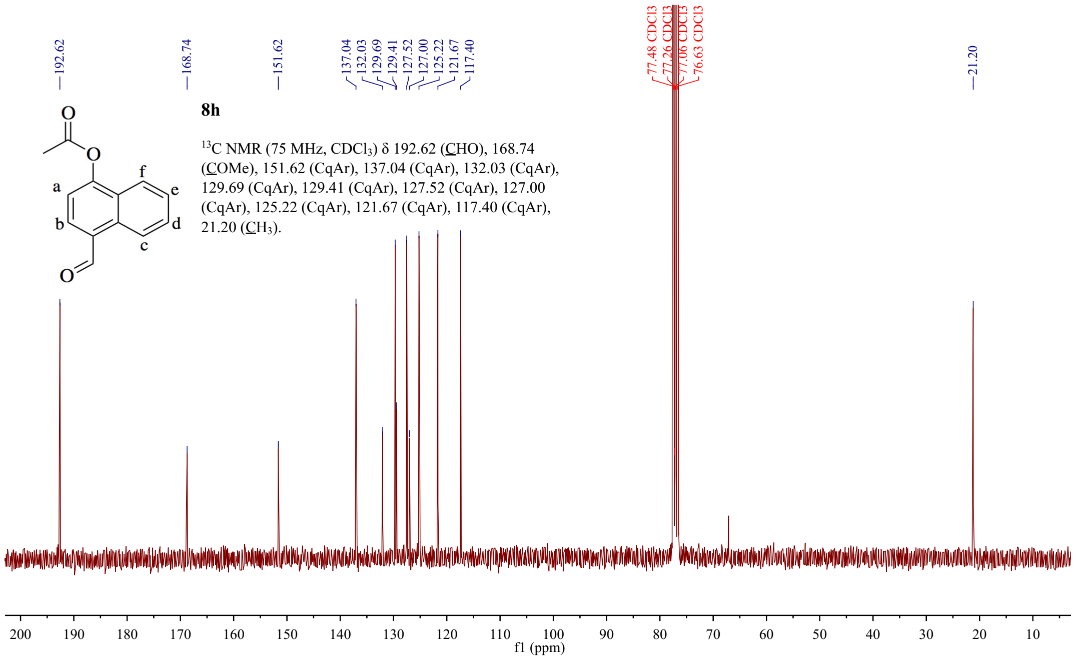

^{13}C NMR spectrum of **4-formylnaphthalen-1-yl acetate**

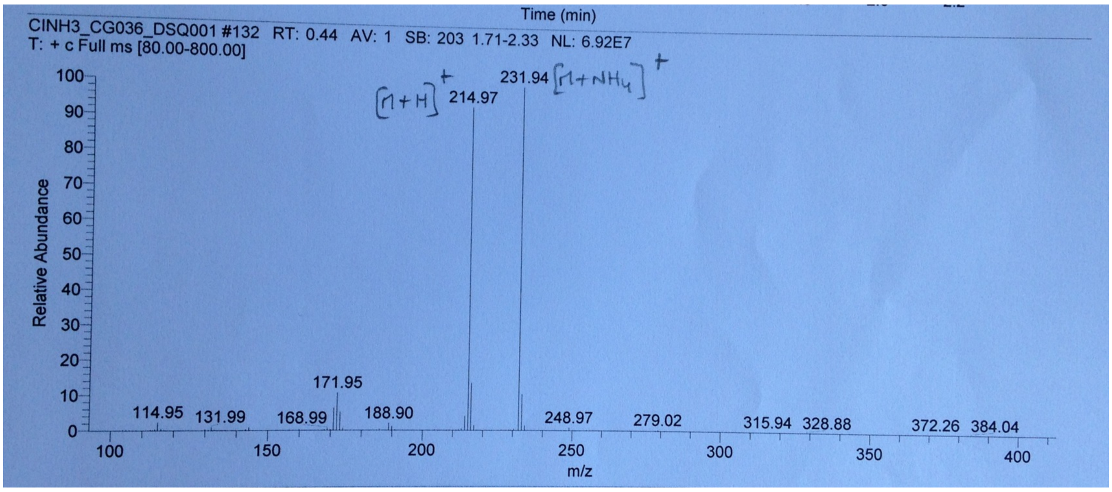

MS Spectrum of **4-formylnaphthalen-1-yl acetate**

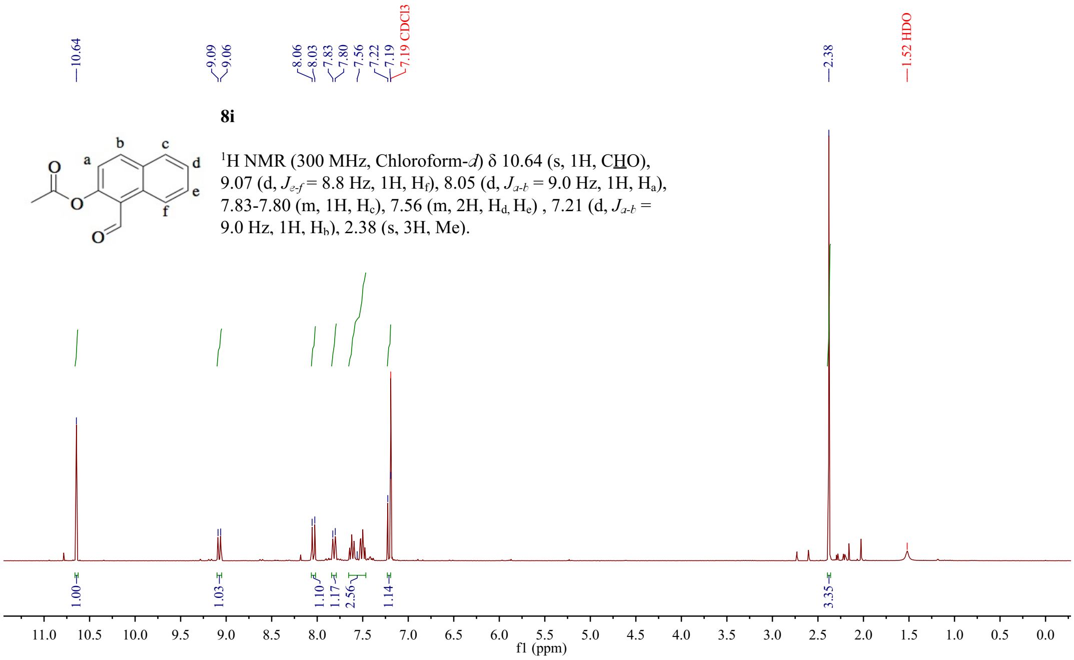

^{1}H NMR spectrum of **1-formylnaphthalen-2-yl acetate**

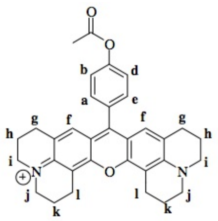

9a

^{1}H NMR (300 MHz, Chloroform-*d*) δ 7.80 – 7.69 (m, 2H, H_a, H_e), 7.27 (s, 4H, Hpy_{PTSA}), 7.01 – 6.92 (m, 2H, H_b, H_d), 6.73 (s, 2H, H_f), 3.47 (m, 8H, H_i, H_j), 2.95 (t, *J* = 6.4 Hz, 4H, H_l), 2.63 (t, *J* = 6.2 Hz, 4H, H_g), 2.33 (s, 3H, Me), 2.18 (s, 3H, Me $_{PTSA}$), 2.02 (m, 4H, H_k), 1.90 (m, 4H, H_h).

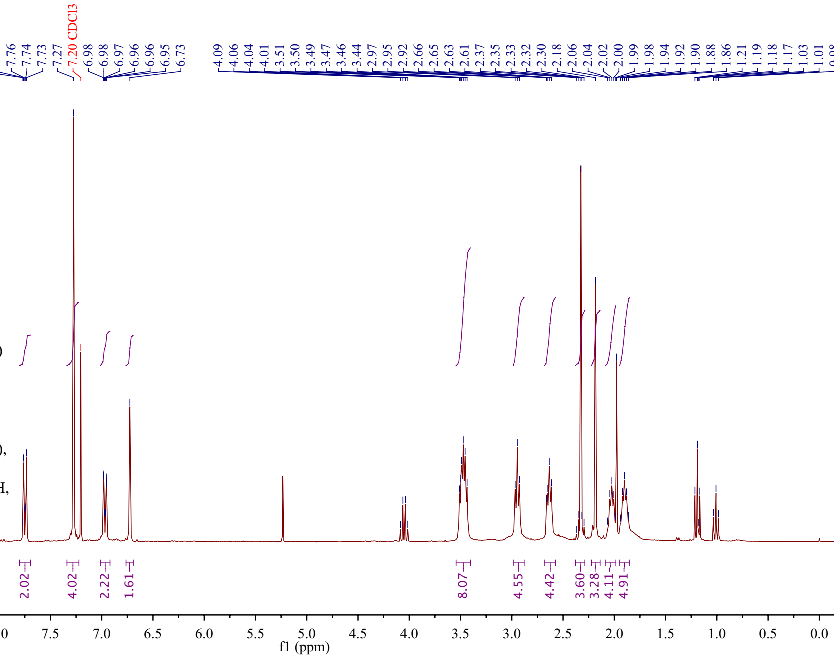

^{1}H NMR spectrum of **Acetylated HR-*p*OH**

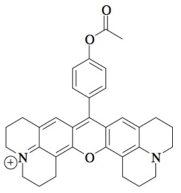

9a

^{13}C NMR (75 MHz, $CDCl_3$) δ 169.31 (COMe), 153.23 (C Ar), 152.11 (C Ar), 151.66 (C Ar), 151.17 (C Ar), 144.53 (C Ar), 138.20 (C Ar), 130.66 (C_b or C_d), 130.13 (C Ar), 128.16 (C_b or C_d), 126.42 (C Ar), 126.27 (C Ar), 123.81 (C_a or C_e), 122.15 (C_a or C_e), 112.76 (C Ar), 105.50 (C Ar), 50.97 (C_i or C_j), 50.51 (C_i or C_j), 27.68 (C_g) , 21.30 (CH_3), 20.65 (C_h), 19.92 (C_l), 19.70 (C_k).

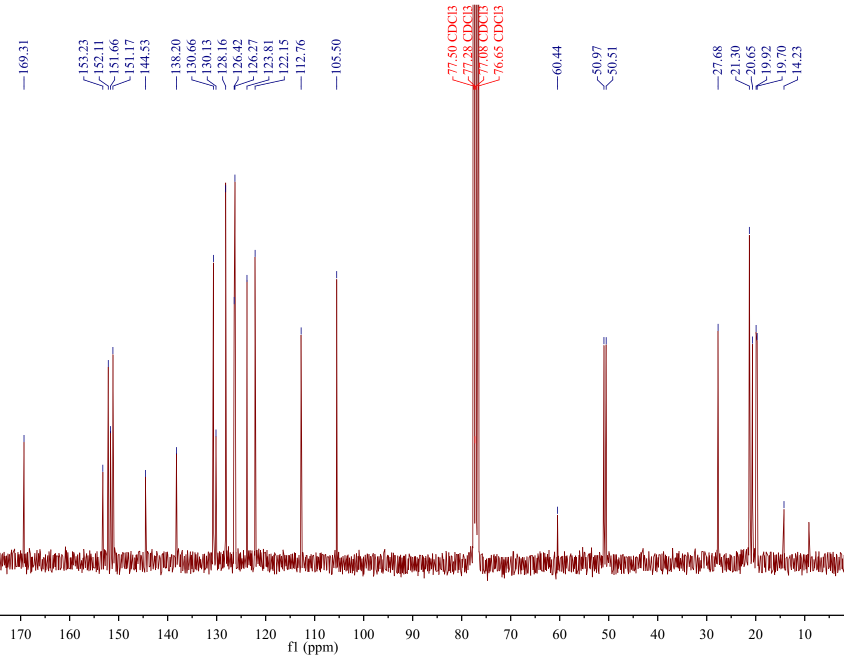

^{13}C NMR spectrum of **Acetylated HR-*p*OH**

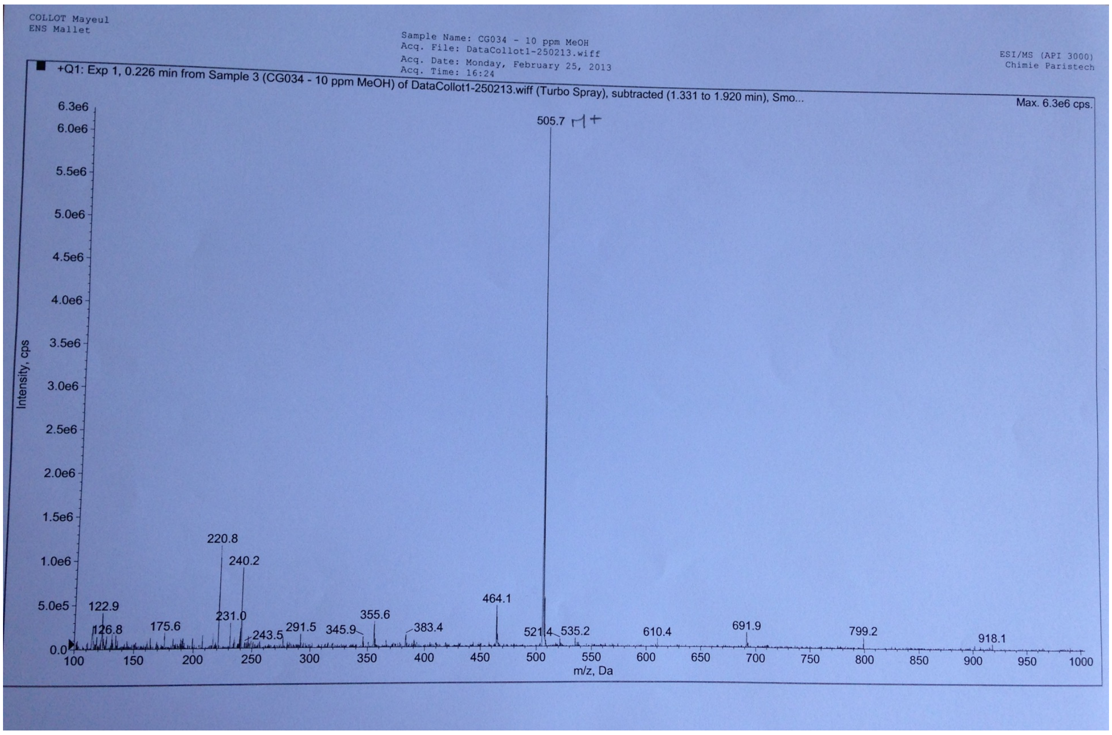

MS spectrum of **Acetylated HR-*p*OH**

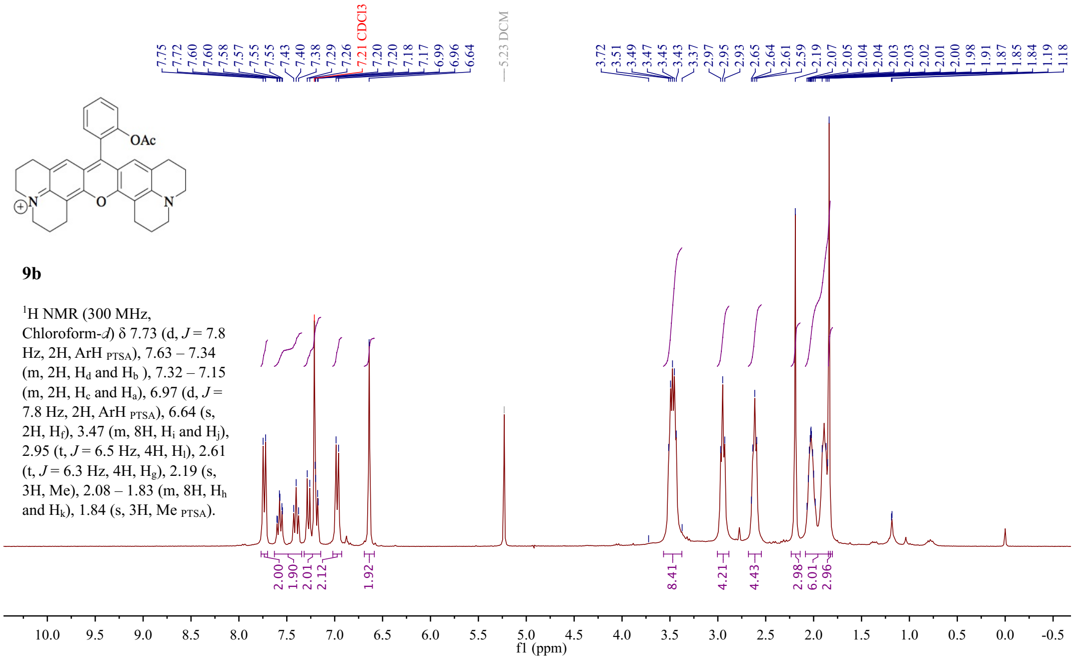

9b

^{1}H NMR (300 MHz, Chloroform-*d*) δ 7.73 (d, J = 7.8 Hz, 2H, ArH $_{PTSA}$), 7.63 – 7.34 (m, 2H, H_d and H_b), 7.32 – 7.15 (m, 2H, H_c and H_a), 6.97 (d, J = 7.8 Hz, 2H, ArH $_{PTSA}$), 6.64 (s, 2H, H_f), 3.47 (m, 8H, H_i and H_j), 2.95 (t, J = 6.5 Hz, 4H, H_l), 2.61 (t, J = 6.3 Hz, 4H, H_g), 2.19 (s, 3H, Me), 2.08 – 1.83 (m, 8H, H_h and H_k), 1.84 (s, 3H, Me $_{PTSA}$).

^{1}H NMR spectrum of **Acetylated HR-*o*OH**

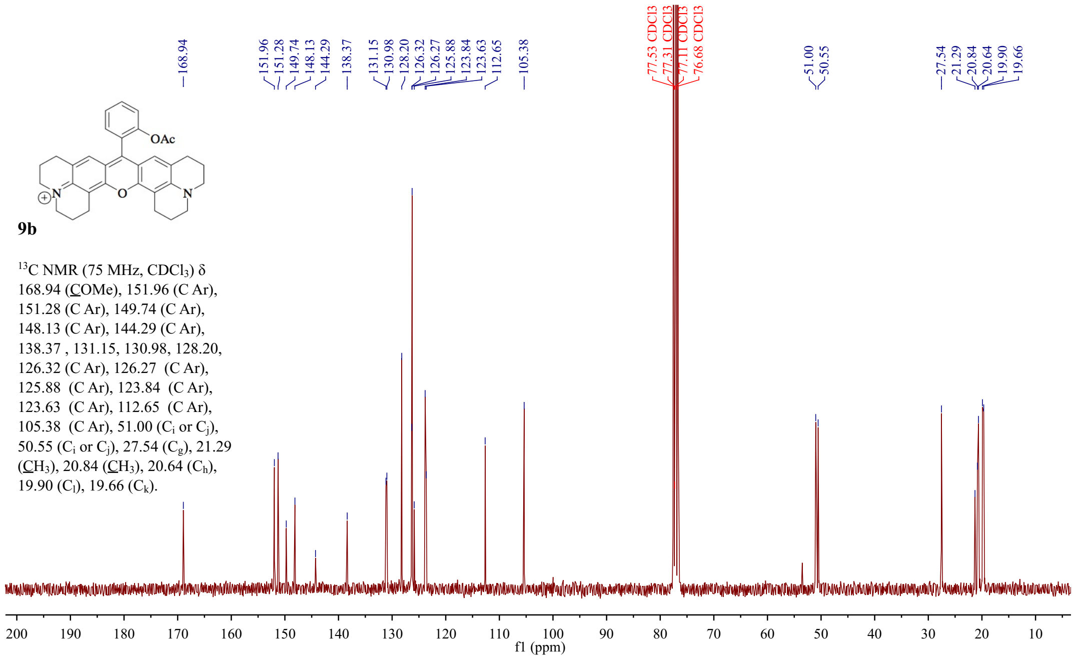

^{13}C NMR spectrum of **Acetylated HR-*o*OH**

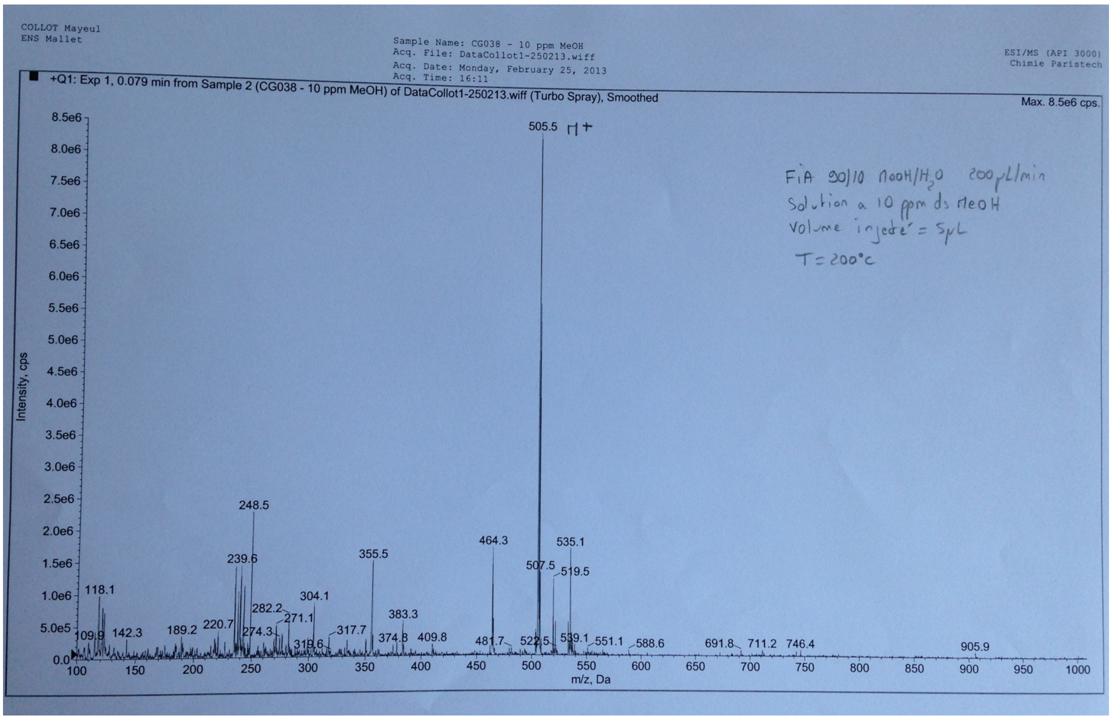

MS spectrum of **Acetylated HR-*o*OH**

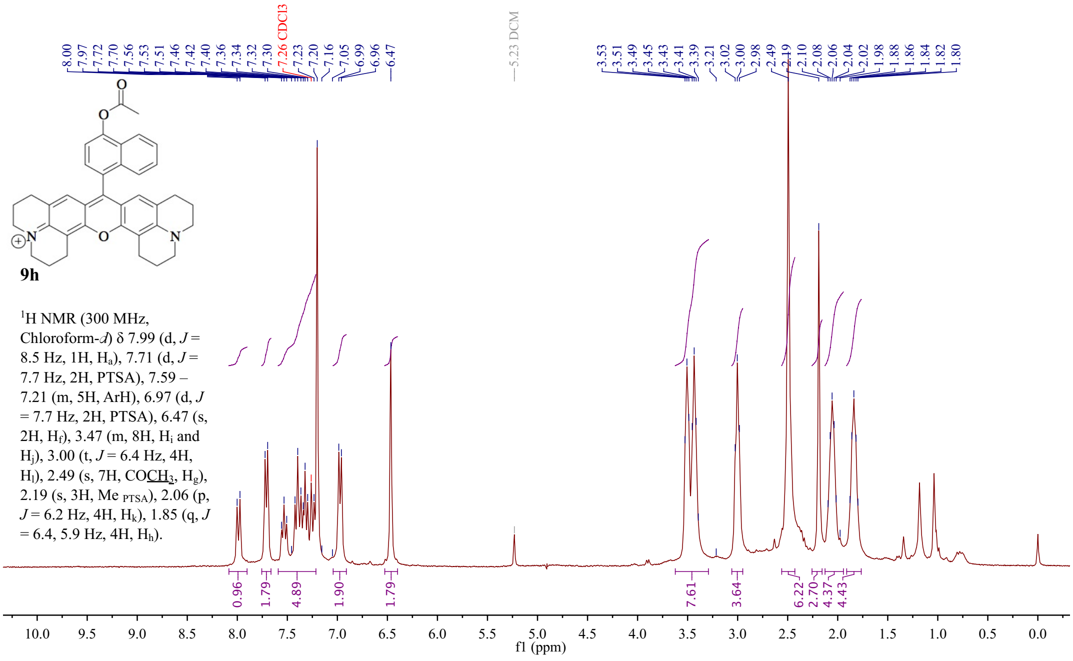

^{1}H NMR spectrum of **Acetylated *p*-Nph**

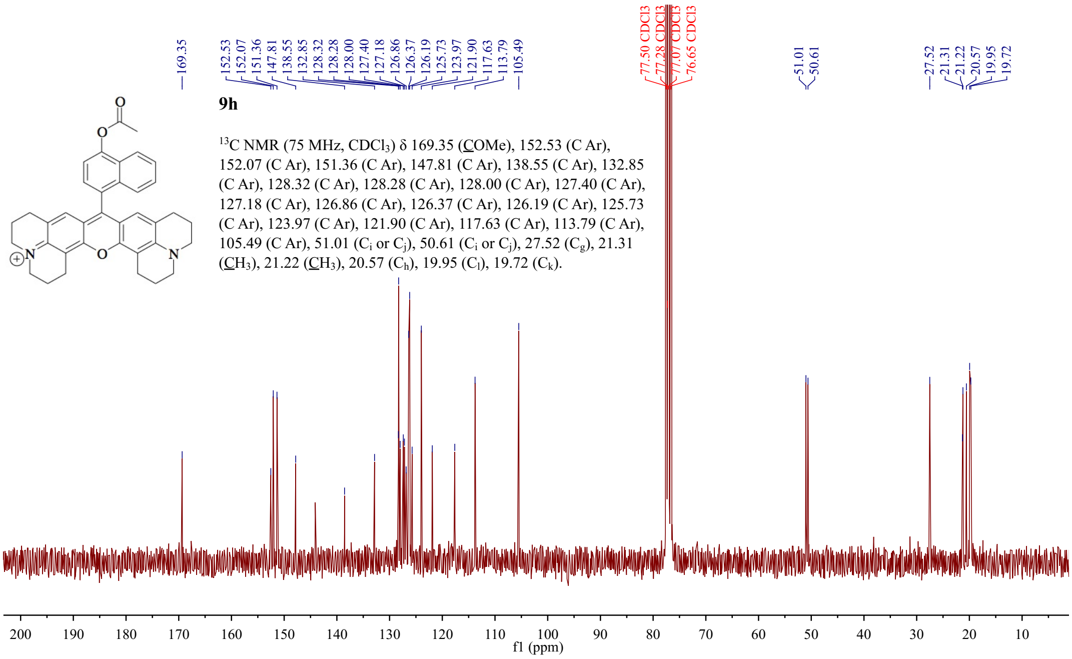

^{13}C NMR spectrum of **Acetylated *p*-Nph**

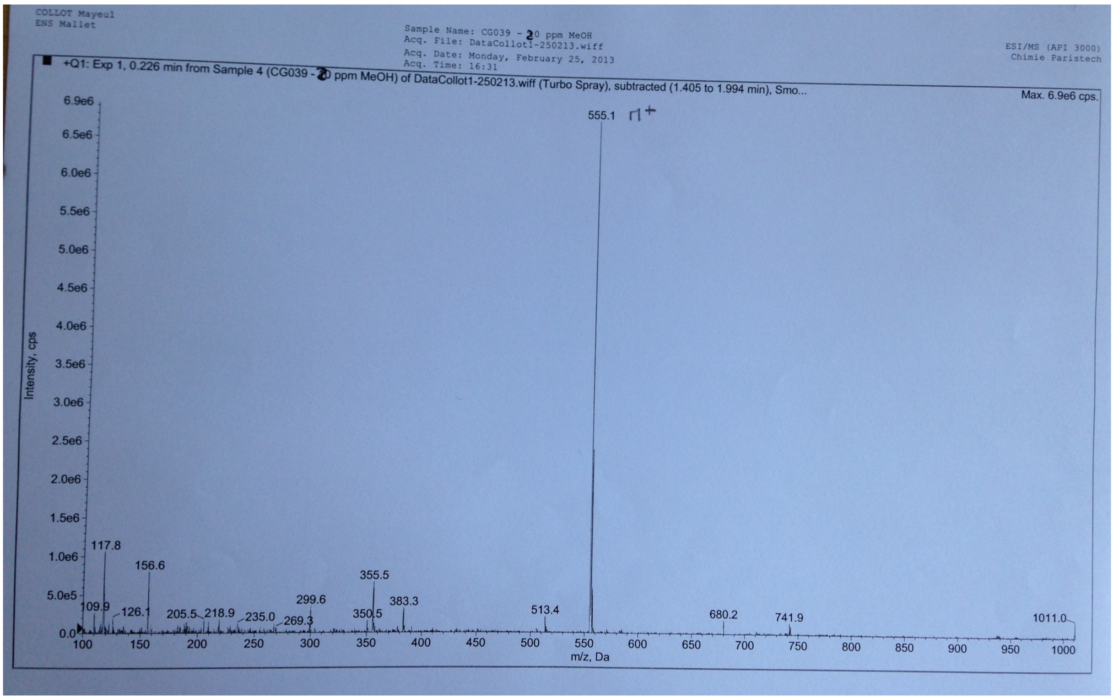

MS spectrum of **Acetylated *p*-Nph**

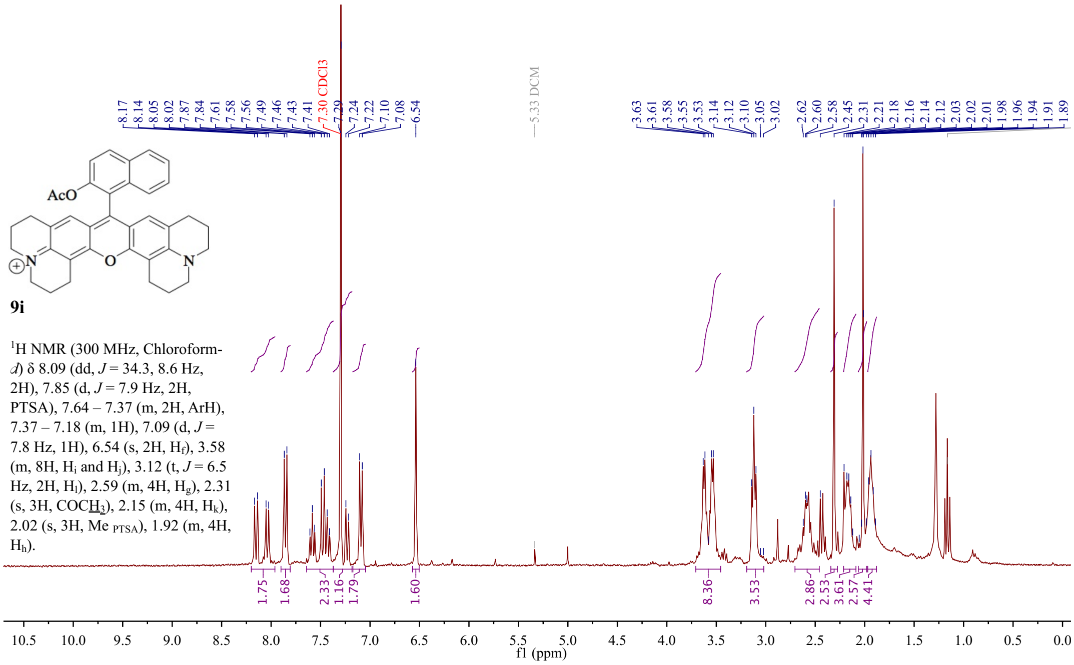

^{1}H NMR spectrum of **Acetylated *o*-Nph**

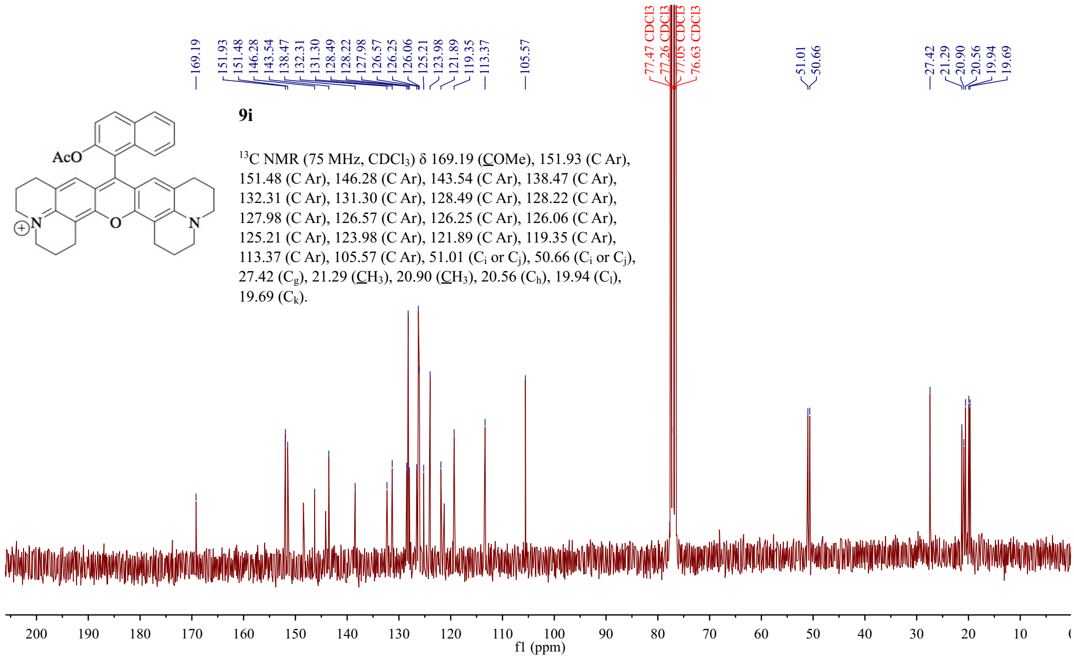

^{13}C NMR spectrum of **Acetylated *o*-Nph**

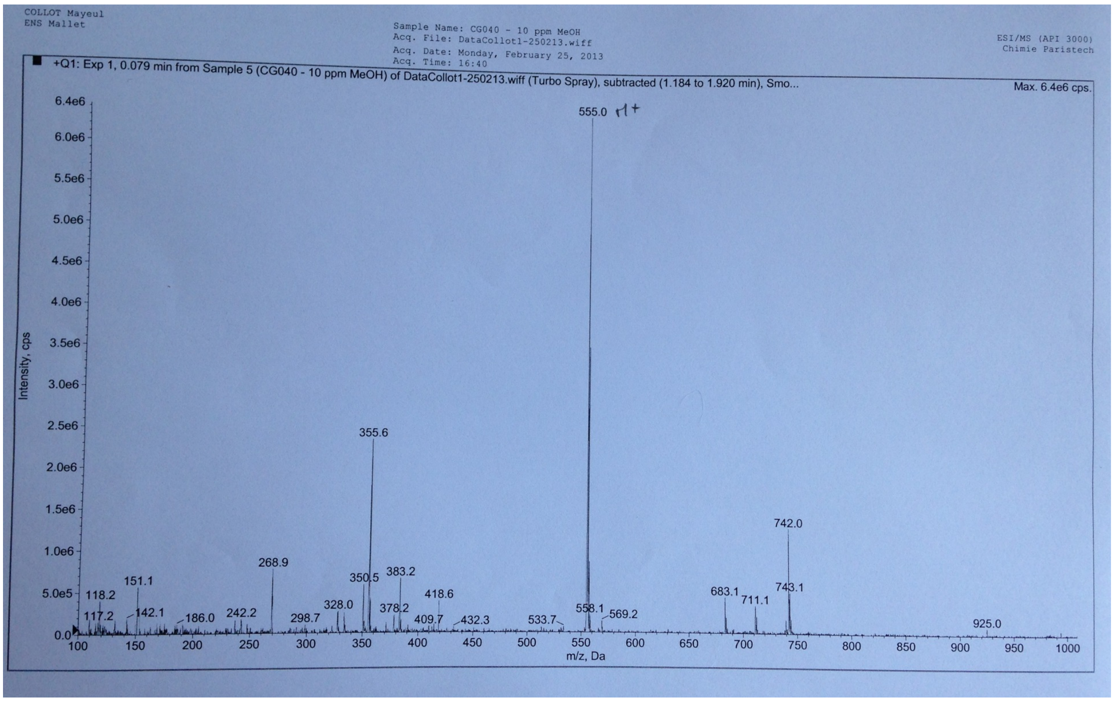

MS spectrum of **Acetylated *o*-Nph**

HRMS of the first set of H-Rubies

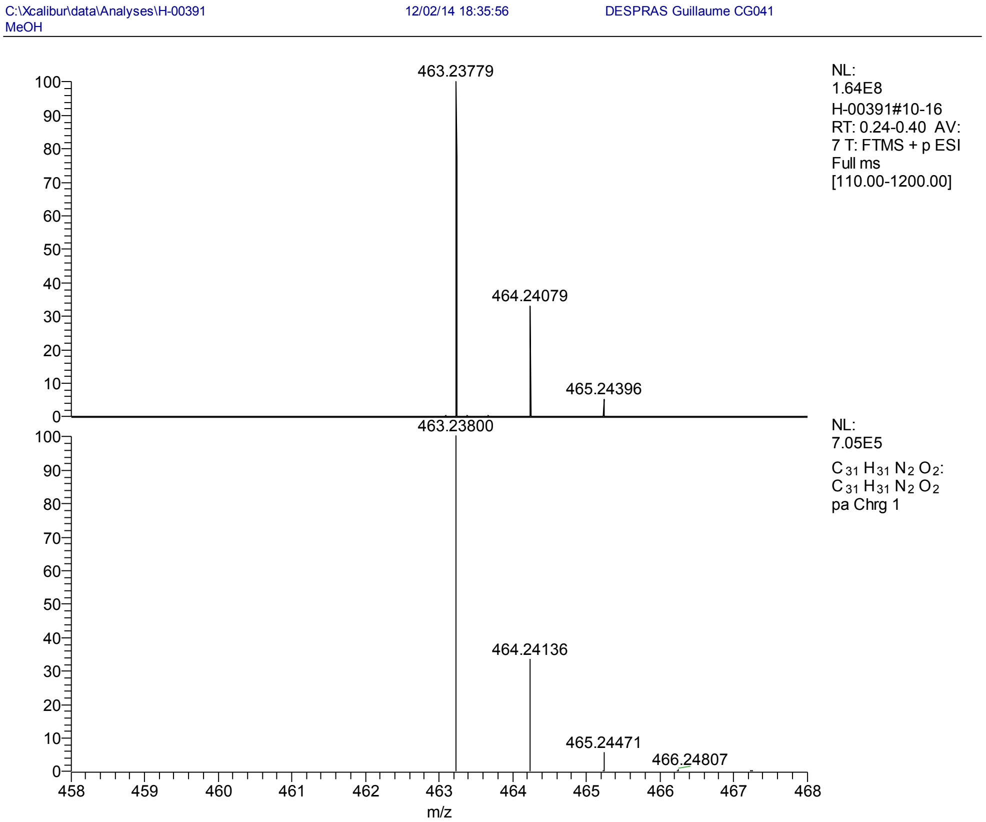

HRMS spectrum of **HR-*p*OH**

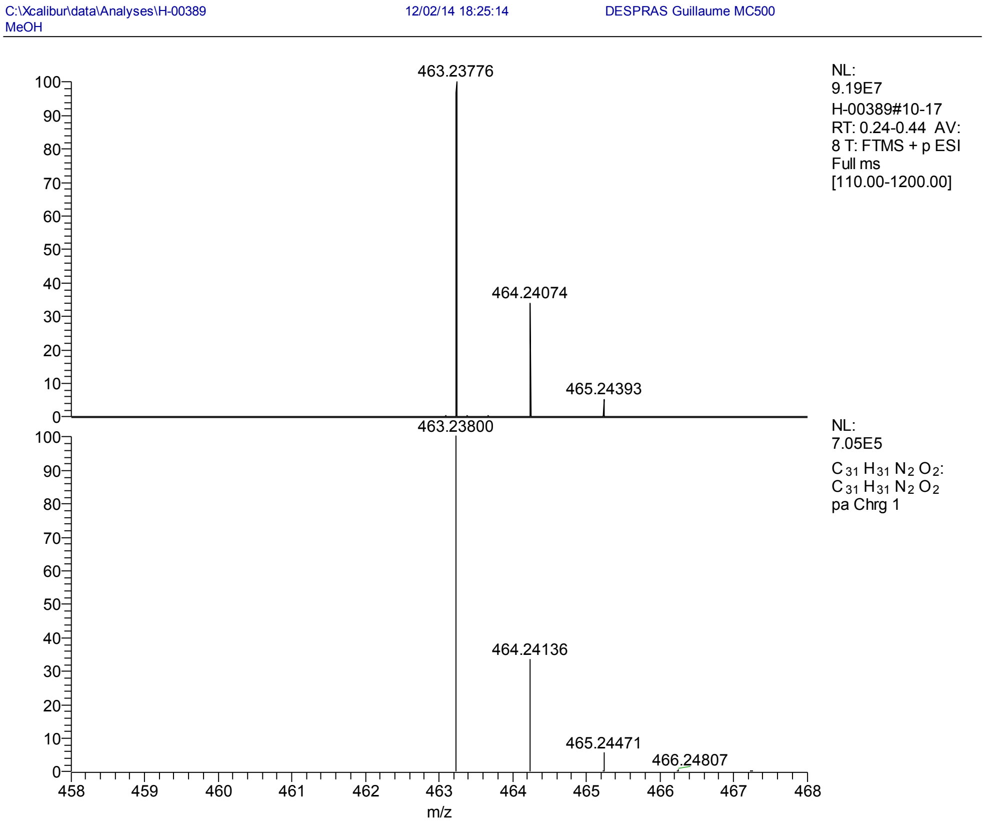

HRMS spectrum of **HR-*m*OH**

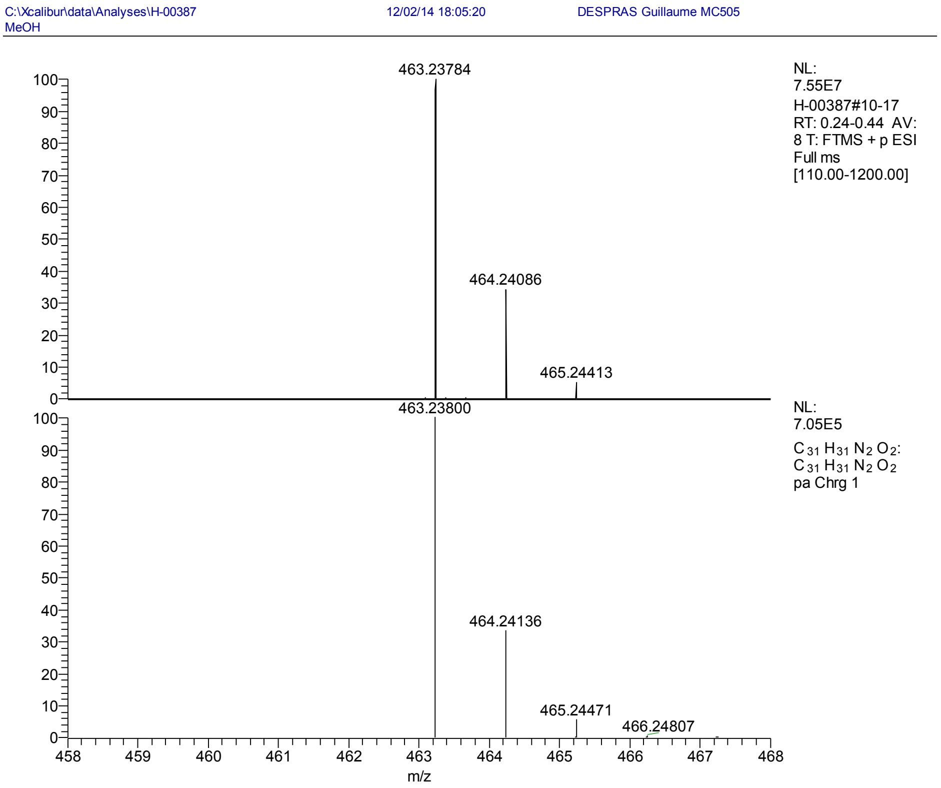

HRMS spectrum of **HR-*o*OH**

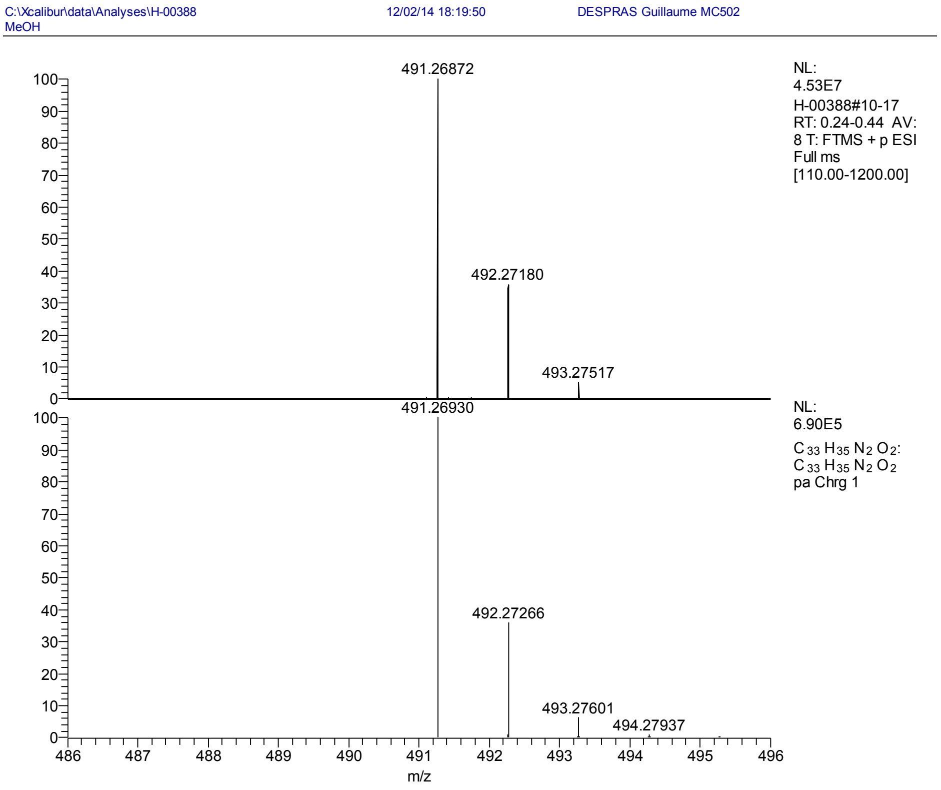

HRMS spectrum of **HR-Me**

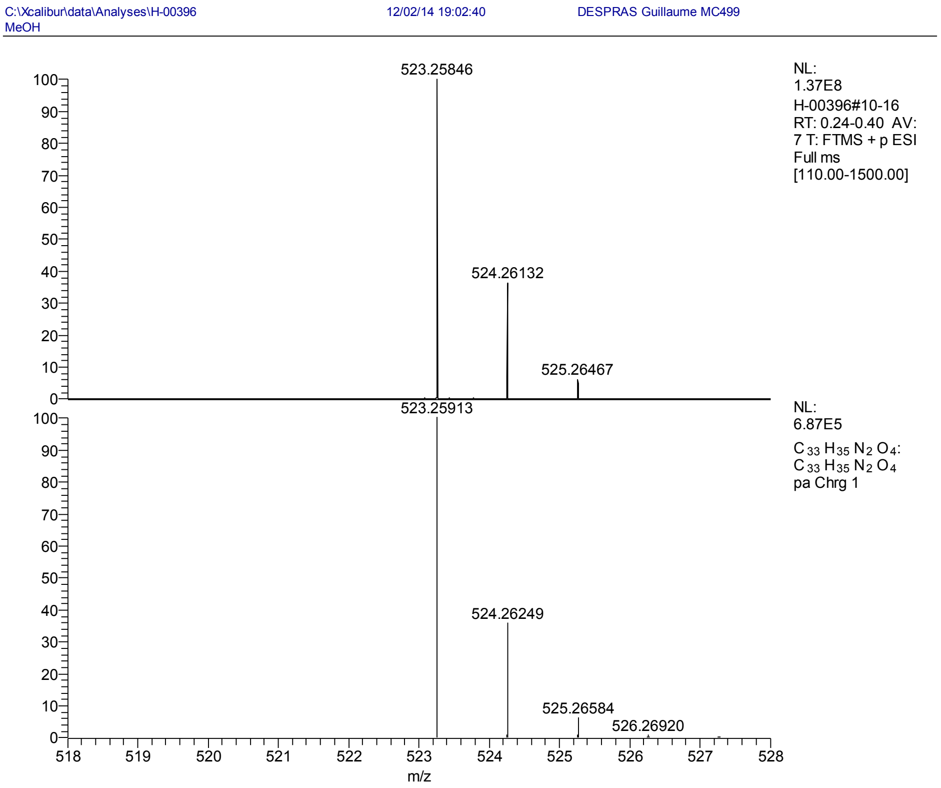

HRMS spectrum of **HR-OMe**

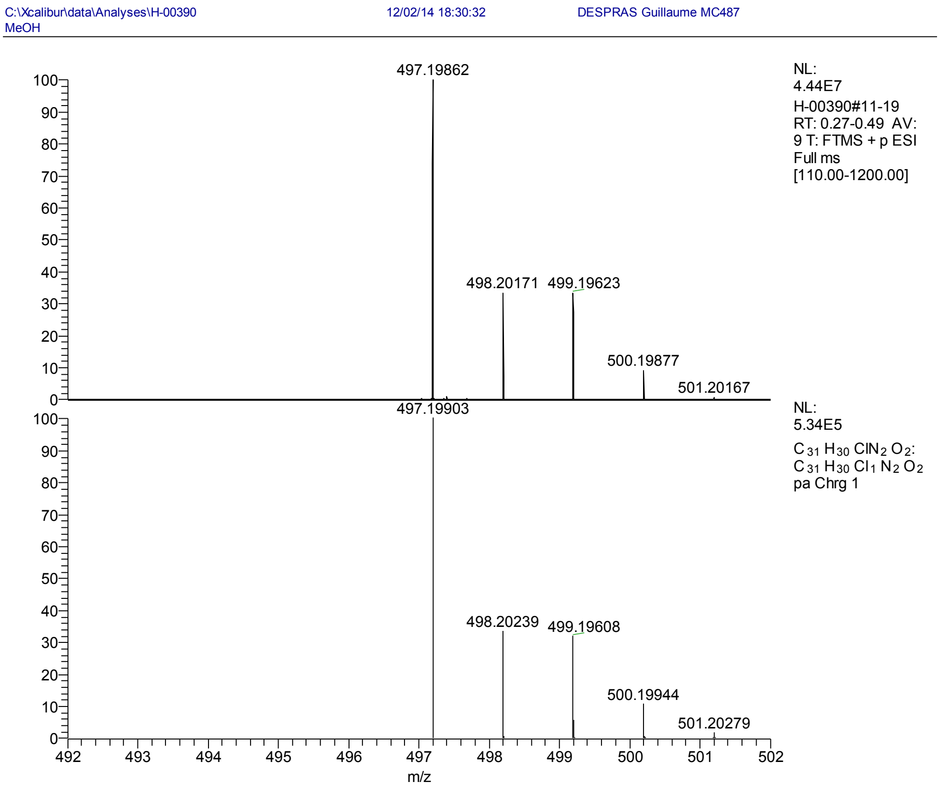

HRMS spectrum of **HR-Cl**

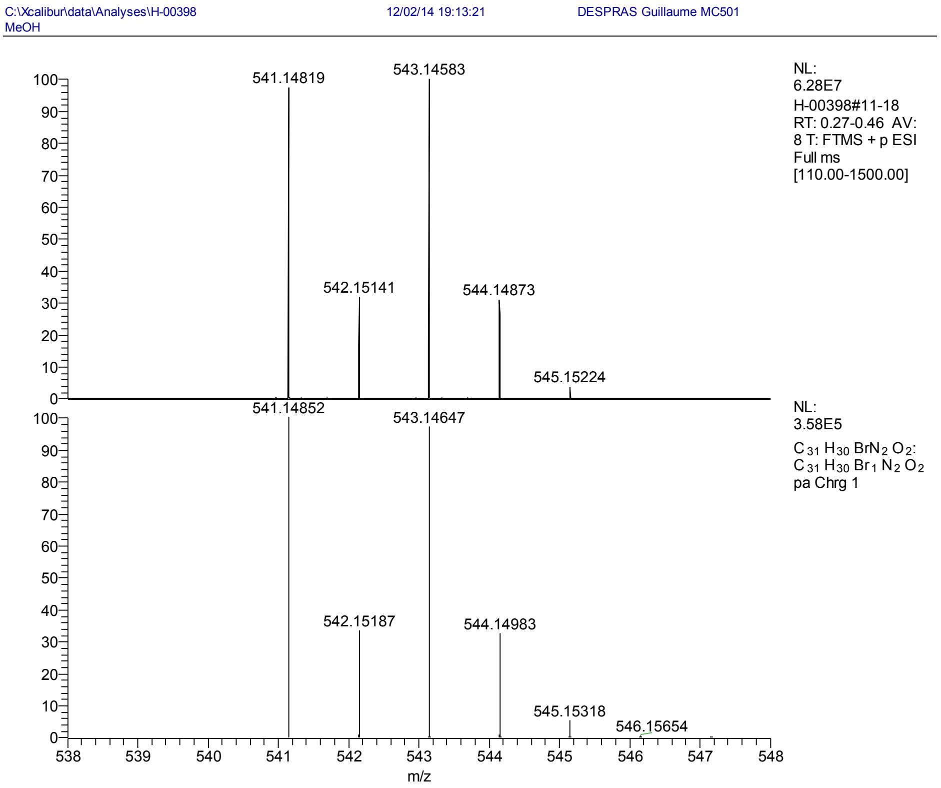

HRMS spectrum of **HR-Br**

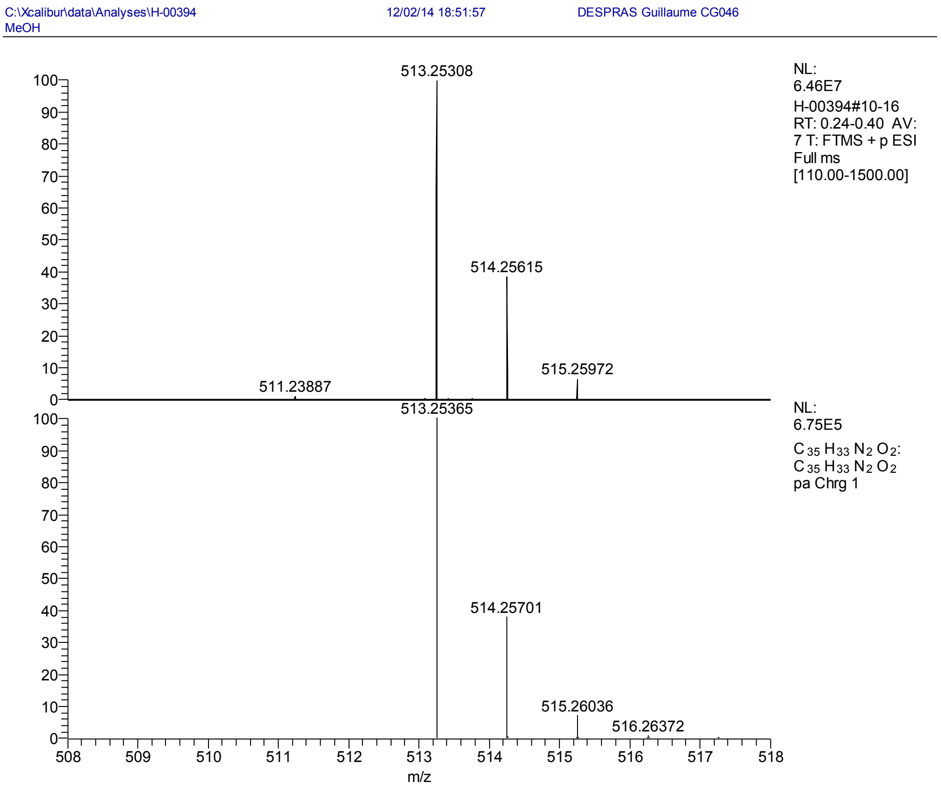

HRMS spectrum of ***p*-Nph**

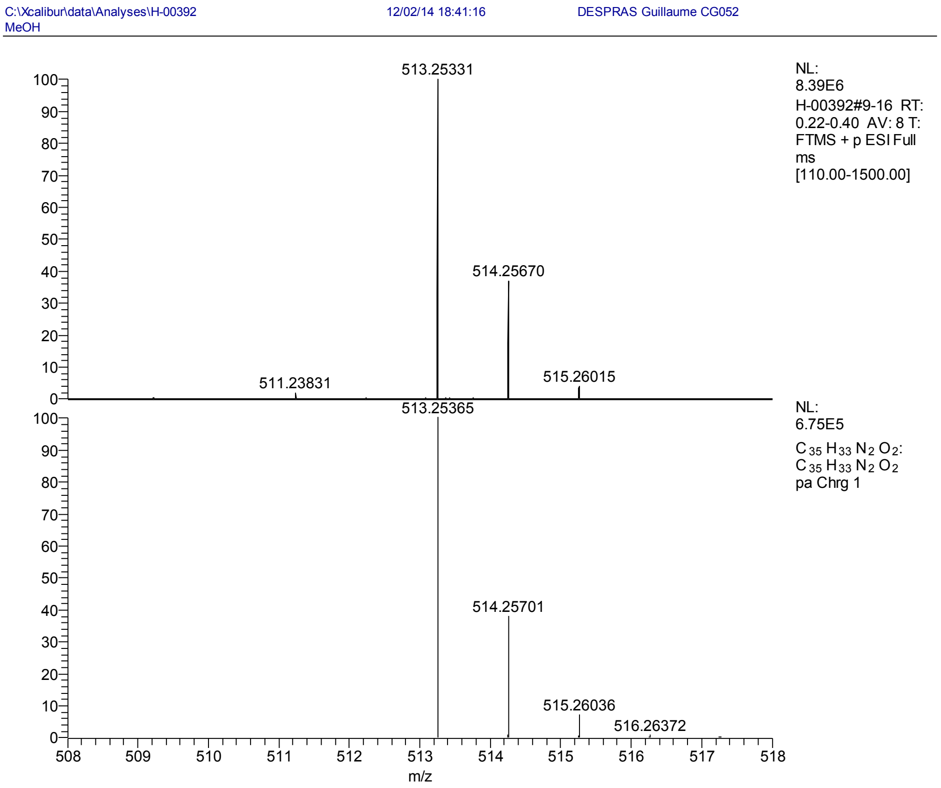

HRMS spectrum of ***o*-Nph**

NMR spectra of functionalisable H-Rubies and their intermediates

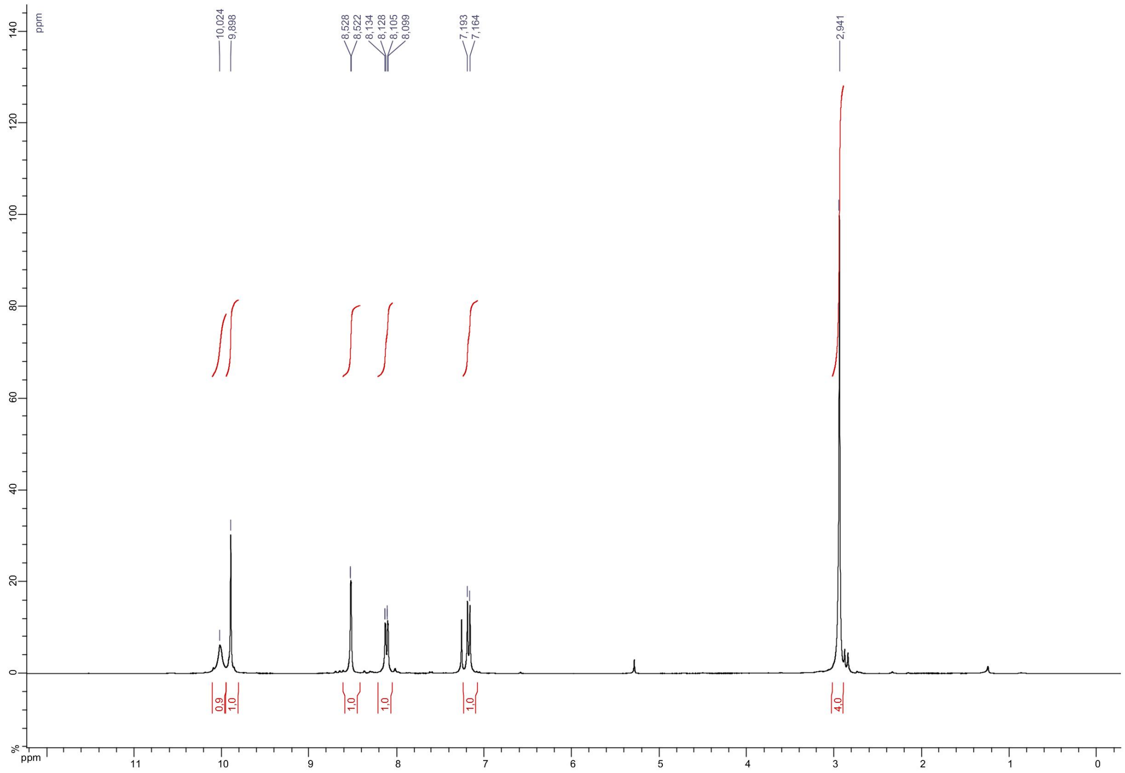

^{1}H NMR spectrum of **1** ($CDCl_3$, 300 MHz)

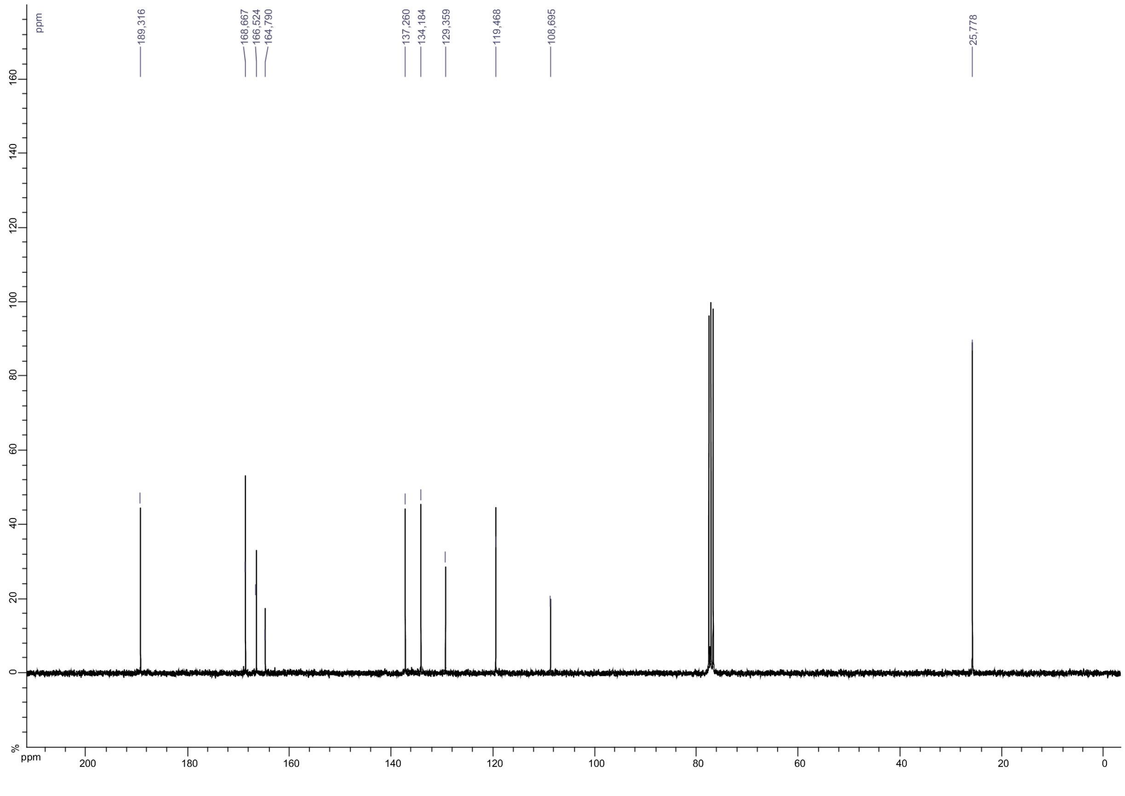

^{13}C NMR spectrum of **1** ($CDCl_3$, 75 MHz)

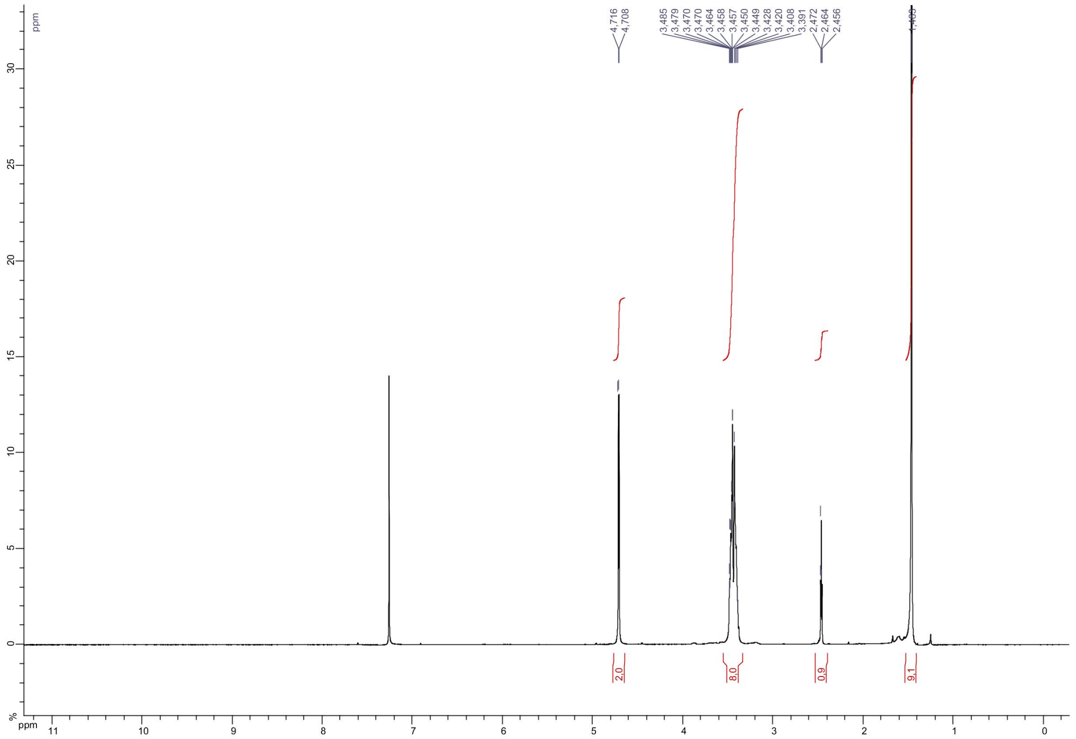

^{1}H NMR spectrum of **1-tert-butyl 4-prop-2-yn-1-yl piperazine-1,4-dicarboxylate** ($CDCl_3$, 300 MHz)

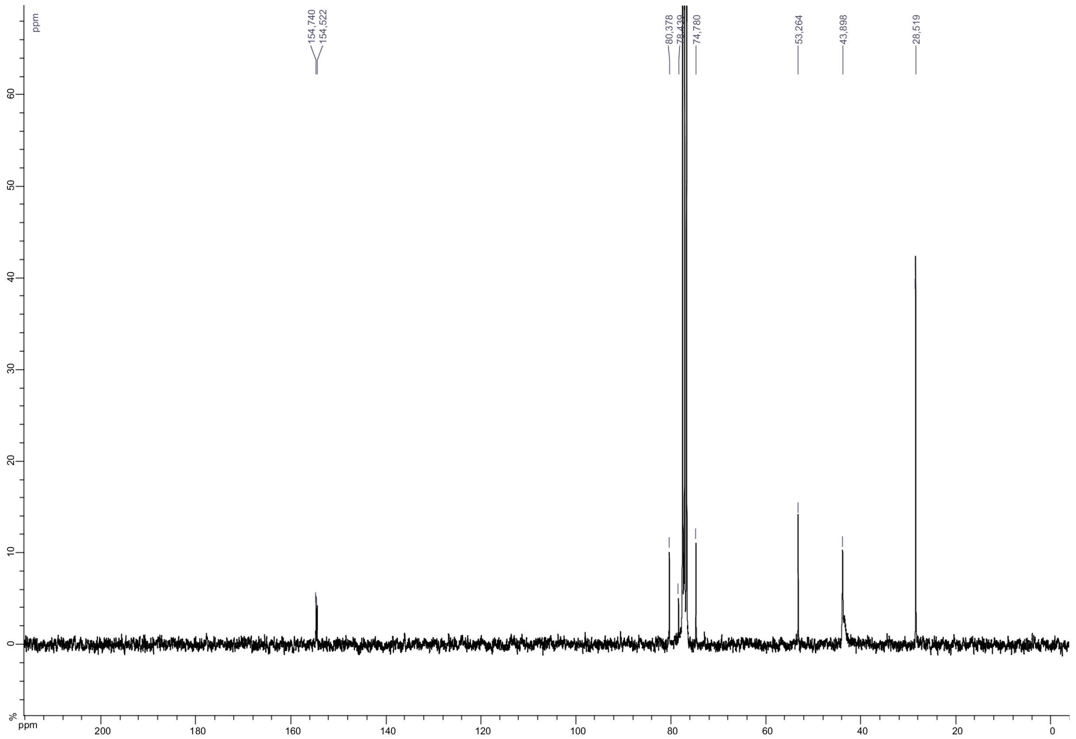

^{13}C NMR spectrum of **1-tert-butyl 4-prop-2-yn-1-yl piperazine-1,4-dicarboxylate** ($CDCl_3$, 75 MHz)

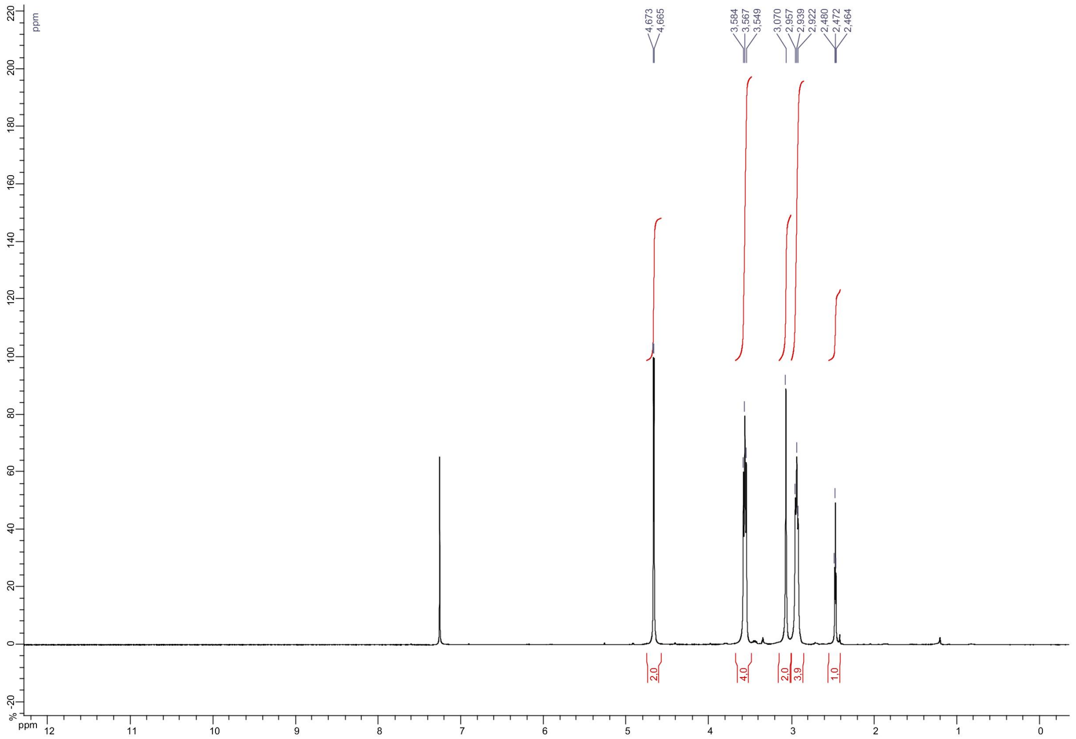

^{1}H NMR spectrum of **4-((prop-2-yn-1-yloxy)carbonyl)piperazin-1-ium 2,2,2-trifluoroacetate** ($CDCl_3$, 300 MHz)

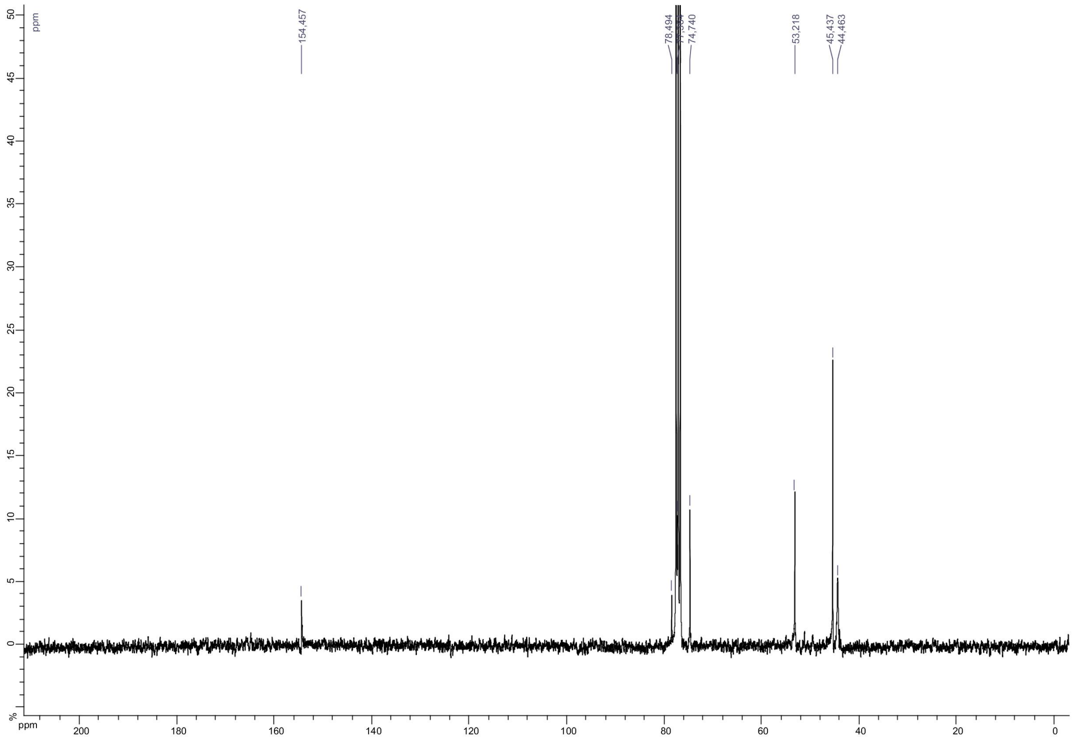

^{13}C NMR spectrum of **4-((prop-2-yn-1-yloxy)carbonyl)piperazin-1-ium 2,2,2-trifluoroacetate** ($CDCl_3$, 75 MHz)

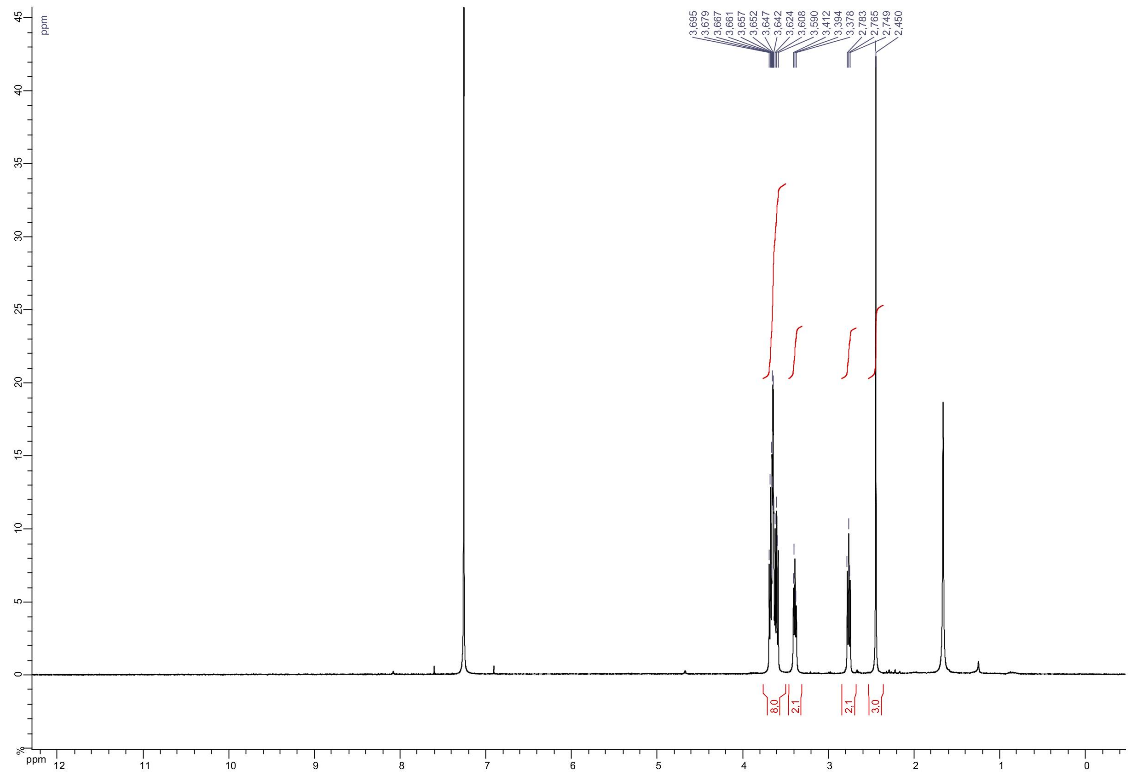

^{1}H NMR spectrum of **2-(2-(2-azidoethoxy)ethoxy)-N-methylethanamine** ($CDCl_3$, 300 MHz)

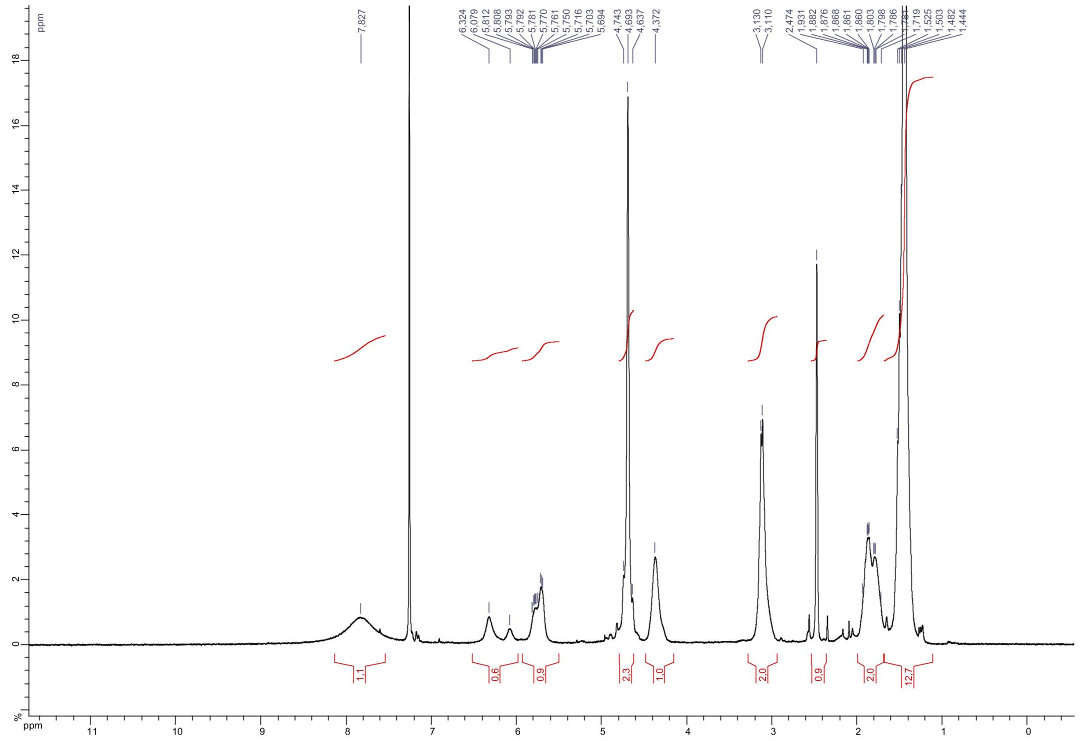

^{1}H NMR spectrum of **6-((tert-butoxycarbonyl)amino)-2-(((prop-2-yn-1-yloxy)carbonyl)amino)hexanoic acid** ($CDCl_3$, 300 MHz)

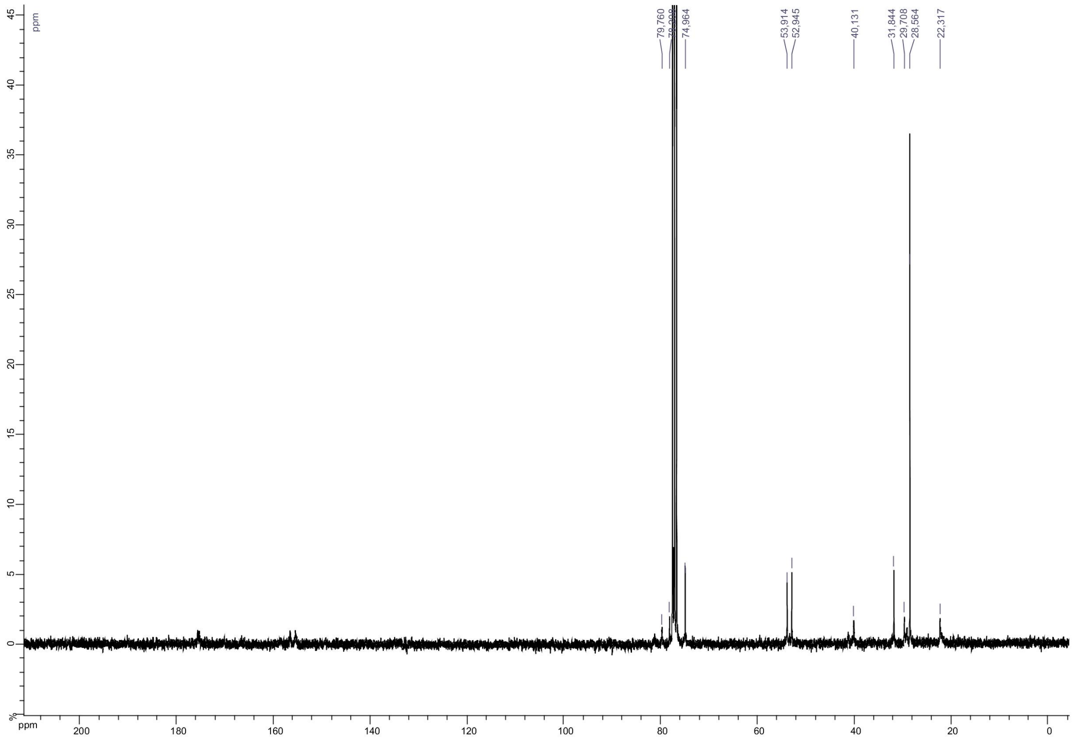

^{13}C NMR spectrum of **6-((tert-butoxycarbonyl)amino)-2-(((prop-2-yn-1-yloxy)carbonyl)amino)hexanoic acid** ($CDCl_3$, 75 MHz)

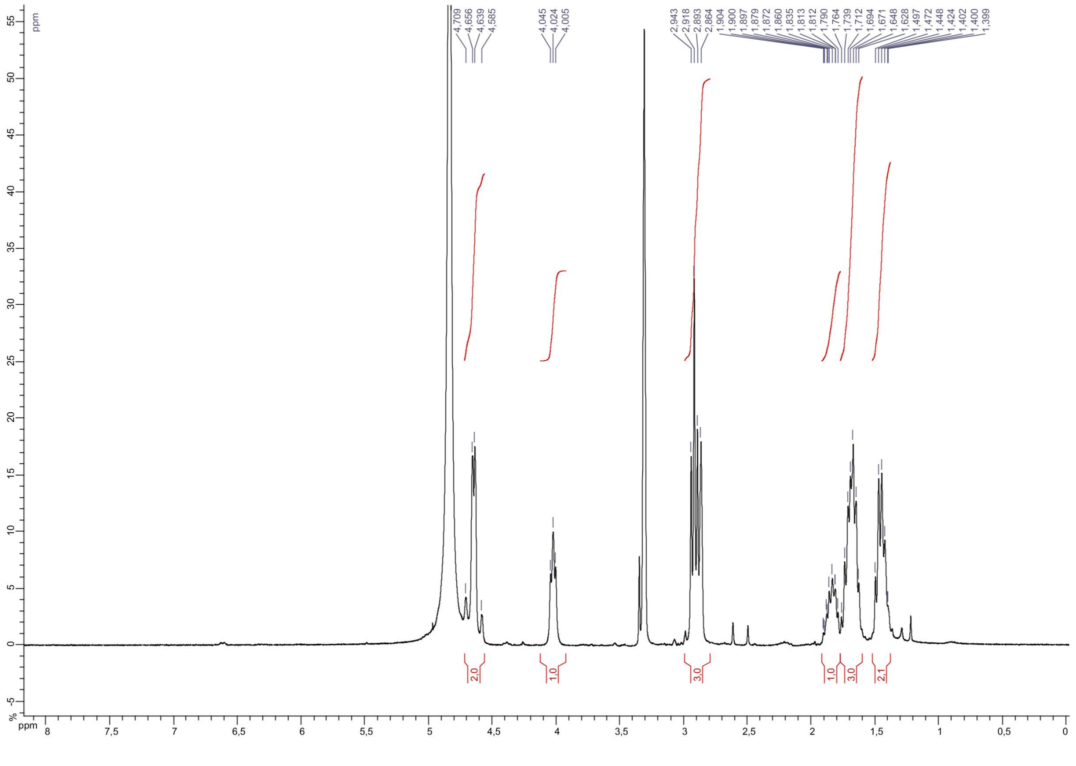

^{1}H NMR spectrum of **5-carboxy-5-(((prop-2-yn-1-yloxy)carbonyl)amino)pentan-1-aminium 2,2,2-trifluoroacetate** ($CDCl_3$, 300 MHz)

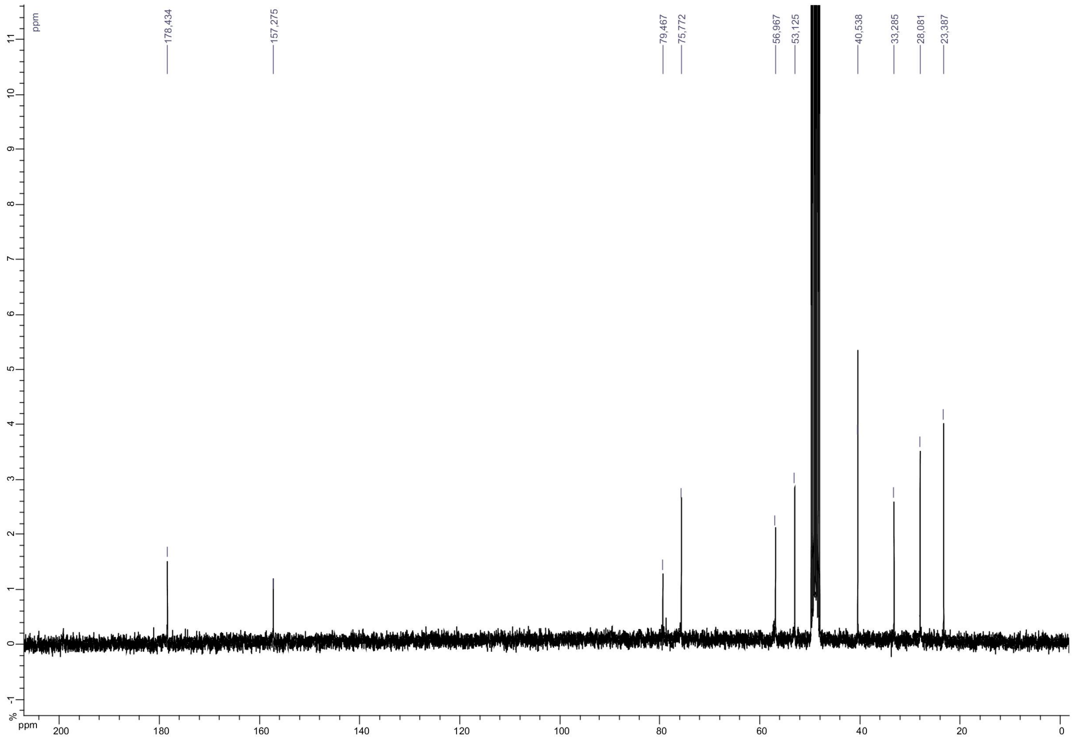

^{13}C NMR spectrum of **5-carboxy-5-(((prop-2-yn-1-yloxy)carbonyl)amino)pentan-1-aminium 2,2,2-trifluoroacetate** ($CDCl_3$, 75 MHz)

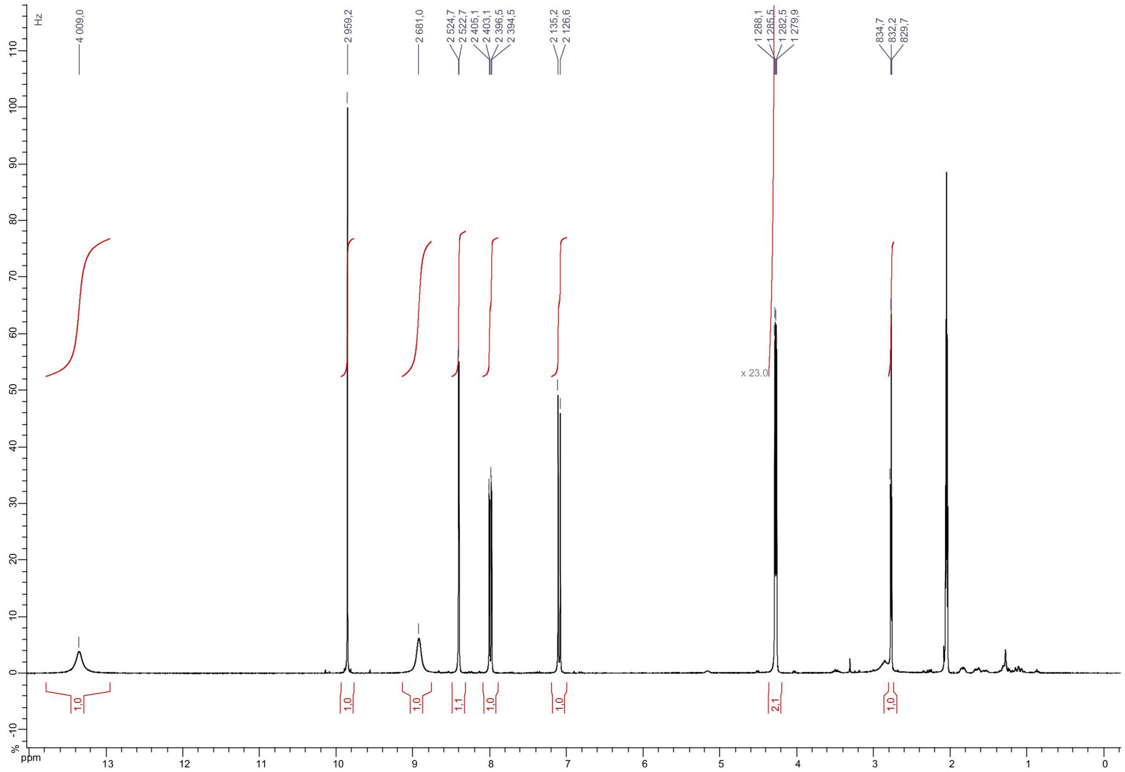

^{1}H NMR spectrum of **2a** ($(CD_3)_3CO$, 300 MHz)

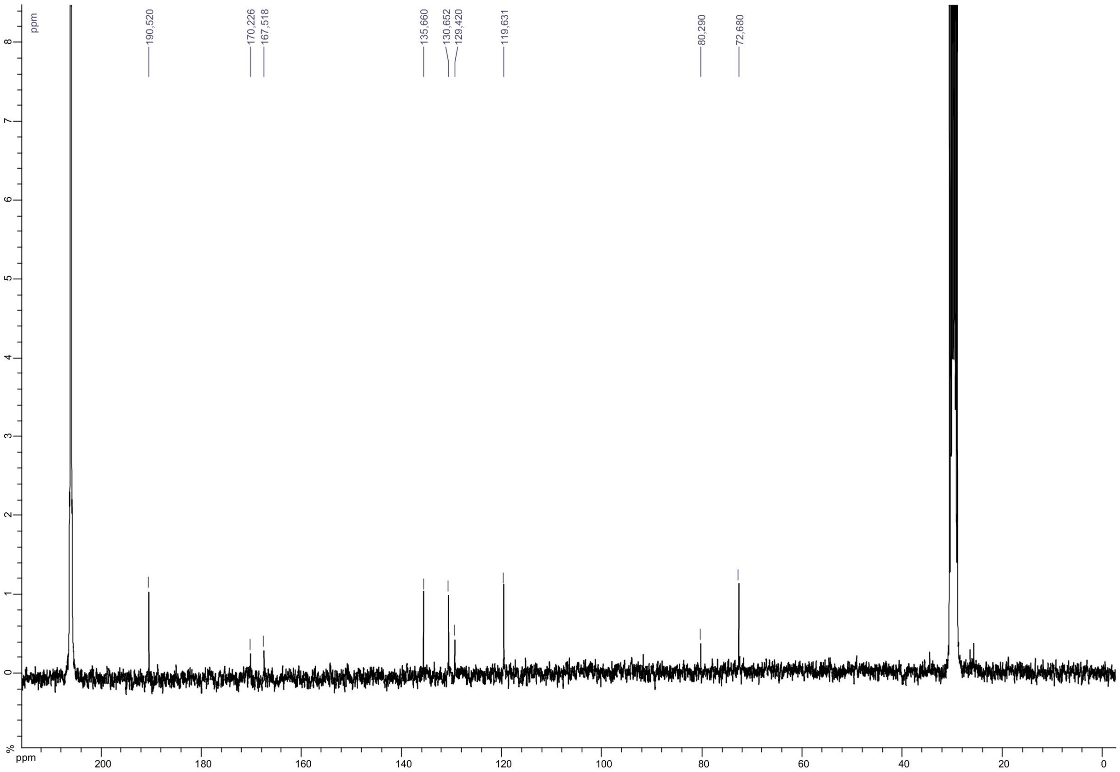

^{13}C NMR spectrum of **2a** ($(CD_3)_3CO$, 75 MHz)

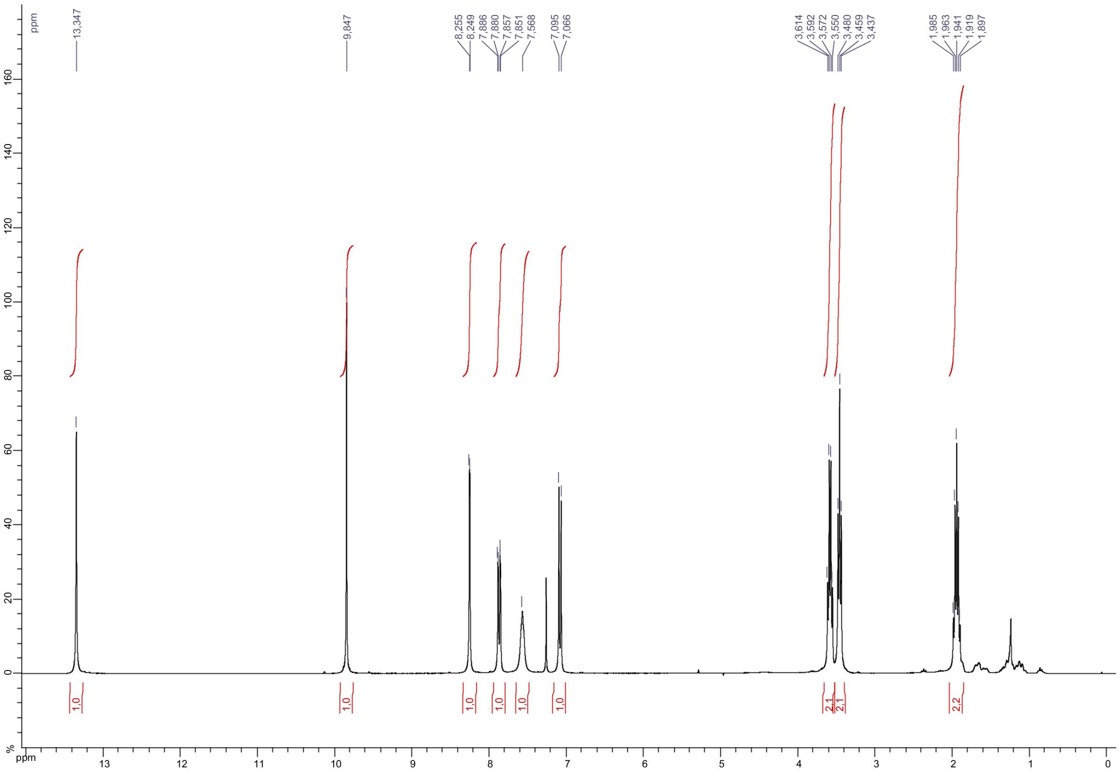

^{1}H NMR spectrum of **2b** ($CDCl_3$, 300 MHz)

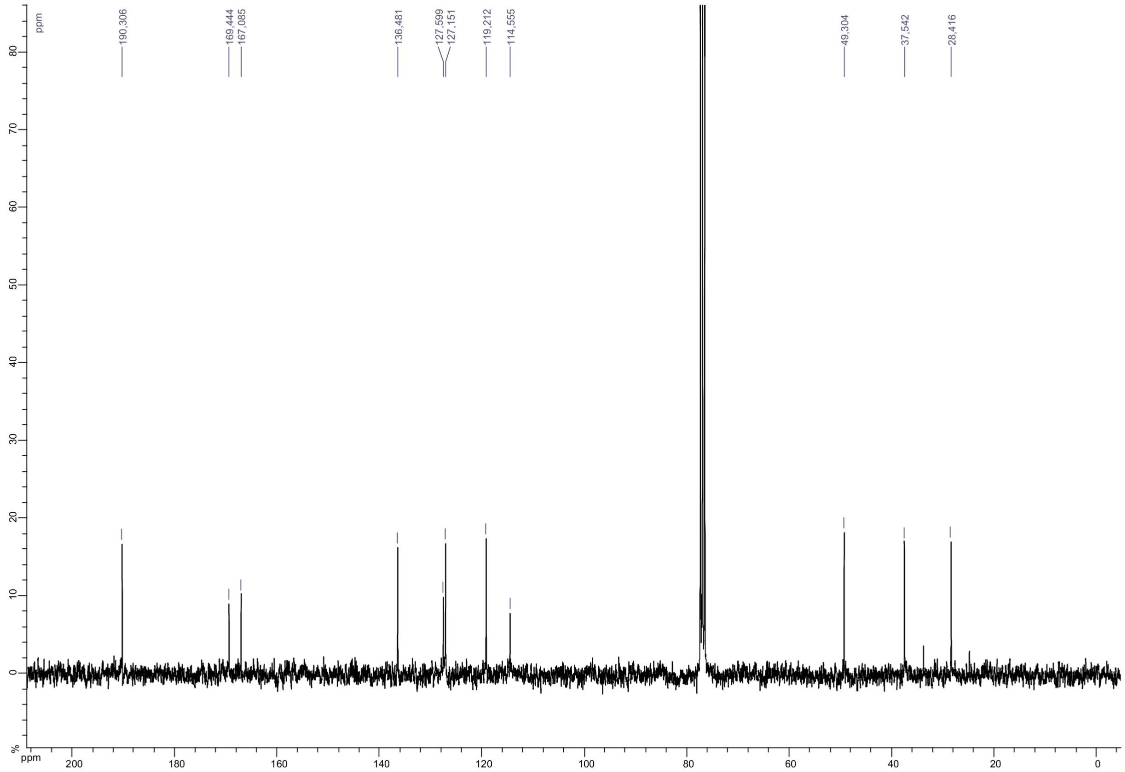

^{13}C NMR spectrum of **2b** ($CDCl_3$, 75 MHz)

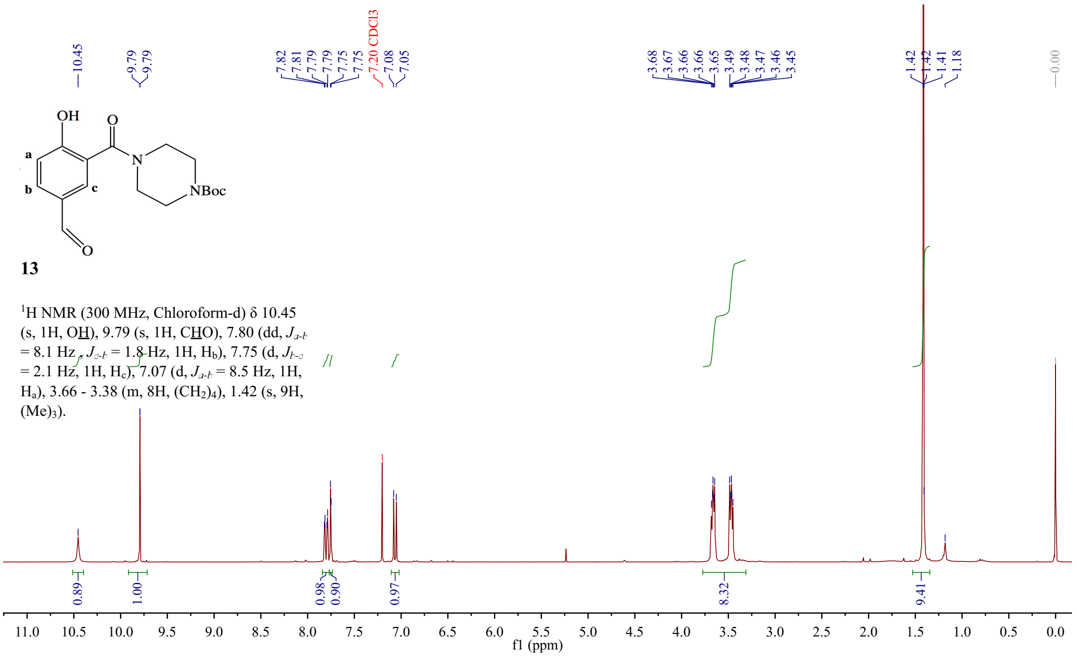

^{1}H NMR spectrum of **2c’** ($CDCl_3$, 300 MHz)

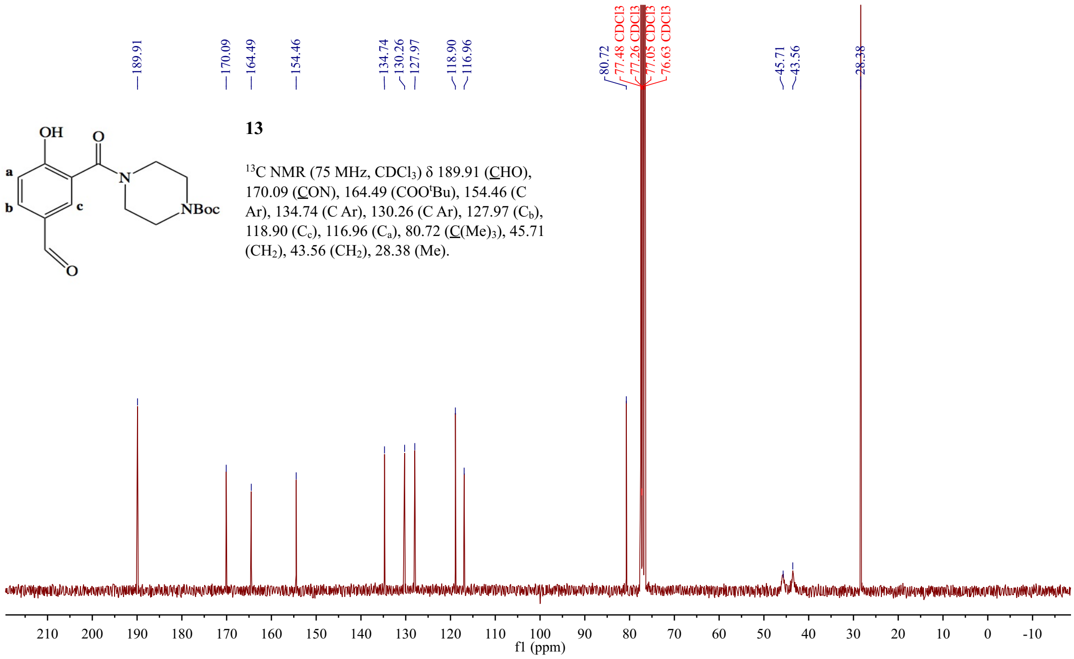

^{13}C NMR spectrum of **2c’** ($CDCl_3$, 75 MHz)

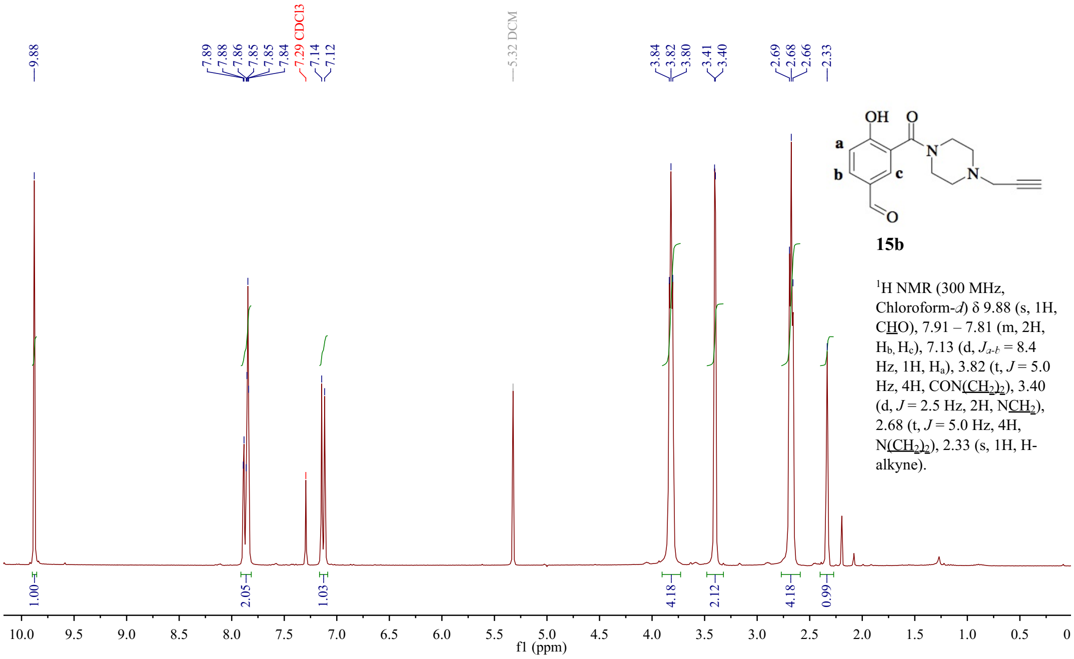

^{1}H NMR spectrum of **2c** ($CDCl_3$, 300 MHz)

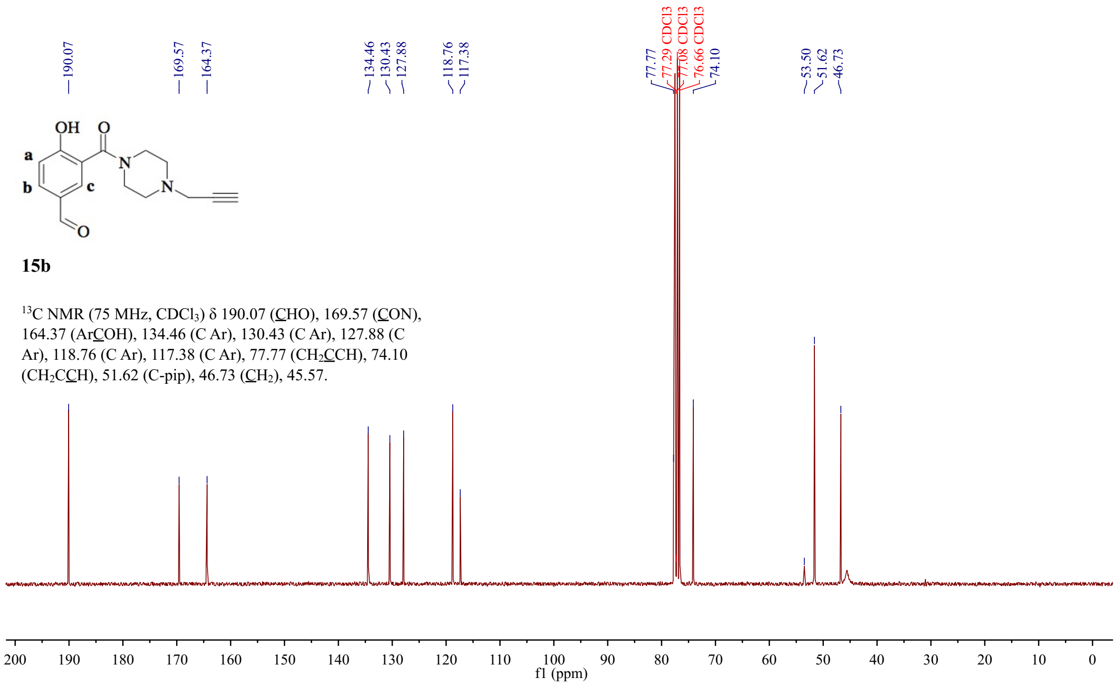

^{13}C NMR spectrum of **2c** ($CDCl_3$, 75 MHz)

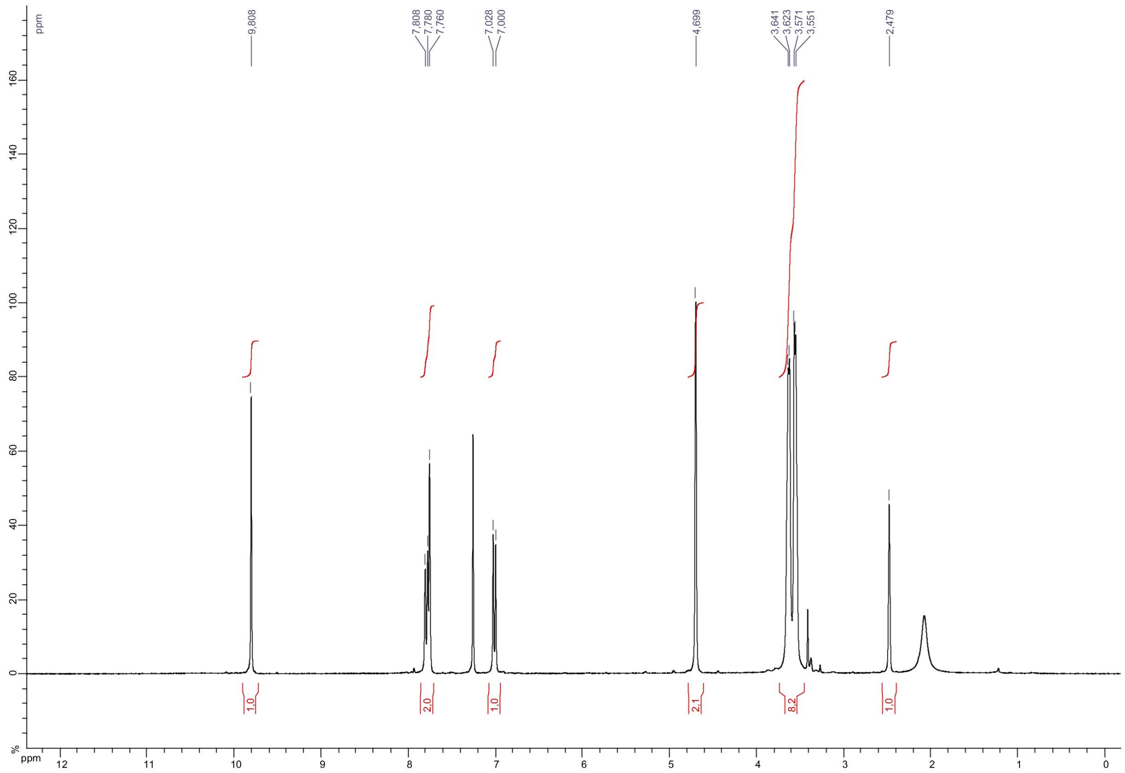

^{1}H NMR spectrum of **2d** ($CDCl_3$, 300 MHz)

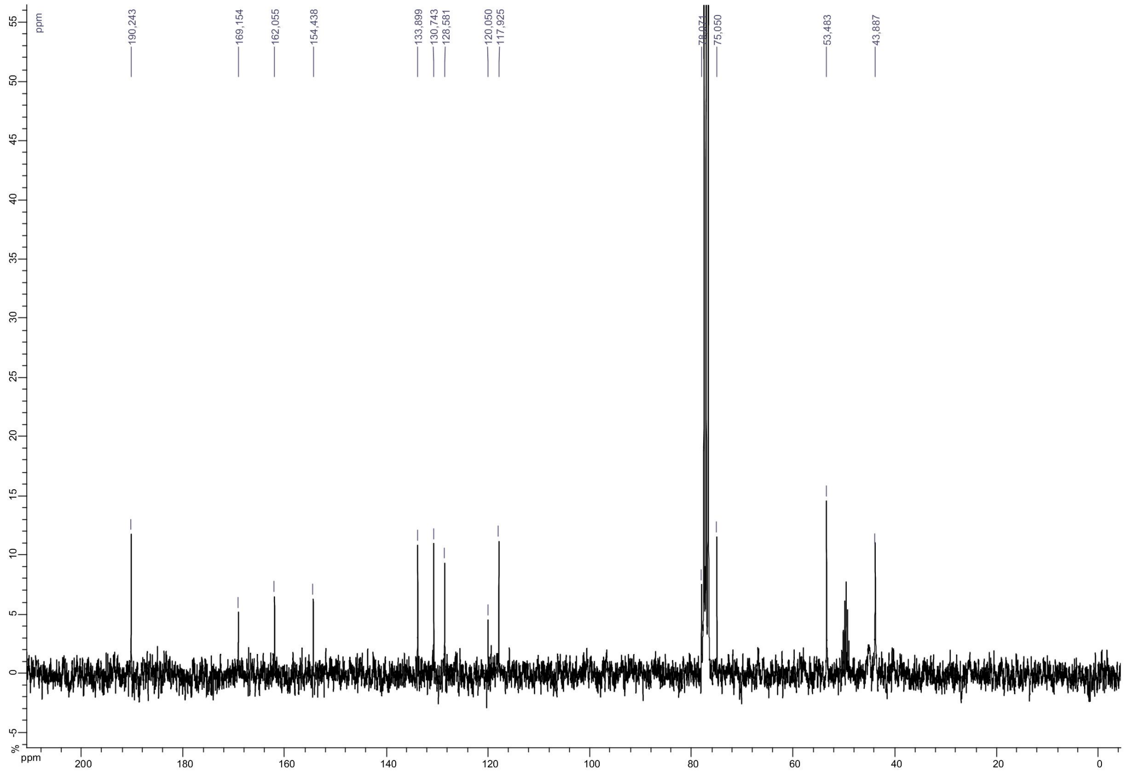

^{13}C NMR spectrum of **2d** ($CDCl_3$, 75 MHz)

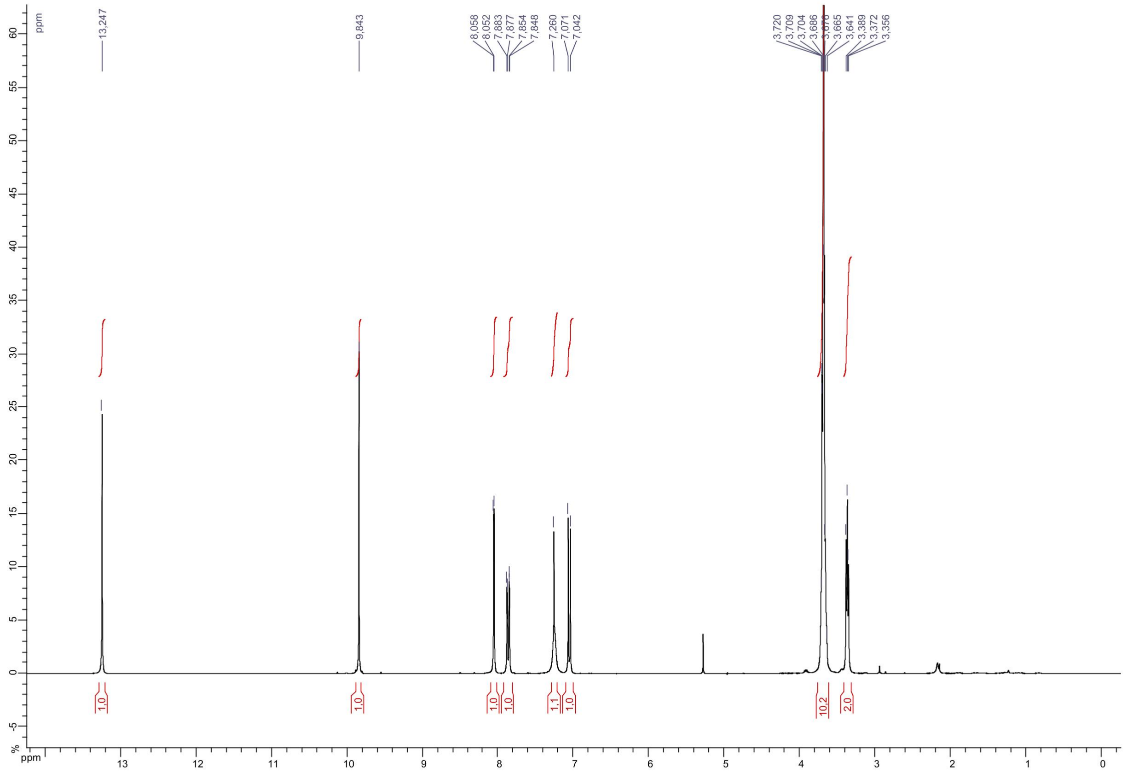

^{1}H NMR spectrum of **2e** ($CDCl_3$, 300 MHz)

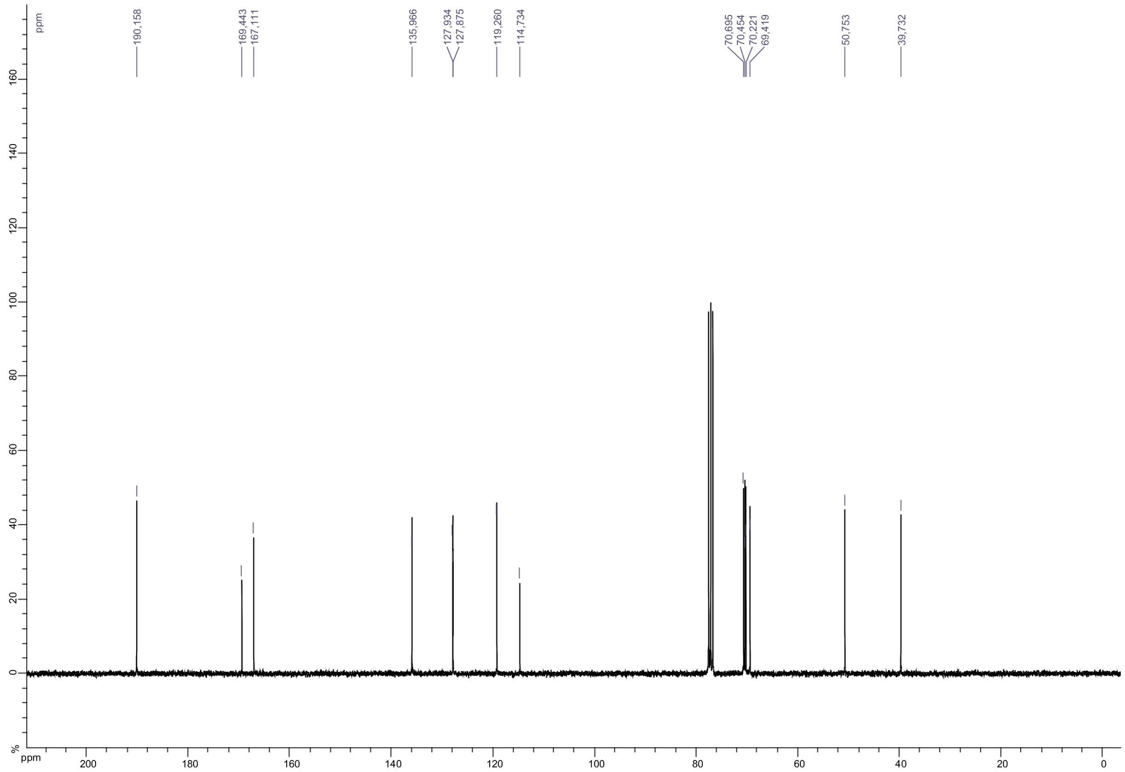

^{13}C NMR spectrum of **2e** ($CDCl_3$, 75 MHz)

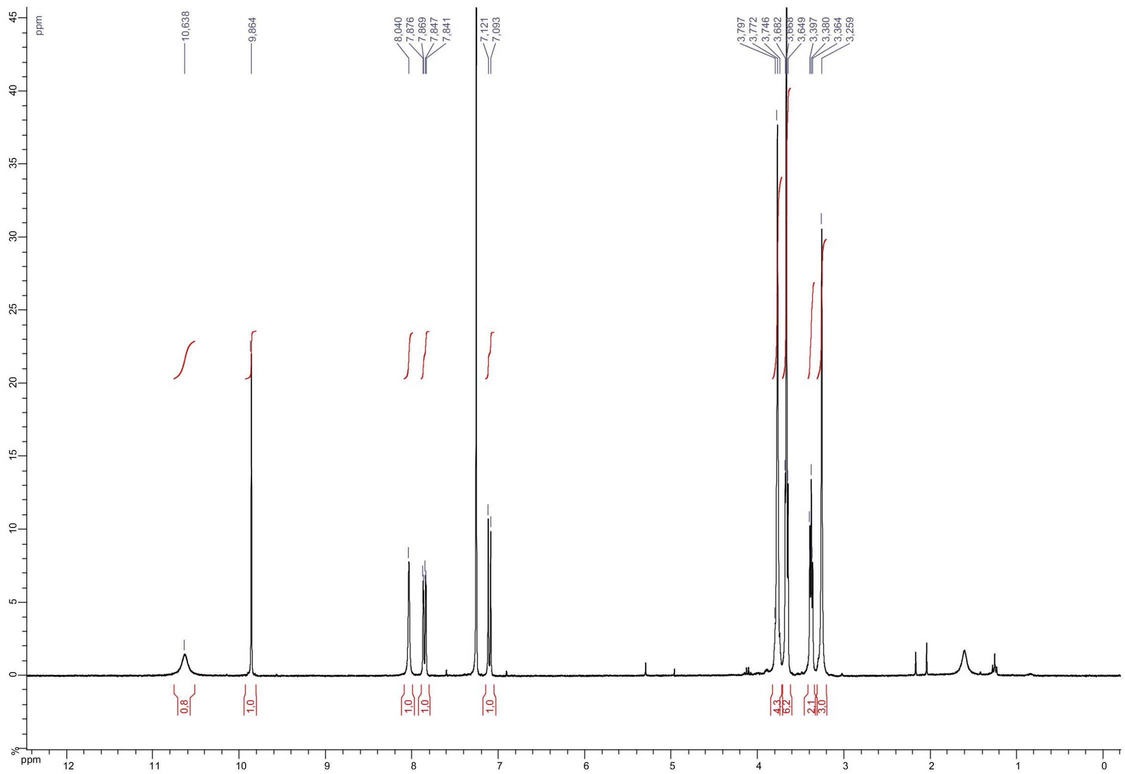

^{1}H NMR spectrum of **2f** ($CDCl_3$, 300 MHz)

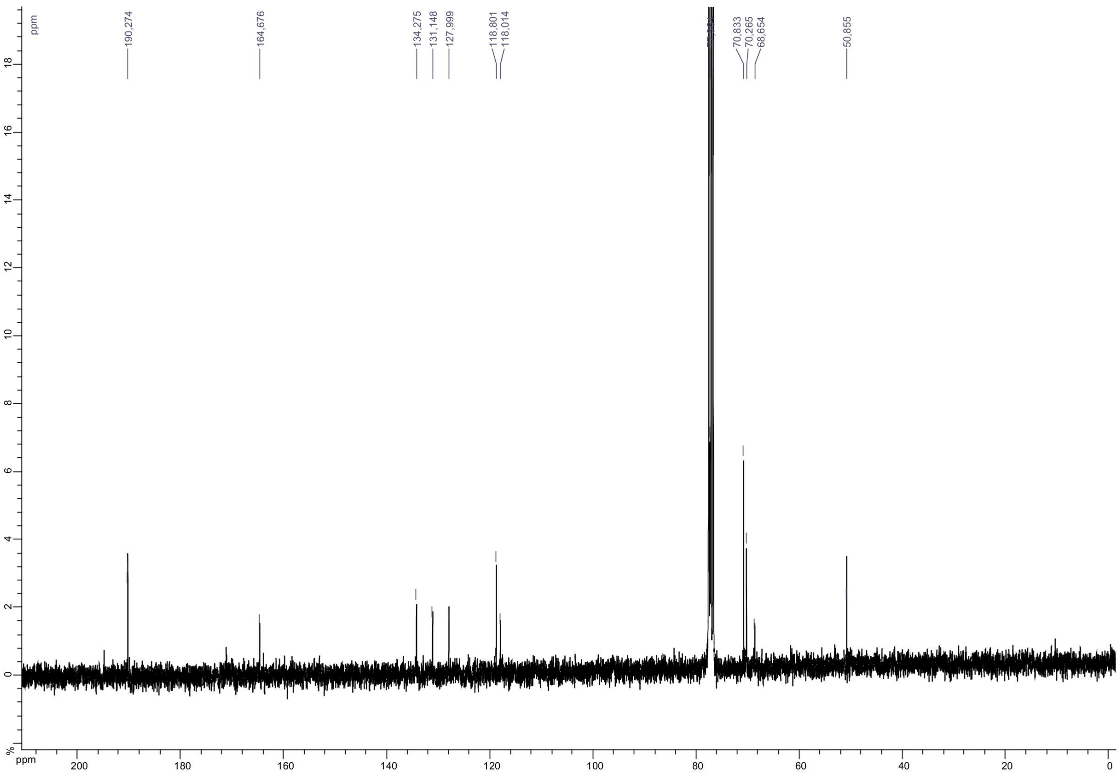

^{13}C NMR spectrum of **2f** ($CDCl_3$, 75 MHz)

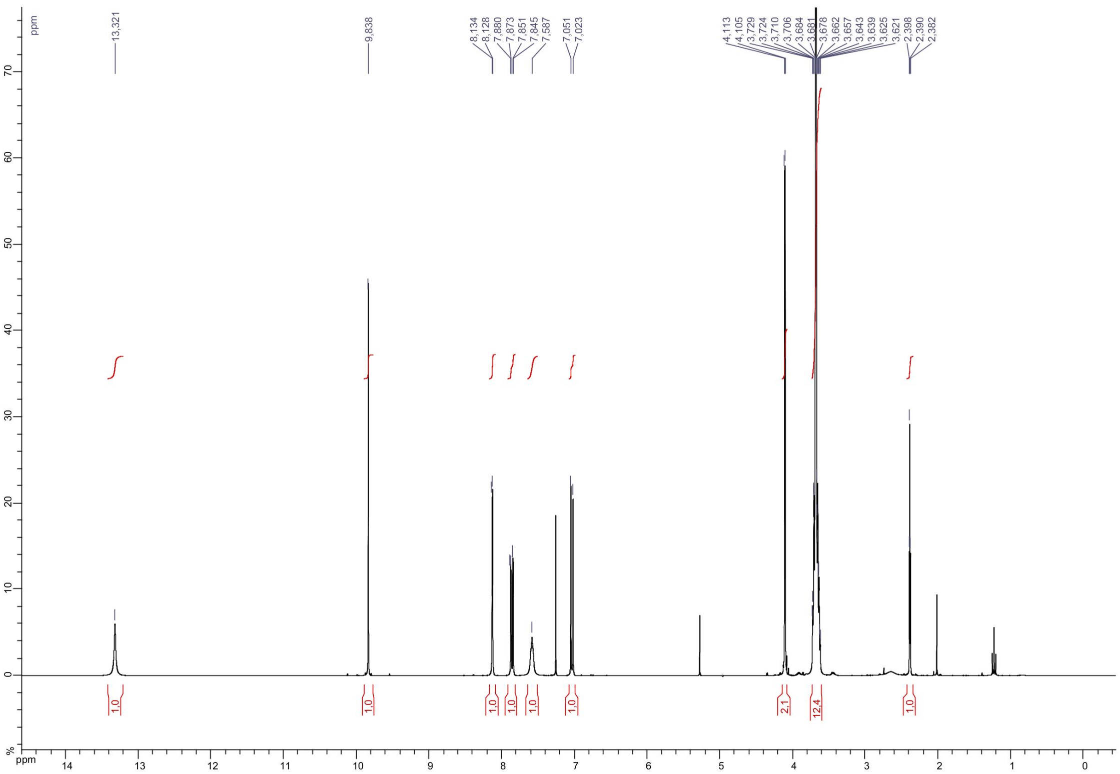

^{1}H NMR spectrum of **2g** ($CDCl_3$, 300 MHz)

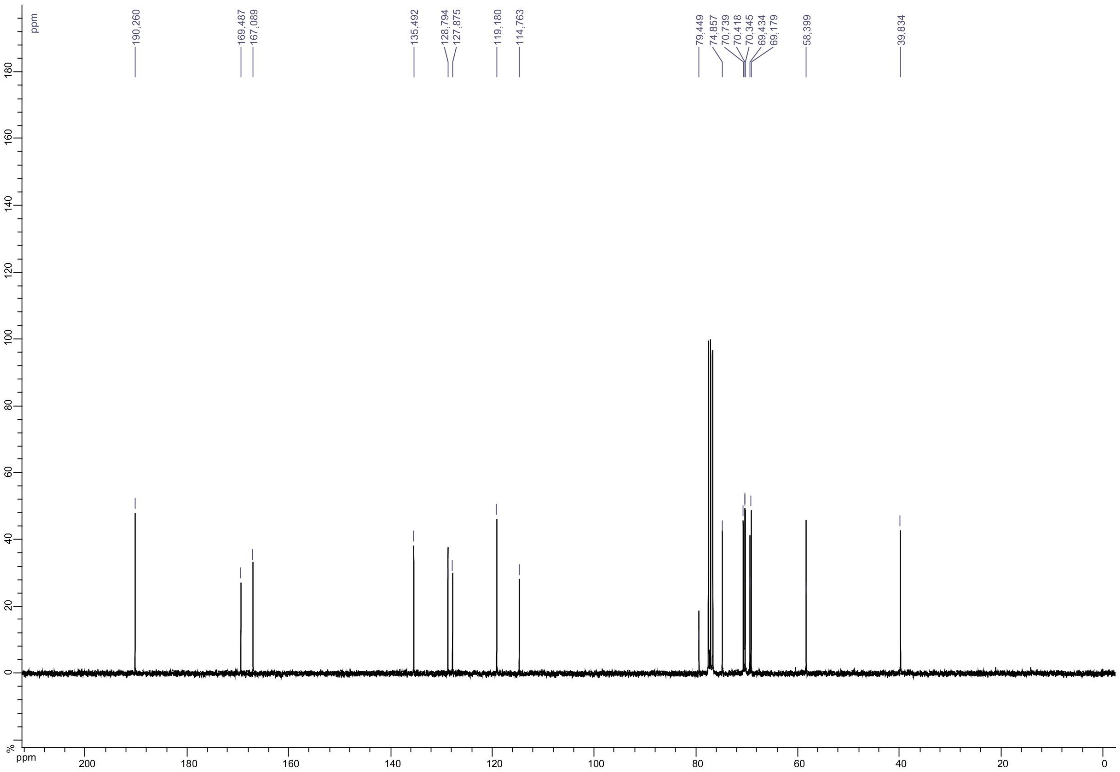

^{13}C NMR spectrum of **2g** ($CDCl_3$, 75 MHz)

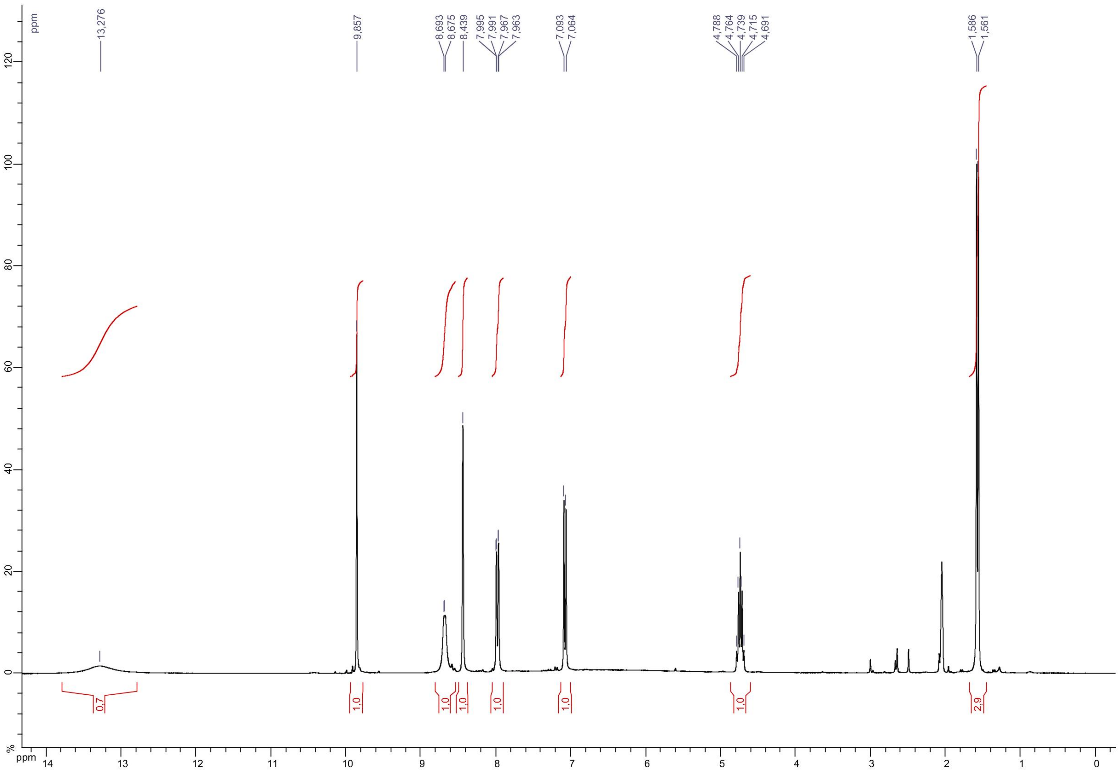

^{1}H NMR spectrum of **2h** ($CDCl_3$, 300 MHz)

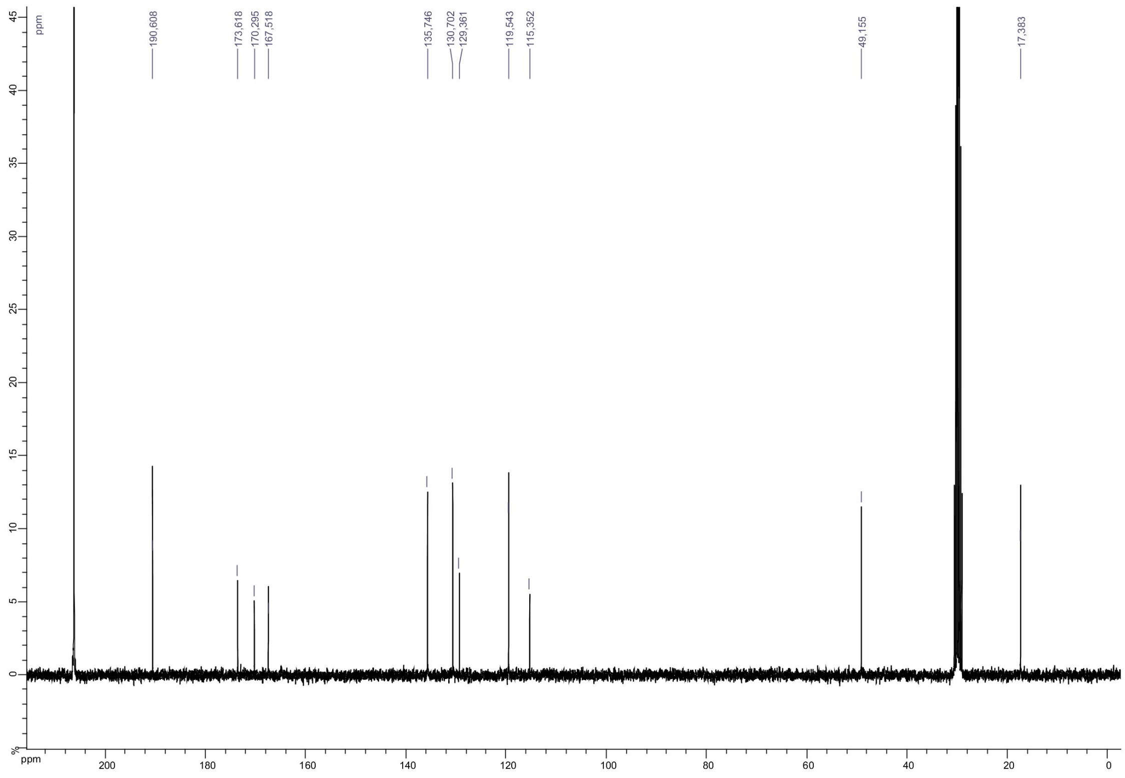

^{13}C NMR spectrum of **2h** ($CDCl_3$, 75 MHz)

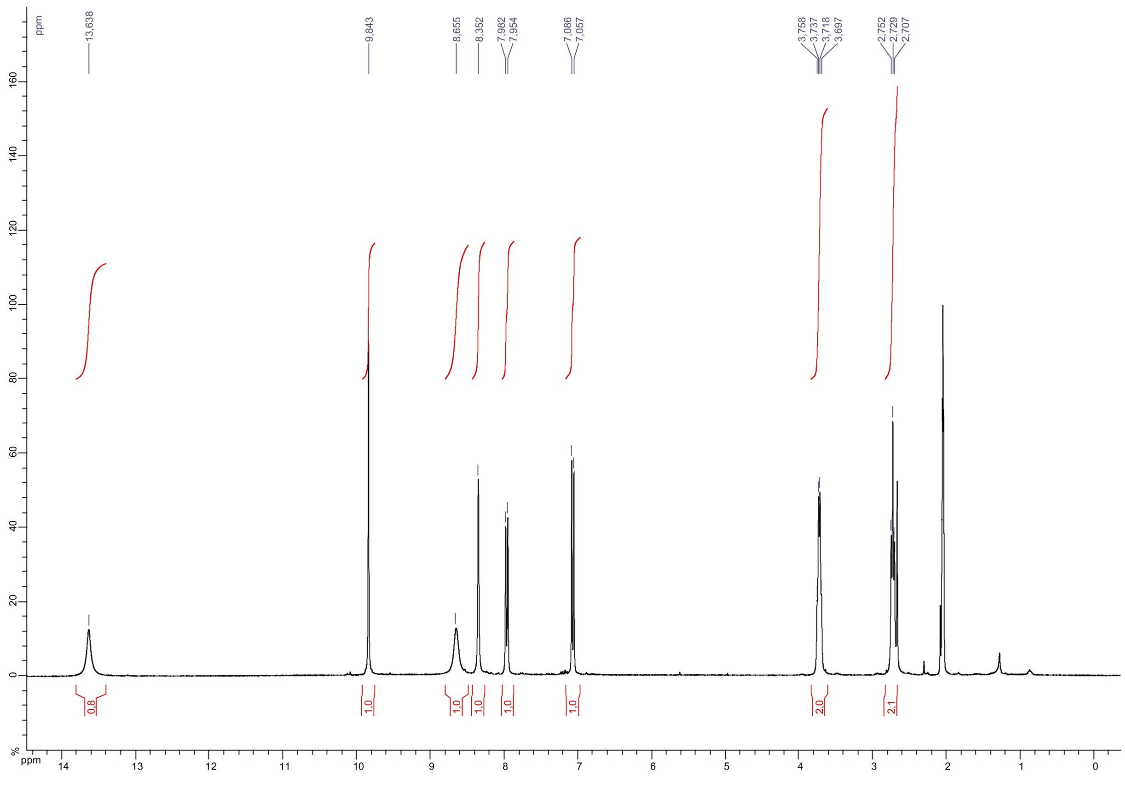

^{1}H NMR spectrum of **2i** ($CDCl_3$, 300 MHz)

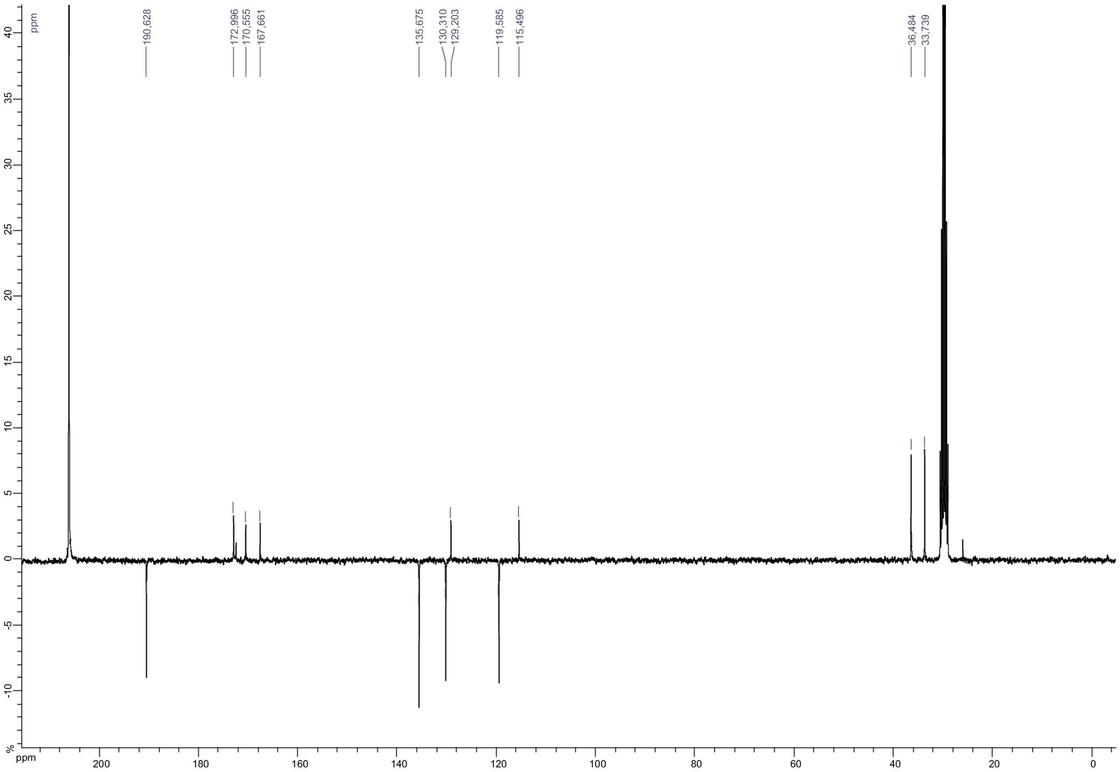

^{13}C NMR (JMOD) spectrum of **2i** ($CDCl_3$, 75 MHz)

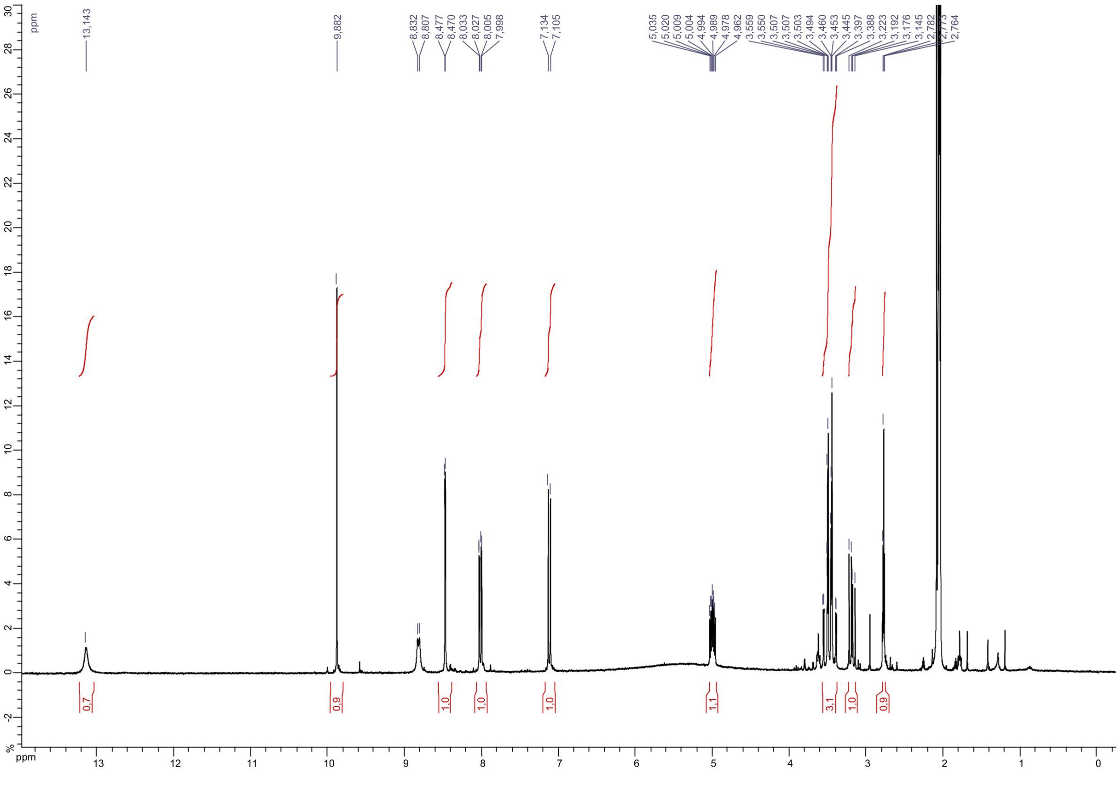

^{1}H NMR spectrum of **2j** ($CDCl_3$, 300 MHz)

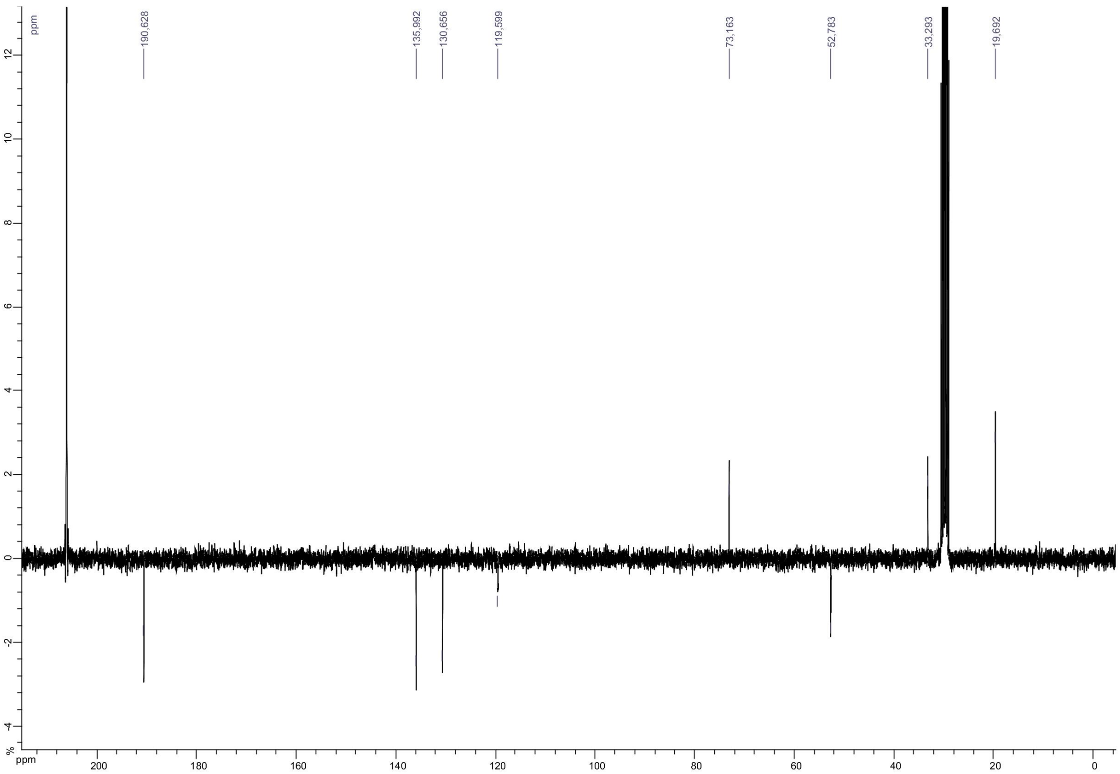

^{13}C NMR (JMOD) spectrum of **2j** ($CDCl_3$, 75 MHz)

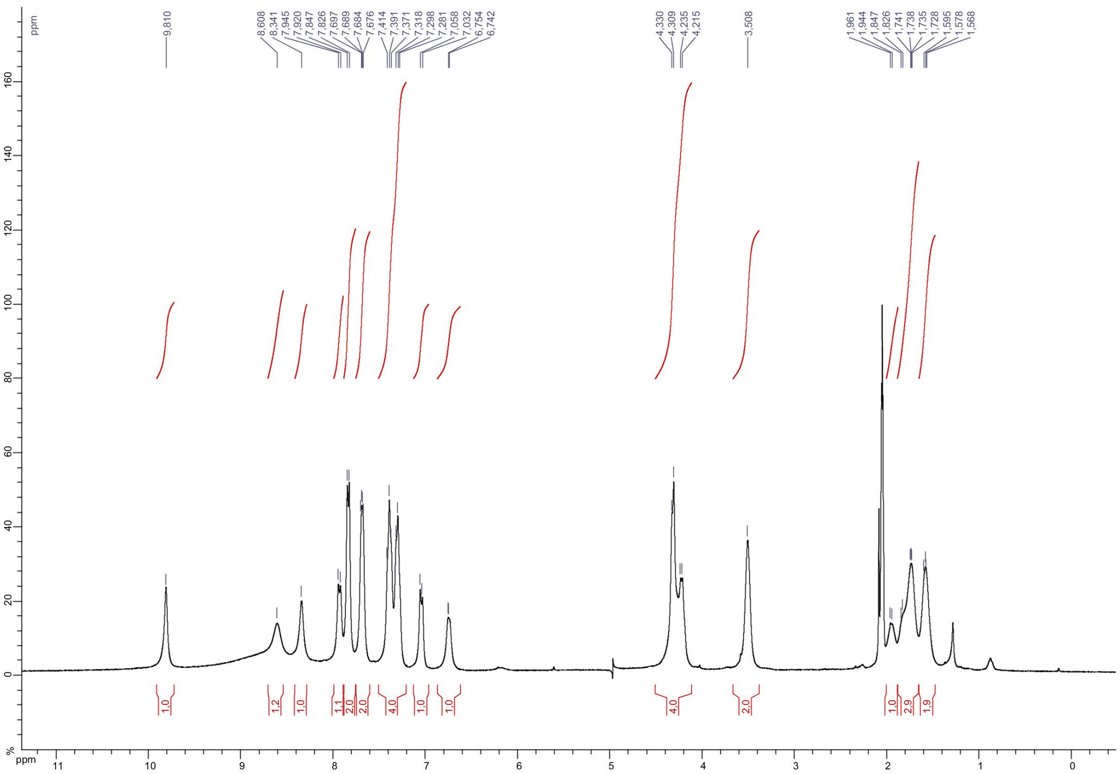

^{1}H NMR spectrum of **2k** ($(CD_3)_3CO$, 300 MHz)

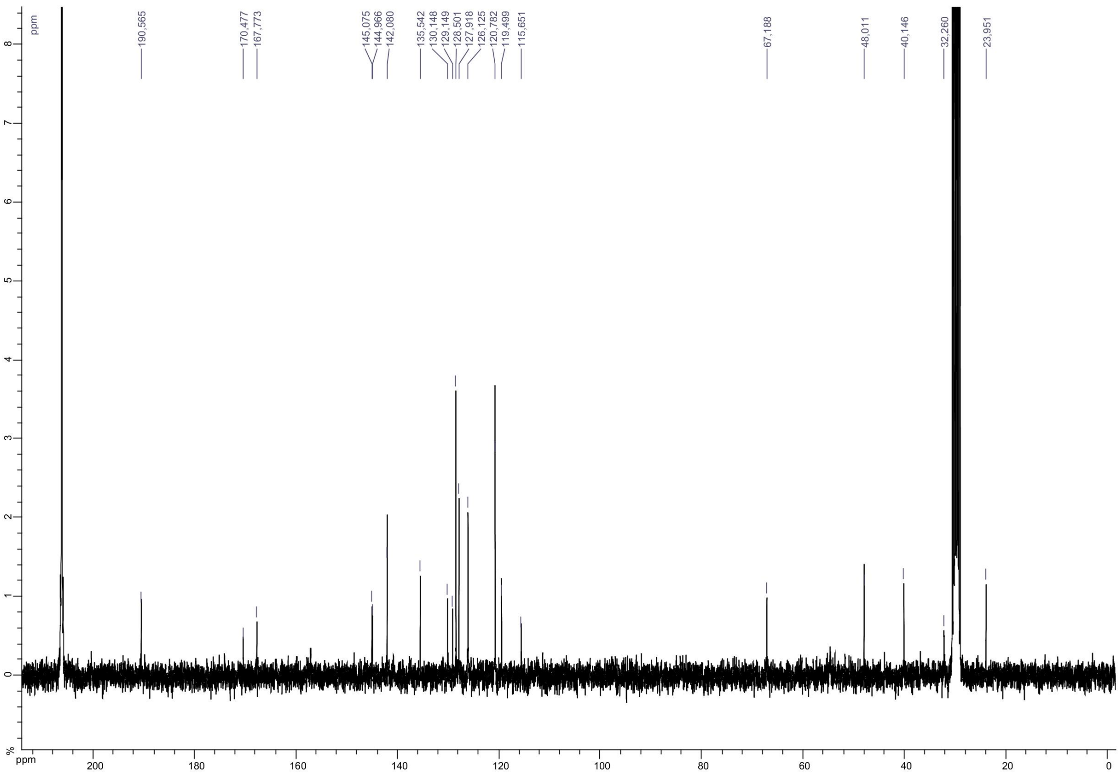

^{13}C NMR spectrum of **2k** ($(CD_3)_3CO$, 75 MHz)

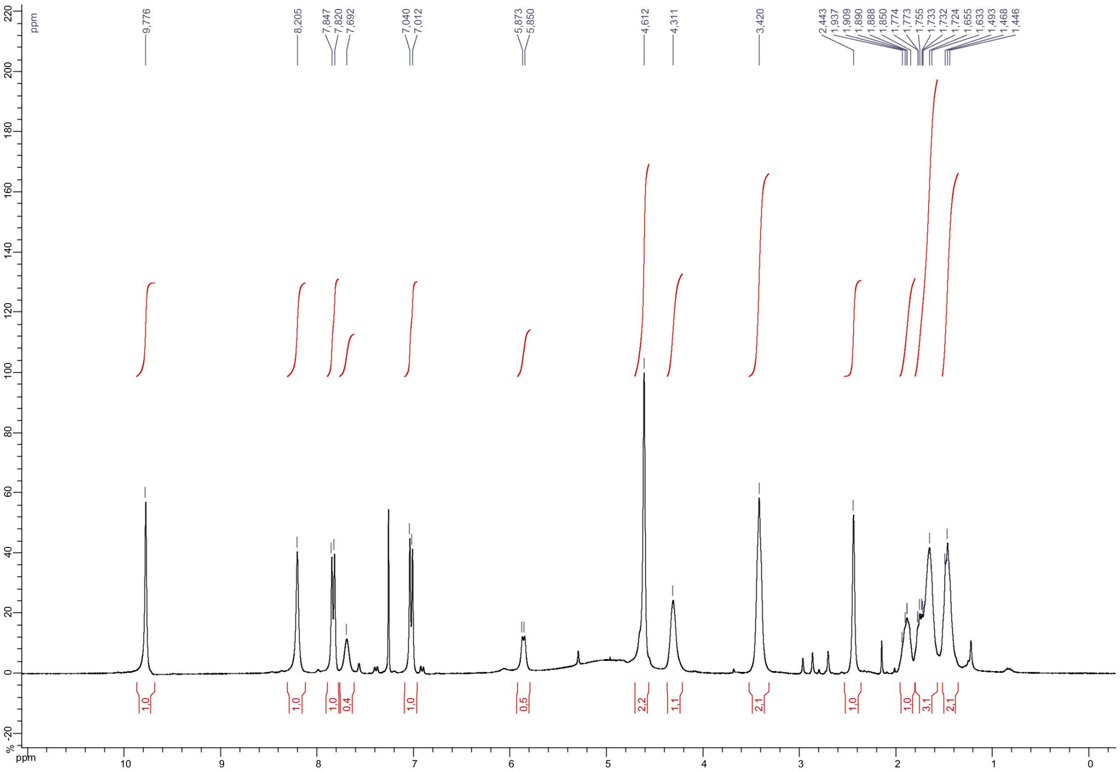

^{1}H NMR spectrum of **2l** ($CDCl_3$, 300 MHz)

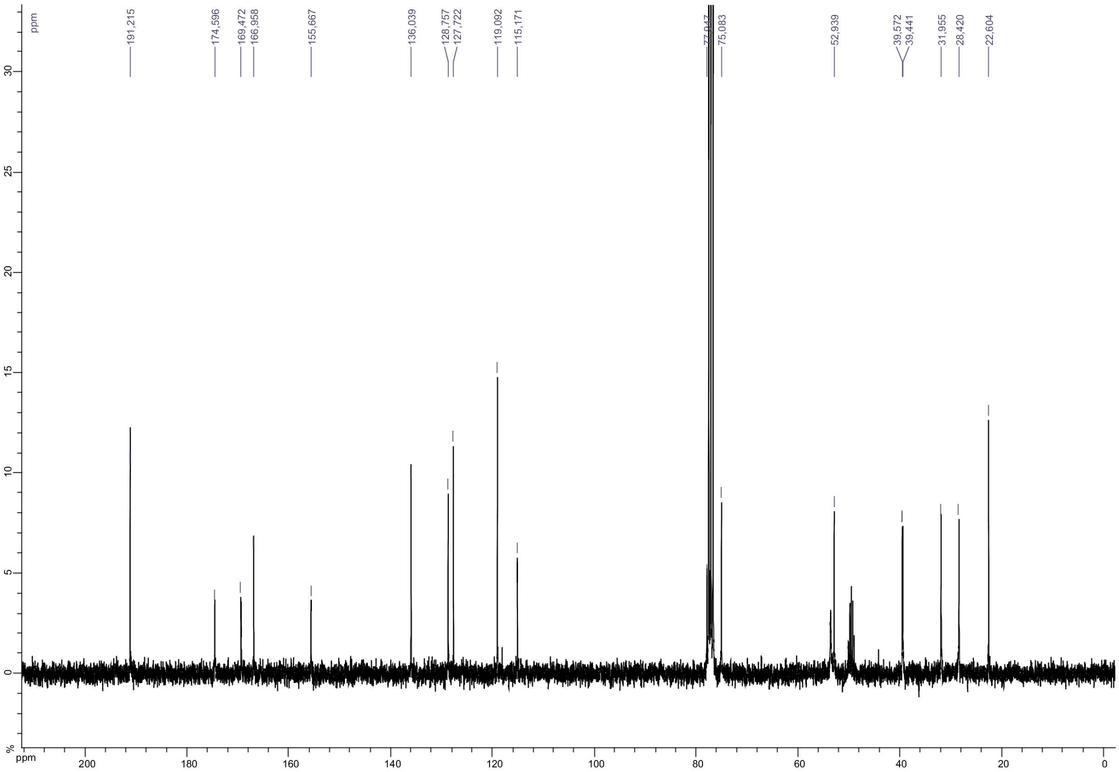

^{13}C NMR spectrum of **2l** ($CDCl_3$ + εCD_3OD, 75 MHz)

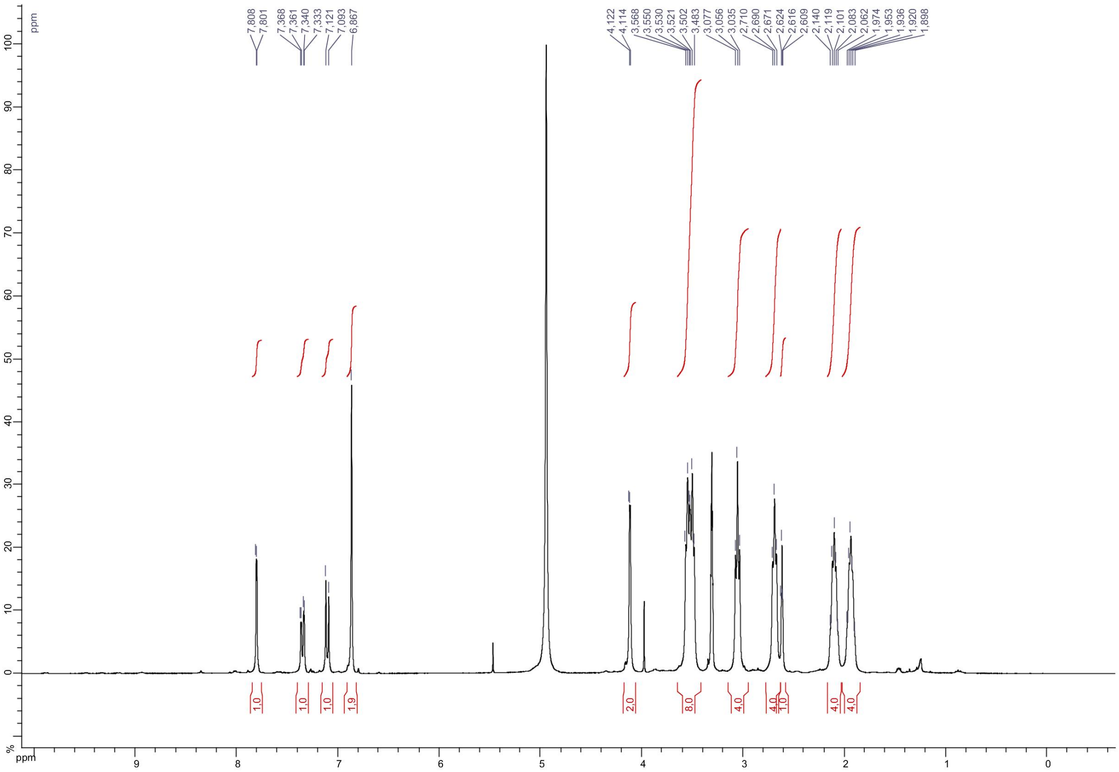

^{1}H NMR spectrum of **2b** (CD_3OD, 300 MHz)

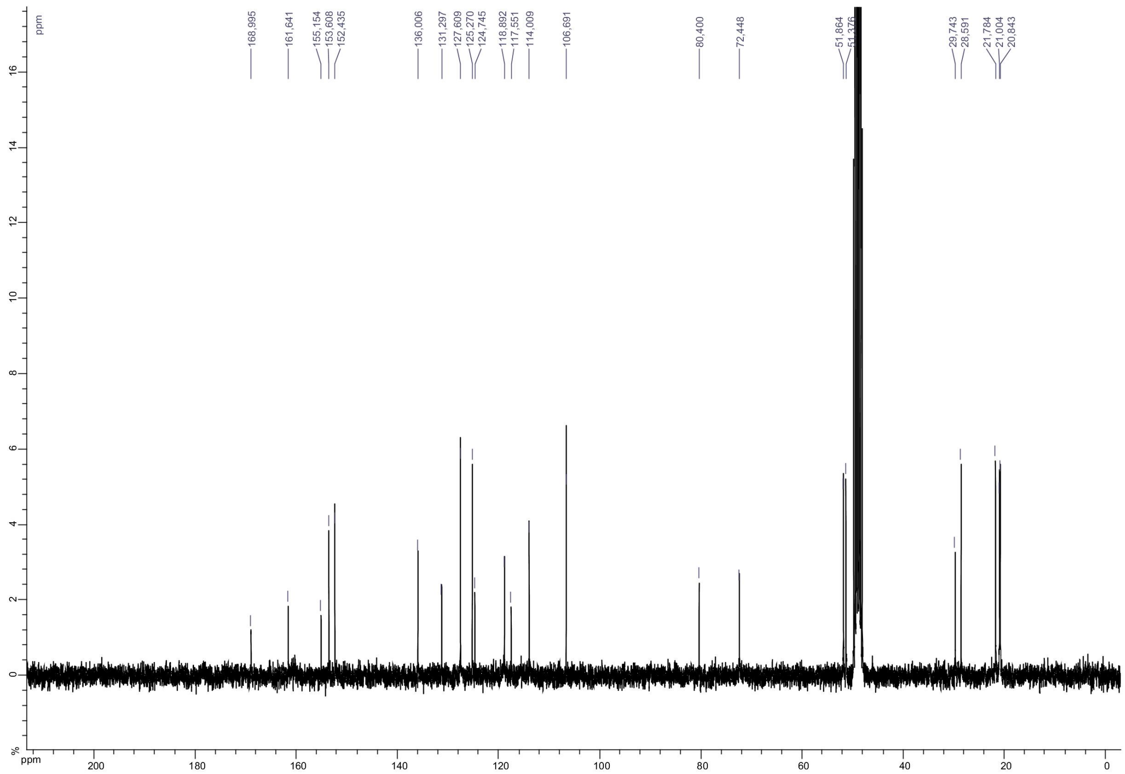

^{13}C NMR spectrum of **2b** (CD_3OD, 75 MHz)

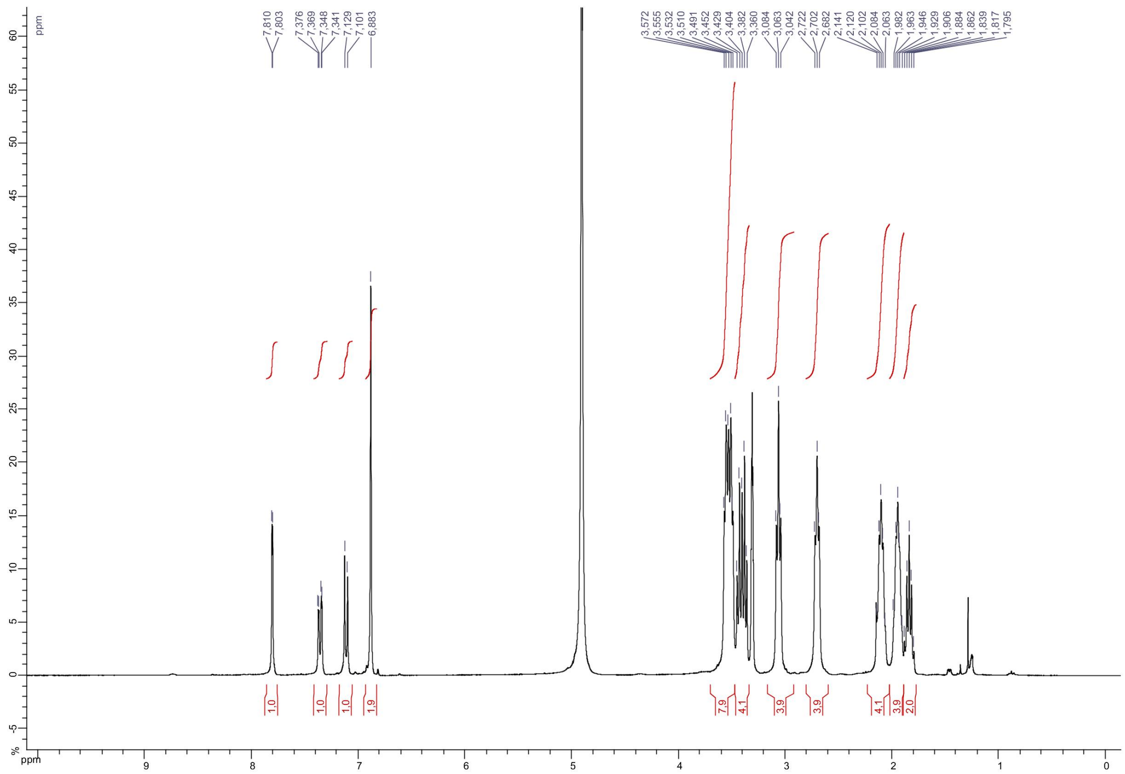

^{1}H NMR spectrum of **3b** (CD_3OD, 300 MHz)

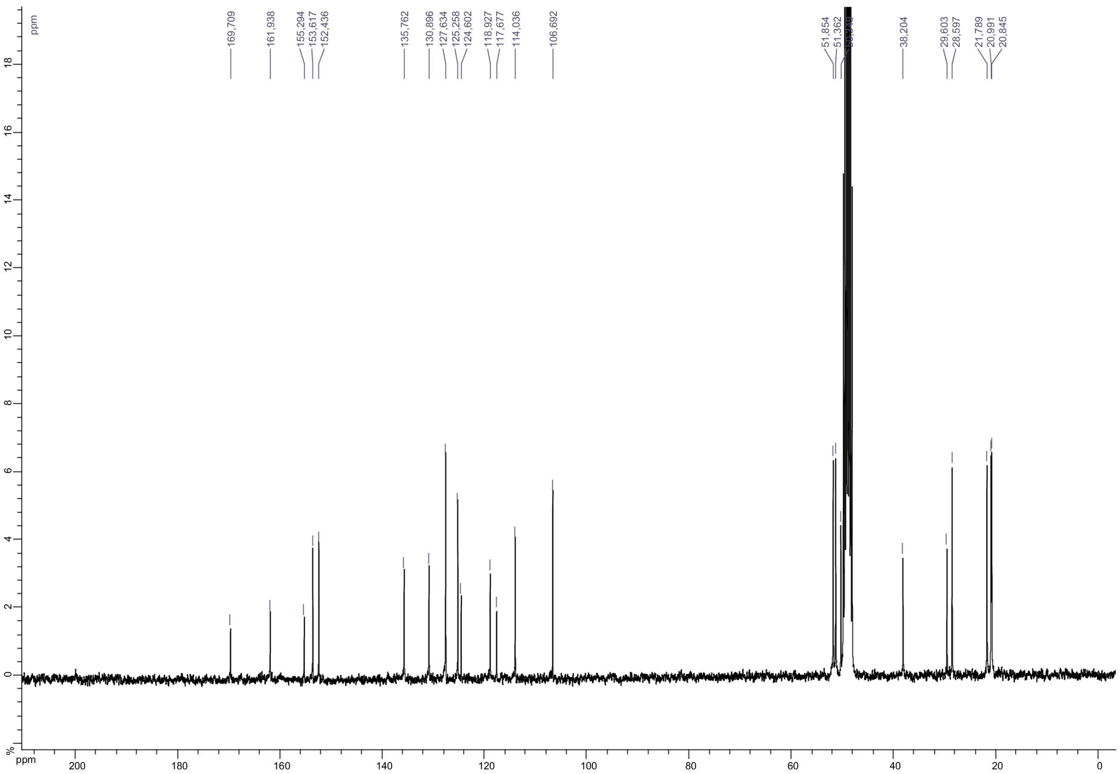

^{13}C NMR spectrum of **3b** (CD_3OD, 75 MHz)

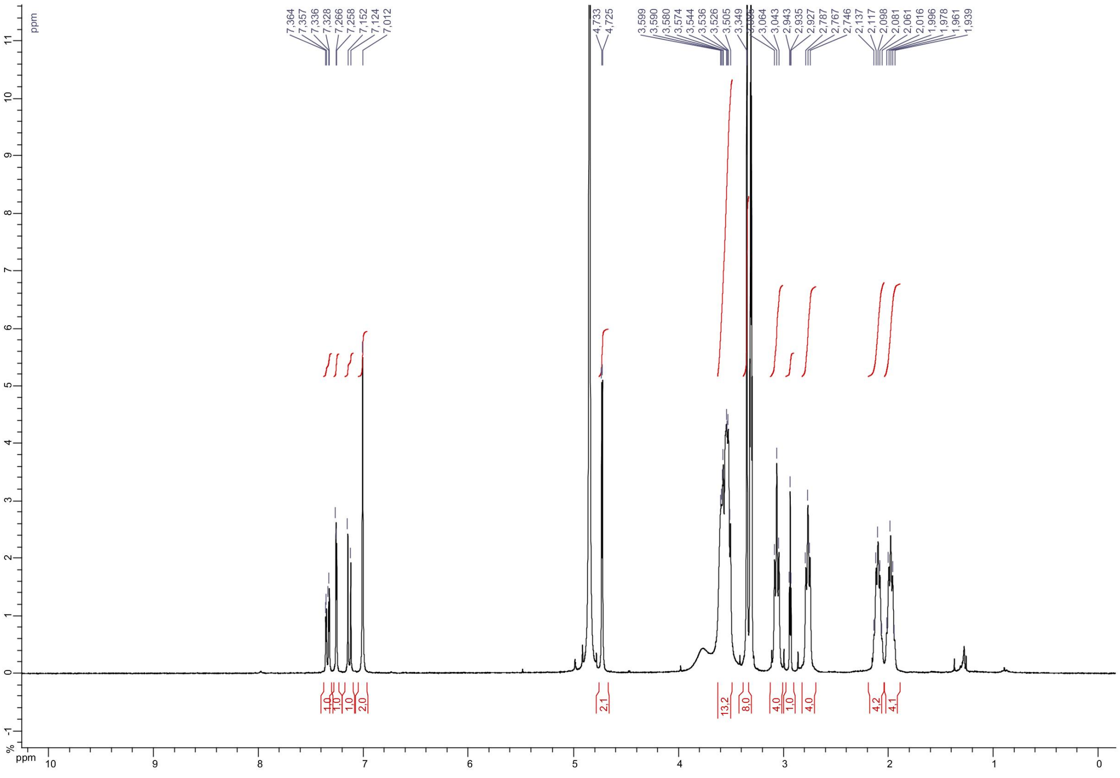

^{1}H NMR spectrum of **4b** (CD_3OD, 300 MHz)

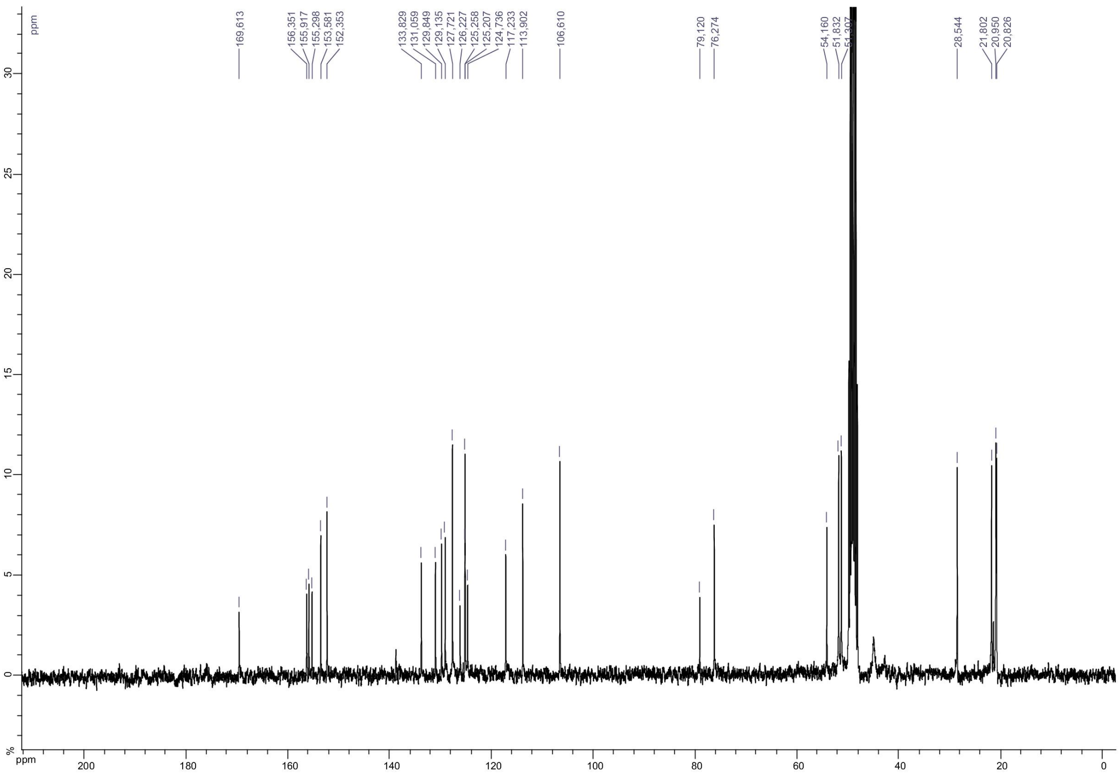

^{13}C NMR spectrum of **4b** (CD_3OD, 75 MHz)

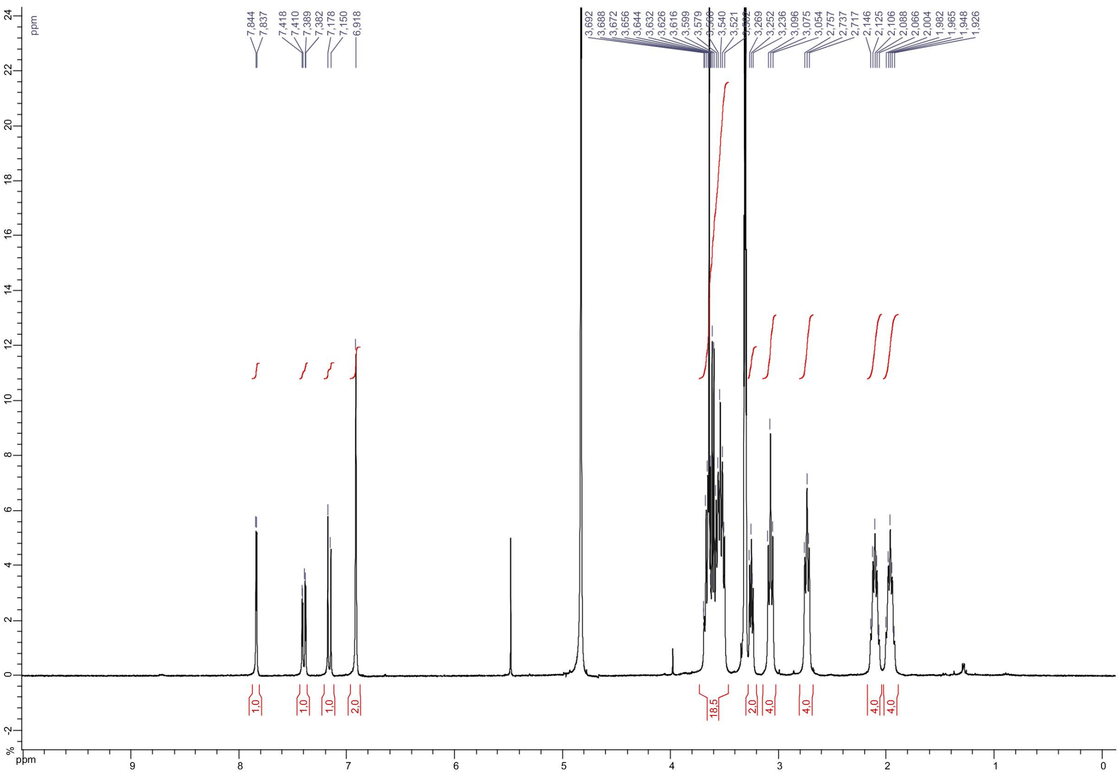

^{1}H NMR spectrum of **5b** (CD_3OD, 300 MHz)

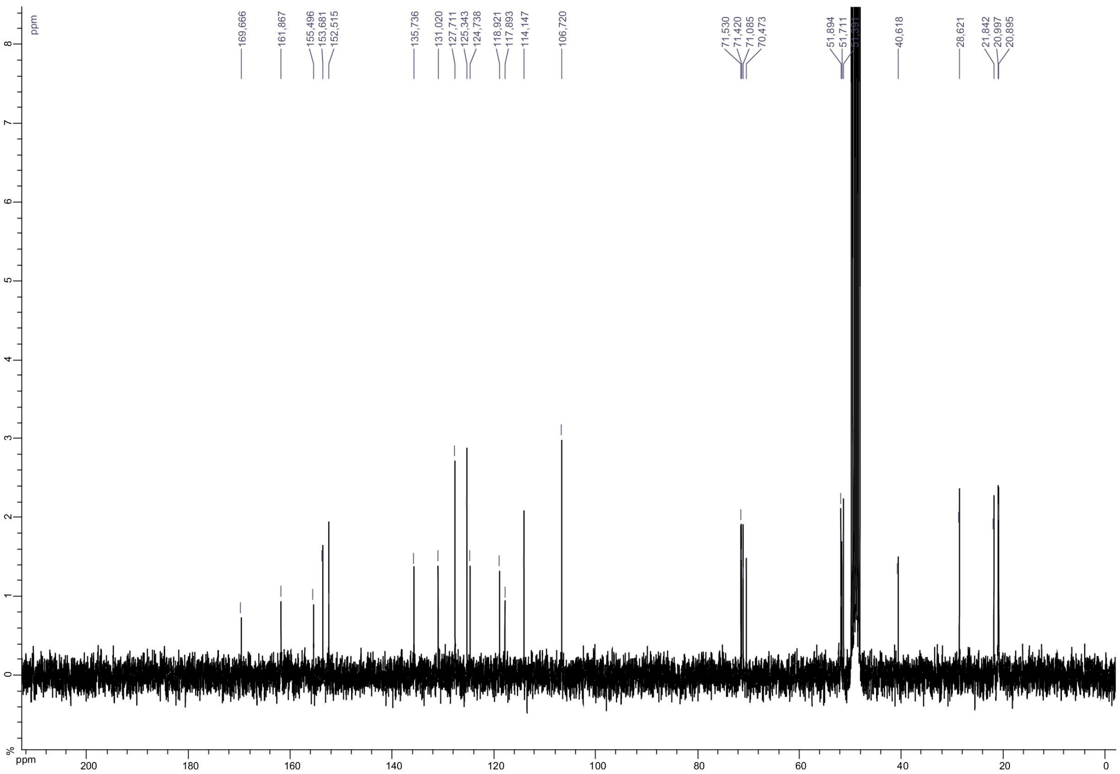

^{13}C NMR spectrum of **5b** (CD_3OD, 75 MHz)

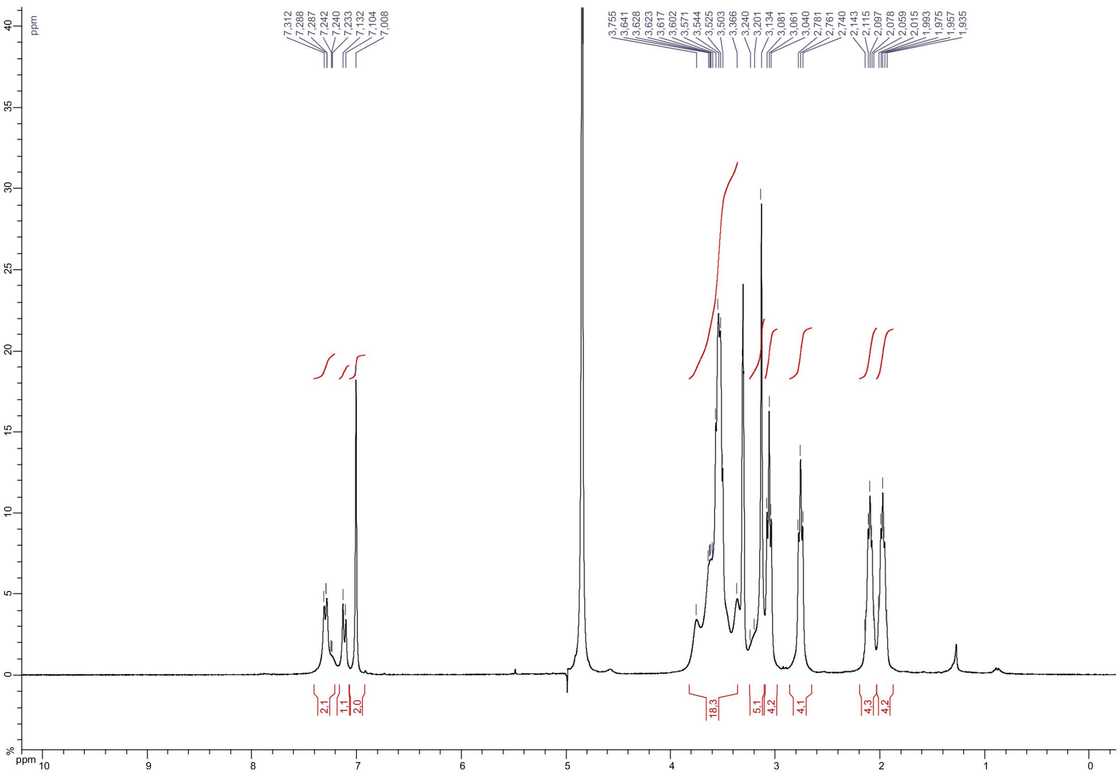

^{1}H NMR spectrum of **6b** (CD_3OD, 300 MHz)

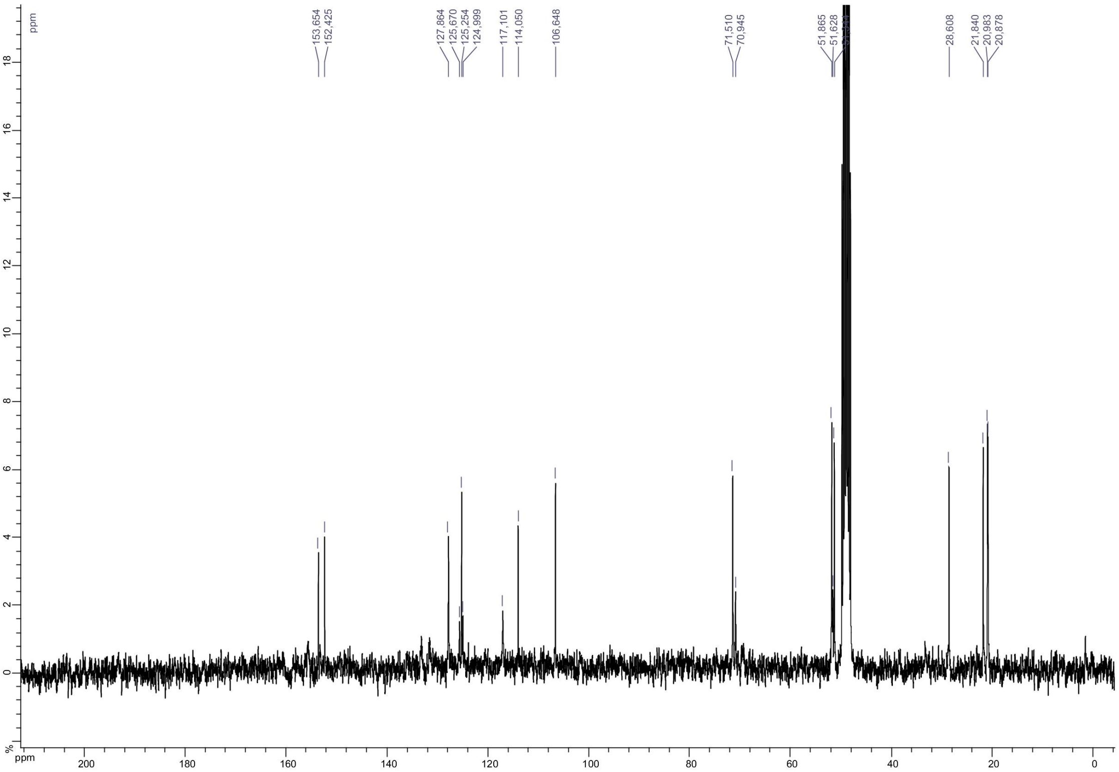

^{13}C NMR spectrum of **6b** (CD_3OD, 75 MHz)

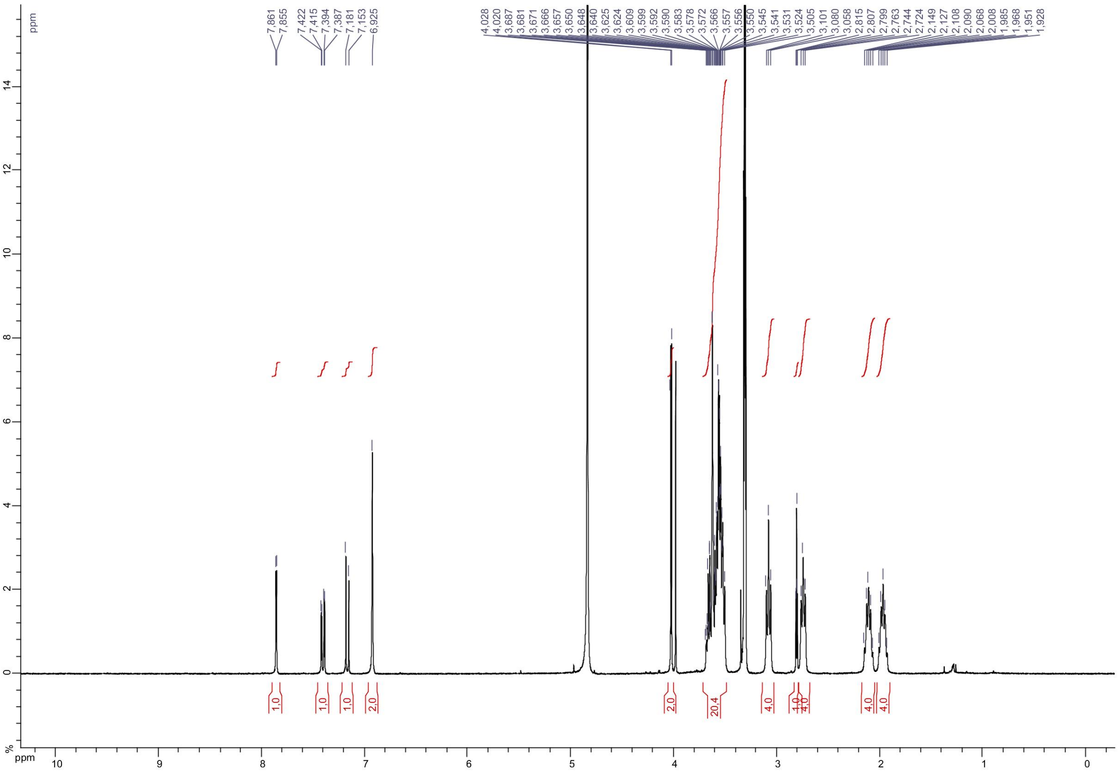

^{1}H NMR spectrum of **7b** (CD_3OD, 300 MHz)

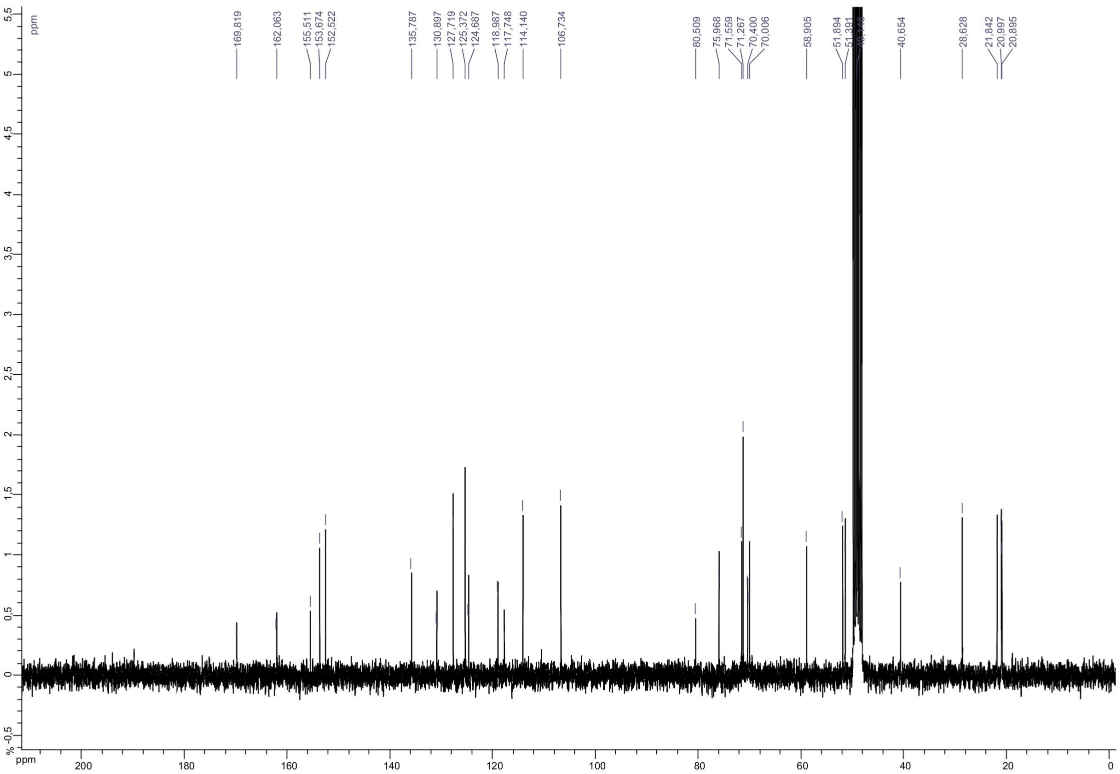

^{13}C NMR spectrum of **7b** (CD_3OD, 75 MHz)

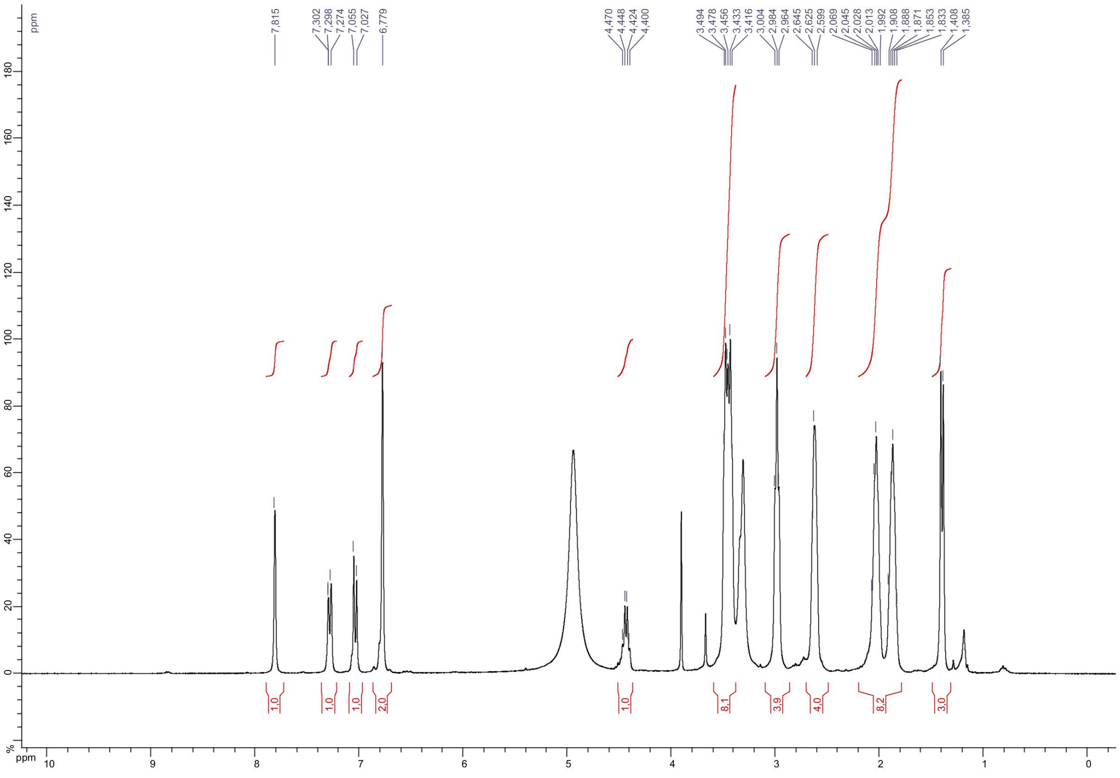

^{1}H NMR spectrum of **8b** (CD_3OD, 300 MHz)

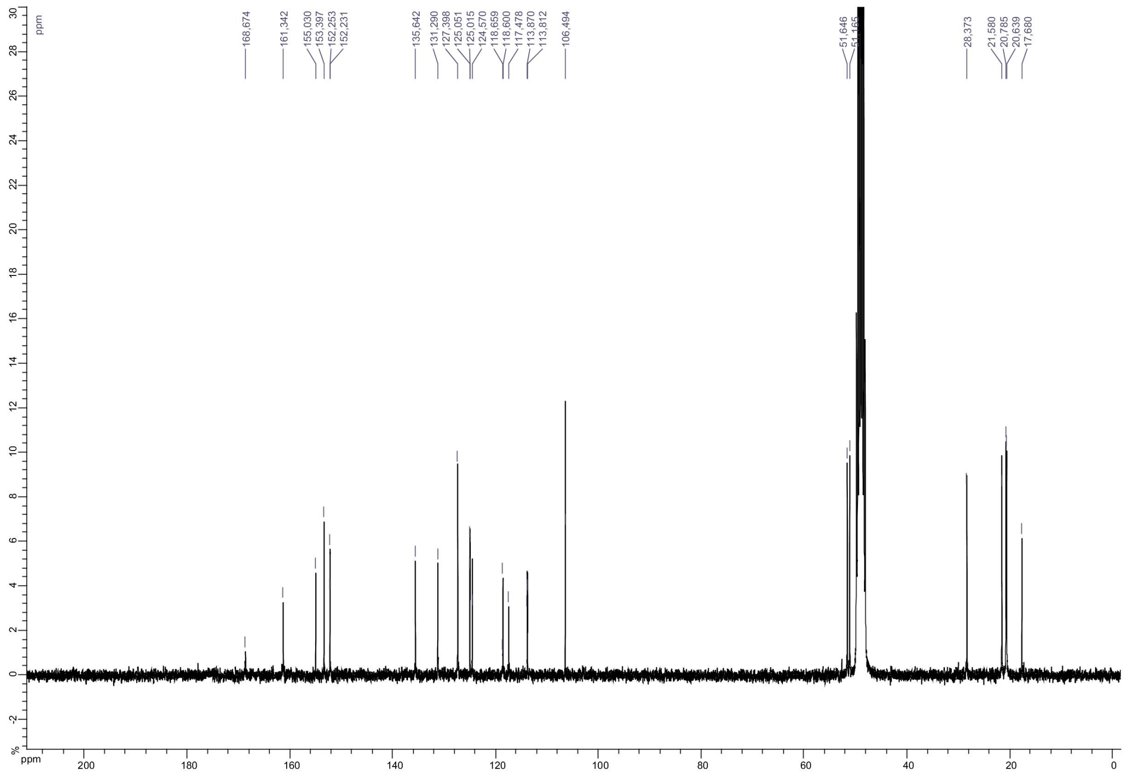

^{13}C NMR spectrum of **8b** (CD_3OD, 75 MHz)

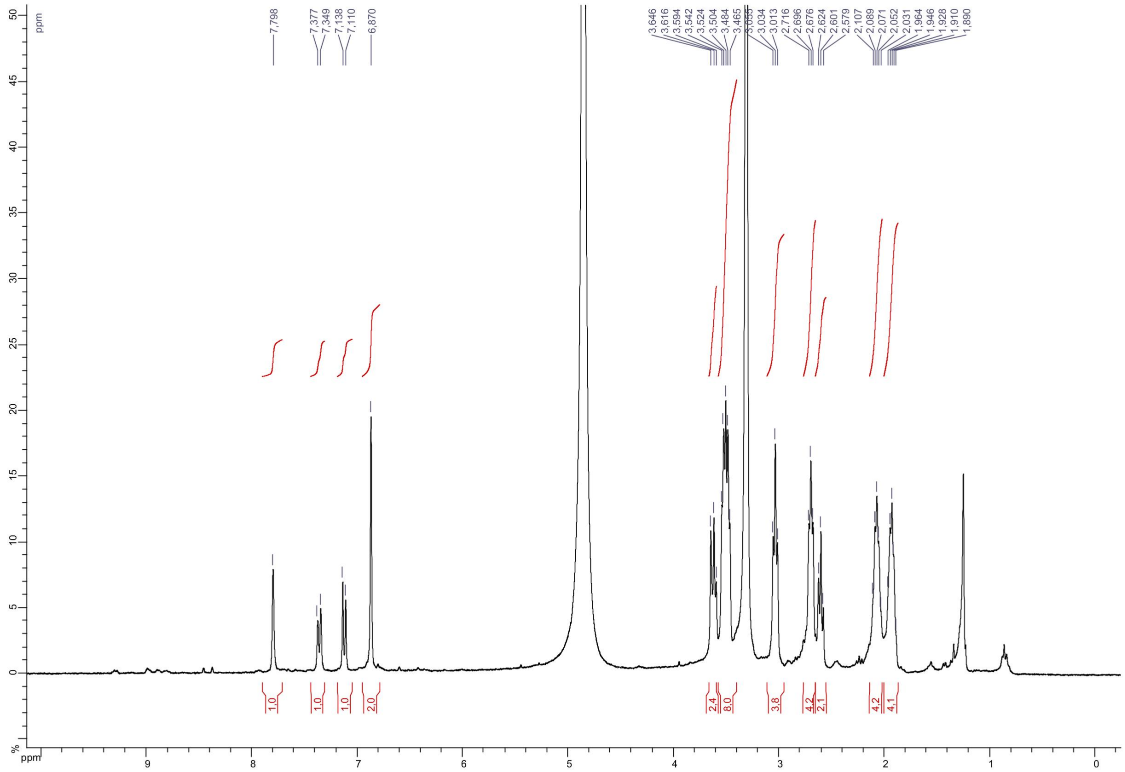

^{1}H NMR spectrum of **9b** (CD_3OD, 300 MHz)

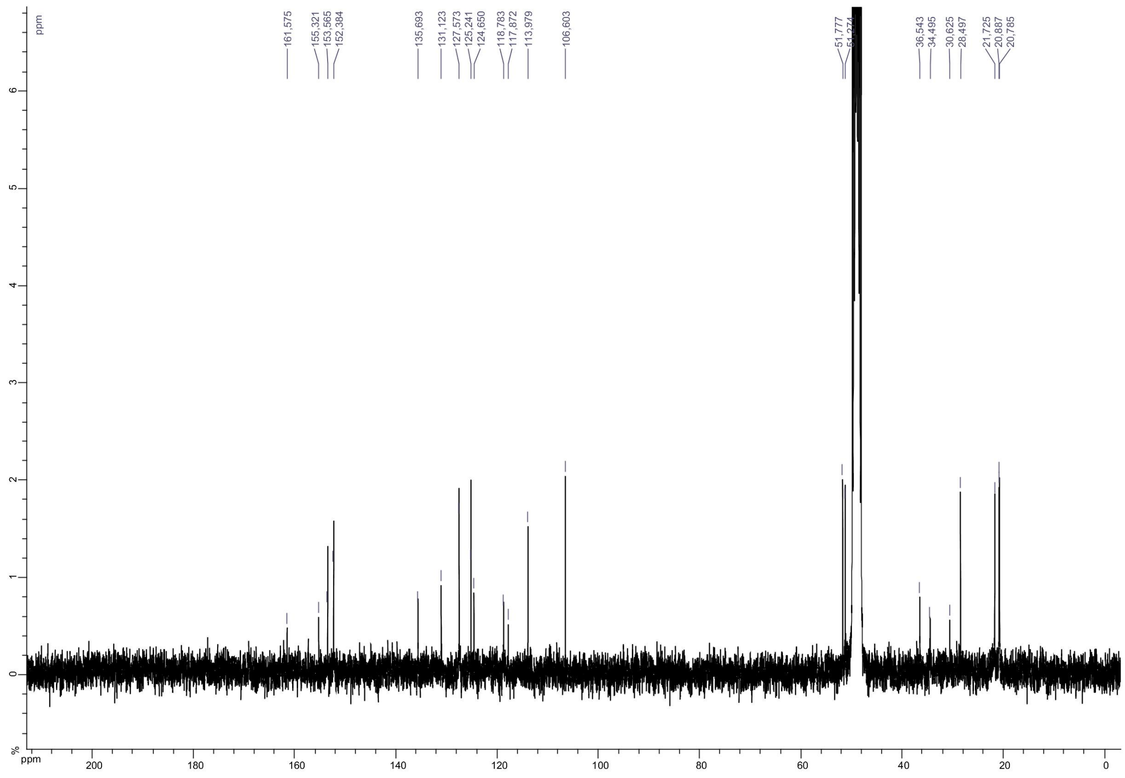

^{13}C NMR spectrum of **9b** (CD_3OD, 75 MHz)

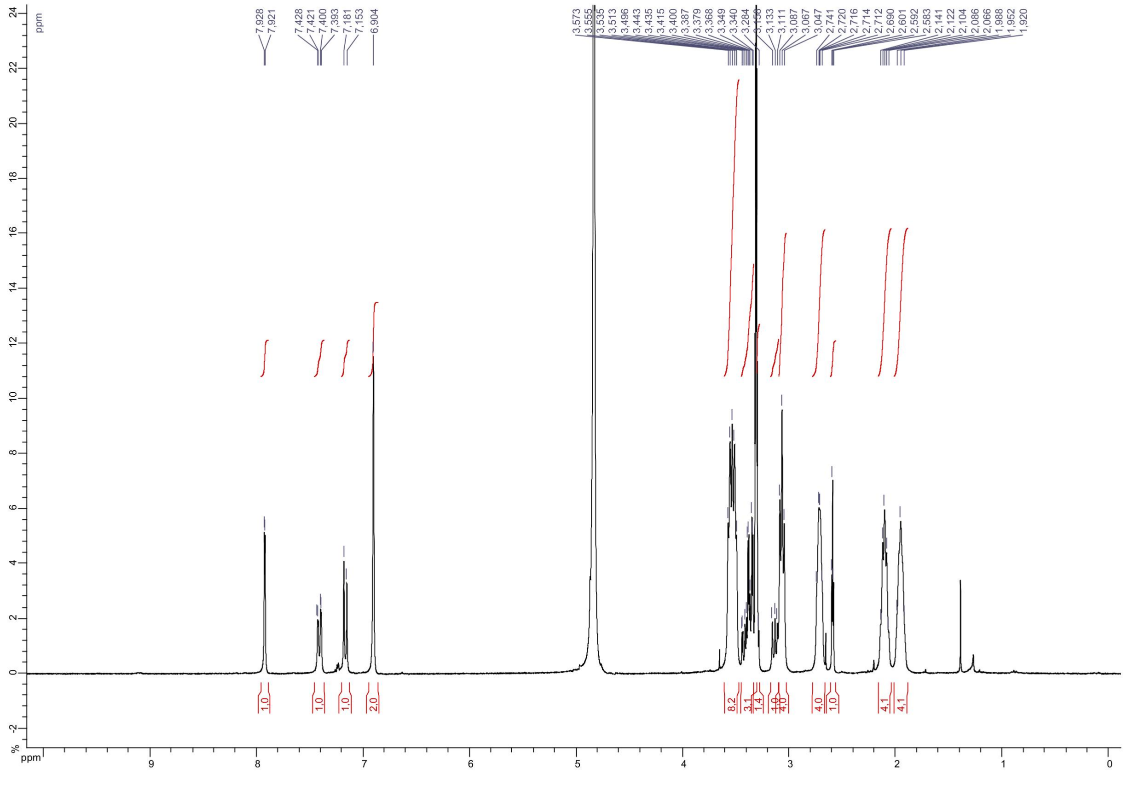

^{1}H NMR spectrum of **10b** (CD_3OD, 300 MHz)

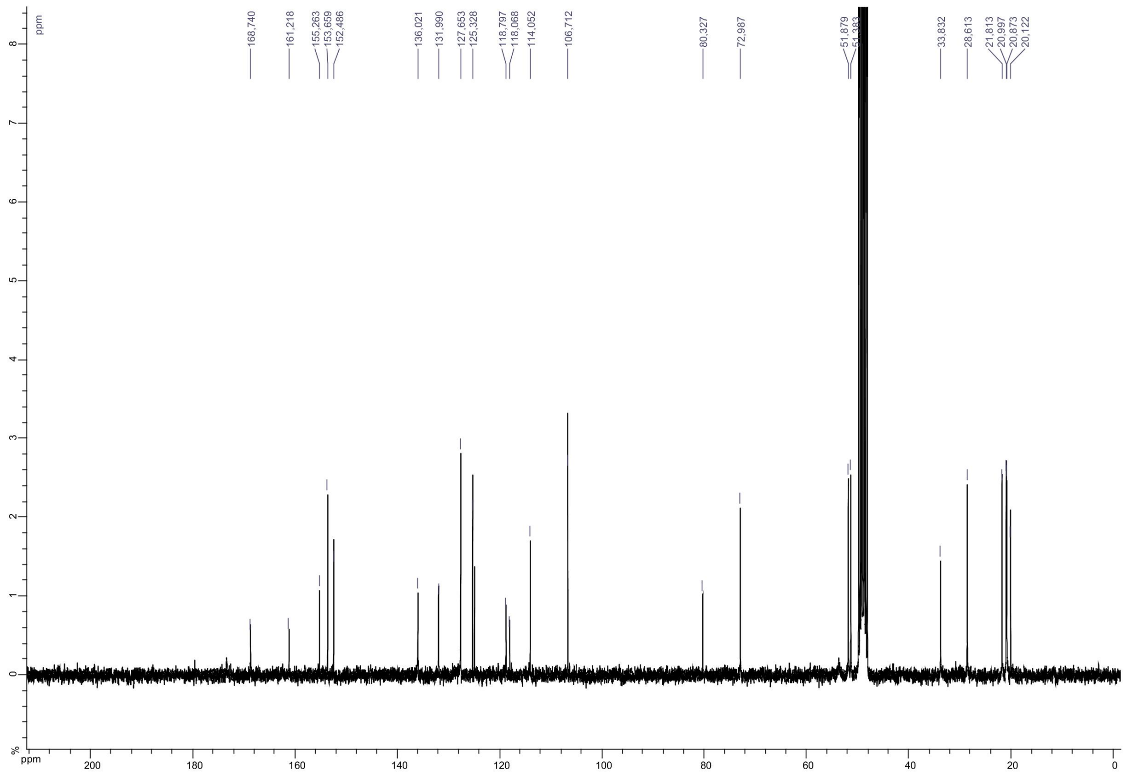

^{13}C NMR spectrum of **10b** (CD_3OD, 75 MHz)

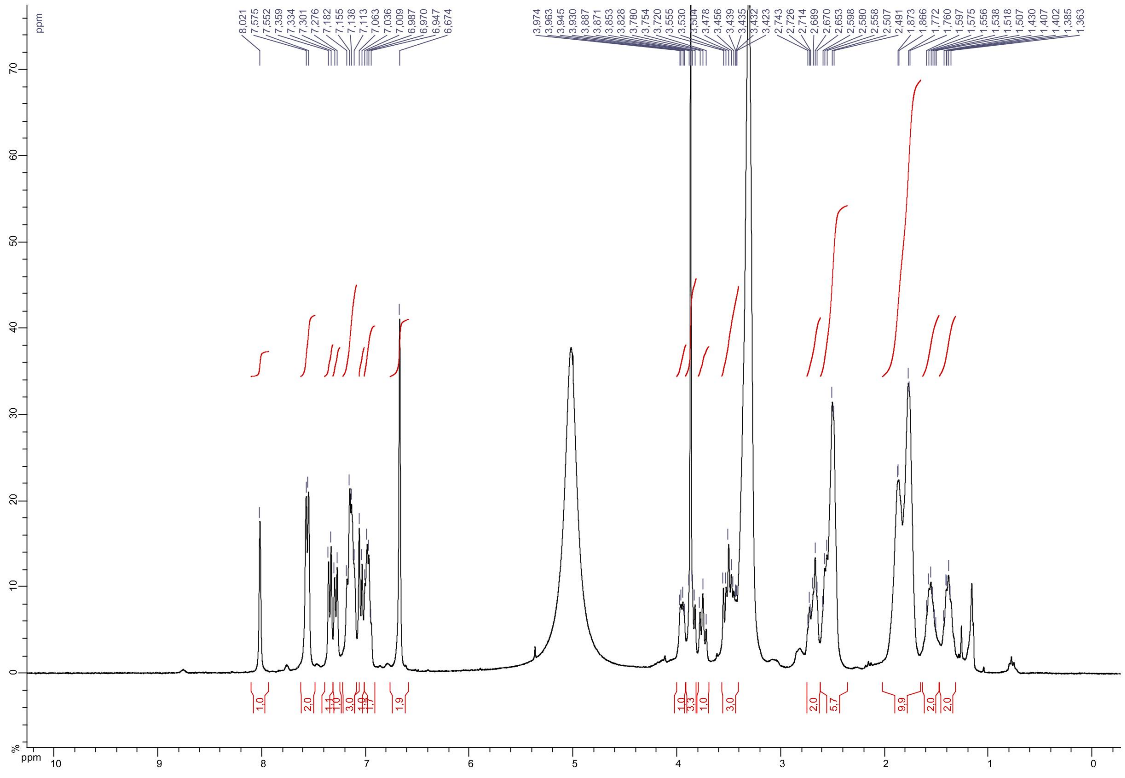

^{1}H NMR spectrum of **11b** (CD_3OD, 300 MHz)

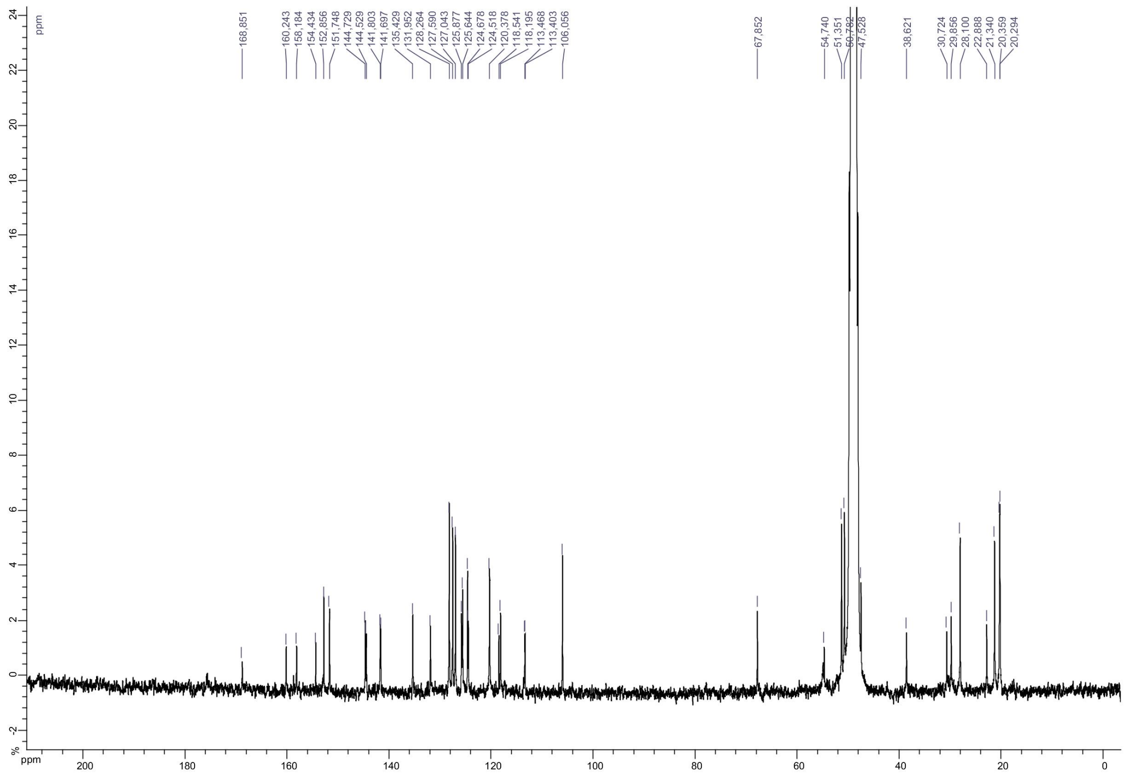

^{13}C NMR spectrum of **11b** (CD_3OD, 75 MHz)

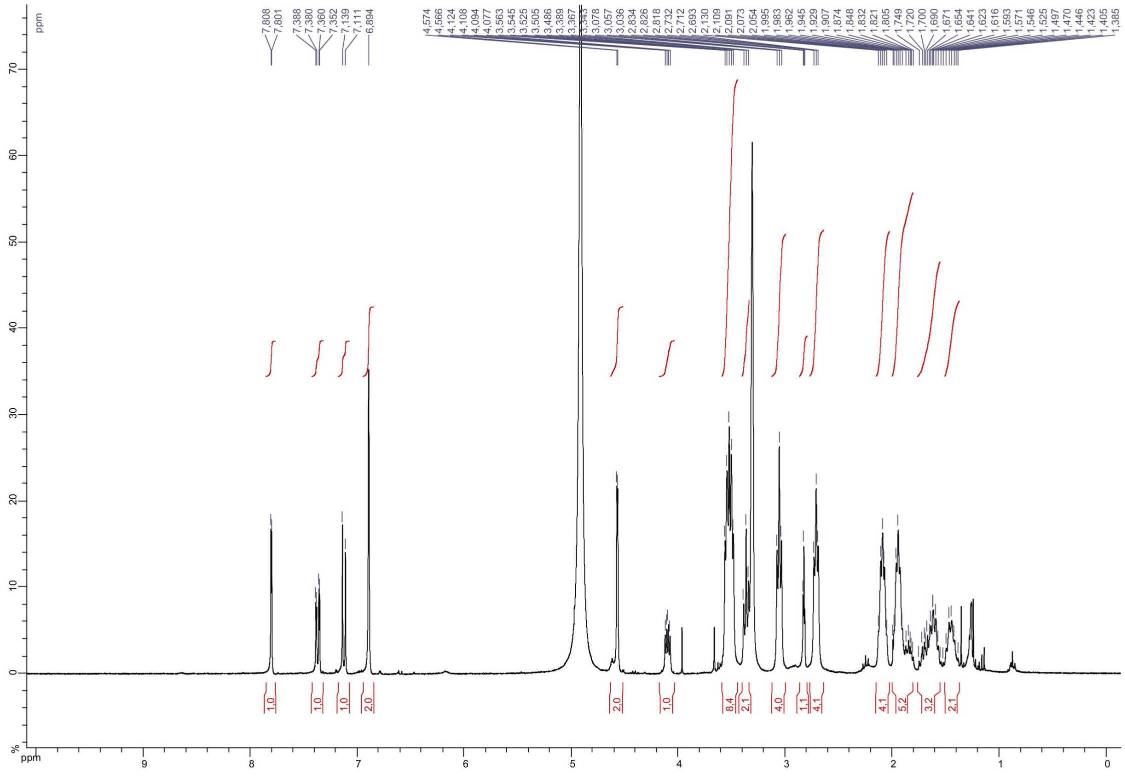

^{1}H NMR spectrum of **12b** (CD_3OD, 300 MHz)

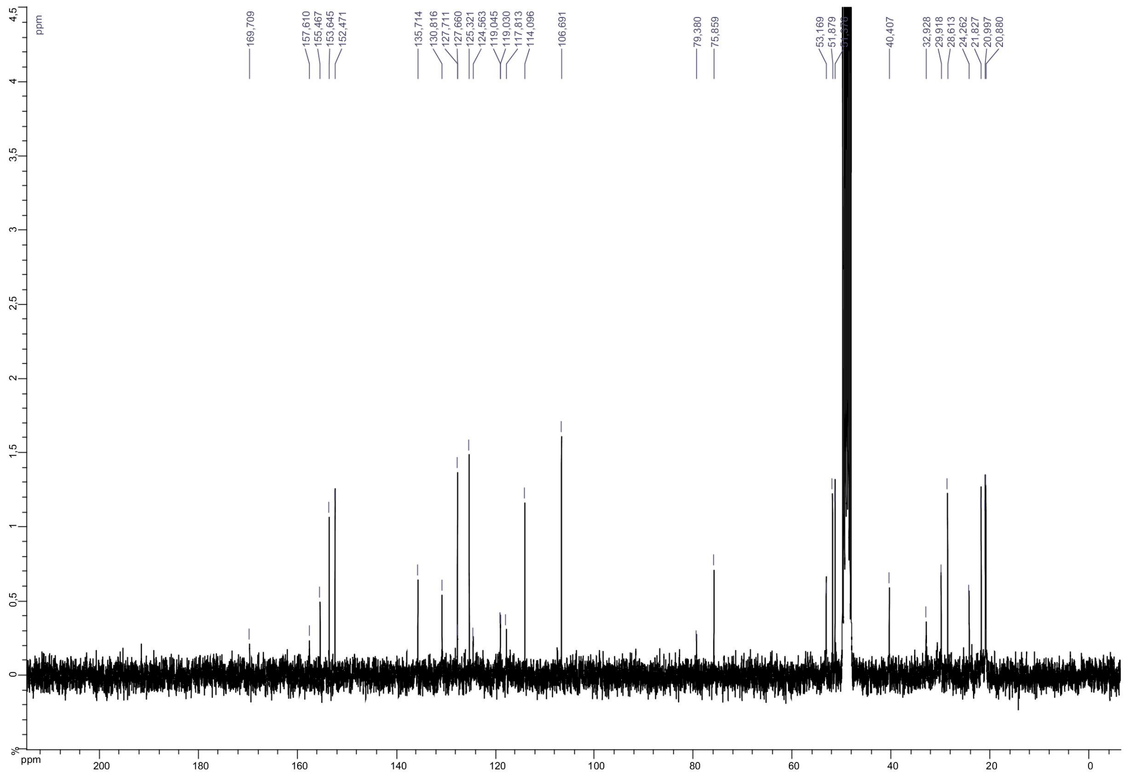

^{13}C NMR spectrum of **12b** (CD_3OD, 75 MHz)

HRMS spectra of functionalisable H-Rubies and their intermediates

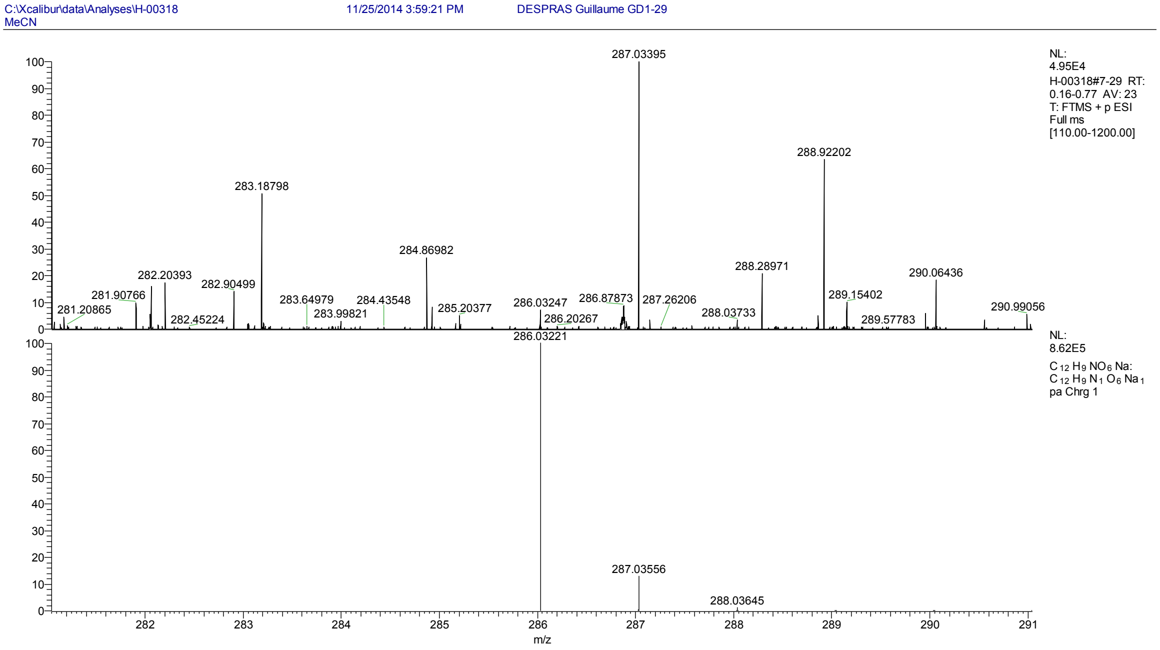

HRMS spectrum of **1**

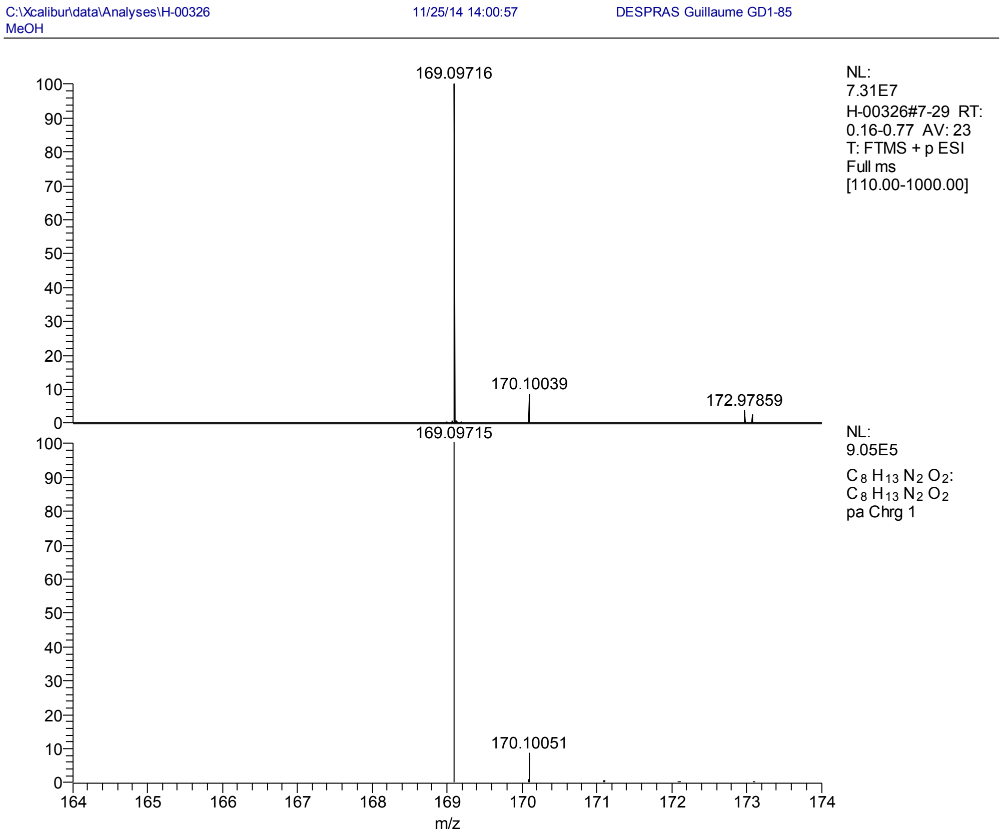

HRMS spectrum of **4-((prop-2-yn-1-yloxy)carbonyl)piperazin-1-ium**

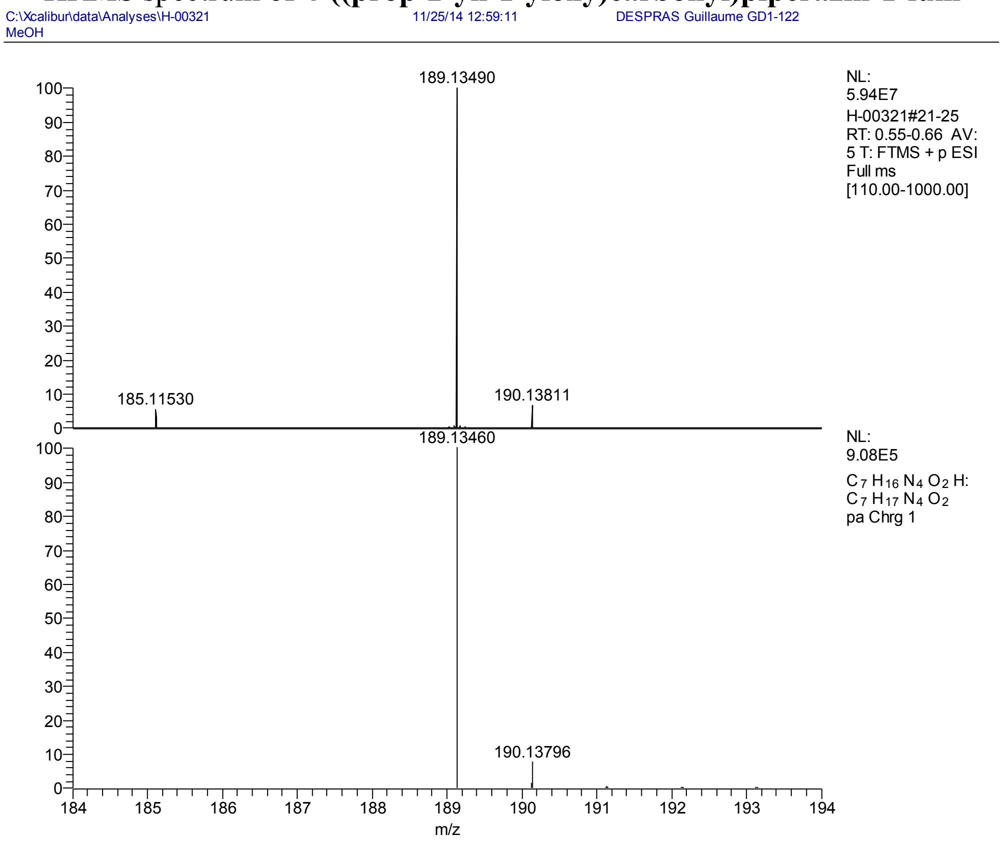

HRMS spectrum of **2-(2-(2-azidoethoxy)ethoxy)-N-methylethanamine**

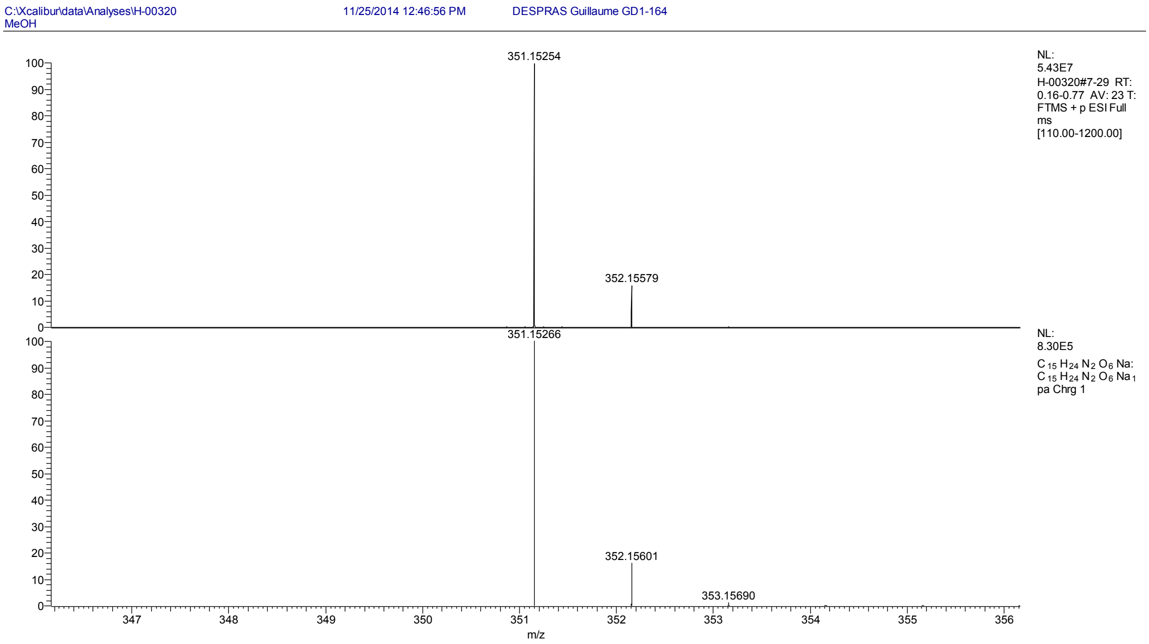

HRMS spectrum of **6-((tert-butoxycarbonyl)amino)-2-(((prop-2-yn-1-yloxy)carbonyl)amino)hexanoic acid**

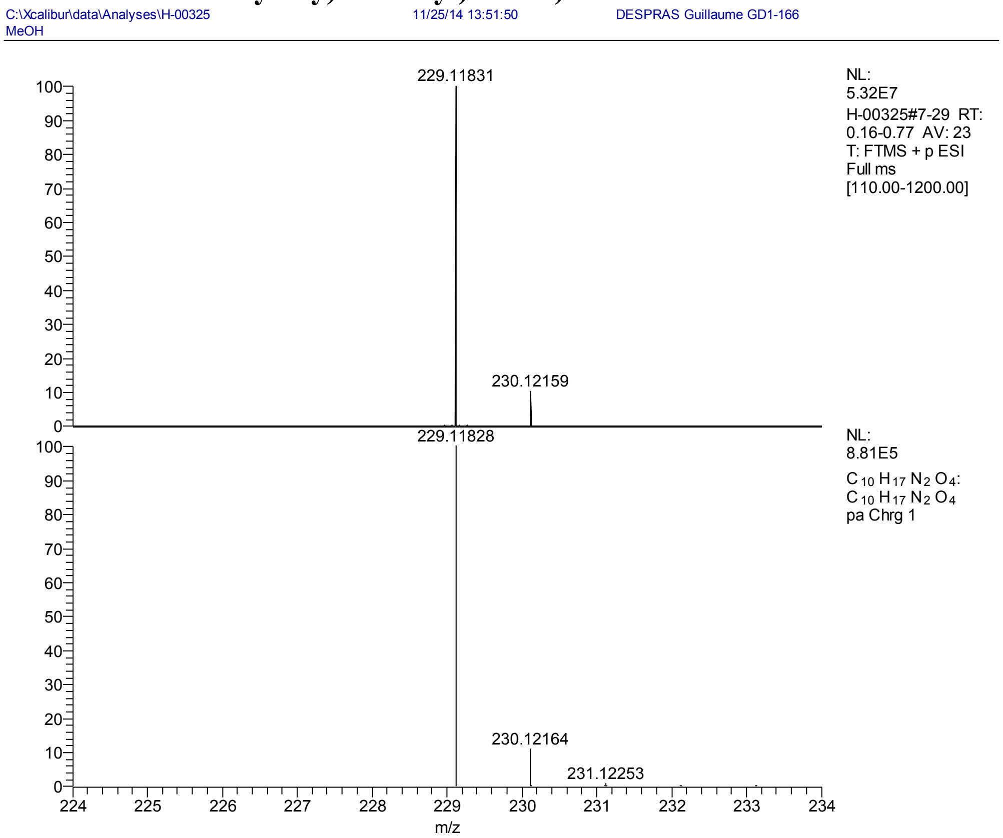

HRMS spectrum of **5-carboxy-5-(((prop-2-yn-1-yloxy)carbonyl)amino)pentan-1-aminium**

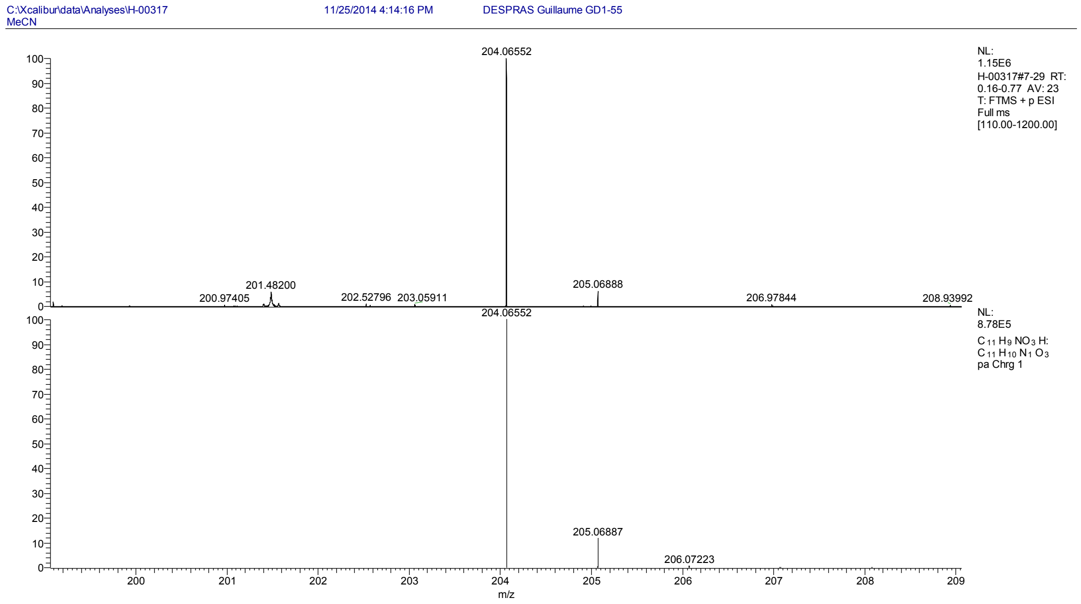

HRMS spectrum of **2a**

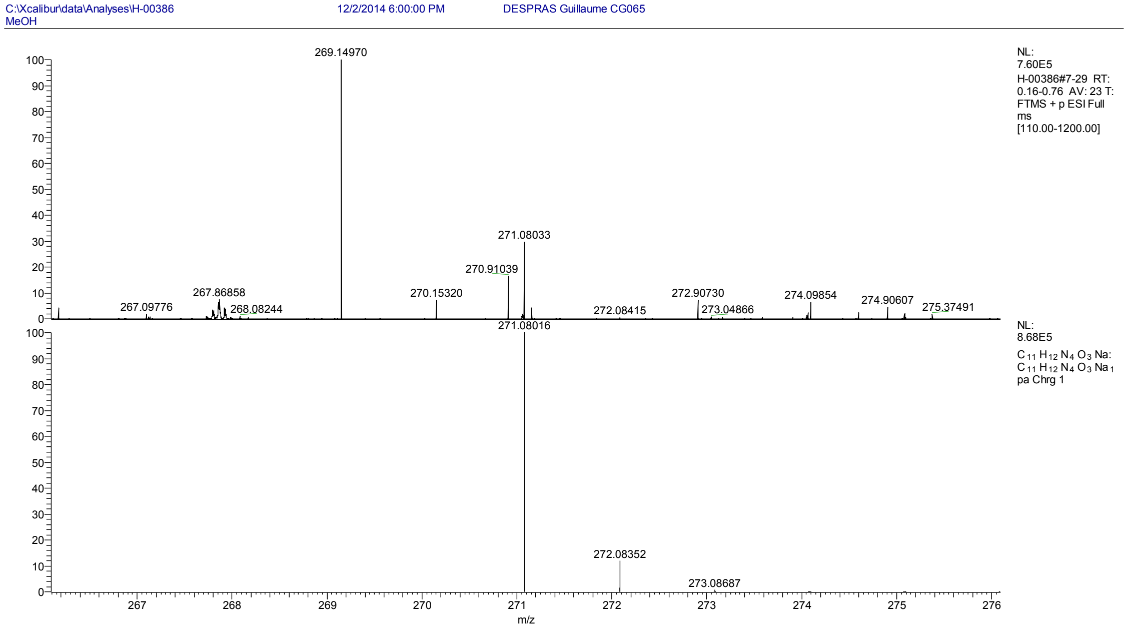

HRMS spectrum of **2b**

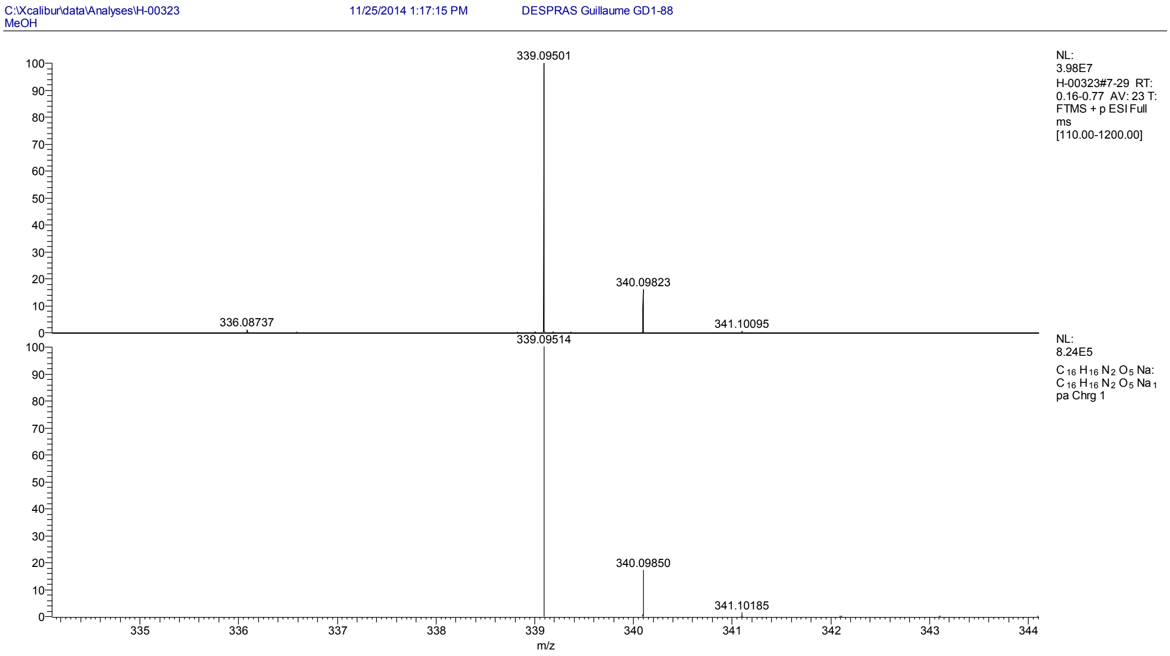

HRMS spectrum of **2d**

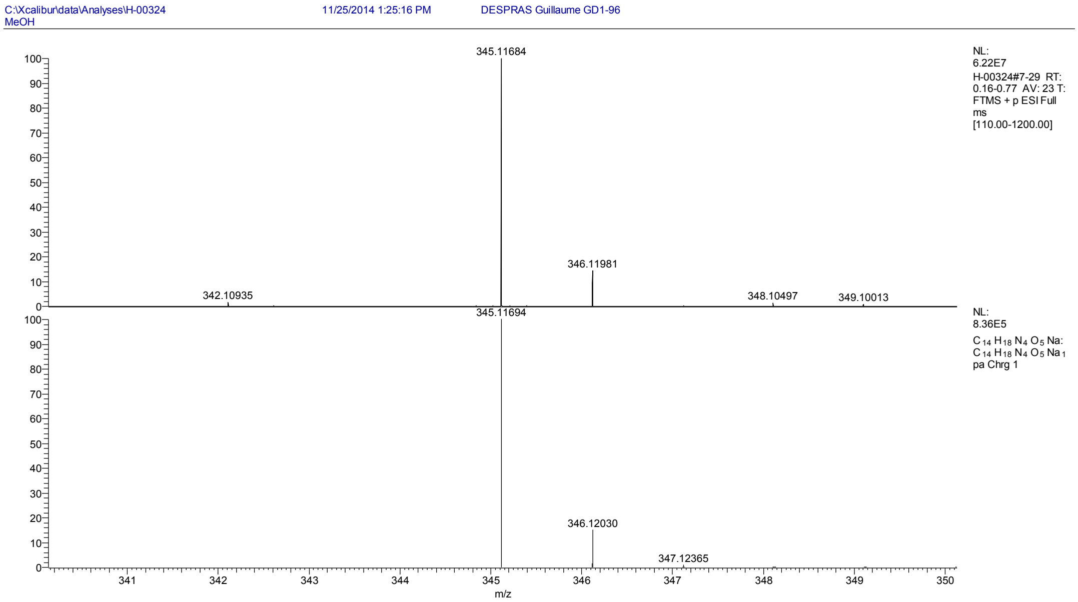

HRMS spectrum of **2e**

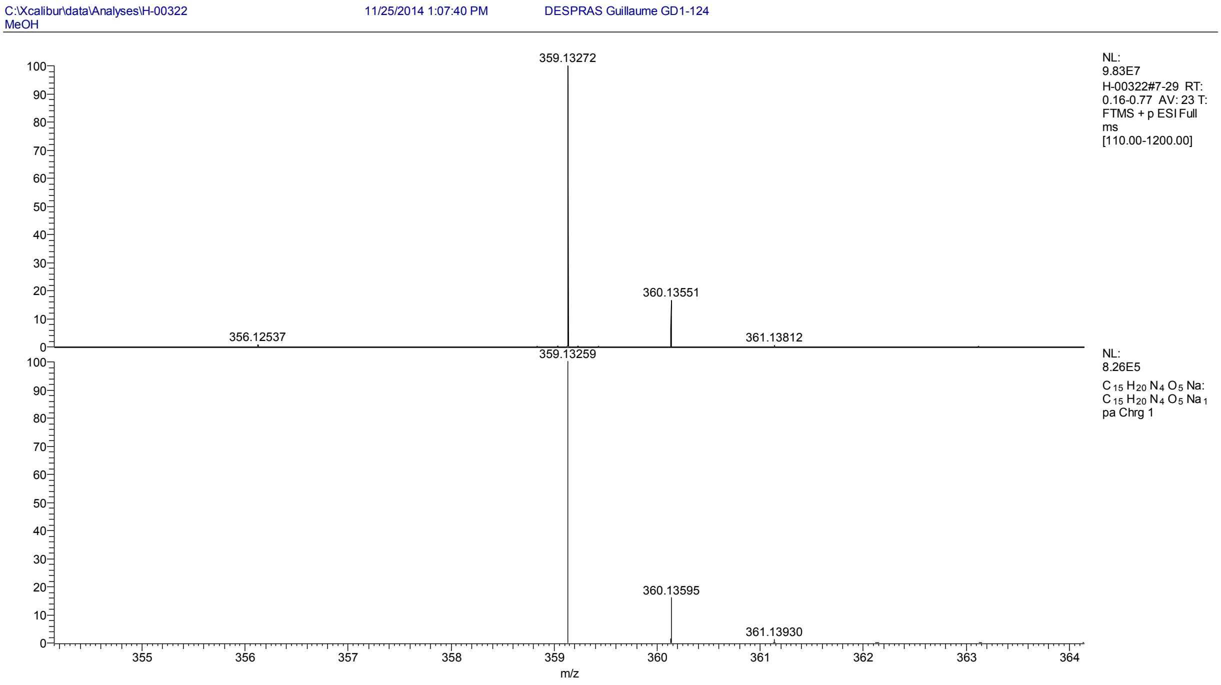

HRMS spectrum of **2f**

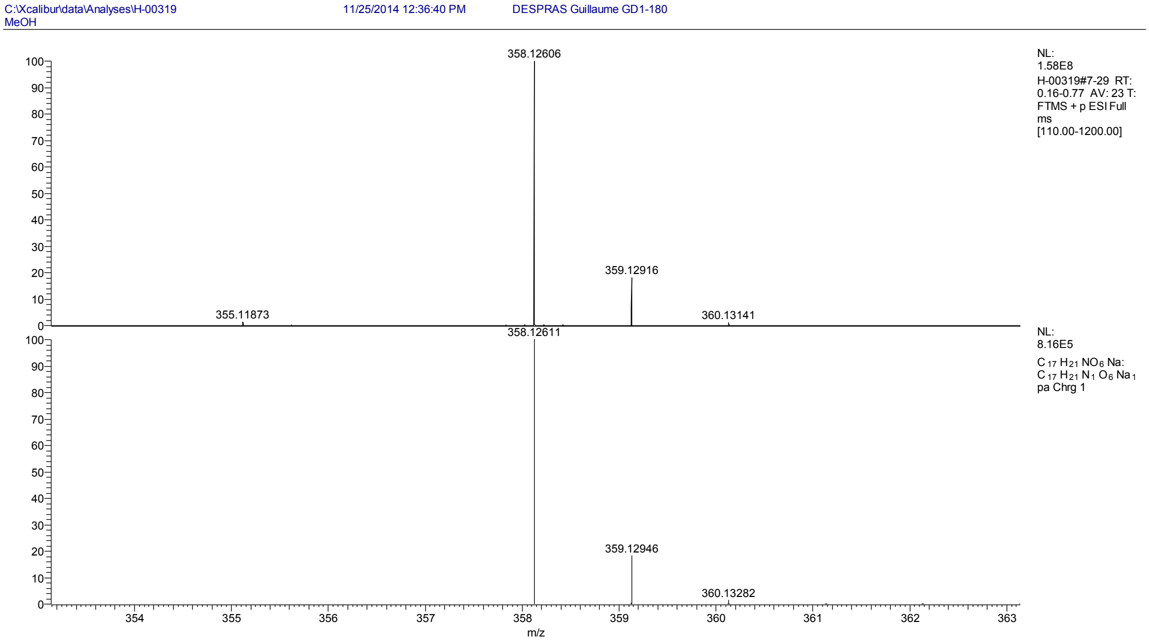

HRMS spectrum of **2g**

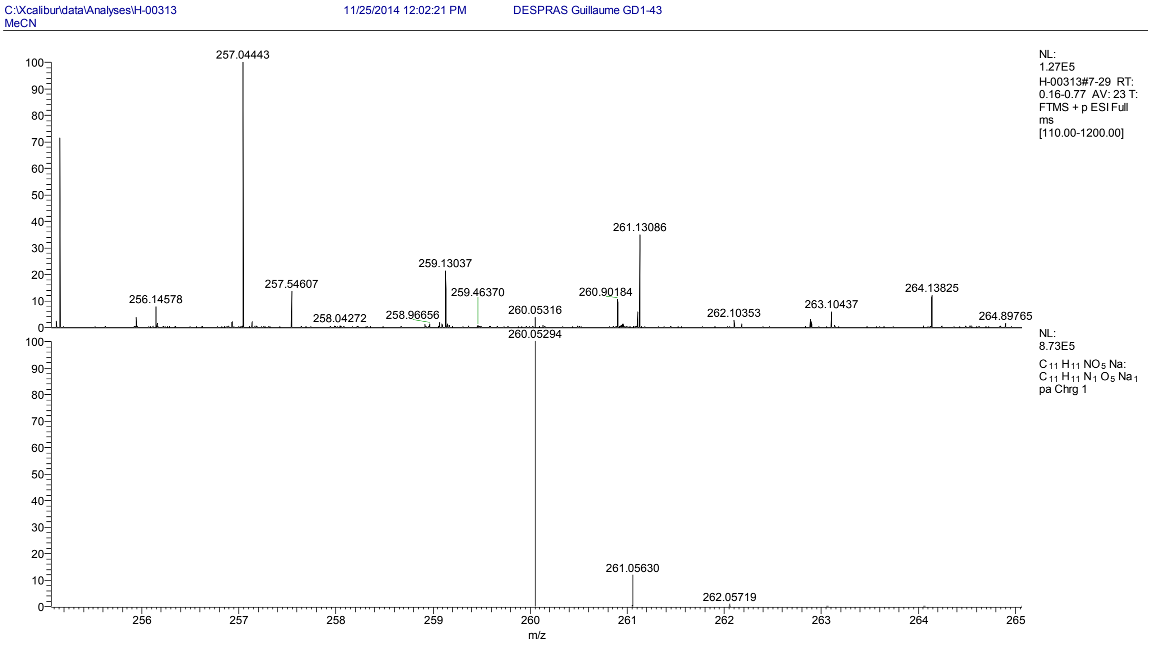

HRMS spectrum of **2h**

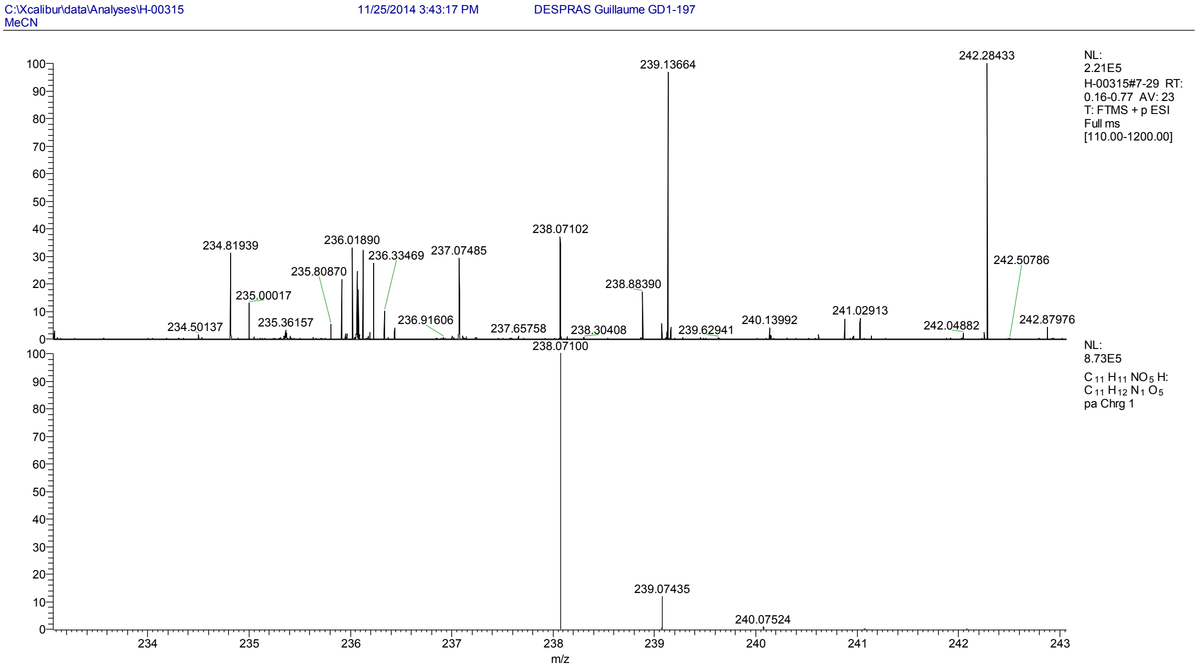

HRMS spectrum of **2i**

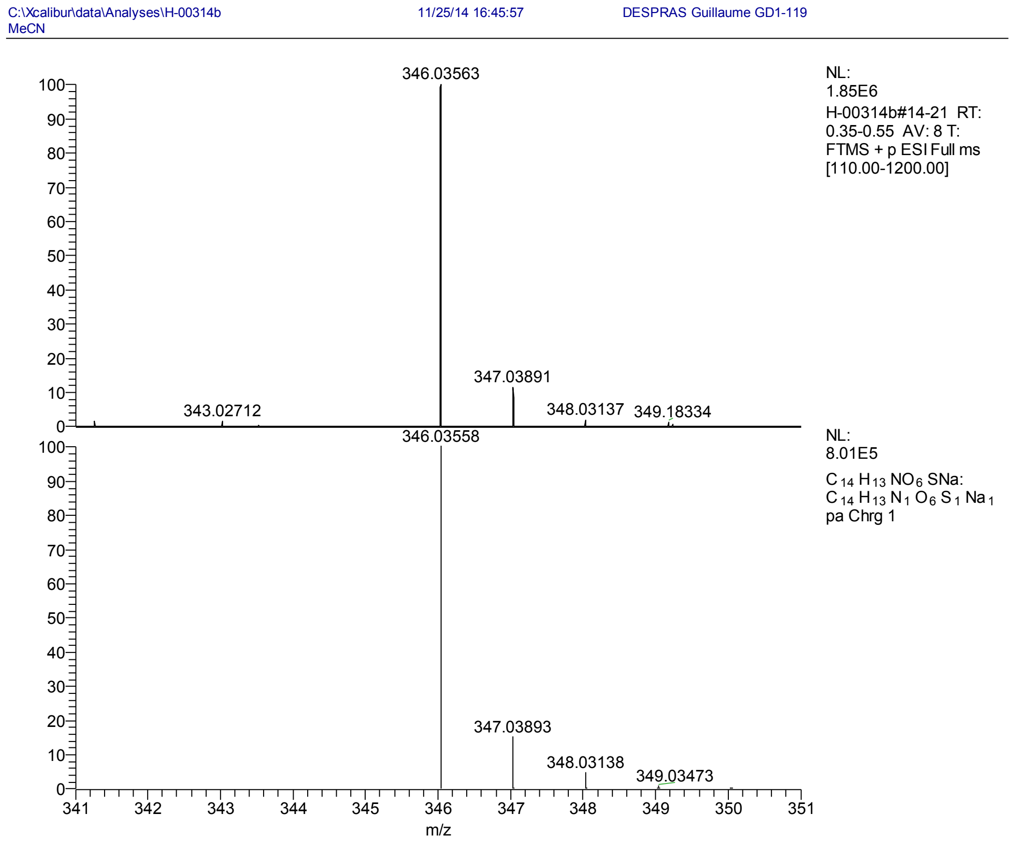

HRMS spectrum of **2j (sulfoxyde)**

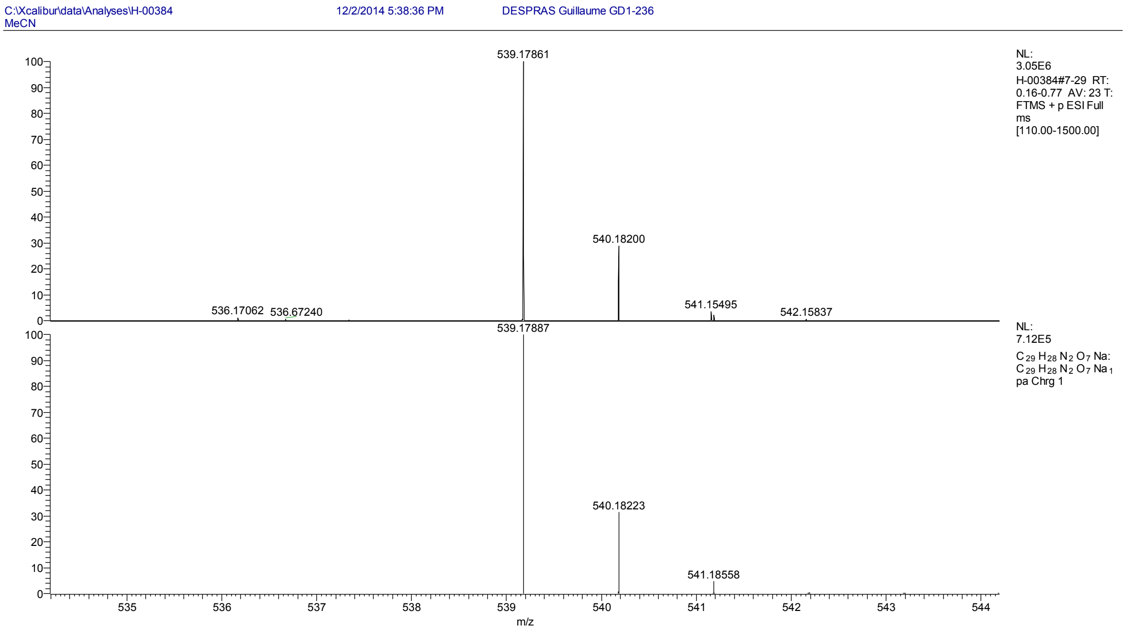

HRMS spectrum of **2k**

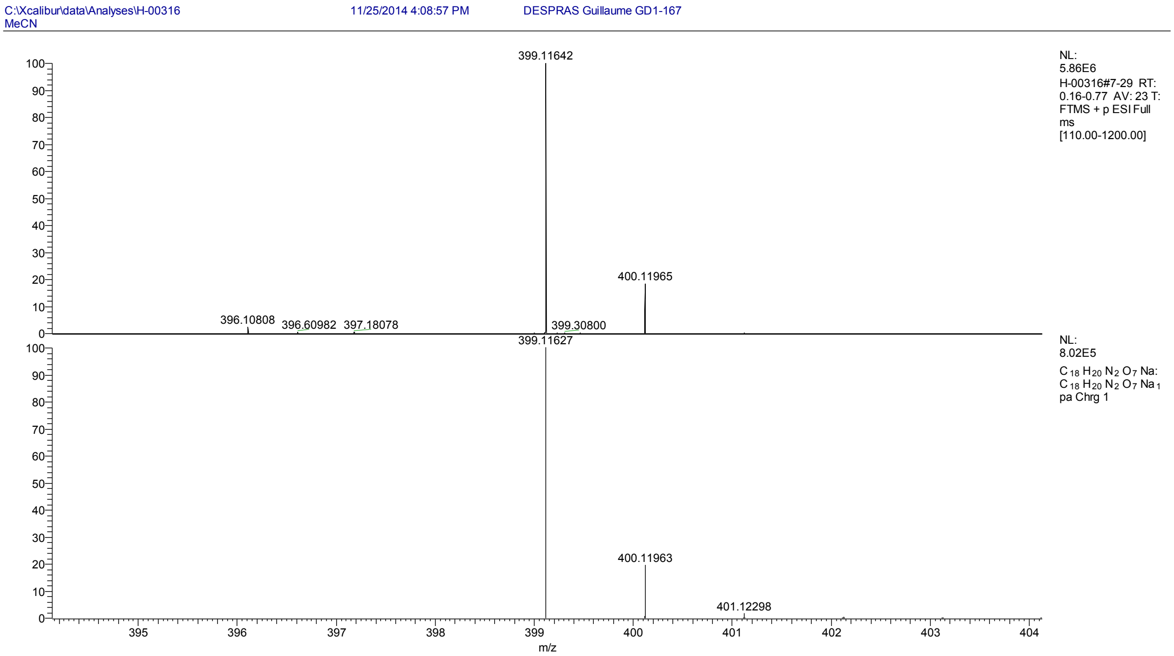

HRMS spectrum of **2l**

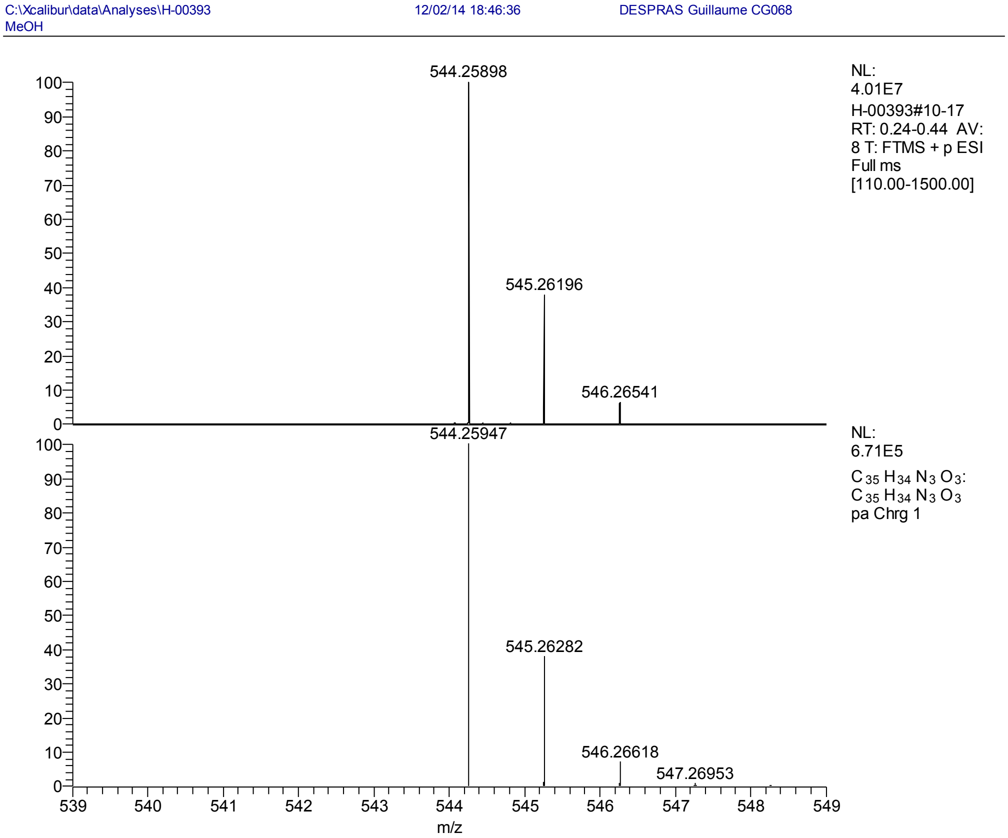

HRMS spectrum of **HR-A**

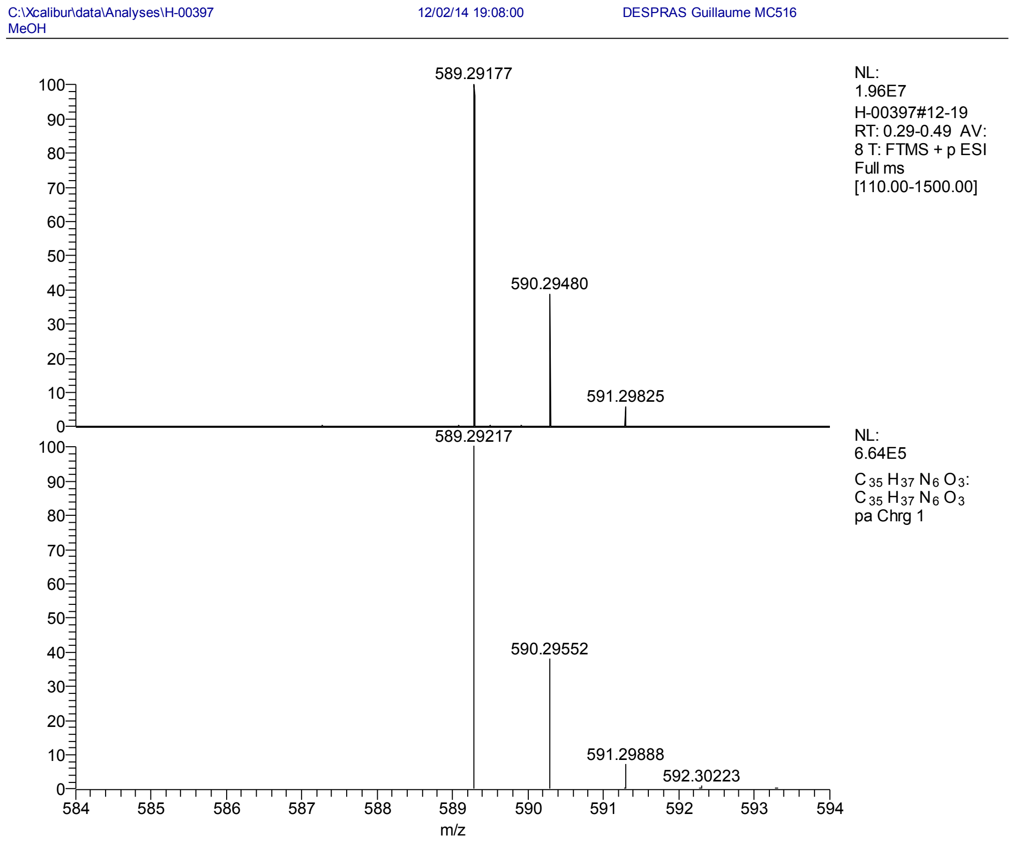

HRMS spectrum of **HR-N3**

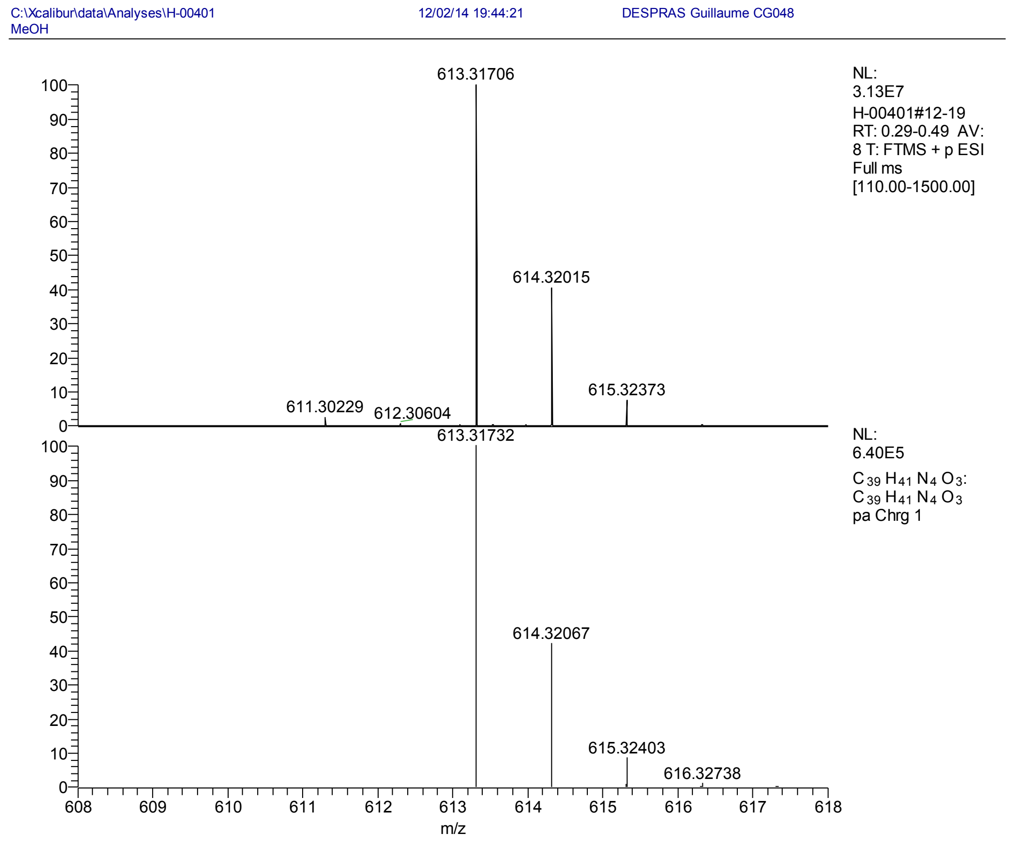

HRMS spectrum of **HR-PiA**

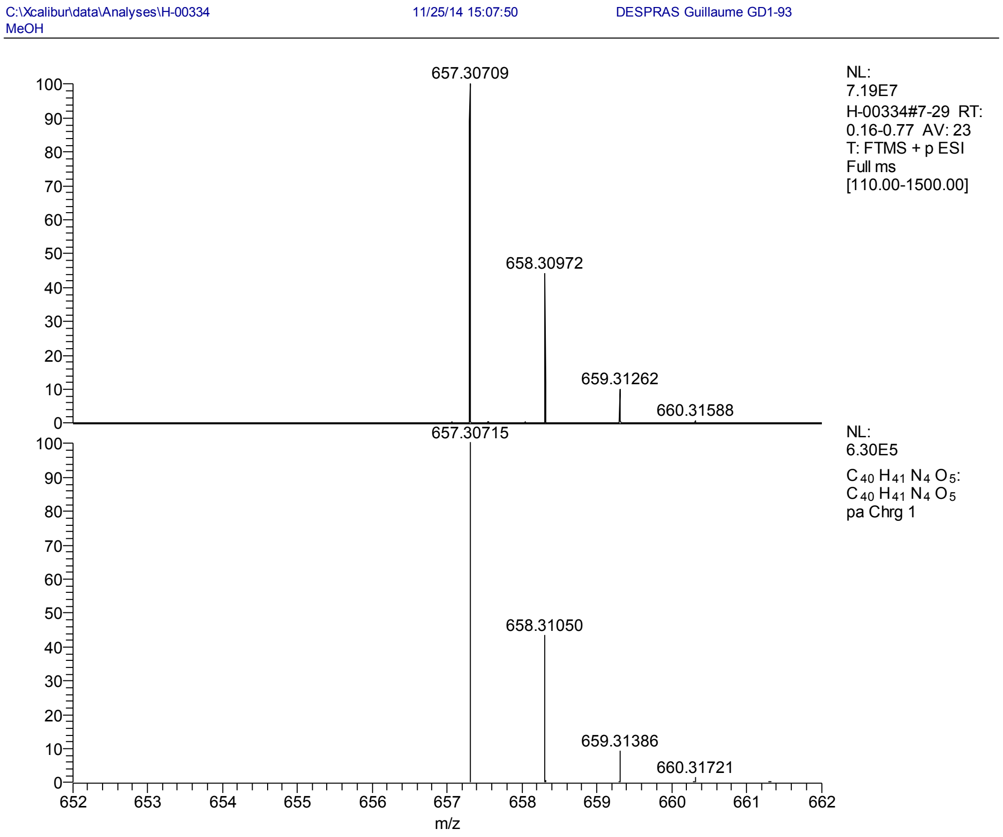

HRMS spectrum of **HR-PiAC**

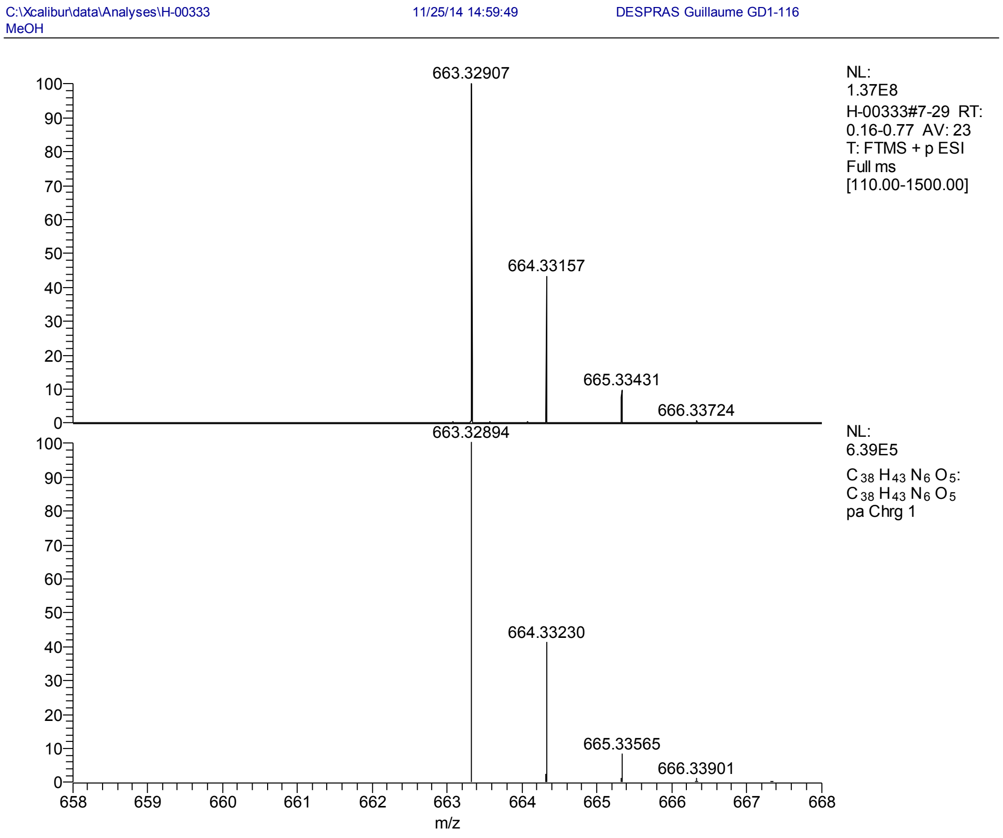

HRMS spectrum of **HR-PN$_3$**

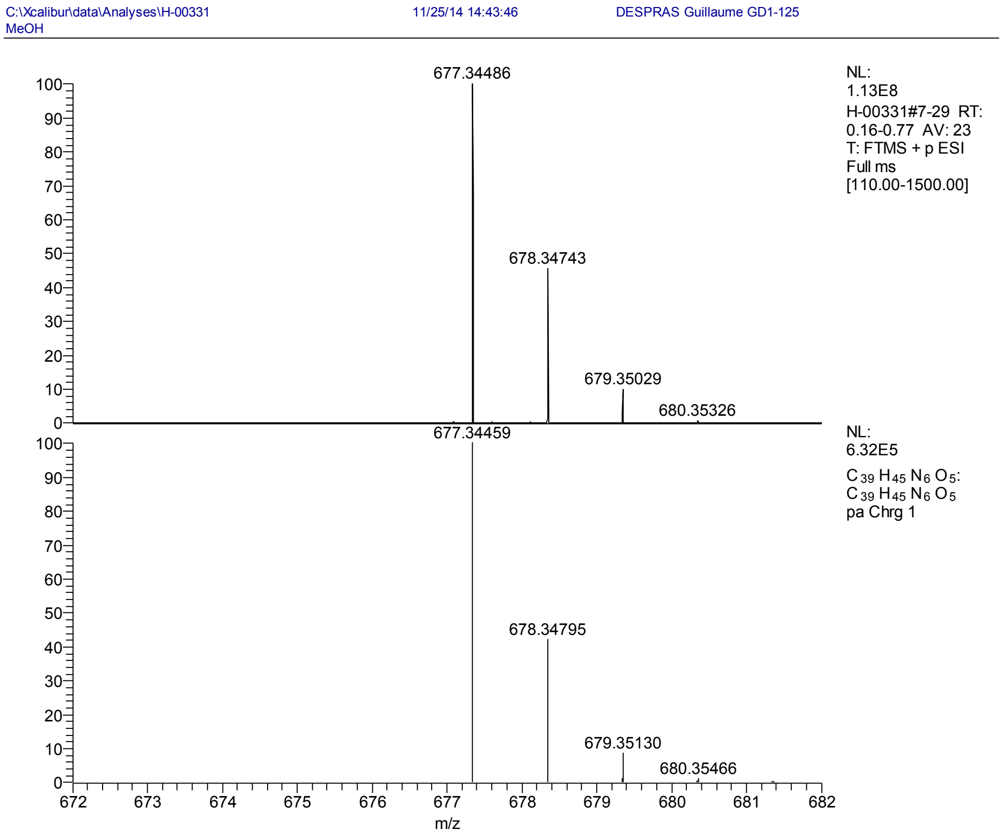

HRMS spectrum of **HR-MPN$_3$**

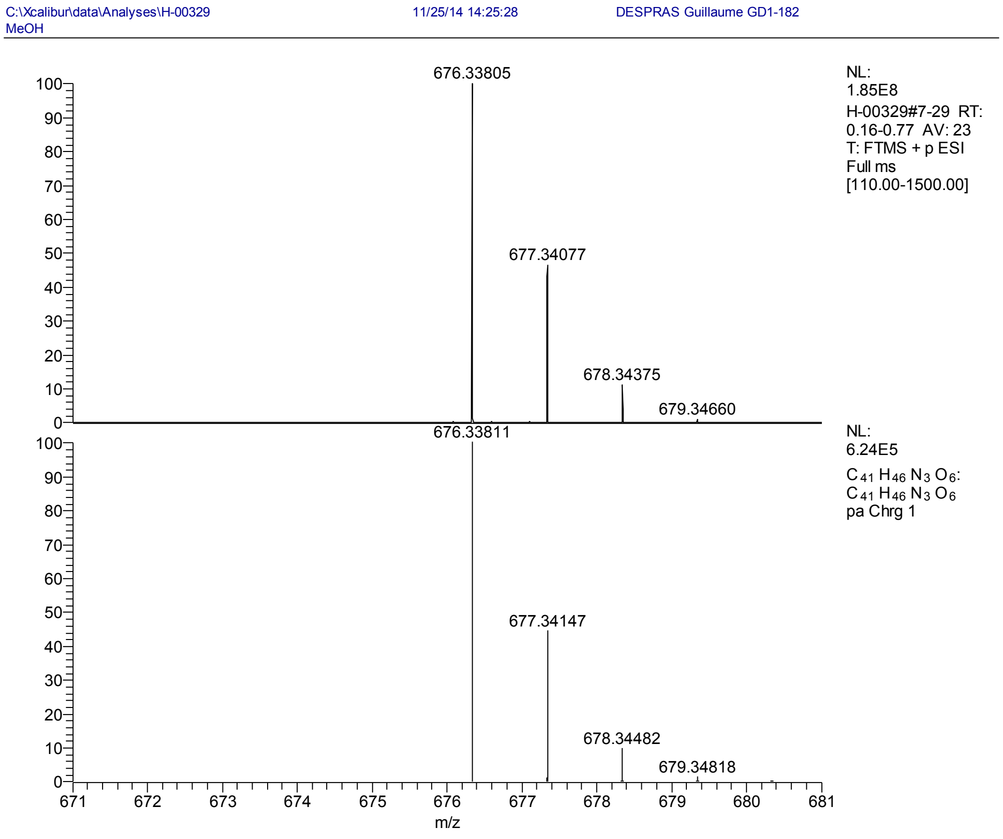

HRMS spectrum of **HR-PA**

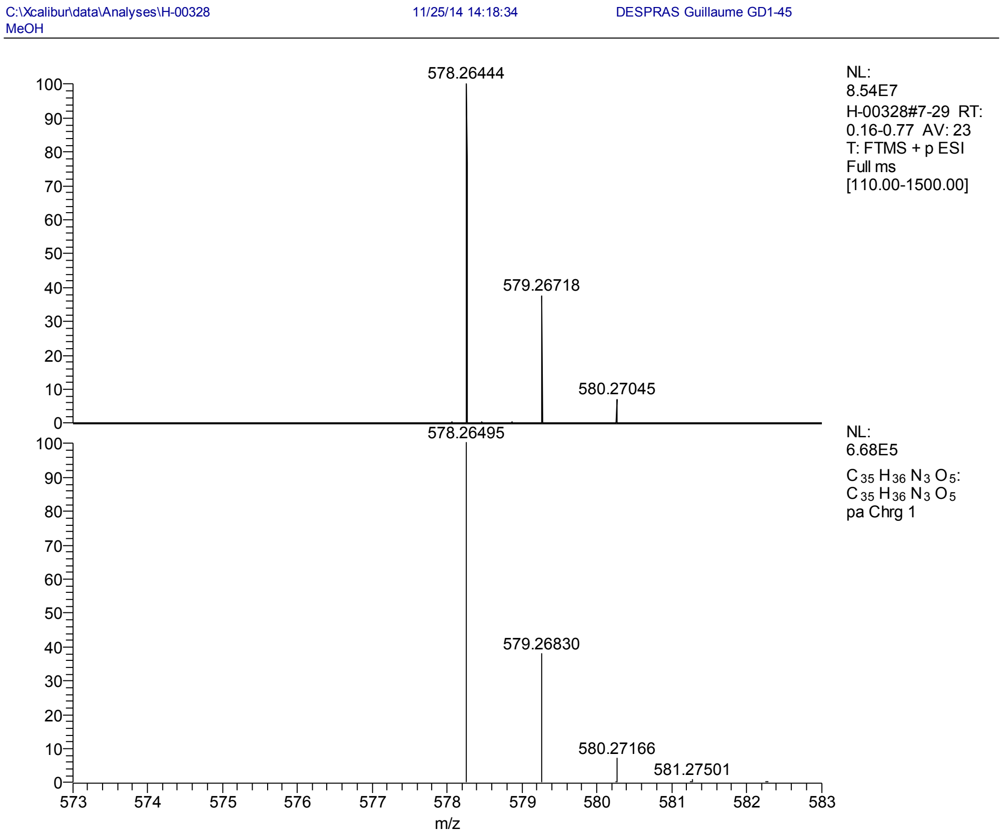

HRMS spectrum of **HR-Ala**

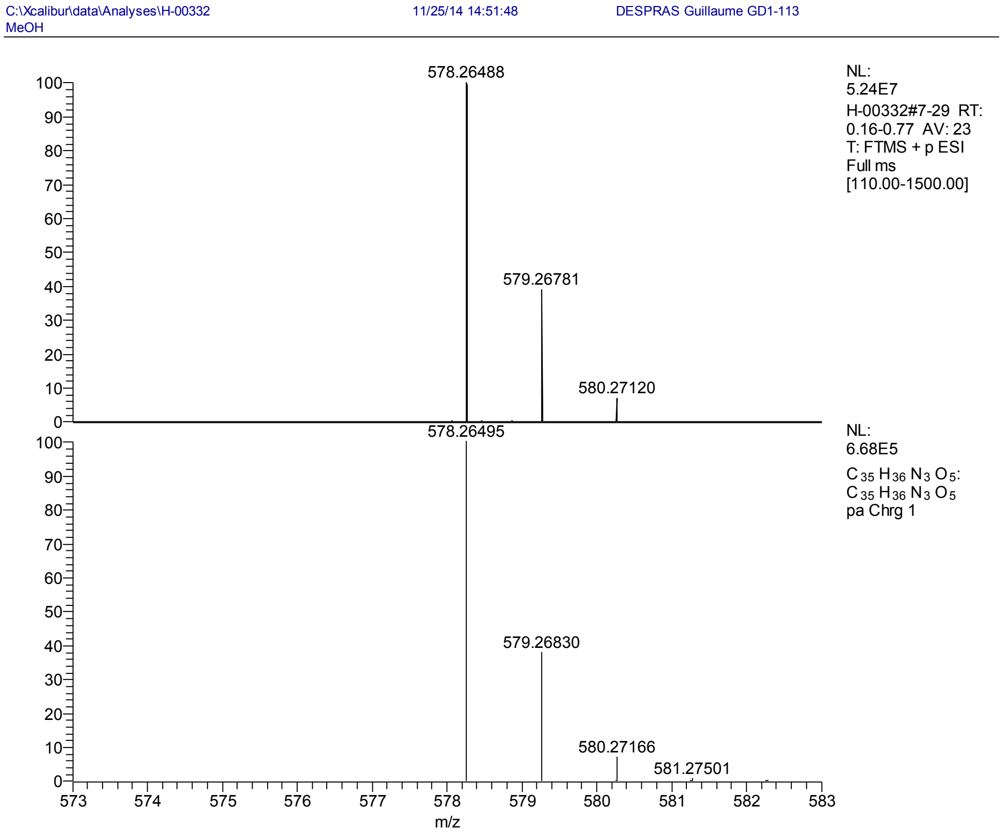

HRMS spectrum of **HR-βAla**

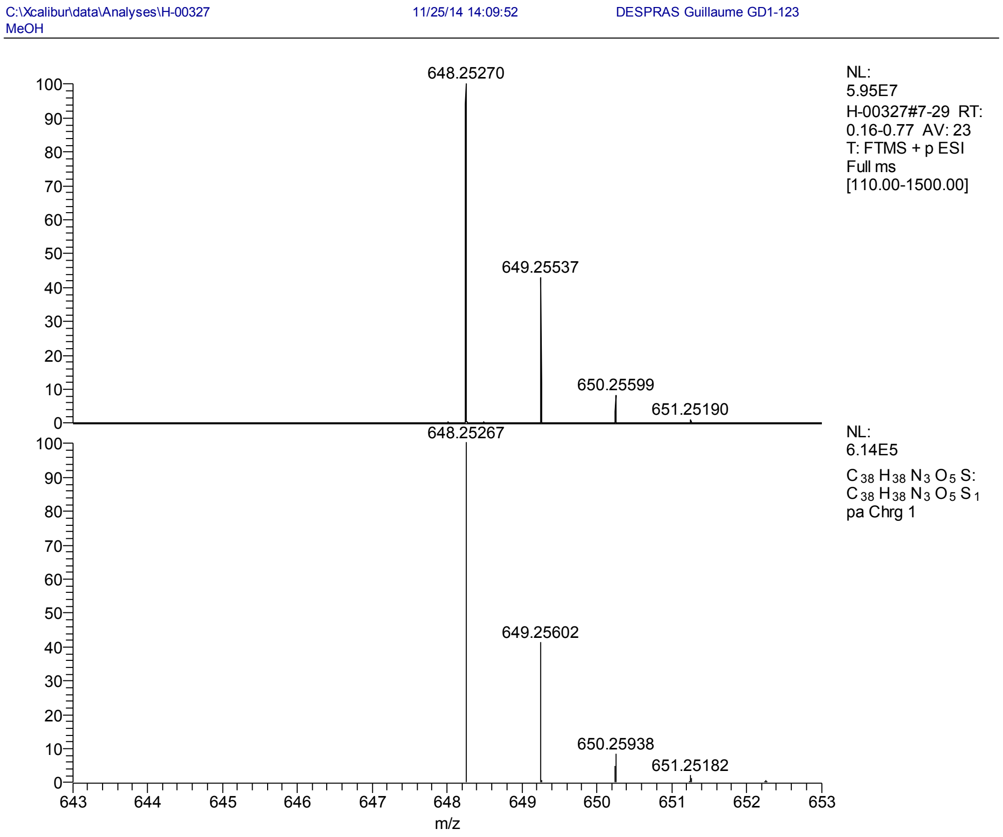

HRMS spectrum of **HR-CysA**

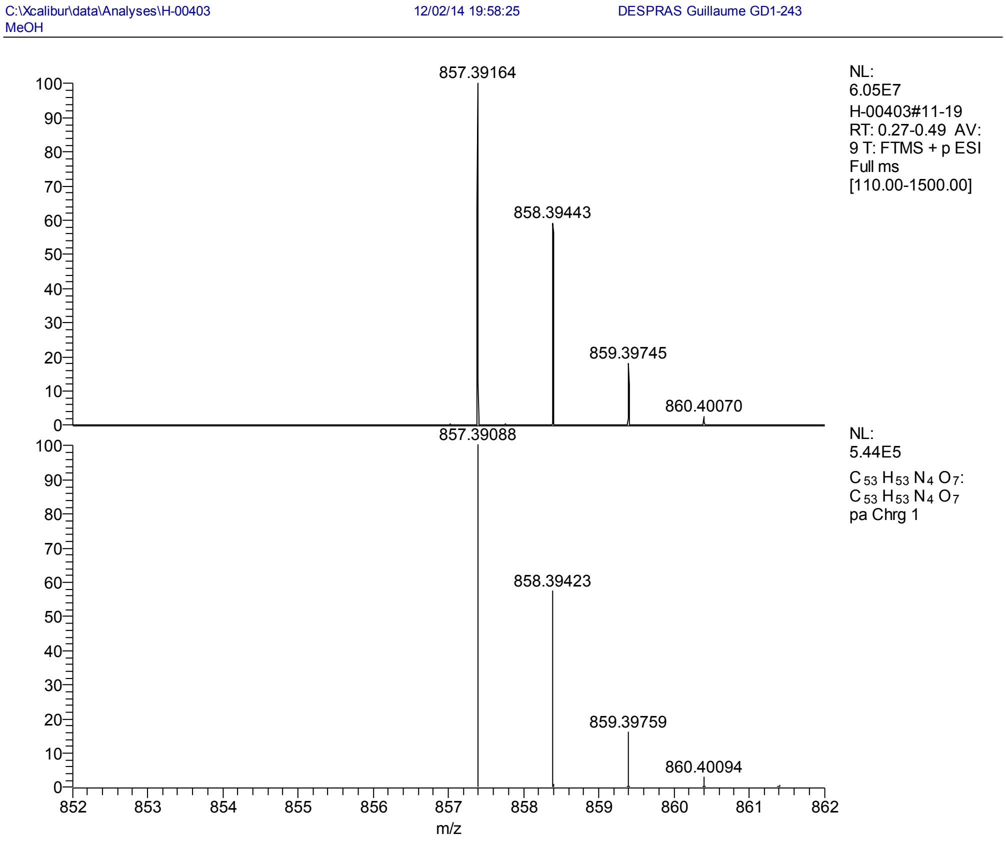

HRMS spectrum of **HR-LysF**

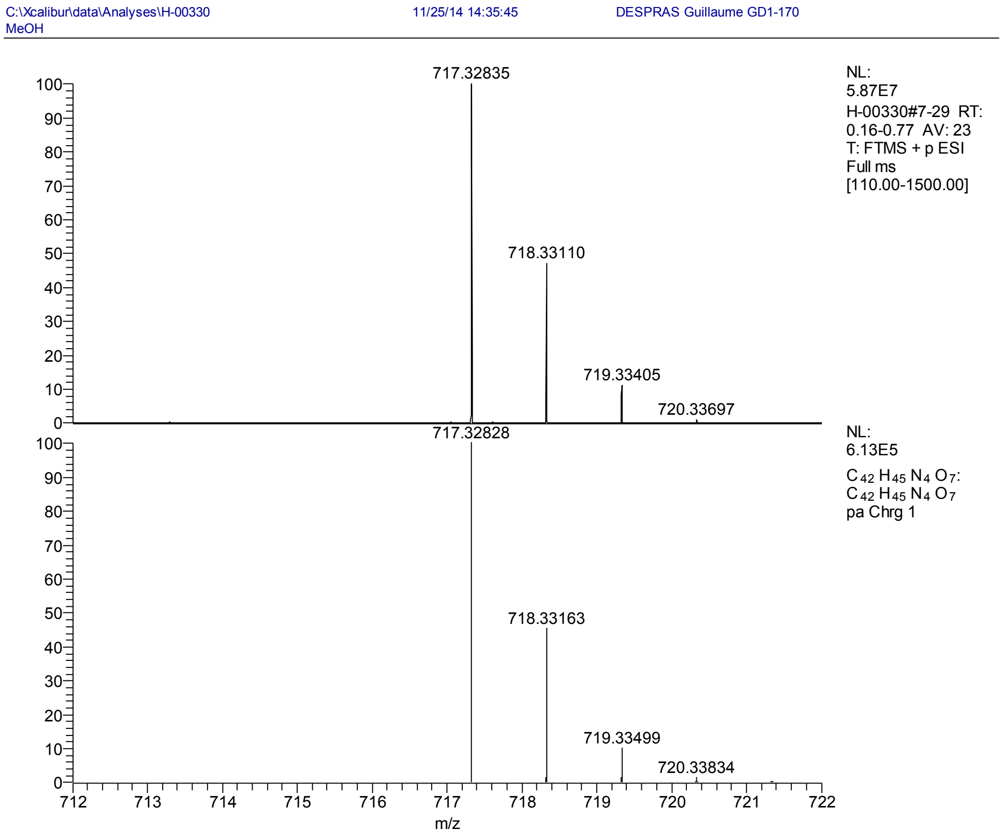

HRMS spectrum of **HR-LysA**
